# Extracellular Vesicles from Human Adipose-Derived Mesenchymal Stem Cells: A Review of Common Cargos

**DOI:** 10.1007/s12015-021-10155-5

**Published:** 2021-04-26

**Authors:** Maria Luz Alonso-Alonso, Laura García-Posadas, Yolanda Diebold

**Affiliations:** 1 Ocular Surface Group, Instituto de Oftalmobiología Aplicada (IOBA), Universidad de Valladolid, Valladolid, Spain; 2 Centro de Investigación Biomédica en Red en el área temática de Bioingeniería, Biomateriales y Nanomedicina (CIBER-BBN), Valladolid, Spain

**Keywords:** Extracellular vesicles, Adipose-derived mesenchymal stem cells, miRNA, Proteomic, Exosome

## Abstract

In recent years, the interest in adipose tissue mesenchymal cell–derived extracellular vesicles (AT-MSC-EVs) has increasingly grown. Numerous articles support the potential of human AT-MSC-EVs as a new therapeutic option for treatment of diverse diseases in the musculoskeletal and cardiovascular systems, kidney, skin, and immune system, among others. This approach makes use of the molecules transported inside of EVs, which play an important role in cell communication and in transmission of macromolecules. However, to our knowledge, there is no database where essential information about AT-MSC-EVs cargo molecules is gathered for easy reference. The aim of this study is to describe the different molecules reported so far in AT-MSC- EVs, their main molecular functions, and biological processes in which they are involved. Recently, the presence of 591 proteins and 604 microRNAs (miRNAs) has been described in human AT-MSC-EVs. The main molecular function enabled by both proteins and miRNAs present in human AT-MSC-EVs is the binding function. Signal transduction and gene silencing are the biological processes in which a greater number of proteins and miRNAs from human AT-MSC-EVs are involved, respectively. In this review we highlight the therapeutics effects of AT-MSC-EVs related with their participation in relevant biological processes including inflammation, angiogenesis, cell proliferation, apoptosis and migration, among others.

**Graphical abstract:**

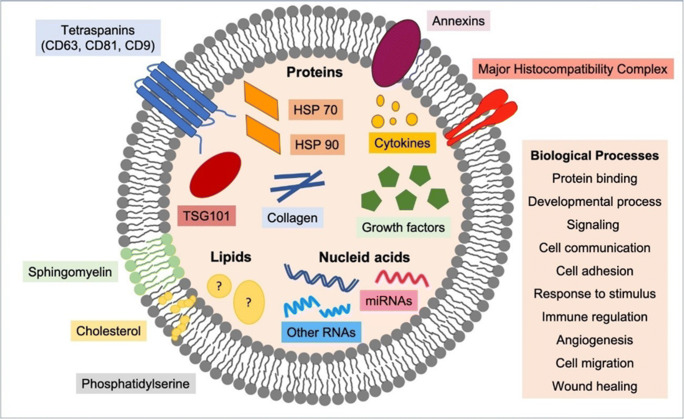

**Supplementary Information:**

The online version contains supplementary material available at 10.1007/s12015-021-10155-5.

## Introduction

“Extracellular vesicle” (EV) is defined by the International Society for Extracellular Vesicles (ISEV) as the “generic term for particles naturally released from the cell that are delimited by a lipid bilayer and cannot replicate, i.e. do not contain a functional nucleus” [1, 2]. These particles contain a significant variety of proteins and RNAs that play important roles in cell-cell communication and in transmission of macromolecules between cells [3–6]. As this feature makes EVs a potential therapeutic approach for various diseases, interest in EV research has significantly increased over the last decade [4, 7]. Importantly, the profile of EV cargo depends on the cell type of origin [8]. In this sense, although a wide range of mammalian cells release EVs [4, 9], mesenchymal stem cells (MSC) are considered one of the most prolific producer cell types [10]. These vesicles are involved in the paracrine properties of MSCs [11–13].

MSCs can be harvested from different tissues, such as bone marrow (BM), adipose tissue (AT), dental pulp, and umbilical cord, among others [14, 15]. BM and AT are the most common sources of MSC for use in research [16–19]. Although BM-MSCs were the first identified MSC [20] type and have been extensively studied [21], AT-MSCs present remarkable advantages by comparison, including higher stability in culture conditions and lower senescence ratio [21]. In addition, the amount of MSC that can be obtained from this tissue, which is usually treated as waste material and discarded [22, 23], is significantly greater than that obtained from BM aspirates [21].

The interest in AT-MSC-EVs has increasingly grown, due to the wide range of AT sources and their relatively easy accessibility [9]. AT-MSC-EVs have been isolated not only from human cells, but also from mouse [24–32], rat [33, 34], pig [35–38], and rabbit [39, 40] cells. The main objective of most published studies on AT-MSC-EVs was to evaluate their potential use as a new therapeutic approach to treat various diseases. Moreover, several of these publications did include an analysis of the molecules transported by the EVs, which is especially relevant to understanding their mechanism of action beyond their observable effects. Taken together, these studies have confirmed the presence of 591 proteins and 604 microRNA (miRNA) in the AT-MSC-EVs. Nevertheless, evaluation of effects of the molecules identified in the cargo focused solely on the disease or tissues under study. However, independent of the specific therapeutic use, the human AT-MSC-EVs are compositionally identical. Therefore, we anticipate that a review collecting together all available information about AT-MSC-EVs cargo and their function will be extremely useful for researchers working in this field.

ISEV recently published a guideline encouraging researchers to report their data to these field-specific databases to detect different studies describing the same molecules [1]. Thus, there is a great need for a well-organised review that collects all relevant information regarding molecules identified so far in AT-MSC-EVs cargo, and their biological activities. This will facilitate future research in this area. Currently, there are two online databases collecting the identified molecules in cargos of EVs derived from different cell types: http://microvesicles.org [41] (formerly http://www.exocarta.org [42]), and http://evpedia.info [43] (link currently unavailable). Both databases are good, reliable sources of information; however, the information available on AT-MSC-EVs cargo is still limited compared to that available on other cell types, such as T cells or prostate cancer cell EV cargos. Thus, this review will provide an updated source not only of identified AT-MSC-EVs cargo molecules, but also their functions and potential therapeutic applications.

Given the growing interest in the MSC-EVs, especially in those derived from AT, the purpose of this study is to provide the AT-MSC research community with a systematic review of publications reporting the cargo of AT-MSC-EVs, including an analysis of their molecular functions and the biological process in which they are involved.

## Methods

A systematic literature search was conducted in the medical databases Pubmed and Web of Science, using the keywords “extracellular vesicles”, “exosome”, “adipose mesenchymal stem cells”, “cargo”, “protein” and “miRNA” without setting a time limit (last searched 6th September 2020). 112 articles published between 2006 and 2020 (inclusive) were reviewed. 48 of these articles were related to human AT-MSC-EV, and 17 to AT-MSC-EVs in other species. The remaining articles were about EVs in general and MSC-EVs from other sources. This study has included both articles that used the nomenclature recommended by ISEV (“EV”) [1] and those which used the terms “exosomes” and “microvesicles”. Given the number of publications that have used these terms during the past decades [2], we considered that the exclusion of them could lead to the loss of relevant information. In addition, although the isolation methods of EVs could have an impact on the cargo composition, it was not an exclusion criterion since there is no single optimal separation method [1].

Different nomenclatures such as adipose stem cells, adipose stromal cells, or adipose-derived stem cells, have been used to identify AT-MSCs. The keyword “adipose mesenchymal stem cells” allowed us to find articles in which authors used several of these nomenclatures. However, we may have missed some information due to this great variety of terms, and this may be a limitation of the present study.

Information regarding proteins (10 articles) and RNA (16 articles) detected in human AT-MSC-EVs was collected in two databases created in Excel (Microsoft Office Excel 2013; Microsoft Corporation, Redmond, WA, USA). Although an article was found in which the lipid content of human AT-MSC-ECs was measured, no more information about lipids was reported. Therefore, it was not possible to include a database of lipids in this review.

To standardise the data and facilitate the recognition of identified proteins, we used the recommended name and identifier code proposed by the Universal Protein Knowledgebase [44] (UniProtKB). This database includes additional information about the short and alternative names for some proteins, which allowed us to identify proteins described by certain authors with these terms. UniProtKB host institutions are the European Bioinformatics Institute (EMBL-EBI), the Swiss Institute of Bioinformatics, and the Protein Information Resource.

For RNA, we used the name of mature micro RNAs (miRNAs) and the code of identification recommended by the RNAcentral database [45] (https://rnacentral.org/). This database is coordinated by EMBL-EBI and integrates information from 41 Expert Databases out of the 53 which constitute the RNAcentral Consortium. In addition, we used the miRBase database [46–51] to classify miRNAs by gene families. miRBase is one of the Expert Databases integrated in the RNAcentral database, and is managed by the University of Manchester. This database also includes information about the previous nomenclature of some miRNAs, which allowed us to correlate the previous miRNA name used by certain authors with the current recommended terminology.

Messenger RNA (mRNA) [52], transfer RNA (tRNA), small ribosomal RNA (rRNA), small nuclear RNA (snRNA), small nucleolar RNA (snoRNA) and small cytoplasmic RNA (scRNA) are also present in AT-MSC-EVs [53, 54]. However, there is less information available on these, therefore, it was possible to include the list of the main tRNAs and mRNA present in AT-MSC-EVs, but not the other types of RNA.

Finally, the web-based tool QuickGO [55] (https://www.ebi.ac.uk/QuickGO/), also managed by EMBL-EBI, was used to search the gene ontology (GO) terms of molecular functions and biological processes of detected proteins and miRNAs. An ontology consists of a set of specific concepts with well-defined relationships between them. The GO was developed by the GO Consortium, as a tool to unify the terminology used to describe the functions of genes and gene products [56].

## Cargo of AT-MSC-EVs

Human AT-MSC-EVs transport different types of proteins [12, 52, 57–65], RNAs [11, 12, 53, 54, 59, 64–74] and lipids [58]. Due to this variety of cargo molecules, AT-MSC-EVs are involved in a wide range of biological functions including migration, immune regulation, cell proliferation, angiogenesis, osteocyte metabolism and nerve regeneration (for a comprehensive review see ref. 9) [9]. Their therapeutic potential is being tested for the treatment of diverse diseases in musculoskeletal [12, 52, 57, 65–67, 75–78] and cardiovascular systems [60, 72, 79–81], nephrology [82, 83], skin [62, 68, 84–86] and immunology [71, 87], among others.

Surprisingly, we could only find one published study about the potential of human AT-MSC-EVs for the treatment of eye diseases [88], despite the fact that human AT-MSC and their conditioned media are being used in ophthalmology [89–99]. For instance, they are being used in 6 out of 403 registered clinical trials with these cells (ClinicalTrials.gov, NCT04484402 NCT03878628, NCT02932852, NCT01808378, NCT02144103 and NCT02024269). In this study, human AT-MSC-EVs showed a protective effect both in vitro and in vivo in a mouse model of dry eye by suppressing the NLRP3 (NOD-like receptor family) inflammasome activation [88]. Moreover, the positive effects of mouse and rabbit AT-MSC-EVs have been demonstrated in in vivo models of laser-induced retinal injury [29] and diabetic retinopathy [40], respectively. In addition, rabbit AT-MSC-EVs seemed to take part in the viability regulation of cultured rabbit corneal stromal cells [39]. There are also several studies which have used human BM-MSC-EVs in ophthalmology, showing their beneficial effects in rat retinal and retinal ganglion cell cultures [100, 101] and in animal models of glaucoma [102, 103] and optic nerve crush [101]. As well as AT-MSC, BM-MSC have also been widely used in ophthalmology [104–113], including 8 out of 293 registered clinical trials with these cells (ClinicalTrials.gov, NCT01531348, NCT01562002 [114], NCT01920867 [115, 116], NCT02325843, NCT02330978, NCT03011541 [117], NCT03173638 and NCT03967275).

In the present review, we comprehensively describe the GO annotations of molecular functions and biological processes of each type of cargo reported in human AT-MSC-EVs.

### Proteins

Proteomic analysis of EV cargo can enhance the knowledge of the functions and mechanisms of action in which these vesicles are involved [28]. To analyse AT-MSC-EVs protein content, researchers used a large variety of techniques such as mass spectrometry [12, 57, 59], antibody arrays [52, 60, 61, 65], Western Blotting [62, 63] and, to a lesser extent, rate immune nephelometry [58]. The EVs in those studies have been isolated by ultracentrifugation [12, 52, 57, 60, 65], filtration and ultracentrifugation [61, 63], commercial EV isolation kits [62], ultrafiltration [58], and affinity purification [59].

So far, 591 proteins have been identified (Table 1). Nevertheless, taking into account both the name and the gene or NCBI Reference Sequences mentioned in the articles, it was not possible to connect the proteins C-peptide, HCR/CRAM-A/B [52, 65], INSL3, macroglobulin [65], CA 19–9, MSHa, PPARg2, TGF-beta 5 and TRA-1-60/TRA-1-81, Pepsinogen I [52] with an UniprotKB code conclusively (Table 1). The presence of the protein families annexin, HSP 70 and HSP 90 has also been described [12] (Table 1). However, as the specific members of these three families were not reported, it was not possible to include them in the GO analyses.

**Table 1:** Proteins detected in human AT-MSC-EVs in alphabetical order

Protein	Abbreviation	UniProtKB	Gene	Ref.
5’-AMP-activated protein kinase catalytic subunit alpha-1*	AAPK1_HUMAN	Q13131	PRKAA1	[65]
72 kDa type IV collagenase*	MMP2_HUMAN	P08253	MMP2	[52]
A disintegrin and metalloproteinase with thrombospondin motifs 1*	ATS1_HUMAN	Q9UHI8	ADAMTS1	[65]
A disintegrin and metalloproteinase with thrombospondin motifs 2*	ATS2_HUMAN	O95450	ADAMTS2	[65]
A disintegrin and metalloproteinase with thrombospondin motifs 4*	ATS4_HUMAN	O75173	ADAMTS4	[52, 65]
A disintegrin and metalloproteinase with thrombospondin motifs 17*	ATS17_HUMAN	Q8TE56	ADAMTS17	[52]
A disintegrin and metalloproteinase with thrombospondin motifs 18*	ATS18_HUMAN	Q8TE60	ADAMTS18	[65]
A disintegrin and metalloproteinase with thrombospondin motifs 19*	ATS19_HUMAN	Q8TE59	ADAMTS19	[52, 65]
Acidic fibroblast growth factor intracellular-binding protein	FIBP_HUMAN	O43427	FIBP	[57]
Activated CDC42 kinase 1*	ACK1_HUMAN	Q07912	TNK2	[52, 65]
Activin receptor type-1B*	ACV1B_HUMAN	P36896	ACVR1B	[65]
Activin receptor type-2B*	AVR2B_HUMAN	Q13705	ACVR2B	[65]
Adenomatous polyposis coli protein*	APC_HUMAN	P25054	APC	[52, 65]
Adhesion G protein-coupled receptor B1*	AGRB1_HUMAN	O14514	ADGRB1	[52]
Adhesion G protein-coupled receptor E5*	AGRE5_HUMAN	P48960	ADGRE5	[52]
ADP-ribosyl cyclase/cyclic ADP-ribose hydrolase 1*	CD38_HUMAN	P28907	CD38	[65]
Agouti-related protein*	AGRP_HUMAN	O00253	AGRP	[52, 65]
Alkaline phosphatase, placental type*	PPB1_HUMAN	P05187	ALPP	[52]
Alpha-1-acid glycoprotein 1*	A1AG1_HUMAN	P02763	ORM1	[65]
Alpha-1-antitrypsin	A1AT_HUMAN	P01009	SERPINA1	[58]
Alpha-1B-glycoprotein*	A1BG_HUMAN	P04217	A1BG	[52, 65]
Alpha-fetoprotein*	FETA_HUMAN	P02771	AFP	[52, 59]
Alpha-lactalbumin	LALBA_HUMAN	P00709	LALBA	[52, 65]
Aminopeptidase N*	AMPN_HUMAN	P15144	ANPEP	[65]
Amphiregulin	AREG_HUMAN	P15514	AREG	[60, 65]
Angiopoietin-1	ANGP1_HUMAN	Q15389	ANGPT1	[52, 61, 65]
Angiopoietin-1 receptor*	TIE2_HUMAN	Q02763	TEK	[61]
Angiopoietin-4	ANGP4_HUMAN	Q9Y264	ANGPT4	[65]
Angiopoietin-related protein 1*	ANGL1_HUMAN	O95841	ANGPTL1	[52]
Angiopoietin-related protein 2*	ANGL2_HUMAN	Q9UKU9	ANGPTL2	[65]
Angiopoietin-related protein 7*	ANGL7_HUMAN	O43827	ANGPTL7	[60]
Angiostatin (cleaved from plasminogen)	PLMN_HUMAN	P00747	PLG	[52, 60, 61]
Annexin**	–	–	–	[12]
Annexin A5	ANXA5_HUMAN	P08758	ANXA5	[59]
Annexin A7	ANXA7_HUMAN	P20073	ANXA7	[65]
Antileukoproteinase*	SLPI_HUMAN	P03973	SLPI	[52]
Apelin receptor*	APJ_HUMAN	P35414	APLNR	[60]
Apolipoprotein A-IV*	APOA4_HUMAN	P06727	APOA4	[52, 65]
Apolipoprotein B-100*	APOB_HUMAN	P04114	APOB	[59, 65]
Apolipoprotein C-I*	APOC1_HUMAN	P02654	APOC1	[65]
Apolipoprotein C-II*	APOC2_HUMAN	P02655	APOC2	[65]
Apolipoprotein E*	APOE_HUMAN	P02649	APOE	[65]
Apolipoprotein M*	APOM_HUMAN	O95445	APOM	[65]
Apoptosis regulator BAX*	BAX_HUMAN	Q07812	BAX	[52]
Artemin	ARTN_HUMAN	Q5T4W7	ARTN	[52, 60, 65]
Aspartyl/asparaginyl beta-hydroxylase*	ASPH_HUMAN	Q12797	ASPH	[52, 65]
Basal cell adhesion molecule	BCAM_HUMAN	P50895	BCAM	[57]
BCL2/adenovirus E1B 19 kDa protein-interacting protein 2*	BNIP2_HUMAN	Q12982	BNIP2	[52, 65]
Beta-2-microglobulin*	B2MG_HUMAN	P61769	B2M	[65]
Beta-Ala-His dipeptidase*	CNDP1_HUMAN	Q96KN2	CNDP1	[52, 65]
Beta-defensin 1*	DEFB1_HUMAN	P60022	DEFB1	[52]
Beta-defensin 4A	DFB4A_HUMAN	O15263	DEFB4A	[65]
Beta-endorphin (Pro-opiomelanocortin)*	COLI_HUMAN	P01189	POMC	[52, 65]
BMP-binding endothelial regulator protein*	BMPER_HUMAN	Q8N8U9	BMPER	[52, 60, 65]
Bone morphogenetic protein 1	BMP1_HUMAN	P13497	BMP1	[57]
Bone morphogenetic protein 3*	BMP3_HUMAN	P12645	BMP3	[65]
Bone morphogenetic protein 4*	BMP4_HUMAN	P12644	BMP4	[52, 65]
Bone morphogenetic protein 5*	BMP5_HUMAN	P22003	BMP5	[52]
Bone morphogenetic protein 6*	BMP6_HUMAN	P22004	BMP6	[65]
Bone morphogenetic protein 7*	BMP7_HUMAN	P18075	BMP7	[52, 65]
Bone morphogenetic protein 8B*	BMP8B_HUMAN	P34820	BMP8B	[52]
Bone morphogenetic protein receptor type-1A	BMR1A_HUMAN	P36894	BMPR1A	[57]
Bone morphogenetic protein receptor type-1B*	BMR1B_HUMAN	O00238	BMPR1B	[65]
Bone morphogenetic protein receptor type-2	BMPR2_HUMAN	Q13873	BMPR2	[57]
Brain-derived neurotrophic factor*	BDNF_HUMAN	P23560	BDNF	[65]
CA 19–9	–	–	ST6GALNAC (partly synthesized by)	[52]
Cadherin-1	CADH1_HUMAN	P12830	CDH1	[57]
Cadherin-2	CADH2_HUMAN	P19022	CDH2	[57]
Cadherin-5	CADH5_HUMAN	P33151	CDH5	[57]
Cadherin-11	CAD11_HUMAN	P55287	CDH11	[57]
Cadherin-13	CAD13_HUMAN	P55290	CDH13	[57]
Cadherin-related family member 2	CDHR2_HUMAN	Q9BYE9	CDHR2	[57]
Cadherin-related family member 5	CDHR5_HUMAN	Q9HBB8	CDHR5	[57]
Calbindin	CALB1_HUMAN	P05937	CALB1	[52, 65]
Calcitonin	CALC_HUMAN	P01258	CALCA	[52]
Calreticulin	CALR_HUMAN	P27797	CALR	[65]
Calsyntenin-1	CSTN1_HUMAN	O94985	CLSTN1	[65]
Carboxypeptidase N subunit 2*	CPN2_HUMAN	P22792	CPN2	[52, 65]
Carcinoembryonic antigen-related cell adhesion molecule 7*	CEAM7_HUMAN	Q14002	CEACAM7	[65]
Caspase-3	CASP3_HUMAN	P42574	CASP3	[65]
Caspase-8	CASP8_HUMAN	Q14790	CASP8	[52]
Cathepsin B	CATB_HUMAN	P07858	CTSB	[65]
Cathepsin D	CATD_HUMAN	P07339	CTSD	[65]
C-C chemokine receptor type 1*	CCR1_HUMAN	P32246	CCR1	[65]
C-C chemokine receptor type 2*	CCR2_HUMAN	P41597	CCR2	[65]
C-C chemokine receptor type 3*	CCR3_HUMAN	P51677	CCR3	[52]
C-C chemokine receptor type 4*	CCR4_HUMAN	P51679	CCR4	[65]
C-C chemokine receptor type 5*	CCR5_HUMAN	P51681	CCR5	[65]
C-C chemokine receptor type 6*	CCR6_HUMAN	P51684	CCR6	[65]
C-C chemokine receptor type 7*	CCR7_HUMAN	P32248	CCR7	[65]
C-C chemokine receptor type 9*	CCR9_HUMAN	P51686	CCR9	[65]
C-C motif chemokine 1*	CCL1_HUMAN	P22362	CCL1	[61, 65]
C-C motif chemokine 2*	CCL2_HUMAN	P13500	CCL2	[52]
C-C motif chemokine 3*	CCL3_HUMAN	P10147	CCL3	[65]
C-C motif chemokine 4*	CCL4_HUMAN	P13236	CCL4	[52]
C-C motif chemokine 5*	CCL5_HUMAN	P13501	CCL5	[65]
C-C motif chemokine 7*	CCL7_HUMAN	P80098	CCL7	[61]
C-C motif chemokine 8*	CCL8_HUMAN	P80075	CCL8	[61, 65]
C-C motif chemokine 13*	CCL13_HUMAN	Q99616	CCL13	[61, 65]
C-C motif chemokine 14*	CCL14_HUMAN	Q16627	CCL14	[52, 60, 65]
C-C motif chemokine 16*	CCL16_HUMAN	O15467	CCL16	[65]
C-C motif chemokine 18*	CCL18_HUMAN	P55774	CCL18	[52]
C-C motif chemokine 19*	CCL19_HUMAN	Q99731	CCL19	[52]
C-C motif chemokine 21*	CCL21_HUMAN	O00585	CCL21	[65]
C-C motif chemokine 22*	CCL22_HUMAN	O00626	CCL22	[65]
C-C motif chemokine 26*	CCL26_HUMAN	Q9Y258	CCL26	[65]
C-C motif chemokine 27*	CCL27_HUMAN	Q9Y4X3	CCL27	[52]
C-C motif chemokine 28*	CCL28_HUMAN	Q9NRJ3	CCL28	[52, 60]
CD166 antigen	CD166_HUMAN	Q13740	ALCAM	[52, 65]
CD27 antigen	CD27_HUMAN	P26842	CD27	[65]
CD44 antigen	CD44_HUMAN	P16070	CD44	[12, 57, 65]
CD59 glycoprotein*	CD59_HUMAN	P13987	CD59	[52]
CD63 antigen	CD63_HUMAN	P08962	CD63	[12]
Cdc42-interacting protein 4	CIP4_HUMAN	Q15642	TRIP10	[57]
Cell division control protein 42 homolog	CDC42_HUMAN	P60953	CDC42	[57]
Cerberus	CER1_HUMAN	O95813	CER1	[65]
Ceruloplasmin	CERU_HUMAN	P00450	CP	[52, 65]
Chitinase-3-like protein 1*	CH3L1_HUMAN	P36222	CHI3L1	[52, 65]
Chordin-like protein 2*	CRDL2_HUMAN	Q6WN34	CHRDL2	[52]
Ciliary neurotrophic factor receptor subunit alpha*	CNTFR_HUMAN	P26992	CNTFR	[52]
Ciliary neurotrophic factor*	CNTF_HUMAN	P26441	CNTF	[52, 65]
Clusterin	CLUS_HUMAN	P10909	CLU	[52]
Coagulation factor XIII A chain	F13A_HUMAN	P00488	F13A1	[52]
Coagulation factor XIII B chain	F13B_HUMAN	P05160	F13B	[65]
Collagen alpha-1(I) chain	CO1A1_HUMAN	P02452	COL1A1	[57]
Collagen alpha-1(III) chain	CO3A1_HUMAN	P02461	COL3A1	[57]
Collagen alpha-1(IV) chain	CO4A1_HUMAN	P02462	COL4A1	[57]
Collagen alpha-1(V) chain	CO5A1_HUMAN	P20908	COL5A1	[57]
Collagen alpha-1(VI) chain	CO6A1_HUMAN	P12109	COL6A1	[57]
Collagen alpha-1(VII) chain	CO7A1_HUMAN	Q02388	COL7A1	[57]
Collagen alpha-1(XII) chain	COCA1_HUMAN	Q99715	COL12A1	[57]
Collagen alpha-1(XV) chain	COFA1_HUMAN	P39059	COL15A1	[57]
Collagen alpha-2(I) chain	CO1A2_HUMAN	P08123	COL1A2	[57]
Collagen alpha-2(IV) chain	CO4A2_HUMAN	P08572	COL4A2	[57]
Collagen alpha-2(V) chain	CO5A2_HUMAN	P05997	COL5A2	[57]
Collagen alpha-2(VI) chain	CO6A2_HUMAN	P12110	COL6A2	[57]
Collagen alpha-3(VI) chain	CO6A3_HUMAN	P12111	COL6A3	[57]
Collagenase 3*	MMP13_HUMAN	P45452	MMP13	[65]
Complement C2*	CO2_HUMAN	P06681	C2	[52, 65]
Complement C3*	CO3_HUMAN	P01024	C3	[65]
Complement C5*	CO5_HUMAN	P01031	C5	[65]
Complement factor H-related protein 2*	FHR2_HUMAN	P36980	CFHR2	[65]
Corticosteroid 11-beta-dehydrogenase isozyme 1*	DHI1_HUMAN	P28845	HSD11B1	[65]
Corticosteroid-binding globulin	CBG_HUMAN	P08185	SERPINA6	[52]
C-peptide***	–	–	INS	[52, 65]
C-reactive protein*	CRP_HUMAN	P02741	CRP	[65]
Creatine kinase B-type*	KCRB_HUMAN	P12277	CKB	[52, 65]
CREB-binding protein*	CBP_HUMAN	Q92793	CREBBP	[52]
Cryptic protein	CFC1_HUMAN	P0CG37	CFC1	[52, 65]
C-X-C chemokine receptor type 6*	CXCR6_HUMAN	O00574	CXCR6	[65]
C-X-C motif chemokine 2*	CXCL2_HUMAN	P19875	CXCL2	[52, 60, 65]
C-X-C motif chemokine 5*	CXCL5_HUMAN	P42830	CXCL5	[65]
C-X-C motif chemokine 9*	CXCL9_HUMAN	Q07325	CXCL9	[52]
C-X-C motif chemokine 10*	CXL10_HUMAN	P02778	CXCL10	[65]
C-X-C motif chemokine 11*	CXL11_HUMAN	O14625	CXCL11	[61, 65]
C-X-C motif chemokine 16*	CXL16_HUMAN	Q9H2A7	CXCL16	[61, 65]
Cyclin-dependent kinase inhibitor 1*	CDN1A_HUMAN	P38936	CDKN1A	[65]
Cystatin A	CYTA_HUMAN	P01040	CSTA	[65]
Cytokine receptor common subunit gamma*	IL2RG_HUMAN	P31785	IL2RG	[52, 65]
Cytoplasmic tyrosine-protein kinase BMX*	BMX_HUMAN	P51813	BMX	[65]
Cytotoxic and regulatory T cell molecule*	CRTAM_HUMAN	O95727	CRTAM	[65]
Cytotoxic T lymphocyte protein 4*	CTLA4_HUMAN	P16410	CTLA4	[52, 65]
DAN domain family member 5*	DAND5_HUMAN	Q8N907	DAND5	[65]
Decorin	PGS2_HUMAN	P07585	DCN	[65]
Dentin matrix acidic phosphoprotein 1*	DMP1_HUMAN	Q13316	DMP1	[65]
Dermcidin	DCD_HUMAN	P81605	DCD	[59]
Dickkopf-related protein 1*	DKK1_HUMAN	O94907	DKK1	[65]
Dickkopf-related protein 3*	DKK3_HUMAN	Q9UBP4	DKK3	[65]
Dickkopf-related protein 4*	DKK4_HUMAN	Q9UBT3	DKK4	[52]
Discoidin domain-containing receptor 2*	DDR2_HUMAN	Q16832	DDR2	[52]
Discoidin, CUB and LCCL domain-containing protein 2*	DCBD2_HUMAN	Q96PD2	DCBLD2	[65]
Echinoderm microtubule-associated protein-like 2*	EMAL2_HUMAN	O95834	EML2	[52, 65]
Ectodysplasin-A*	EDA_HUMAN	Q92838	EDA	[60, 65]
Ectonucleotide pyrophosphatase/ phosphodiesterase family member 2*	ENPP2_HUMAN	Q13822	ENPP2	[52]
EGF-like repeat and discoidin I-like domain-containing protein 3	EDIL3_HUMAN	O43854	EDIL3	[57]
Elongation factor 1-alpha 1	EF1A1_HUMAN	P68104	EEF1A1	[12]
Elongation factor 2*	EF2_HUMAN	P13639	EEF2	[12]
Embryonic growth/differentiation factor 1*	GDF1_HUMAN	P27539	GDF1	[52]
Endoglin	EGLN_HUMAN	P17813	ENG	[52]
Endostatin (cleaved from Collagen alpha-1(XVIII) chain)	COIA1_HUMAN	P39060	COL18A1	[52, 57, 60, 65]
Endothelial cell-selective adhesion molecule*	ESAM_HUMAN	Q96AP7	ESAM	[65]
Endothelin-1 receptor*	EDNRA_HUMAN	P25101	EDNRA	[52, 65]
Eotaxin	CCL11_HUMAN	P51671	CCL11	[65]
Ephrin type-A receptor 4*	EPHA4_HUMAN	P54764	EPHA4	[52]
Ephrin type-A receptor 6*	EPHA6_HUMAN	Q9UF33	EPHA6	[65]
Ephrin type-A receptor 8*	EPHA8_HUMAN	P29322	EPHA8	[65]
Ephrin type-B receptor 4*	EPHB4_HUMAN	P54760	EPHB4	[65]
Epidermal growth factor receptor*	EGFR_HUMAN	P00533	EGFR	[57, 65]
Epidermal growth factor receptor substrate 15-like 1	EP15R_HUMAN	Q9UBC2	EPS15L1	[57]
Epithelial cell adhesion molecule*	EPCAM_HUMAN	P16422	EPCAM	[65]
Erythropoietin	EPO_HUMAN	P01588	EPO	[52]
Erythropoietin receptor	EPOR_HUMAN	P19235	EPOR	[65]
E-Selectin	LYAM2_HUMAN	P16581	SELE	[52]
EVI5-like protein	EVI5L_HUMAN	Q96CN4	EVI5L	[52]
FAS-associated death domain protein*	FADD_HUMAN	Q13158	FADD	[65]
Fatty acid-binding protein 5	FABP5_HUMAN	Q01469	FABP5	[59]
Ferritin light chain*	FRIL_HUMAN	P02792	FTL	[65]
Fetuin-B	FETUB_HUMAN	Q9UGM5	FETUB	[65]
Fibrinogen-like protein 1*	FGL1_HUMAN	Q08830	FGL1	[52, 65]
Fibrinopeptide A (cleaved from Fibrinogen alpha chain)	FIBA_HUMAN	P02671	FGA	[52]
Fibroblast growth factor 2*	FGF2_HUMAN	P09038	FGF2	[57, 65]
Fibroblast growth factor 4*	FGF4_HUMAN	P08620	FGF4	[61]
Fibroblast growth factor 5*	FGF5_HUMAN	P12034	FGF5	[52]
Fibroblast growth factor 6*	FGF6_HUMAN	P10767	FGF6	[65]
Fibroblast growth factor 8*	FGF8_HUMAN	P55075	FGF8	[65]
Fibroblast growth factor 10*	FGF10_HUMAN	O15520	FGF10	[52]
Fibroblast growth factor 11*	FGF11_HUMAN	Q92914	FGF11	[52]
Fibroblast growth factor 12*	FGF12_HUMAN	P61328	FGF12	[65]
Fibroblast growth factor 13*	FGF13_HUMAN	Q92913	FGF13	[52]
Fibroblast growth factor 16*	FGF16_HUMAN	O43320	FGF16	[52]
Fibroblast growth factor 17*	FGF17_HUMAN	O60258	FGF17	[52, 65]
Fibroblast growth factor 18*	FGF18_HUMAN	O76093	FGF18	[52, 65]
Fibroblast growth factor 20*	FGF20_HUMAN	Q9NP95	FGF20	[52, 65]
Fibroblast growth factor 21*	FGF21_HUMAN	Q9NSA1	FGF21	[65]
Fibroblast growth factor receptor 1	FGFR1_HUMAN	P11362	FGFR1	[57]
Fibroblast growth factor receptor 3*	FGFR3_HUMAN	P22607	FGFR3	[65]
Fibroblast growth factor receptor 4	FGFR4_HUMAN	P22455	FGFR4	[57]
Fibroblast growth factor-binding protein 1*	FGFP1_HUMAN	Q14512	FGFBP1	[65]
Fibronectin	FINC_HUMAN	P02751	FN1	[52, 57]
Filaggrin-2	FILA2_HUMAN	Q5D862	FLG2	[59]
Follistatin	FST_HUMAN	P19883	FST	[52, 61, 65]
Follistatin-related protein 3*	FSTL3_HUMAN	O95633	FSTL3	[65]
Forkhead box protein N3*	FOXN3_HUMAN	O00409	FOXN3	[52]
Frizzled-1	FZD1_HUMAN	Q9UP38	FZD1	[52, 57, 65]
Frizzled-3	FZD3_HUMAN	Q9NPG1	FZD3	[52, 65]
Frizzled-6	FZD6_HUMAN	O60353	FZD6	[57]
Frizzled-7	FZD7_HUMAN	O75084	FZD7	[65]
Fructose-bisphosphate aldolase A*	ALDOA_HUMAN	P04075	ALDOA	[52]
Fructose-bisphosphate aldolase B	ALDOB_HUMAN	P05062	ALDOB	[65]
Fructose-bisphosphate aldolase C*	ALDOC_HUMAN	P09972	ALDOC	[52, 65]
Furin	FURIN_HUMAN	P09958	FURIN	[65]
Galanin peptides	GALA_HUMAN	P22466	GAL	[52]
Galectin-10*	LEG10_HUMAN	Q05315	CLC	[52, 65]
Galectin-3	LEG3_HUMAN	P17931	LGALS3	[52, 65]
Gamma-Thrombin (cleaved from prothrombin)	THRB_HUMAN	P00734	F2	[65]
GATA-type zinc finger protein 1*	ZGLP1_HUMAN	P0C6A0	ZGLP1	[52]
GDNF family receptor alpha-3*	GFRA3_HUMAN	O60609	GFRA3	[52]
Geminin*	GEMI_HUMAN	O75496	GMNN	[65]
Glial cell line-derived neurotrophic factor*	GDNF_HUMAN	P39905	GDNF	[65]
Glutathione peroxidase 1*	GPX1_HUMAN	P07203	GPX1	[65]
Glutathione peroxidase 3*	GPX3_HUMAN	P22352	GPX3	[65]
Glyceraldehyde 3-phosphate dehydrogenase	G3P_HUMAN	P04406	GAPDH	[12]
Glycogen phosphorylase, brain form*	PYGB_HUMAN	P11216	PYGB	[65]
Glycoprotein hormones alpha chain*	GLHA_HUMAN	P01215	CGA	[52]
Glypican-3	GPC3_HUMAN	P51654	GPC3	[60]
Glypican-5	GPC5_HUMAN	P78333	GPC5	[65]
Granulocyte colony-stimulating factor*	CSF3_HUMAN	P09919	CSF3	[52, 60, 61, 65]
Granulocyte-macrophage colony-stimulating factor receptor subunit alpha*	CSF2R_HUMAN	P15509	CSF2RA	[52, 65]
Granulocyte-macrophage colony-stimulating factor*	CSF2_HUMAN	P04141	CSF2	[52, 61]
Granzyme A	GRAA_HUMAN	P12544	GZMA	[52, 65]
Gremlin-1	GREM1_HUMAN	O60565	GREM1	[52]
Growth arrest and DNA damage-inducible protein GADD45 alpha*	GA45A_HUMAN	P24522	GADD45A	[52]
Growth factor receptor-bound protein 2	GRB2_HUMAN	P62993	GRB2	[57]
Growth/differentiation factor 2*	GDF2_HUMAN	Q9UK05	GDF2	[65]
Growth/differentiation factor 3*	GDF3_HUMAN	Q9NR23	GDF3	[52, 65]
Growth/differentiation factor 5*	GDF5_HUMAN	P43026	GDF5	[52, 65]
Growth/differentiation factor 8*	GDF8_HUMAN	O14793	MSTN	[52]
Growth/differentiation factor 9*	GDF9_HUMAN	O60383	GDF9	[52, 65]
Growth/differentiation factor 11*	GDF11_HUMAN	O95390	GDF11	[52, 57, 65]
Guanine nucleotide-binding protein G(I)/G(S)/G(O) subunit gamma-12	GBG12_HUMAN	Q9UBI6	GNG12	[57]
Guanine nucleotide-binding protein subunit alpha-13	GNA13_HUMAN	Q14344	GNA13	[57]
Haptoglobin	HPT_HUMAN	P00738	HP	[52]
HCR / CRAM-A/B***	–	–	CCHCR1	[52, 65]
Heat shock protein 70 kDa**	–	–	–	[12]
Heat shock protein 90 kDa**	–	–	–	[12]
Heat shock protein 105 kDa*	HS105_HUMAN	Q92598	HSPH1	[12]
Heat shock protein beta-1*	HSPB1_HUMAN	P04792	HSPB1_HUMAN	[12, 52, 65]
Hepatocyte growth factor activator	HGFA_HUMAN	Q04756	HGFAC	[57]
Hepatocyte growth factor receptor*	MET_HUMAN	P08581	MET	[52]
Hepatocyte growth factor-like protein alpha chain (cleaved from hepatocyte growth factor-like protein)*	HGFL_HUMAN	P26927	MST1	[52]
Hepatocyte growth factor-regulated tyrosine kinase substrate	HGS_HUMAN	O14964	HGS	[57]
Hepcidin	HEPC_HUMAN	P81172	HAMP	[65]
Histone H4	H4_HUMAN	P62805	H4C1	[59]
HLA class II histocompatibility antigen gamma chain*	HG2A_HUMAN	P04233	CD74	[65]
Homeobox protein NANOG*	NANOG_HUMAN	Q9H9S0	NANOG	[65]
Hornerin	HORN_HUMAN	Q86YZ3	HRNR	[59]
Inhibin beta A chain*	INHBA_HUMAN	P08476	INHBA	[65]
Inhibin beta B chain*	INHBB_HUMAN	P09529	INHBB	[65]
Inhibin beta C chain*	INHBC_HUMAN	P55103	INHBC	[60]
INSL3***	–	–	–	[65]
Insulin receptor*	INSR_HUMAN	P06213	INSR	[52, 65]
Insulin-degrading enzyme*	IDE_HUMAN	P14735	IDE	[65]
Insulin-like growth factor 1 receptor	IGF1R_HUMAN	P08069	IGF1R	[57]
Insulin-like growth factor I*	IGF1_HUMAN	P05019	IGF1	[65]
Insulin-like growth factor-binding protein 1*	IBP1_HUMAN	P08833	IGFBP1	[65]
Insulin-like growth factor-binding protein 3	IBP3_HUMAN	P17936	IGFBP3	[57]
Insulin-like growth factor-binding protein 4*	IBP4_HUMAN	P22692	IGFBP4	[52]
Insulin-like growth factor-binding protein 5*	IBP5_HUMAN	P24593	IGFBP5	[65]
Insulin-like growth factor-binding protein 7*	IBP7_HUMAN	Q16270	IGFBP7	[60, 65]
Insulin-like growth factor-binding protein complex acid labile subunit	ALS_HUMAN	P35858	IGFALS	[57]
Integrin alpha-1	ITA1_HUMAN	P56199	ITGA1	[57]
Integrin alpha-2	ITA2_HUMAN	P17301	ITGA2	[57]
Integrin alpha-3	ITA3_HUMAN	P26006	ITGA3	[57]
Integrin alpha-4	ITA4_HUMAN	P13612	ITGA4	[57]
Integrin alpha-5	ITA5_HUMAN	P08648	ITGA5	[57]
Integrin alpha-6	ITA6_HUMAN	P23229	ITGA6	[57]
Integrin alpha-7	ITA7_HUMAN	Q13683	ITGA7	[57]
Integrin alpha-10	ITA10_HUMAN	O75578	ITGA10	[57]
Integrin alpha-11	ITA11_HUMAN	Q9UKX5	ITGA11	[57]
Integrin alpha-M*	ITAM_HUMAN	P11215	ITGAM	[52]
Integrin alpha-V	ITAV_HUMAN	P06756	ITGAV	[52, 57, 65]
Integrin beta-1	ITB1_HUMAN	P05556	ITGB1	[57]
Integrin beta-1-binding protein 1	ITBP1_HUMAN	O14713	ITGB1BP1	[57]
Integrin beta-3	ITB3_HUMAN	P05106	ITGB3	[57]
Integrin beta-5	ITB5_HUMAN	P18084	ITGB5	[57]
Integrin-linked protein kinase	ILK_HUMAN	Q13418	ILK	[57]
Inter-alpha-trypsin inhibitor heavy chain H2	ITIH2_HUMAN	P19823	ITIH2	[59]
Intercellular adhesion molecule 1	ICAM1_HUMAN	P05362	ICAM1	[57]
Intercellular adhesion molecule 2*	ICAM2_HUMAN	P13598	ICAM2	[57, 65]
Interferon beta*	IFNB_HUMAN	P01574	IFNB1	[65]
Interferon gamma*	IFNG_HUMAN	P01579	IFNG	[52, 65]
Interferon lambda-1*	IFNL1_HUMAN	Q8IU54	IFNL1	[65]
Interferon lambda-2*	IFNL2_HUMAN	Q8IZJ0	IFNL2	[65]
Interferon regulatory factor 6*	IRF6_HUMAN	O14896	IRF6	[52]
Interleukin-1 alpha*	IL1A_HUMAN	P01583	IL1A	[52, 60, 65]
Interleukin-1 beta*	IL1B_HUMAN	P01584	IL1B	[61]
Interleukin-1 family member 10*	IL1FA_HUMAN	Q8WWZ1	IL1F10	[52, 65]
Interleukin-1 receptor accessory protein-like 1*	IRPL1_HUMAN	Q9NZN1	IL1RAPL1	[52, 65]
Interleukin-1 receptor type 1*	IL1R1_HUMAN	P14778	IL1R1	[52]
Interleukin-1 receptor type 2*	IL1R2_HUMAN	P27930	IL1R2	[52]
Interleukin-1 receptor-like 1*	ILRL1_HUMAN	Q01638	IL1RL1	[52]
Interleukin-1 receptor-like 2*	ILRL2_HUMAN	Q9HB29	IL1RL2	[52]
Interleukin-2*	IL2_HUMAN	P60568	IL2	[52]
Interleukin-2 receptor subunit alpha*	IL2RA_HUMAN	P01589	IL2RA	[65]
Interleukin-2 receptor subunit beta*	IL2RB_HUMAN	P14784	IL2RB	[52]
Interleukin-4*	IL4_HUMAN	P05112	IL4	[61]
Interleukin-5*	IL5_HUMAN	P05113	IL5	[52]
Interleukin-6*	IL6_HUMAN	P05231	IL6	[52, 62]
Interleukin-7*	IL7_HUMAN	P13232	IL7	[52, 65]
Interleukin-7 receptor subunit alpha*	IL7RA_HUMAN	P16871	IL7R	[65]
Interleukin-8*	IL8_HUMAN	P10145	CXCL8	[52, 65]
Interleukin-9*	IL9_HUMAN	P15248	IL9	[52, 65]
Interleukin-10*	IL10_HUMAN	P22301	IL10	[52, 61]
Interleukin-10 receptor subunit alpha*	I10R1_HUMAN	Q13651	IL10RA	[52]
Interleukin-11*	IL11_HUMAN	P20809	IL11	[52]
Interleukin-12 subunit alpha*	IL12A_HUMAN	P29459	IL12A	[61]
Interleukin-12 subunit beta*	IL12B_HUMAN	P29460	IL12B	[61]
Interleukin-13 receptor subunit alpha-1*	I13R1_HUMAN	P78552	IL13RA1	[52, 65]
Interleukin-13 receptor subunit alpha-2*	I13R2_HUMAN	Q14627	IL13RA2	[65]
Interleukin-13*	IL13_HUMAN	P35225	IL13	[52]
Interleukin-15*	IL15_HUMAN	P40933	IL15	[52]
Interleukin-17 receptor B*	I17RB_HUMAN	Q9NRM6	IL17RB	[52, 65]
Interleukin-17 receptor C*	I17RC_HUMAN	Q8NAC3	IL17RC	[52]
Interleukin-17A*	IL17_HUMAN	Q16552	IL17A	[52, 65]
Interleukin-17C*	IL17C_HUMAN	Q9P0M4	IL17C	[65]
Interleukin-19*	IL19_HUMAN	Q9UHD0	IL19	[65]
Interleukin-20 receptor subunit alpha*	I20RA_HUMAN	Q9UHF4	IL20RA	[52]
Interleukin-21 receptor*	IL21R_HUMAN	Q9HBE5	IL21R	[65]
Interleukin-21*	IL21_HUMAN	Q9HBE4	IL21	[52, 65]
Interleukin-23 receptor*	IL23R_HUMAN	Q5VWK5	IL23R	[65]
Interleukin-23 subunit alpha*	IL23A_HUMAN	Q9NPF7	IL23A	[52, 65]
Interleukin-24*	IL24_HUMAN	Q13007	IL24	[65]
Interleukin-27 subunit alpha*	IL27A_HUMAN	Q8NEV9	IL27	[65]
Interleukin-36 gamma*	IL36G_HUMAN	Q9NZH8	IL36G	[65]
Interleukin-36 receptor antagonist protein*	I36RA_HUMAN	Q9UBH0	IL36RN	[65]
Interstitial collagenase*	MMP1_HUMAN	P03956	MMP1	[52, 61]
Islet amyloid polypeptide*	IAPP_HUMAN	P10997	IAPP	[52, 65]
Junctional adhesion molecule C	JAM3_HUMAN	Q9BX67	JAM3	[57]
Junctional adhesion molecule-like*	JAML_HUMAN	Q86YT9	JAML	[65]
Kallikrein 2	KLK2_HUMAN	P20151	KLK2	[52]
Kallikrein 11	KLK11_HUMAN	Q9UBX7	KLK11	[65]
Keratin, type I cytoskeletal 19*	K1C19_HUMAN	P08727	KRT19	[52, 65]
Kremen protein 1*	KREM1_HUMAN	Q96MU8	KREMEN1	[52]
Kremen protein 2*	KREM2_HUMAN	Q8NCW0	KREMEN2	[60, 65]
Lactadherin*	MFGM_HUMAN	Q08431	MFGE8	[60]
Lactotransferrin*	TRFL_HUMAN	P02788	LTF	[52, 59]
Lactoylglutathione lyase*	LGUL_HUMAN	Q04760	GLO1	[65]
Laminin subunit alpha-1	LAMA1_HUMAN	P25391	LAMA1	[57]
Laminin subunit alpha-2	LAMA2_HUMAN	P24043	LAMA2	[57]
Laminin subunit alpha-4	LAMA4_HUMAN	Q16363	LAMA4	[57]
Laminin subunit alpha-5	LAMA5_HUMAN	O15230	LAMA5	[57]
Laminin subunit beta-1	LAMB1_HUMAN	P07942	LAMB1	[57]
Laminin subunit beta-2	LAMB2_HUMAN	P55268	LAMB2	[57]
Laminin subunit gamma-1	LAMC1_HUMAN	P11047	LAMC1	[57]
Latent-transforming growth factor beta-binding protein 1	LTBP1_HUMAN	Q14766	LTBP1	[57]
Layilin	LAYN_HUMAN	Q6UX15	LAYN	[65]
Leucine-rich alpha-2-glycoprotein*	A2GL_HUMAN	P02750	LRG1	[52, 65]
Leukocyte surface antigen CD47	CD47_HUMAN	Q08722	CD47	[57]
Lipopolysaccharide-binding protein*	LBP_HUMAN	P18428	LBP	[65]
L-lactate dehydrogenase A chain*	LDHA_HUMAN	P00338	LDHA	[12]
Low affinity immunoglobulin epsilon Fc receptor*	FCER2_HUMAN	P06734	FCER2	[65]
Low-density lipoprotein receptor*	LDLR_HUMAN	P01130	LDLR	[65]
Low-density lipoprotein receptor-related protein 6*	LRP6_HUMAN	O75581	LRP6	[60]
L-Selectin	LYAM1_HUMAN	P14151	SELL	[52]
Lutropin-choriogonadotropic hormone receptor*	LSHR_HUMAN	P22888	LHCGR	[52]
Lymphocyte activation gene 3 protein*	LAG3_HUMAN	P18627	LAG3	[52]
Lymphotoxin-alpha*	TNFB_HUMAN	P01374	LTA	[52]
Lymphotoxin-beta	TNFC_HUMAN	Q06643	LTB	[65]
Lysosome membrane protein 2*	SCRB2_HUMAN	Q14108	SCARB2	[65]
Lysosome-associated membrane glycoprotein 2*	LAMP2_HUMAN	P13473	LAMP2	[12]
Macrophage migration inhibitory factor*	MIF_HUMAN	P14174	MIF	[65]
Mammaglobin A	SG2A2_HUMAN	Q13296	SCGB2A2	[52]
Mast/stem cell growth factor receptor Kit	KIT_HUMAN	P10721	KIT	[57]
Matrilysin*	MMP7_HUMAN	P09237	MMP7	[65]
Matrix metalloproteinase-9*	MMP9_HUMAN	P14780	MMP9	[61, 65]
Matrix metalloproteinase-14*	MMP14_HUMAN	P50281	MMP14	[65]
Matrix metalloproteinase-19*	MMP19_HUMAN	Q99542	MMP19	[52]
Matrix metalloproteinase-20*	MMP20_HUMAN	O60882	MMP20	[52, 60, 65]
Matrix metalloproteinase-24*	MMP24_HUMAN	Q9Y5R2	MMP24	[52, 65]
Megakaryocyte-associated tyrosine-protein kinase*	MATK_HUMAN	P42679	MATK	[52]
Metalloproteinase inhibitor 2	TIMP2_HUMAN	P16035	TIMP2	[60]
Metalloproteinase inhibitor 3*	TIMP3_HUMAN	P35625	TIMP3	[65]
MHC class I polypeptide-related sequence A*	MICA_HUMAN	Q29983	MICA	[65]
Microglobulin***	–	–	–	[65]
Microtubule-associated tumor suppressor 1*	MTUS1_HUMAN	Q9ULD2	MTUS1	[65]
Mitogen-activated protein kinase 1	MK01_HUMAN	P28482	MAPK1	[57]
Mitogen-activated protein kinase 3	MK03_HUMAN	P27361	MAPK3	[57]
Monocyte differentiation antigen CD14*	CD14_HUMAN	P08571	CD14	[65]
MSHa***	–	–	MSX1	[52]
Mucin-1*	MUC1_HUMAN	P15941	MUC1	[65]
Mucin-16*	MUC16_HUMAN	Q8WXI7	MUC16	[52, 65]
Mucosal addressin cell adhesion molecule 1	MADCA_HUMAN	Q13477	MADCAM1	[57]
Muscle, skeletal receptor tyrosine-protein kinase*	MUSK_HUMAN	O15146	MUSK	[52]
Myeloid-derived growth factor	MYDGF_HUMAN	Q969H8	MYDGF	[57]
Natriuretic peptides B*	ANFB_HUMAN	P16860	NPPB	[52]
Natural killer cell receptor 2B4*	CD244_HUMAN	Q9BZW8	CD244	[65]
Neprilysin	NEP_HUMAN	P08473	MME	[63]
Netrin-1*	NET1_HUMAN	O95631	NTN1	[52]
Netrin-G2	NTNG2_HUMAN	Q96CW9	NTNG2	[52]
Neural cell adhesion molecule 1*	NCAM1_HUMAN	P13591	NCAM1	[65]
Neural cell adhesion molecule L1-like protein	NCHL1_HUMAN	O00533	CHL1	[57]
Neuregulin-1 (cleaved form pro-neuregulin-1, membrane-bound isoform)	NRG1_HUMAN	Q02297	NRG1	[52]
Neuregulin-2 (cleaved pro-neuregulin-2, membrane-bound isoform)*	NRG2_HUMAN	O14511	NRG2	[52]
Neuregulin-3 (cleaved pro-neuregulin-3, membrane-bound isoform)*	NRG3_HUMAN	P56975	NRG3	[52]
Neurofibromin*	NF1_HUMAN	P21359	NF1	[52]
Neurogenic differentiation factor 1*	NDF1_HUMAN	Q13562	NEUROD1	[65]
Neuronal pentraxin-1	NPTX1_HUMAN	Q15818	NPTX1	[52]
Neuropeptide Y (cleaved form pro-neuropeptide Y)	NPY_HUMAN	P01303	NPY	[65]
Neurosecretory protein VGF*	VGF_HUMAN	O15240	VGF	[52]
Neuroserpin*	NEUS_HUMAN	Q99574	SERPINI1	[65]
Neurturin	NRTN_HUMAN	Q99748	NRTN	[65]
Neutrophil collagenase*	MMP8_HUMAN	P22894	MMP8	[52]
Neutrophil-activating peptide 2 (cleaved from Platelet basic protein)*	CXCL7_HUMAN	P02775	PPBP	[65]
Non-receptor tyrosine-protein kinase TYK2*	TYK2_HUMAN	P29597	TYK2	[65]
Nucleoside diphosphate kinase A	NDKA_HUMAN	P15531	NME1	[65]
Orexin receptor type 1*	OX1R_HUMAN	O43613	HCRTR1	[65]
OX-2 membrane glycoprotein*	OX2G_HUMAN	P41217	CD200	[65]
Pentraxin-related protein PTX3	PTX3_HUMAN	P26022	PTX3	[59, 60]
Peptide YY	PYY_HUMAN	P10082	PYY	[65]
Periostin	POSTN_HUMAN	Q15063	POSTN	[59]
Phosphatidylinositol 3-kinase regulatory subunit beta*	P85B_HUMAN	O00459	PIK3R2	[52]
Phosphoglycerate Kinase 1	PGK1_HUMAN	P00558	PGK1	[12]
Plakophilin-1	PKP1_HUMAN	Q13835	PKP1	[59]
Plasma protease C1 inhibitor*	IC1_HUMAN	P05155	SERPING1	[52]
Platelet endothelial cell adhesion molecule*	PECA1_HUMAN	P16284	PECAM1	[61]
Platelet glycoprotein 4*	CD36_HUMAN	P16671	CD36	[65]
Platelet-derived growth factor D*	PDGFD_HUMAN	Q9GZP0	PDGFD	[65]
Platelet-derived growth factor receptor alpha*	PGFRA_HUMAN	P16234	PDGFRA	[52, 57, 65]
Platelet-derived growth factor receptor beta*	PGFRB_HUMAN	P09619	PDGFRB	[52, 57]
Platelet-derived growth factor subunit B	PDGFB_HUMAN	P01127	PDGFB	[57]
Polyubiquitin-B*	UBB_HUMAN	P0CG47	UBB	[52, 65]
PPARg2***	–	–	PPARG	[52]
Probetacellulin*	BTC_HUMAN	P35070	BTC	[52, 65]
Pro-epidermal growth factor*	EGF_HUMAN	P01133	EGF	[61]
Progesterone receptor	PRGR_HUMAN	P06401	PGR	[52]
pro-Glucagon	GLUC_HUMAN	P01275	GCG	[65]
Progranulin	GRN_HUMAN	P28799	GRN	[65]
Proheparin-binding EGF-like growth factor*	HBEGF_HUMAN	Q99075	HBEGF	[65]
Prokineticin-1*	PROK1_HUMAN	P58294	PROK1	[65]
ProSAAS	PCS1N_HUMAN	Q9UHG2	PCSK1N	[52]
Prostaglandin D2 receptor 2*	PD2R2_HUMAN	Q9Y5Y4	PTGDR2	[65]
Protein AMBP*	AMBP_HUMAN	P02760	AMBP	[65]
Protein FAM3B	FAM3B_HUMAN	P58499	FAM3B	[52, 65]
Protein S100-A6	S10A6_HUMAN	P06703	S100A6	[65]
Protein S100-A8	S10A8_HUMAN	P05109	S100A8	[52, 65]
Protein S100-A10	S10AA_HUMAN	P60903	S100A10	[65]
Protein S100-A12*	S10AC_HUMAN	P80511	S100A12	[65]
Protein Wnt-5a	WNT5A_HUMAN	P41221	WNT5A	[57]
Protein Wnt-5b	WNT5B_HUMAN	Q9H1J7	WNT5B	[57]
Protein wntless homolog	WLS_HUMAN	Q5T9L3	WLS	[57]
Protocadherin Fat 1	FAT1_HUMAN	Q14517	FAT1	[57]
Protocadherin Fat 4	FAT4_HUMAN	Q6V0I7	FAT4	[57]
Protocadherin gamma-C3	PCDGK_HUMAN	Q9UN70	PCDHGC3	[57]
Protocadherin-7	PCDH7_HUMAN	O60245	PCDH7	[57]
Protocadherin-9	PCDH9_HUMAN	Q9HC56	PCDH9	[57]
Protocadherin-18	PCD18_HUMAN	Q9HCL0	PCDH18	[57]
Proto-oncogene tyrosine-protein kinase receptor Ret*	RET_HUMAN	P07949	RET	[65]
P-selectin	LYAM3_HUMAN	P16109	SELP	[52]
Ras-related protein R-Ras	RRAS_HUMAN	P10301	RRAS	[57]
Ras-related protein R-Ras2	RRAS2_HUMAN	P62070	RRAS2	[57]
Receptor tyrosine-protein kinase erbB-2*	ERBB2_HUMAN	P04626	ERBB2	[65]
Receptor tyrosine-protein kinase erbB-4*	ERBB4_HUMAN	Q15303	ERBB4	[65]
Receptor-interacting serine/ threonine-protein kinase 1*	RIPK1_HUMAN	Q13546	RIPK1	[65]
Receptor-type tyrosine-protein kinase FLT3*	FLT3_HUMAN	P36888	FLT3	[65]
Receptor-type tyrosine-protein phosphatase delta*	PTPRD_HUMAN	P23468	PTPRD	[52]
Rho family-interacting cell polarization regulator 1	RIPR1_HUMAN	Q6ZS17	RIPOR1	[57]
Rho GTPase-activating protein 1	RHG01_HUMAN	Q07960	ARHGAP1	[57]
Rho guanine nucleotide exchange factor 1	ARHG1_HUMAN	Q92888	ARHGEF1	[57]
Rho guanine nucleotide exchange factor 7	ARHG7_HUMAN	Q14155	ARHGEF7	[57]
Rho-associated protein kinase 1*	ROCK1_HUMAN	Q13464	ROCK1	[52, 57]
Rho-associated protein kinase 2	ROCK2_HUMAN	O75116	ROCK2	[57]
Rho-related GTP-binding protein RhoB	RHOB_HUMAN	P62745	RHOB	[57]
Rho-related GTP-binding protein RhoE	RND3_HUMAN	P61587	RND3	[57]
Rho-related GTP-binding protein RhoG	RHOG_HUMAN	P84095	RHOG	[57]
Ribosomal oxygenase 2*	RIOX2_HUMAN	Q8IUF8	RIOX2	[52]
Scavenger receptor cysteine-rich type 1 protein M130*	C163A_HUMAN	Q86VB7	CD163	[52]
Sclerostin*	SOST_HUMAN	Q9BQB4	SOST	[65]
Secreted frizzled-related protein 1*	SFRP1_HUMAN	Q8N474	SFRP1	[65]
Serum amyloid A-1 protein*	SAA1_HUMAN	P0DJI8	SAA1	[52]
Secreted frizzled-related protein 3*	SFRP3_HUMAN	Q92765	FRZB	[65]
Secreted frizzled-related protein 4*	SFRP4_HUMAN	Q6FHJ7	SFRP4	[60]
Serine/threonine-protein kinase MRCK alpha	MRCKA_HUMAN	Q5VT25	CDC42BPA	[57]
Serine/threonine-protein kinase MRCK beta	MRCKB_HUMAN	Q9Y5S2	CDC42BPB	[57]
Serotransferrin	TRFE_HUMAN	P02787	TF	[59]
Sex hormone-binding globulin*	SHBG_HUMAN	P04278	SHBG	[52]
Sialic acid-binding Ig-like lectin 5*	SIGL5_HUMAN	O15389	SIGLEC5	[65]
Sialic acid-binding Ig-like lectin 9*	SIGL9_HUMAN	Q9Y336	SIGLEC9	[52]
Signal peptide, CUB and EGF-like domain-containing protein 3	SCUB3_HUMAN	Q8IX30	SCUBE3	[57]
Signal transducer CD24*	CD24_HUMAN	P25063	CD24	[65]
SLIT-ROBO Rho GTPase-activating protein 1	SRGP1_HUMAN	Q7Z6B7	SRGAP1	[57]
SLIT-ROBO Rho GTPase-activating protein 2	SRGP2_HUMAN	O75044	SRGAP2	[57]
Solute carrier family 2, facilitated glucose transporter member 1*	GTR1_HUMAN	P11166	SLC2A1	[52, 65]
Solute carrier family 2, facilitated glucose transporter member 2*	GTR2_HUMAN	P11168	SLC2A2	[52]
Solute carrier family 2, facilitated glucose transporter member 3*	GTR3_HUMAN	P11169	SLC2A3	[65]
Solute carrier family 2, facilitated glucose transporter member 5*	GTR5_HUMAN	P22732	SLC2A5	[52, 65]
Somatotropin*	SOMA_HUMAN	P01241	GH1	[52]
Sonic hedgehog protein*	SHH_HUMAN	Q15465	SHH	[52]
SPARC	SPRC_HUMAN	P09486	SPARC	[60]
Sphingosine 1-phosphate receptor 1*	S1PR1_HUMAN	P21453	S1PR1	[52, 65]
Stromal cell-derived factor 1*	SDF1_HUMAN	P48061	CXCL12	[52]
Stromelysin-2*	MMP10_HUMAN	P09238	MMP10	[65]
Stromelysin-3	MMP11_HUMAN	P24347	MMP11	[52, 65]
SWI/SNF-related matrix-associated actin-dependent regulator of chromatin subfamily E member 1*	SMCE1_HUMAN	Q969G3	SMARCE1	[65]
TGF-beta 5***	–	–	TGFB5	[52]
TGF-beta receptor type-2	TGFR2_HUMAN	P37173	TGFBR2	[57]
Thioredoxin-interacting protein*	TXNIP_HUMAN	Q9H3M7	TXNIP	[52, 65]
Thrombopoietin	TPO_HUMAN	P40225	THPO	[65]
Thrombospondin-1	TSP1_HUMAN	P07996	THBS1	[59, 60]
Thrombospondin-2	TSP2_HUMAN	P35442	THBS2	[52]
Thyroid peroxidase*	PERT_HUMAN	P07202	TPO	[52]
Thyrotropin subunit beta*	TSHB_HUMAN	P01222	TSHB	[52]
T lymphocyte activation antigen CD80*	CD80_HUMAN	P33681	CD80	[60, 65]
Toll-like receptor 2*	TLR2_HUMAN	O60603	TLR2	[65]
Toll-like receptor 4*	TLR4_HUMAN	O00206	TLR4	[65]
TRA-1-60 and TRA-1-81***	–	–	PODXL	[52]
Transcription factor SOX-2*	SOX2_HUMAN	P48431	SOX2	[65]
Transcription initiation factor TFIID subunit 4*	TAF4_HUMAN	O00268	TAF4	[65]
Transferrin receptor protein 1*	TFR1_HUMAN	P02786	TFRC	[52, 65]
Transforming growth factor alpha* (cleaved from Protransforming growth factor alpha)	TGFA_HUMAN	P01135	TGFA	[61]
Transforming growth factor beta receptor type 3*	TGBR3_HUMAN	Q03167	TGFBR3	[65]
Transforming growth factor beta-1 (cleaved from Transforming growth factor beta-1 proprotein)*	TGFB1_HUMAN	P01137	TGFB1	[52, 57, 65]
Transforming growth factor beta-3* (cleaved form Transforming growth factor beta-3 proprotein)	TGFB3_HUMAN	P10600	TGFB3	[61]
Transforming growth factor-beta-induced protein ig-h3	BGH3_HUMAN	Q15582	TGFBI	[57, 59]
Transforming protein RhoA	RHOA_HUMAN	P61586	RHOA	[57]
Transient receptor potential cation channel subfamily M member 7*	TRPM7_HUMAN	Q96QT4	TRPM7	[65]
Triggering receptor expressed on myeloid cells 1*	TREM1_HUMAN	Q9NP99	TREM1	[65]
Troponin C, slow skeletal and cardiac muscles*	TNNC1_HUMAN	P63316	TNNC1	[52]
Tumor necrosis factor ligand superfamily member 10*	TNF10_HUMAN	P50591	TNFSF10	[65]
Tumor necrosis factor ligand superfamily member 11*	TNF11_HUMAN	O14788	TNFSF11	[65]
Tumor necrosis factor ligand superfamily member 13*	TNF13_HUMAN	O75888	TNFSF13	[60]
Tumor necrosis factor ligand superfamily member 15*	TNF15_HUMAN	O95150	TNFSF15	[65]
Tumor necrosis factor ligand superfamily member 4*	TNFL4_HUMAN	P23510	TNFSF4	[65]
Tumor necrosis factor ligand superfamily member 6*	TNFL6_HUMAN	P48023	FASLG	[65]
Tumor necrosis factor ligand superfamily member 8*	TNFL8_HUMAN	P32971	TNFRSF8	[52, 65]
Tumor necrosis factor receptor superfamily member 10A*	TR10A_HUMAN	O00220	TNFRSF10A	[52]
Tumor necrosis factor receptor superfamily member 10B*	TR10B_HUMAN	O14763	TNFRSF10B	[52]
Tumor necrosis factor receptor superfamily member 11B*	TR11B_HUMAN	O00300	TNFRSF11B	[60]
Tumor necrosis factor receptor superfamily member 13B*	TR13B_HUMAN	O14836	TNFRSF13B	[52]
Tumor necrosis factor receptor superfamily member 13C*	TR13C_HUMAN	Q96RJ3	TNFRSF13C	[52, 60, 65]
Tumor necrosis factor receptor superfamily member 6B*	TNF6B_HUMAN	O95407	TNFRSF6B	[65]
Tumor necrosis factor receptor superfamily member 14*	TNR14_HUMAN	Q92956	TNFRSF14	[65]
Tumor necrosis factor receptor superfamily member 17*	TNR17_HUMAN	Q02223	TNFRSF17	[65]
Tumor necrosis factor receptor superfamily member 19*	TNR19_HUMAN	Q9NS68	TNFRSF19	[65]
Tumor necrosis factor receptor superfamily member 25*	TNR25_HUMAN	Q93038	TNFRSF25	[52, 65]
Tumor necrosis factor receptor superfamily member 27*	TNR27_HUMAN	Q9HAV5	EDA2R	[52, 65]
Tumor necrosis factor receptor type 1-associated DEATH domain protein*	TRADD_HUMAN	Q15628	TRADD	[52]
Tumor necrosis factor*	TNFA_HUMAN	P01375	TNF	[52]
Tyrosine-protein kinase ABL1*	ABL1_HUMAN	P00519	ABL1	[52, 65]
Tyrosine-protein kinase BTK*	BTK_HUMAN	Q06187	BTK	[52, 65]
Tyrosine-protein kinase Fer*	FER_HUMAN	P16591	FER	[52, 65]
Tyrosine-protein kinase FRK*	FRK_HUMAN	P42685	FRK	[52]
Tyrosine-protein kinase Fyn*	FYN_HUMAN	P06241	FYN	[52, 65]
Tyrosine-protein kinase HCK*	HCK_HUMAN	P08631	HCK	[52]
Tyrosine-protein kinase ITK/TSK*	ITK_HUMAN	Q08881	ITK	[52]
Tyrosine-protein kinase Lck*	LCK_HUMAN	P06239	LCK	[52]
Tyrosine-protein kinase Lyn*	LYN_HUMAN	P07948	LYN	[52]
Tyrosine-protein kinase receptor Tie-1*	TIE1_HUMAN	P35590	TIE1	[61]
Tyrosine-protein kinase receptor UFO*	UFO_HUMAN	P30530	AXL	[60, 65]
Tyrosine-protein kinase Tec*	TEC_HUMAN	P42680	TEC	[52]
Tyrosine-protein kinase TXK*	TXK_HUMAN	P42681	TXK	[52]
Tyrosine-protein kinase ZAP-70*	ZAP70_HUMAN	P43403	ZAP70	[52]
Urokinase plasminogen activator surface receptor*	UPAR_HUMAN	Q03405	PLAUR	[57, 61]
Vascular endothelial growth factor A*	VEGFA_HUMAN	P15692	VEGFA	[61, 65]
Vascular endothelial growth factor C*	VEGFC_HUMAN	P49767	VEGFC	[65]
Vascular endothelial growth factor D*	VEGFD_HUMAN	O43915	VEGFD	[61, 65]
Vascular endothelial growth factor receptor 1*	VGFR1_HUMAN	P17948	FLT1	[52]
Vascular endothelial growth factor receptor 2*	VGFR2_HUMAN	P35968	KDR	[61, 65]
Vascular endothelial growth factor receptor 3*	VGFR3_HUMAN	P35916	FLT4	[61]
Vinculin	VINC_HUMAN	P18206	VCL	[57]
Vitamin D-binding protein	VTDB_HUMAN	P02774	GC	[59]
Vitronectin	VTNC_HUMAN	P04004	VTN	[57]
WAP, Kazal, immunoglobulin, Kunitz and NTR domain-containing protein 1*	WFKN1_HUMAN	Q96NZ8	WFIKKN1	[52]
X-linked interleukin-1 receptor accessory protein-like 2*	IRPL2_HUMAN	Q9NP60	IL1RAPL2	[65]

*The referred article used alternative or short names

**The specific member of this family detected has been not described

***Name and gene referred by the article cited

The detailed molecular functions enabled by each protein are collected in Table 1S. The results showed that 577 proteins contribute to different molecular functions described by 710 GO terms. For the BMP-binding endothelial regulator protein, carcinoembryonic antigen-related cell adhesion molecule, coagulation factor XIII B chain and kremen protein 2, no GO annotations were found.

**Fig. 1:**
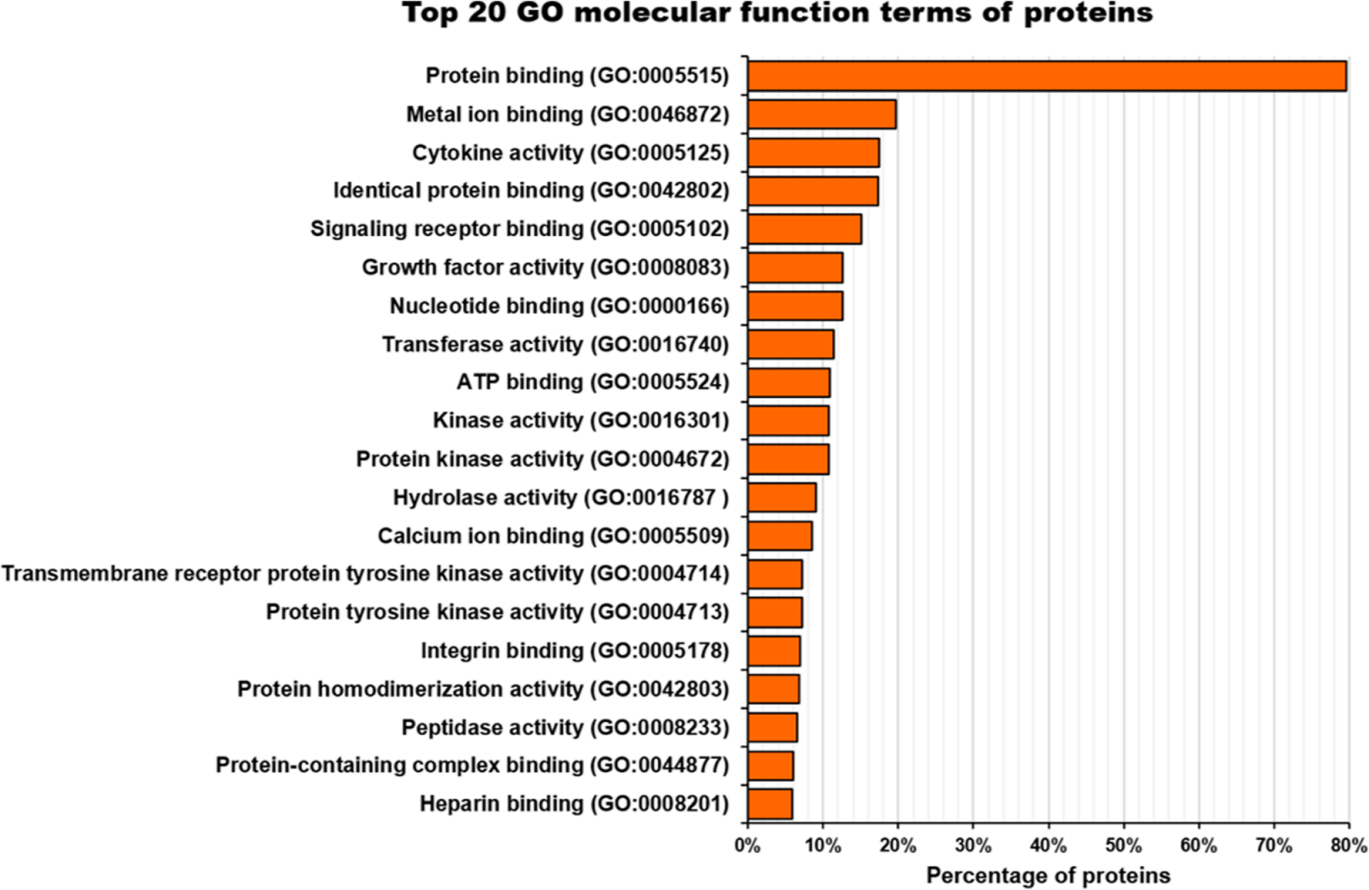
The top 20 gene ontology (GO) molecular function terms of the proteins detected in human AT-MSC-EVs. The 80% of the proteins associated with these EVs enables the protein binding

**Fig. 2:**
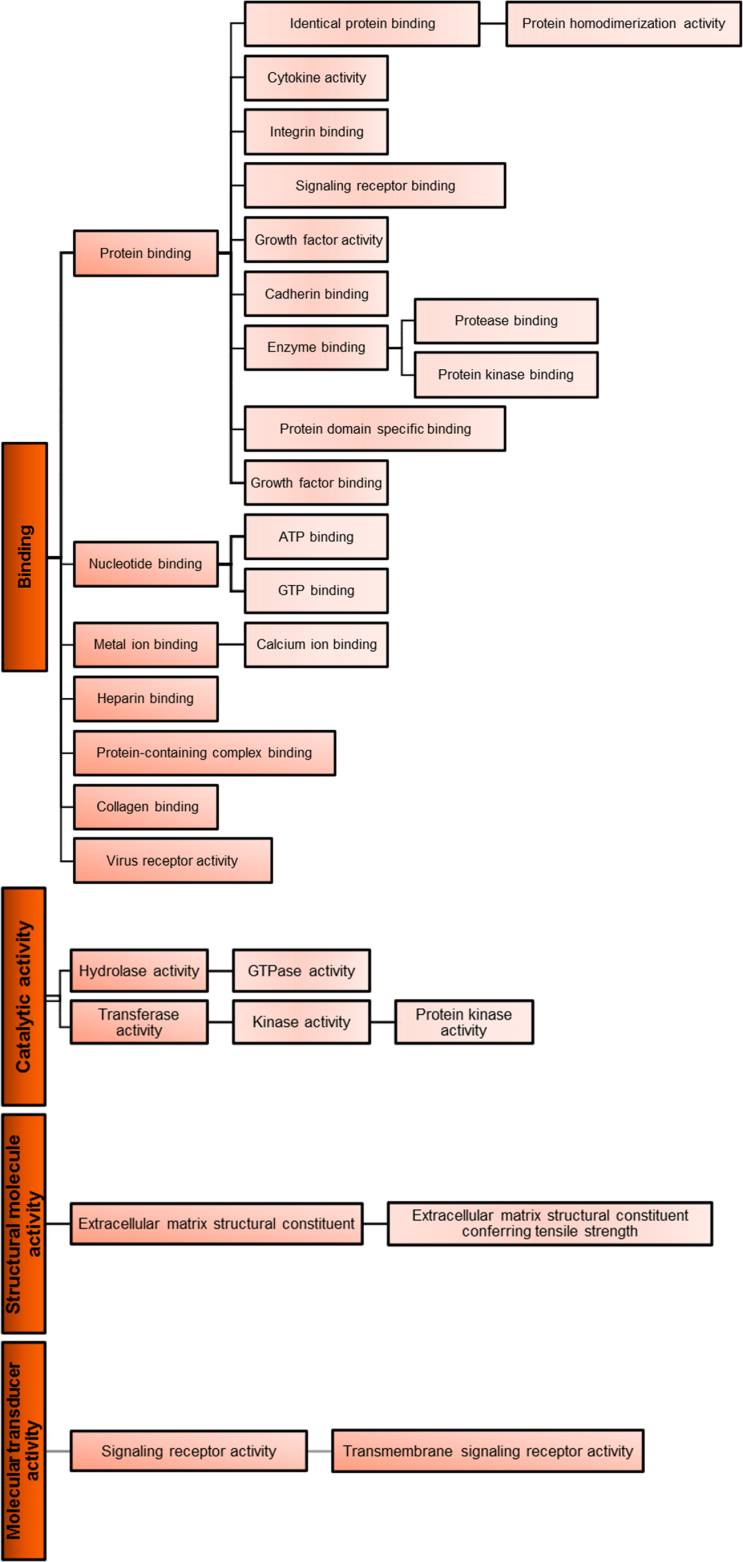
Simplified outline of the main molecular functions enabled by proteins detected in EVs derived from human AT-MSC. For a complete review of the relationships between gene ontology terms see the chart view in the web-based tool QuickGO (https://www.ebi.ac.uk/QuickGO/)

The main molecular functions enabled by the AT-MSC-EVs proteins are described by specific child terms (more specific terms) of binding: protein binding (80%), metal ion binding (20%), cytokine activity (18%), identical protein binding (17%), and signaling receptor binding (15%) (Fig. 1). Therefore, binding seems to be the most relevant molecular function of AT-MSC-EVs. The number of AT-MSC-EVs proteins involved in each molecular function is variable. Most described molecular functions are enabled by a limited number of proteins (less than 10), and only 11.6% of the functions are enabled by 10 or more proteins.They are related by specific terms of four molecular functions: binding, catalytic activity, structural molecule activity and molecular transducer activity (Fig. 2).

**Fig. 3:**
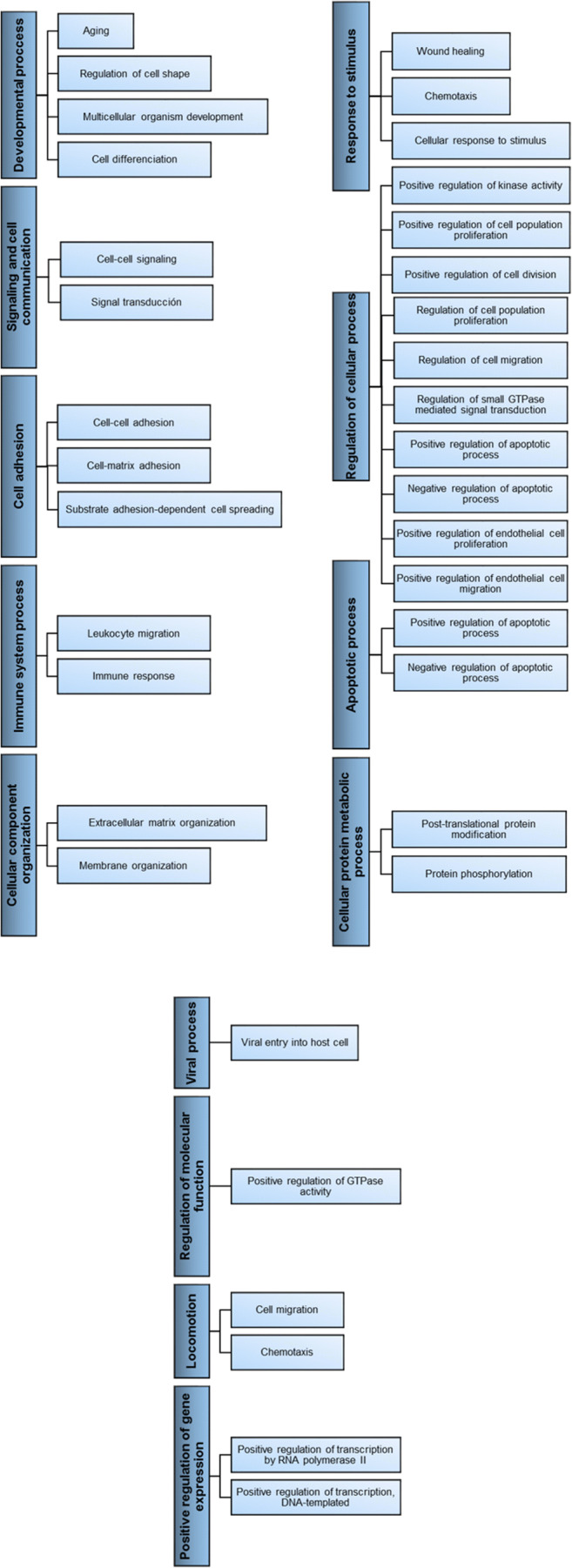
Simplified outline of the main biological processes in which proteins detected in EVs derived from human AT-MSC are involved. For a complete review of the relationships between gene ontology terms see the chart view in the web-based tool QuickGO (https://www.ebi.ac.uk/QuickGO/)

578 of the AT-MSC-EVs proteins identified play a role in different biological processes described by 3884 GO terms. For carcinoembryonic antigen-related cell adhesion molecule 7, layilin, and sex hormone-binding globulin, no GO annotations were found. The proteins involved in each process are reported in Table 2S. The biological processes in which a relatively large number of proteins are involved are: developmental process, signaling and cell communication, cell adhesion, immune system process, cellular component organization, response to stimulus, regulation of cellular process, apoptotic process, cellular protein metabolic process, viral process, regulation of molecular function, locomotion, and positive regulation of gene expression (Fig. 3).

**Fig. 4:**
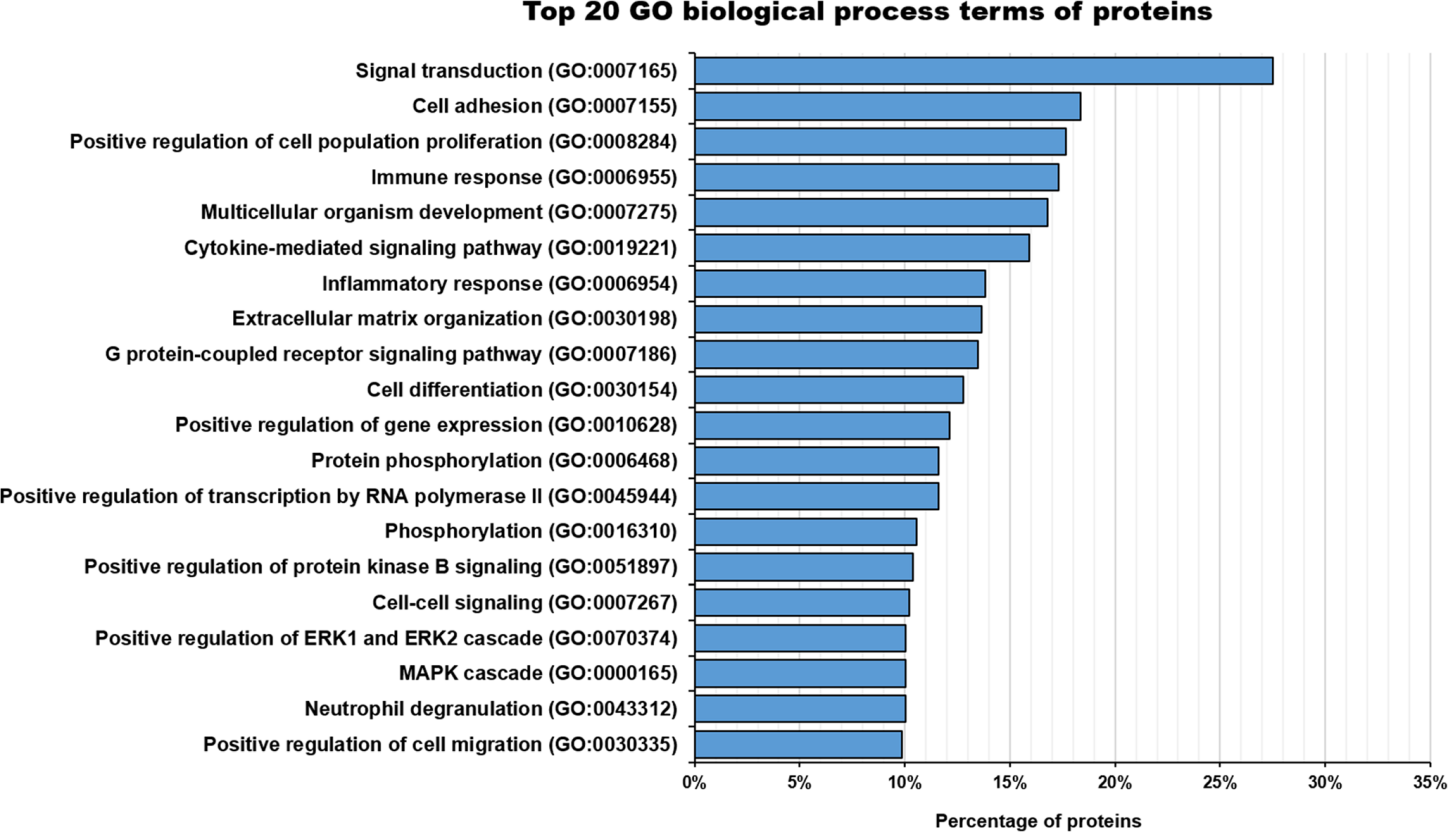
The top 20 gene ontology (GO) biological process terms of the proteins detected in human AT-MSC-EVs. The 28% of the proteins are involved in signal transduction

The proteins detected in AT-MSC-EV cargo are involved in a great number of biological processes, but only a few of these processes utilise a large number of proteins. The biological processes in which the largest number of proteins take part are cell adhesion (in which 18% of proteins are involved) and specific child terms of signaling and cell communication (28% signal transduction), regulation of cellular processes (18% positive regulation of cell population proliferation), immune system process (17% immune response) and developmental processes (17% multicellular organism development) (Fig. 4).

### Therapeutic Approaches of AT-MSC-EV Proteins

These results illustrate the role of AT-MSC-EVs in cell-cell communication [3–6], and the promising therapeutic effects observed in different research fields. Regarding the musculoskeletal system, AT-MSC-EVs have shown protective effects against cartilage degeneration, promotion of cell proliferation and migration of osteoarthritis chondrocytes, and antisenescence effects in osteoarthritis osteoblasts in vitro and in vivo [66, 78]. They have also shown protective properties on muscle damage in an in vivo model of hindlimb ischemia and in an in vitro model of ischemia/reperfusion [52]. These effects may be a consequence of the presence of proteins such as lactotransferrin, C-X-C motif chemokine 16, protein Wnt-5a, and transforming protein RhoA, which are involved in positive regulation of chondrocyte proliferation, positive regulation of cell migration, regulation of inflammatory response and regulation of osteoblast proliferation, respectively. The complete list of proteins involved in these processes is reported in Table 2S.

With regard to cardiology and vascular system, AT-MSC-EVs are involved in a wide range of biological processes, including heart development, contraction and morphogenesis, positive regulation of cardiac muscle cell proliferation and hypertrophy, regulation of cardiac muscle cell apoptotic process and proliferation, blood vessel maturation, remodeling and morphogenesis, regulation of blood vessel diameter and angiogenesis, among others (Table 2S). Hence, numerous proteins detected in AT-MSC-EVs could account for the protective effects observed in cardiac function and cardiomyocytes after their injection in an in vivo model of myocardial infarction [79] . In addition, the effects of AT-MSC-EVs in angiogenesis have been also studied in vitro and in vivo [60, 72, 80]. Proteins detected in AT-MSC-EVs such as IL-1 alpha and apelin receptor are proangiogenic, while SPARC is antiangiogenic (Table 2S).

Human AT-MSC-EVs also have an inhibitory effect on vein graft neointima formation, as observed in a mouse model of vein grafting [81]. This effect correlated with decreased macrophage infiltration, attenuated inflammatory cytokine expression, and reduced activation of MAPK and phosphatidylinositol-3 kinase signaling pathways [81]. EV proteins potentially involved in these processes are thrombospondin-1 (inflammatory response), IL-4 (negative regulation of macrophage activation), growth factor receptor-bound protein 2 (regulation of MAPK cascade) and MAP kinase 1 (regulation of phosphatidylinositol 3-kinase signaling) (Table 2S).

The effects of AT-MSC-EVs proteins in the vascular system may also be related to the cardio-renal protection observed in a deoxycorticosterone acetate-salt hypertensive animal model [82]. Thus, the administration of AT-MSC-EVs in this in vivo model protected against renal damage, preserved renal function, reduced inflammatory response, prevented fibrosis in the kidney and in cardiac tissue, and conserved normal blood pressure [82]. The administration of AT-MSC-EVs also showed a renal protective effect in an in vivo model of acute kidney injury [83]. Proteins detected in AT-MSC-EVs such as integrin alpha-3, IL-4, IL-10, collagen alpha-2(I) chain or periostin could be implicated in these outcomes (Table 2S).

Finally, the action of AT-MSC-EVs in skin diseases has also been studied [62, 68, 84, 85]. Human AT-MSC-EVs enhanced cutaneous repair and regeneration, both in vitro and in vivo, by the promotion of cell migration and proliferation, the inhibition of cell apoptosis and the regulation of fibroblast differentiation during skin wound healing [68, 84, 85]. This is unsurprising, considering that the main biological processes of proteins described previously include response to stimulus (wound healing) and regulation of cellular processes (cell proliferation and migration) and apoptotic processes (Fig. 3, Table 2S). Proteins involved in these biological processes, along with those previously described in the vascular system, could support the protective effect of skin flaps against ischemia/reperfusion injury [62]. Although several proteins may be involved, in this study the observed effect was ascribed to the promotion of angiogenesis via IL-6, along with other mechanisms [62].

### miRNA

AT-MSC-EVs cargo also contains several types of RNA, mainly miRNA, tRNA, mRNA, rRNA, snRNA, snoRNA and scRNA [53, 54]. AT-MSC-EVs are rich in miRNA [12, 54, 69, 70], which represents approximately 44% of all small, non-coding RNA detected in AT-MSC [53]. Currently, 604 miRNAs have been identified in AT-MSC-EVs (Table 2). The methods used for RNA analysis were sequencing systems [11, 53, 54, 59, 66, 67, 71, 74], quantitative real-time PCR [64, 65, 68, 72, 73], OpenArray systems [69, 70] and GeneChip RNA array [12], among others. The isolation methods of EVs used in those studies were centrifugation and/or ultracentrifugation [12, 64, 65, 67–69, 72, 74], commercial EV isolation kits [11, 53, 54, 59, 71, 73] and multi-filtration [66].

**Table 2:** miRNAs detected in human AT-MSC-EVs in alphabetical order

Family	Name	RNAcental	Sequence	Ref.
let-7 [74]	hsa-let-7a-3p	URS000004F5D8_9606	CUAUACAAUCUACUGUCUUUC	[53]
	hsa-let-7a-5p (hsa-let-7a) [65]	URS0000416056_9606	UGAGGUAGUAGGUUGUAUAGUU	[11, 12, 53, 54, 65, 66, 69]
	hsa-let-7b-3p (hsa-let-7b*) [65]	URS00005918D5_9606	CUAUACAACCUACUGCCUUCCC	[53, 65]
	hsa-let-7b-5p (hsa-let-7b) [65]	URS0000324096_9606	UGAGGUAGUAGGUUGUGUGGUU	[12, 53, 54, 65, 66]
	hsa-let-7c-5p	URS000050DE77_9606	UGAGGUAGUAGGUUGUAUGGUU	[12, 53, 54]
	hsa-let-7d-5p (hsa-let-7d) [65]	URS00000A07C1_9606	AGAGGUAGUAGGUUGCAUAGUU	[54, 65]
	hsa-let-7e-5p (hsa-let-7e) [65]	URS000000B1C9_9606	UGAGGUAGGAGGUUGUAUAGUU	[12, 53, 54, 65, 66]
	hsa-let-7f-5p (hsa-let-7f) [65, 67]	URS00003B7674_9606	UGAGGUAGUAGAUUGUAUAGUU	[11, 53, 54, 65–67, 72]
	hsa-let-7 g-5p (hsa-let-7 g) [65]	URS00004AFF8D_9606	UGAGGUAGUAGUUUGUACAGUU	[54, 65]
	hsa-let-7i-3p (hsa-let-7i*)	URS0000237CBD_9606	CUGCGCAAGCUACUGCCUUGCU	[65]
	hsa-let-7i-5p	URS00004023EA_9606	UGAGGUAGUAGUUUGUGCUGUU	[53, 54, 72]
	hsa-miR-98-5p (hsa-miR-98) [65]	URS00004E0808_9606	UGAGGUAGUAAGUUGUAUUGUU	[53, 65]
mir-1	hsa-miR-206	URS000034B6F5_9606	UGGAAUGUAAGGAAGUGUGUGG	[65]
mir-10	hsa-miR-100-3p (hsa-miR-100*)	URS00001A405B_9606	CAAGCUUGUAUCUAUAGGUAUG	[65]
	hsa-miR-100-5p (hsa-miR-100) [65, 74]	URS000040D674_9606	AACCCGUAGAUCCGAACUUGUG	[11, 12, 53, 54, 65, 74]
	hsa-miR-10a-3p (hsa-miR-10a*)	URS00002F4762_9606	CAAAUUCGUAUCUAGGGGAAUA	[65]
	hsa-miR-10a-5p (hsa-miR-10a) [65, 67]	URS000016D2D4_9606	UACCCUGUAGAUCCGAAUUUGUG	[11, 53, 54, 65, 67]
	hsa-miR-10b-3p (hsa-miR-10b*) [65]	URS00004AC389_9606	ACAGAUUCGAUUCUAGGGGAAU	[53, 65, 70]
	hsa-miR-10b-5p (hsa-miR-10b) [65, 67]	URS000058760A_9606	UACCCUGUAGAACCGAAUUUGUG	[11, 53, 54, 65, 67]
	hsa-miR-125a-3p	URS00001F0C23_9606	ACAGGUGAGGUUCUUGGGAGCC	[65]
	hsa-miR-125a-5p	URS00005A4DCF_9606	UCCCUGAGACCCUUUAACCUGUGA	[53, 54, 65]
	hsa-miR-125b-1-3 (hsa-miR-125b-1*)[65]p	URS00002DABEA_9606	ACGGGUUAGGCUCUUGGGAGCU	[53, 54, 65]
	hsa-miR-125b-2-3p (hsa-miR-125b-2*)	URS00001925C1_9606	UCACAAGUCAGGCUCUUGGGAC	[65]
	hsa-miR-125b-5p (hsa-miR-125b) [65]	URS0000209905_9606	UCCCUGAGACCCUAACUUGUGA	[12, 53, 54, 65, 66, 72]
	hsa-miR-99a-3p	URS00005C62FC_9606	CAAGCUCGCUUCUAUGGGUCUG	[65]
	hsa-miR-99a-5p	URS0000157026_9606	AACCCGUAGAUCCGAUCUUGUG	[54, 65]
	hsa-miR-99b-3p (hsa-miR-99b*)	URS00001C308D_9606	CAAGCUCGUGUCUGUGGGUCCG	[65]
	hsa-miR-99b-5p (hsa-miR-99b) [65]	URS00002C10B3_9606	CACCCGUAGAACCGACCUUGCG	[11, 54, 65]
mir-101	hsa-miR-101–3p (hsa-miR-101) [65]	URS00001230A0_9606	UACAGUACUGUGAUAACUGAA	[54, 65, 69]
mir-103	hsa-miR-103a-3p (hsa-miR-103) [65]	URS0000476BE1_9606	AGCAGCAUUGUACAGGGCUAUGA	[12, 54, 65, 69]
	hsa-miR-107	URS00005743AE_9606	AGCAGCAUUGUACAGGGCUAUCA	[54, 65]
mir-1179	hsa-miR-1179	URS000048B5E9_9606	AAGCAUUCUUUCAUUGGUUGG	[65]
mir-1183	hsa-miR-1183	URS000075A336_9606	CACUGUAGGUGAUGGUGAGAGUGGGCA	[65]
mir-1204	hsa-miR-1204	URS000075E520_9606	UCGUGGCCUGGUCUCCAUUAU	[65]
mir-1207	hsa-miR-1207-5p	URS000055C019_9606	UGGCAGGGAGGCUGGGAGGGG	[72]
mir-1208	hsa-miR-1208	URS000075B904_9606	UCACUGUUCAGACAGGCGGA	[65]
mir-122	hsa-miR-122-5p (hsa-miR-122) [65]	URS00003380CC_9606	UGGAGUGUGACAAUGGUGUUUG	[59, 65]
mir-1225	hsa-miR-1225-3p	URS000075D62D_9606	UGAGCCCCUGUGCCGCCCCCAG	[65]
	hsa-miR-1225-5p	URS000075D0F5_9606	GUGGGUACGGCCCAGUGGGGGG	[72]
mir-1226	hsa-miR-1226-5p	URS000075EAB0_9606	GUGAGGGCAUGCAGGCCUGGAUGGGG	[65]
mir-1227	hsa-miR-1227-3p (hsa-miR-1227)	URS000075CFA8_9606	CGUGCCACCCUUUUCCCCAG	[65]
mir-1228	hsa-miR-1228-5p (hsa-miR-1228*) [65]	URS00004F1E01_9606	GUGGGCGGGGGCAGGUGUGUG	[65, 67]
mir-1233	hsa-miR-1233-3p (hsa-miR-1233)	URS000075D36A_9606	UGAGCCCUGUCCUCCCGCAG	[65]
mir-1238	hsa-miR-1238-3p (hsa-miR-1238)	URS000075E57E_9606	CUUCCUCGUCUGUCUGCCCC	[65]
mir-124	hsa-miR-124-3p	URS000020BE6A_9606	UAAGGCACGCGGUGAAUGCC	[54]
mir-1244	hsa-miR-1244	URS000075B58F_9606	AAGUAGUUGGUUUGUAUGAGAUGGUU	[65]
mir-1246	hsa-miR-1246	URS000028C188_9606	AAUGGAUUUUUGGAGCAGG	[54, 66, 72]
mir-1247	hsa-miR-1247-3p	URS000032835F_9606	CCCCGGGAACGUCGAGACUGGAGC	[67]
	hsa-miR-1247-5p (hsa-miR-1247) [65]	URS000057DF36_9606	ACCCGUCCCGUUCGUCCCCGGA	[65, 67]
mir-1249	hsa-miR-1249-3p (hsa-miR-1249)	URS000060AABB_9606	ACGCCCUUCCCCCCCUUCUUCA	[65]
mir-1253	hsa-miR-1253	URS000075A7EC_9606	AGAGAAGAAGAUCAGCCUGCA	[65]
mir-1254	hsa-miR-1254	URS000047047A_9606	AGCCUGGAAGCUGGAGCCUGCAGU	[54, 64]
mir-1255	hsa-miR-1255b-5p (hsa-miR-1255b)	URS0000211070_9606	CGGAUGAGCAAAGAAAGUGGUU	[65]
mir-1256	hsa-miR-1256	URS0000098B3B_9606	AGGCAUUGACUUCUCACUAGCU	[65]
mir-126	hsa-miR-126-3p (hsa-miR-126) [65]	URS00001F1DA8_9606	UCGUACCGUGAGUAAUAAUGCG	[54, 65]
	hsa-miR-126-5p (hsa-miR-126*) [65]	URS00001D69F6_9606	CAUUAUUACUUUUGGUACGCG	[54, 65]
mir-1260a	hsa-miR-1260a (hsa-miR-1260)	URS00000D0874_9606	AUCCCACCUCUGCCACCA	[65]
mir-1260b	hsa-miR-1260b	URS0000239117_9606	AUCCCACCACUGCCACCAU	[12]
mir-1262	hsa-miR-1262	URS0000568FF8_9606	AUGGGUGAAUUUGUAGAAGGAU	[65]
mir-1267	hsa-miR-1267	URS000075AEB2_9606	CCUGUUGAAGUGUAAUCCCCA	[65]
mir-1268	hsa-miR-1268a	URS00005A8A8D_9606	CGGGCGUGGUGGUGGGGG	[72]
mir-127	hsa-miR-127-3p (hsa-miR-127) [65]	URS00001E3DAA_9606	UCGGAUCCGUCUGAGCUUGGCU	[11, 54, 65, 71]
mir-1270	hsa-miR-1270	URS00002E0524_9606	CUGGAGAUAUGGAAGAGCUGUGU	[65]
mir-1271	hsa-miR-1271-5p (hsa-miR-1271)	URS00001F61BA_9606	CUUGGCACCUAGCAAGCACUCA	[65]
mir-1272	hsa-miR-1272	URS00000E1E9E_9606	GAUGAUGAUGGCAGCAAAUUCUGAAA	[65]
mir-1273	hsa-miR-1273a	–	GGGCGACAAAGCAAGACUCUUUCUU	[54, 66]
	hsa-miR-1273d	URS00003CF845_9606	GAACCCAUGAGGUUGAGGCUGCAGU	[54]
	hsa-miR-1273e	URS0000361F30_9606	UUGCUUGAACCCAGGAAGUGGA	[54]
	hsa-miR-1273f	URS00003DD70F_9606	GGAGAUGGAGGUUGCAGUG	[54, 66]
	hsa-miR-1273g-3p	URS00002B60FB_9606	ACCACUGCACUCCAGCCUGAG	[12, 54, 66]
miR-1275	hsa-miR-1275	URS000009EA8F_9606	GUGGGGGAGAGGCUGUC	[65, 73]
mir-128	hsa-miR-128-1-5p	URS0000537082_9606	CGGGGCCGUAGCACUGUCUGAGA	[67]
	hsa-miR-128-3p (hsa-miR-128a) [65]	URS000024A59E_9606	UCACAGUGAACCGGUCUCUUU	[54, 65]
mir-1285	hsa-miR-1285-3p (hsa-miR-1285) [65]	URS0000399545_9606	UCUGGGCAACAAAGUGAGACCU	[65, 66]
	hsa-miR-1285-5p	URS000050A3A3_9606	GAUCUCACUUUGUUGCCCAGG	[54, 66]
mir-129	hsa-miR-129-2-3p	URS000048F59D_9606	AAGCCCUUACCCCAAAAAGCAU	[54]
	hsa-miR-129-5p	URS00004E1410_9606	CUUUUUGCGGUCUGGGCUUGC	[54]
mir-1290	hsa-miR-1290	URS000043F369_9606	UGGAUUUUUGGAUCAGGGA	[54, 65, 66]
mir-1291	hsa-miR-1291	URS000047E28E_9606	UGGCCCUGACUGAAGACCAGCAGU	[54, 65]
mir-1292	hsa-miR-1292-5p	URS00005586D0_9606	UGGGAACGGGUUCCGGCAGACGCUG	[67]
mir-130	hsa-miR-130a-3p (hsa-miR-130a)	URS0000315338_9606	CAGUGCAAUGUUAAAAGGGCAU	[65]
	hsa-miR-130b-5p (hsa-miR-130b*)	URS000032A4F7_9606	ACUCUUUCCCUGUUGCACUAC	[65]
	hsa-miR-130b-3p (hsa-miR-130b)	URS00002C0FCB_9606	CAGUGCAAUGAUGAAAGGGCAU	[65]
	hsa-miR-301a-3p (hsa-miR-301)	URS00001C11BC_9606	CAGUGCAAUAGUAUUGUCAAAGC	[65]
	hsa-miR-301b-3p (hsa-miR-301b)	URS0000251D0B_9606	CAGUGCAAUGAUAUUGUCAAAGC	[65]
mir-1303	hsa-miR-1303	URS000032FC1A_9606	UUUAGAGACGGGGUCUUGCUCU	[54, 65]
mir-1305	hsa-miR-1305	URS000040EC3B_9606	UUUUCAACUCUAAUGGGAGAGA	[65]
mir-1306	hsa-miR-1306-5p	URS0000500449_9606	CCACCUCCCCUGCAAACGUCCA	[67]
mir-1307	hsa-miR-1307-5p	URS00000EEF5F_9606	UCGACCGGACCUCGACCGGCU	[54]
mir-132	hsa-miR-132-3p (hsa-miR-132)	URS00006054DA_9606	UAACAGUCUACAGCCAUGGUCG	[65]
	hsa-miR-212-3p (hsa-miR-212)	URS00001D6BAE_9606	UAACAGUCUCCAGUCACGGCC	[65]
	hsa-miR-212-5p	URS00001AFC71_9606	ACCUUGGCUCUAGACUGCUUACU	[54]
mir-134	hsa-miR-134-5p	URS0000272A92_9606	UGUGACUGGUUGACCAGAGGGG	[54, 59]
mir-1343	hsa-miR-1343-5p	URS0000759B67_9606	UGGGGAGCGGCCCCCGGGUGGG	[67]
mir-135	hsa-miR-135b-3p (hsa-miR-135b*)	URS0000488C83_9606	AUGUAGGGCUAAAAGCCAUGGG	[65]
	hsa-miR-135b-5p (hsa-miR-135b) [65]	URS000001C659_9606	UAUGGCUUUUCAUUCCUAUGUGA	[65, 71]
mir-136	hsa-miR-136-3p (hsa-miR-136*) [65]	URS0000204177_9606	CAUCAUCGUCUCAAAUGAGUCU	[54, 65]
	hsa-miR-136-5p (hsa-miR-136) [65]	URS00004EAB18_9606	ACUCCAUUUGUUUUGAUGAUGGA	[54, 65]
mir-138	hsa-miR-138-2-3p (hsa-miR-138-2*)	URS000075AA94_9606	GCUAUUUCACGACACCAGGGUU	[65]
	hsa-miR-138-5p (hsa-miR-138) [65]	URS000040780F_9606	AGCUGGUGUUGUGAAUCAGGCCG	[54, 65]
mir-139	hsa-miR-139-3p	URS000023BE29_9606	UGGAGACGCGGCCCUGUUGGAGU	[65]
	hsa-miR-139-5p	URS000025D232_9606	UCUACAGUGCACGUGUCUCCAGU	[54, 65]
mir-140	hsa-miR-140-3p	URS00000821E0_9606	UACCACAGGGUAGAACCACGG	[54, 65]
mir-142	hsa-miR-142-3p	URS00002620A7_9606	UGUAGUGUUUCCUACUUUAUGGA	[65]
	hsa-miR-142-5p	URS00001E0AEA_9606	CAUAAAGUAGAAAGCACUACU	[65]
mir-143	hsa-miR-143-3p (hsa-miR-143) [65]	URS00005C2A6D_9606	UGAGAUGAAGCACUGUAGCUC	[11, 53, 54, 65]
mir-144	hsa-miR-144-3p (hsa-miR-144) [65]	URS000037C5A8_9606	UACAGUAUAGAUGAUGUACU	[53, 54, 65]
	hsa-miR-144-5p (hsa-miR-144*)	URS00002E92A8_9606	GGAUAUCAUCAUAUACUGUAAG	[65]
mir-145	hsa-miR-145-3p (hsa-miR-145*)	URS000052F380_9606	GGAUUCCUGGAAAUACUGUUCU	[65]
	hsa-miR-145-5p (hsa-miR-145) [65]	URS0000527F89_9606	GUCCAGUUUUCCCAGGAAUCCCU	[12, 65, 66]
mir-146	hsa-miR-146a-5p (hsa-miR-146a) [65]	URS000050B527_9606	UGAGAACUGAAUUCCAUGGGUU	[11, 65, 69–71]
	hsa-miR-146b-3p	URS000050CCE0_9606	UGCCCUGUGGACUCAGUUCUGG	[65]
	hsa-miR-146b-5p (hsa-miR-146b) [65]	URS000061B694_9606	UGAGAACUGAAUUCCAUAGGCU	[11, 65]
mir-1468	hsa-miR-1468-5p	URS00002ECEE4_9606	CUCCGUUUGCCUGUUUCGCUG	[54]
mir-148	hsa-miR-148a-3p (hsa-miR-148a) [63, 64, 70]	URS00003BBF48_9606	UCAGUGCACUACAGAACUUUGU	[54, 64, 65, 74]
	hsa-miR-148a-5p (hsa-miR-148a*)	URS00003E16E5_9606	AAAGUUCUGAGACACUCCGACU	[65]
	hsa-miR-148b-3p (hsa-miR-148b)	URS0000521626_9606	UCAGUGCAUCACAGAACUUUGU	[65]
	hsa-miR-148b-5p (hsa-miR-148b*)	URS00005A7A84_9606	AAGUUCUGUUAUACACUCAGGC	[65]
	hsa-miR-152-3p	URS00003AFD9B_9606	UCAGUGCAUGACAGAACUUGG	[53, 59]
mir-149	hsa-miR-149-3p	URS000042C6A6_9606	AGGGAGGGACGGGGGCUGUGC	[12, 67]
	hsa-miR-149-5p (hsa-miR-149)	URS00001C770D_9606	UCUGGCUCCGUGUCUUCACUCCC	[65]
mir-15	hsa-miR-15a-3p (hsa-miR-15a*)	URS00001C94E0_9606	CAGGCCAUAUUGUGCUGCCUCA	[65]
	hsa-miR-15a-5p (hsa-miR-15a)	URS00003D1AE3_9606	UAGCAGCACAUAAUGGUUUGUG	[65]
	hsa-miR-15b-3p (hsa-miR-15b*)	URS000045A9D7_9606	CGAAUCAUUAUUUGCUGCUCUA	[65]
	hsa-miR-15b-5p (hsa-miR-15b) [65]	URS00004AD914_9606	UAGCAGCACAUCAUGGUUUACA	[65, 72]
	hsa-miR-16-1-3p (hsa-miR-16-1*)	URS000061CB8F_9606	CCAGUAUUAACUGUGCUGCUGA	[65]
	hsa-miR-16-2-3p (hsa-miR-16-2*)	URS00001E9CCB_9606	CCAAUAUUACUGUGCUGCUUUA	[65]
	hsa-miR-16-5p (hsa-miR-16) [65]	URS00004BCD9C_9606	UAGCAGCACGUAAAUAUUGGCG	[12, 54, 59, 65, 69]
	hsa-miR-195-3p	URS0000476C64_9606	CCAAUAUUGGCUGUGCUGCUCC	[53]
	hsa-miR-195-5p (hsa-miR-195)	URS00005B3525_9606	UAGCAGCACAGAAAUAUUGGC	[65]
mir-150	hsa-miR-150-5p (hsa-miR-150)	URS000016FD1A_9606	UCUCCCAACCCUUGUACCAGUG	[65]
mir-153	hsa-miR-153-3p	URS0000068B85_9606	UUGCAUAGUCACAAAAGUGAUC	[54]
mir-1538	hsa-miR-1538	URS00005235AA_9606	CGGCCCGGGCUGCUGCUGUUCCU	[67]
mir-154	hsa-miR-154-3p (hsa-miR-154*)	URS00000C0921_9606	AAUCAUACACGGUUGACCUAUU	[65]
	hsa-miR-323a-3p (hsa-miR-323-3p)	URS00003CCAB4_9606	CACAUUACACGGUCGACCUCU	[65]
	hsa-miR-323b-5p (hsa-miR-453)	URS000075D04C_9606	AGGUUGUCCGUGGUGAGUUCGCA	[65]
	hsa-miR-369-3p	URS0000442B0D_9606	AAUAAUACAUGGUUGAUCUUU	[65]
	hsa-miR-369-5p	URS00002A71AD_9606	AGAUCGACCGUGUUAUAUUCGC	[65]
	hsa-miR-377-5p (hsa-miR-377*)	URS000036BEF1_9606	AGAGGUUGCCCUUGGUGAAUUC	[65]
	hsa-miR-381–3p	URS00001FFA8C_9606	UAUACAAGGGCAAGCUCUCUGU	[54]
	hsa-miR-382-5p (hsa-miR-382)	URS000035E174_9606	GAAGUUGUUCGUGGUGGAUUCG	[65]
	hsa-miR-409-3p	URS00002915C8_9606	GAAUGUUGCUCGGUGAACCCCU	[54, 65]
	hsa-miR-409-5p	URS0000081E1F_9606	AGGUUACCCGAGCAACUUUGCAU	[54, 65]
	hsa-miR-410-3p	URS000047E765_9606	AAUAUAACACAGAUGGCCUGU	[54]
	hsa-miR-539-5p (hsa-miR-539)	URS00003E59B7_9606	GGAGAAAUUAUCCUUGGUGUGU	[65]
mir-155	hsa-miR-155-5p (hsa-miR-155) [65]	URS0000338542_9606	UUAAUGCUAAUCGUGAUAGGGGU	[65, 71]
mir-17	hsa-miR-106a-5p (hsa-miR-106a)	URS00003FE4D4_9606	AAAAGUGCUUACAGUGCAGGUAG	[65]
	hsa-miR-106b-3p (hsa-miR-106b*)	URS0000384021_9606	CCGCACUGUGGGUACUUGCUGC	[65]
	hsa-miR-106b-5p (hsa-miR-106b)	URS00004449AE_9606	UAAAGUGCUGACAGUGCAGAU	[65]
	hsa-miR-17-3p (hsa-miR-17*)	URS00004636A3_9606	ACUGCAGUGAAGGCACUUGUAG	[65]
	hsa-miR-17-5p (hsa-miR-17)	URS00002075FA_9606	CAAAGUGCUUACAGUGCAGGUAG	[65]
	hsa-miR-18a-3p (hsa-miR-18a*)	URS00004131FE_9606	ACUGCCCUAAGUGCUCCUUCUGG	[65]
	hsa-miR-18a-5p (hsa-miR-18a)	URS000035CC3E_9606	UAAGGUGCAUCUAGUGCAGAUAG	[65]
	hsa-miR-18b-5p (hsa-miR-18b)	URS00004565E5_9606	UAAGGUGCAUCUAGUGCAGUUAG	[65]
	hsa-miR-20a-3p (hsa-miR-20a*)	URS0000042E1F_9606	ACUGCAUUAUGAGCACUUAAAG	[65]
	hsa-miR-20a-5p (hsa-miR-20a) [65]	URS0000574A2C_9606	UAAAGUGCUUAUAGUGCAGGUAG	[65, 72]
mir-17	hsa-miR-20b-5p (hsa-miR-20b)	URS00002B3783_9606	CAAAGUGCUCAUAGUGCAGGUAG	[65]
	hsa-miR-93-3p (hsa-miR-93*)	URS00000FB1B1_9606	ACUGCUGAGCUAGCACUUCCCG	[65]
	hsa-miR-93-5p	URS0000149452_9606	CAAAGUGCUGUUCGUGCAGGUAG	[54, 59]
mir-181	hsa-miR-181a-2-3p (hsa-miR-181a-2*)	URS0000241987_9606	ACCACUGACCGUUGACUGUACC	[65]
	hsa-miR-181a-3p (hsa-miR-213)	URS000003F252_9606	ACCAUCGACCGUUGAUUGUACC	[65]
	hsa-miR-181a-5p (hsa-miR-181a) [65]	URS00003DA300_9606	AACAUUCAACGCUGUCGGUGAGU	[54, 65]
	hsa-miR-181b-5p	URS0000605E00_9606	AACAUUCAUUGCUGUCGGUGGGU	[54]
	hsa-miR-181c-3p (hsa-miR-181c*)	URS0000244A71_9606	AACCAUCGACCGUUGAGUGGAC	[65]
	hsa-miR-181c-5p (hsa-miR-181c) [65]	URS000018C928_9606	AACAUUCAACCUGUCGGUGAGU	[54, 65]
mir-182	hsa-miR-182-5p (hsa-miR-182)	URS00001CC379_9606	UUUGGCAAUGGUAGAACUCACACU	[65]
mir-1825	hsa-miR-1825	URS000075AF4A_9606	UCCAGUGCCCUCCUCUCC	[65]
mir-183	hsa-miR-183-3p	URS0000345DEB_9606	GUGAAUUACCGAAGGGCCAUAA	[65]
	hsa-miR-183-5p (hsa-miR-183)	URS0000528CBC_9606	UAUGGCACUGGUAGAAUUCACU	[65]
mir-184	hsa-miR-184	URS0000543D82_9606	UGGACGGAGAACUGAUAAGGGU	[65]
mir-185	hsa-miR-185-3p	URS00002367FA_9606	AGGGGCUGGCUUUCCUCUGGUC	[67]
	hsa-miR-185-5p (hsa-miR-185)	URS00004176D4_9606	UGGAGAGAAAGGCAGUUCCUGA	[65, 70]
mir-186	hsa-miR-186-5p (hsa-miR-186) [65, 70]	URS000040DCFF_9606	CAAAGAAUUCUCCUUUUGGGCU	[54, 65, 70]
mir-188	hsa-miR-532-3p	URS00004B4B85_9606	CCUCCCACACCCAAGGCUUGCA	[65, 67]
	hsa-miR-532-5p (hsa-miR-532)	URS00004E8341_9606	CAUGCCUUGAGUGUAGGACCGU	[65, 70]
	hsa-miR-660-5p (hsa-miR-660)	URS0000116A70_9606	UACCCAUUGCAUAUCGGAGUUG	[65, 70]
mir-19	hsa-miR-19a-3p (hsa-miR-19a)	URS000006FDD4_9606	UGUGCAAAUCUAUGCAAAACUGA	[65, 70]
	hsa-miR-19b-1-5p (hsa-miR-19b-1*)	URS00001B9622_9606	AGUUUUGCAGGUUUGCAUCCAGC	[65]
	hsa-miR-19b-3p (hsa-miR-19b) [65, 70]	URS000013D17D_9606	UGUGCAAAUCCAUGCAAAACUGA	[65, 66, 70, 72]
mir-190	hsa-miR-190a-5p (hsa-miR-190)	URS0000520927_9606	UGAUAUGUUUGAUAUAUUAGGU	[65]
mir-1908	hsa-miR-1908-3p	URS000075E4A7_9606	CCGGCCGCCGGCUCCGCCCCG	[54]
	hsa-miR-1908-5p	URS00002373FD_9606	CGGCGGGGACGGCGAUUGGUC	[67]
mir-191	hsa-miR-191–3p (hsa-miR-191*)	URS00002B2B5C_9606	GCUGCGCUUGGAUUUCGUCCCC	[65]
	hsa-miR-191-5p (hsa-miR-191) [65, 70]	URS00005C2E31_9606	CAACGGAAUCCCAAAAGCAGCUG	[11, 54, 65, 66, 70]
mir-1914	hsa-miR-1914-3p	URS000075E34C_9606	GGAGGGGUCCCGCACUGGGAGG	[67]
mir-1915	hsa-miR-1915-3p	URS000039BFD2_9606	CCCCAGGGCGACGCGGCGGG	[12, 72]
mir-192	hsa-miR-192-3p (hsa-miR-192*)	URS00000B59A2_9606	CUGCCAAUUCCAUAGGUCACAG	[65]
	hsa-miR-192-5p (hsa-miR-192) [65]	URS0000155642_9606	CUGACCUAUGAAUUGACAGCC	[54, 65]
mir-193	hsa-miR-193a-3p	URS00005DBAF3_9606	AACUGGCCUACAAAGUCCCAGU	[65]
	hsa-miR-193a-5p	URS0000367985_9606	UGGGUCUUUGCGGGCGAGAUGA	[54, 65, 66]
	hsa-miR-193b-3p (hsa-miR-193b)	URS00000AA464_9606	AACUGGCCCUCAAAGUCCCGCU	[65]
	hsa-miR-193b-5p (hsa-miR-193b*) [65]	URS00000E1DC5_9606	CGGGGUUUUGAGGGCGAGAUGA	[53, 65]
mir-194	hsa-miR-194-5p (hsa-miR-194)	URS000029C2DC_9606	UGUAACAGCAACUCCAUGUGGA	[65]
mir-196	hsa-miR-196a-5p	URS00000DA6A7_9606	UAGGUAGUUUCAUGUUGUUGGG	[53, 59]
	hsa-miR-196b-5p (hsa-miR-196b) [65]	URS0000611746_9606	UAGGUAGUUUCCUGUUGUUGGG	[53, 65]
mir-197	hsa-miR-197-3p (hsa-miR-197)	URS000061E740_9606	UUCACCACCUUCUCCACCCAGC	[65]
	hsa-miR-197-5p	URS000020E2DD_9606	CGGGUAGAGAGGGCAGUGGGAGG	[67]
mir-1972	hsa-miR-1972	URS000042A1A2_9606	UCAGGCCAGGCACAGUGGCUCA	[54, 66]
mir-198	hsa-miR-198	URS000075CAC3_9606	GGUCCAGAGGGGAGAUAGGUUC	[65]
mir-199	hsa-miR-199a-5p (hsa-miR-199a) [65]	URS0000554A4F_9606	CCCAGUGUUCAGACUACCUGUUC	[53, 54, 65]
	hsa-miR-199b-3p hsa-miR-199a-3p^##^	URS00003F2D94_9606	ACAGUAGUCUGCACAUUGGUUA	[53, 65, 66, 72]
	hsa-miR-199b-5p (hsa-miR-199b) [65, 67]	URS0000029EBD_9606	CCCAGUGUUUAGACUAUCUGUUC	[53, 65, 67]
mir-203	hsa-miR-203a-3p	URS00004DA9DB_9606	GUGAAAUGUUUAGGACCACUAG	[65]
mir-204	hsa-miR-204-3p	URS000059A01D_9606	GCUGGGAAGGCAAAGGGACGU	[54]
	hsa-miR-204-5p (hsa-miR-204) [65]	URS000029D9F1_9606	UUCCCUUUGUCAUCCUAUGCCU	[54, 65]
mir-205	hsa-miR-205-5p (hsa-miR-205) [65, 68]	URS0000446722_9606	UCCUUCAUUCCACCGGAGUCUG	[54, 65, 68]
mir-21	hsa-miR-21–3p (hsa-miR-21*) [65]	URS000009262D_9606	CAACACCAGUCGAUGGGCUGU	[54, 65]
	hsa-miR-21-5p (hsa-miR-21) [59, 65, 67, 74]	URS000039ED8D_9606	UAGCUUAUCAGACUGAUGUUGA	[11, 54, 59, 65–67, 71, 73, 74]
mir-210	hsa-miR-210-5p	URS000075D16F_9606	AGCCCCUGCCCACCGCACACUG	[67]
mir-214	hsa-miR-214-3p (hsa-miR-214) [65]	URS00002C11C3_9606	ACAGCAGGCACAGACAGGCAGU	[12, 65, 66]
	hsa-miR-214-5p (hsa-miR-214*)	URS00004DAA89_9606	UGCCUGUCUACACUUGCUGUGC	[65]
mir-216	hsa-miR-216a-5p (hsa-miR-216a)	URS0000318E24_9606	UAAUCUCAGCUGGCAACUGUGA	[65]
mir-218	hsa-miR-218-2-3p (hsa-miR-218-2*)	URS00001F9A0F_9606	CAUGGUUCUGUCAAGCACCGCG	[65]
	hsa-miR-218-5p	URS000020D84A_9606	UUGUGCUUGAUCUAACCAUGU	[54]
mir-219	hsa-miR-219a-5p (hsa-miR-219)	URS0000565C8D_9606	UGAUUGUCCAAACGCAAUUCU	[65]
mir-22	hsa-miR-22-3p (hsa-miR-22) [65]	URS0000096022_9606	AAGCUGCCAGUUGAAGAACUGU	[11, 12, 53, 54, 65]
	hsa-miR-22-5p (hsa-miR-22*) [65]	URS0000142DC3_9606	AGUUCUUCAGUGGCAAGCUUUA	[65, 70]
mir-221	hsa-miR-221–3p (hsa-miR-221) [65]	URS0000170CF4_9606	AGCUACAUUGUCUGCUGGGUUUC	[12, 54, 59, 65, 66, 69]
	hsa-miR-222-3p (hsa-miR-222) [63, 64, 66]	URS00002C6949_9606	AGCUACAUCUGGCUACUGGGU	[11, 12, 54, 59, 64, 65, 70]
	hsa-miR-222-5p (hsa-miR-222*)	URS0000153377_9606	CUCAGUAGCCAGUGUAGAUCCU	[65]
mir-223	hsa-miR-223-3p (hsa-miR-223)	URS00000B7E30_9606	UGUCAGUUUGUCAAAUACCCCA	[65]
	hsa-miR-223-5p (hsa-miR-223*)	URS0000485CBB_9606	CGUGUAUUUGACAAGCUGAGUU	[65]
mir-224	hsa-miR-224-5p (hsa-miR-224)	URS00002BBD4E_9606	CAAGUCACUAGUGGUUCCGUU	[65]
mir-23	hsa-miR-23a-3p (hsa-miR-23a) [64, 65]	URS00005540D2_9606	AUCACAUUGCCAGGGAUUUCC	[12, 59, 64–66, 69, 72]
	hsa-miR-23b-3p	URS0000183BED_9606	AUCACAUUGCCAGGGAUUACC	[12, 54, 59, 66]
mir-24	hsa-miR-24-1-5p (hsa-miR-24-1*)	URS00002D0FC3_9606	UGCCUACUGAGCUGAUAUCAGU	[65]
	hsa-miR-24-2-5p (hsa-miR-24-2*)	URS00001DEE11_9606	UGCCUACUGAGCUGAAACACAG	[65]
	hsa-miR-24-3p (hsa-miR-24) [65]	URS000059273E_9606	UGGCUCAGUUCAGCAGGAACAG	[12, 53, 59, 65, 66]
mir-25	hsa-miR-25-3p (hsa-miR-25) [65]	URS00004F9744_9606	CAUUGCACUUGUCUCGGUCUGA	[54, 65, 66]
	hsa-miR-25-5p (hsa-miR-25*)	URS00001A9746_9606	AGGCGGAGACUUGGGCAAUUG	[65]
	hsa-miR-92a-3p (hsa-miR-92a) [65]	URS00003768C5_9606	UAUUGCACUUGUCCCGGCCUGU	[11, 54, 65, 66]
	hsa-miR-92b-3p	URS000025576D_9606	UAUUGCACUCGUCCCGGCCUCC	[11, 54]
	hsa-miR-92b-5p (hsa-miR-92b*)	URS00001A7F58_9606	AGGGACGGGACGCGGUGCAGUG	[65]
mir-26	hsa-miR-26b-3p (hsa-miR-26b*)	URS000021C6A8_9606	CCUGUUCUCCAUUACUUGGCU	[65]
	hsa-miR-26b-5p (hsa-miR-26b)	URS0000316FA5_9606	UUCAAGUAAUUCAGGAUAGGU	[65]
	hsa-miR-26a-1-3p (hsa-miR-26a-1*)	URS00000C0D3F_9606	CCUAUUCUUGGUUACUUGCACG	[65]
	hsa-miR-26a-5p (hsa-miR-26a) [65]	URS000019B0F7_9606	UUCAAGUAAUCCAGGAUAGGCU	[11, 54, 65, 69]
mir-27	hsa-miR-27a-3p (hsa-miR-27a) [65]	URS00003B95DA_9606	UUCACAGUGGCUAAGUUCCGC	[12, 53, 65]
	hsa-miR-27a-5p (hsa-miR-27a*) [65]	URS00001B341F_9606	AGGGCUUAGCUGCUUGUGAGCA	[65, 70]
	hsa-miR-27b-3p (hsa-miR-27b) [65]	URS000059311D_9606	UUCACAGUGGCUAAGUUCUGC	[54, 65]
	hsa-miR-27b-5p (hsa-miR-27b*)	URS0000330617_9606	AGAGCUUAGCUGAUUGGUGAAC	[65]
mir-28	hsa-miR-151a-3p (hsa-miR-151–3p) [65]	URS000016C318_9606	CUAGACUGAAGCUCCUUGAGG	[11, 54, 65]
	hsa-miR-151a-5p (hsa-miR-151-5p) [65]	URS00005F8E5B_9606	UCGAGGAGCUCACAGUCUAGU	[54, 65]
	hsa-miR-151b	URS00003E6479_9606	UCGAGGAGCUCACAGUCU	[54]
	hsa-miR-28-3p	URS00001799A3_9606	CACUAGAUUGUGAGCUCCUGGA	[54, 65]
	hsa-miR-28-5p (hsa-miR-28)	URS00003E47B1_9606	AAGGAGCUCACAGUCUAUUGAG	[65]
mir-2861	hsa-miR-2861	URS00003B13B8_9606	GGGGCCUGGCGGUGGGCGG	[72]
mir-29	hsa-miR-29a-3p (hsa-miR-29a) [65]	URS00002F4D78_9606	UAGCACCAUCUGAAAUCGGUUA	[54, 65]
	hsa-miR-29a-5p (hsa-miR-29a*) [65]	URS0000076995_9606	ACUGAUUUCUUUUGGUGUUCAG	[65, 70]
	hsa-miR-29b-1-5p (hsa-miR-29b-1*)	URS00001123BD_9606	GCUGGUUUCAUAUGGUGGUUUAGA	[65]
	hsa-miR-29b-2-5p (hsa-miR-29b-2*)	URS0000403C02_9606	CUGGUUUCACAUGGUGGCUUAG	[65]
	hsa-miR-29b-3p (hsa-miR-29b) [65]	URS000024463E_9606	UAGCACCAUUUGAAAUCAGUGUU	[54, 65]
	hsa-miR-29c-3p (hsa-miR-29c) [65]	URS0000272A3D_9606	UAGCACCAUUUGAAAUCGGUUA	[54, 65]
mir-296	hsa-miR-296-5p (hsa-miR-296) [65]	URS00001C3AC1_9606	AGGGCCCCCCCUCAAUCCUGU	[65, 67]
mir-299	hsa-miR-299-3p	URS00003B1F5C_9606	UAUGUGGGAUGGUAAACCGCUU	[54, 65]
	hsa-miR-299-5p	URS000017DBB8_9606	UGGUUUACCGUCCCACAUACAU	[65]
mir-30	hsa-miR-30a-3p	URS0000065D58_9606	CUUUCAGUCGGAUGUUUGCAGC	[65]
	hsa-miR-30a-5p	URS000043D1A9_9606	UGUAAACAUCCUCGACUGGAAG	[54, 65]
	hsa-miR-30b-5p (hsa-miR-30b)	URS00005165DA_9606	UGUAAACAUCCUACACUCAGCU	[65, 70]
	hsa-miR-30c-5p (hsa-miR-30c) [65]	URS000019907A_9606	UGUAAACAUCCUACACUCUCAGC	[54, 65]
	hsa-miR-30d-3p (hsa-miR-30d*)	URS00004B2A47_9606	CUUUCAGUCAGAUGUUUGCUGC	[65]
	hsa-miR-30d-5p (hsa-miR-30d) [65]	URS000005CF5F_9606	UGUAAACAUCCCCGACUGGAAG	[54, 65]
	hsa-miR-30e-3p	URS00004DC6A5_9606	CUUUCAGUCGGAUGUUUACAGC	[65, 70]
	hsa-miR-30e-5p	URS00001DE669_9606	UGUAAACAUCCUUGACUGGAAG	[54]
mir-302	hsa-miR-302a-3p (hsa-miR-302a)	URS0000070CD2_9606	UAAGUGCUUCCAUGUUUUGGUGA	[65]
	hsa-miR-302c-3p (hsa-miR-302c)	URS000027080C_9606	UAAGUGCUUCCAUGUUUCAGUGG	[65]
	hsa-miR-302d-3p (hsa-miR-302d)	URS000041E949_9606	UAAGUGCUUCCAUGUUUGAGUGU	[65]
mir-31	hsa-miR-31–3p (hsa-miR-31*)	URS00002A291B_9606	UGCUAUGCCAACAUAUUGCCAU	[65]
	hsa-miR-31-5p (hsa-miR-31) [65]	URS00005416E3_9606	AGGCAAGAUGCUGGCAUAGCU	[12, 59, 65]
mir-3180	hsa-miR-3180-3p	URS00002C4233_9606	UGGGGCGGAGCUUCCGGAGGCC	[67]
mir-32	hsa-miR-32-5p (hsa-miR-32)	URS00004C47FB_9606	UAUUGCACAUUACUAAGUUGCA	[65]
mir-320	hsa-miR-320a-3p^#^ (hsa-miR-320) [65]	URS00003CF1AD_9606	AAAAGCUGGGUUGAGAGGGCGA	[54, 65]
	hsa-miR-320b	URS000058BF17_9606	AAAAGCUGGGUUGAGAGGGCAA	[54, 65]
	hsa-miR-320c	URS0000010D30_9606	AAAAGCUGGGUUGAGAGGGU	[54]
mir-322	hsa-miR-424-3p (hsa-miR-424*) [65]	URS00002BCF86_9606	CAAAACGUGAGGCGCUGCUAU	[54, 65]
	hsa-miR-424-5p (hsa-miR-424)	URS00000F0F49_9606	CAGCAGCAAUUCAUGUUUUGAA	[65]
mir-324	hsa-miR-324-3p	URS00004390F6_9606	ACUGCCCCAGGUGCUGCUGG	[65, 70]
	hsa-miR-324-5p	URS000075BEBE_9606	CGCAUCCCCUAGGGCAUUGGUG	[65]
mir-326	hsa-miR-326	URS00000A939F_9606	CCUCUGGGCCCUUCCUCCAG	[65]
mir-329	hsa-miR-543	URS000019F055_9606	AAACAUUCGCGGUGCACUUCUU	[65]
mir-33	hsa-miR-33a-3p (hsa-miR-33a*)	URS00003E3B82_9606	CAAUGUUUCCACAGUGCAUCAC	[65]
	hsa-miR-33a-5p (hsa-miR-33a)	URS0000483184_9606	GUGCAUUGUAGUUGCAUUGCA	[65]
	hsa-miR-33b-5p (hsa-miR-33b)	URS00004C8DD5_9606	GUGCAUUGCUGUUGCAUUGC	[65]
mir-330	hsa-miR-330-3p (hsa-miR-330)	URS000007A060_9606	GCAAAGCACACGGCCUGCAGAGA	[65]
	hsa-miR-330-5p	URS00003380C1_9606	UCUCUGGGCCUGUGUCUUAGGC	[65]
mir-331	hsa-miR-331–3p (hsa-miR-331)	URS00003DDE27_9606	GCCCCUGGGCCUAUCCUAGAA	[65]
	hsa-miR-331-5p	URS00001597DC_9606	CUAGGUAUGGUCCCAGGGAUCC	[65]
mir-335	hsa-miR-335-3p (hsa-miR-335*)	URS00005092C2_9606	UUUUUCAUUAUUGCUCCUGACC	[65]
	hsa-miR-335-5p (hsa-miR-335)	URS0000237AF9_9606	UCAAGAGCAAUAACGAAAAAUGU	[65]
mir-337	hsa-miR-337-3p	URS0000564D66_9606	CUCCUAUAUGAUGCCUUUCUUC	[65]
	hsa-miR-337-5p	URS0000306C70_9606	GAACGGCUUCAUACAGGAGUU	[65]
mir-338	hsa-miR-338-3p	URS00000254A6_9606	UCCAGCAUCAGUGAUUUUGUUG	[54, 65]
mir-339	hsa-miR-339-3p	URS000055B190_9606	UGAGCGCCUCGACGACAGAGCCG	[65]
	hsa-miR-339-5p	URS000003FD55_9606	UCCCUGUCCUCCAGGAGCUCACG	[54]
mir-34	hsa-miR-34a-3p (hsa-miR-34a*)	URS00000EED18_9606	CAAUCAGCAAGUAUACUGCCCU	[65]
	hsa-miR-34a-5p (hsa-miR-34a) [65]	URS000030BD69_9606	UGGCAGUGUCUUAGCUGGUUGU	[65, 71]
	hsa-miR-34b-3p (hsa-miR-34b)	URS000027352D_9606	CAAUCACUAACUCCACUGCCAU	[65]
	hsa-miR-34c-5p (hsa-miR-34c)	URS00002C7B2B_9606	AGGCAGUGUAGUUAGCUGAUUGC	[64, 65]
mir-340	hsa-miR-340-3p (hsa-miR-340*)	URS000048521E_9606	UCCGUCUCAGUUACUUUAUAGC	[65]
	hsa-miR-340-5p (hsa-miR-340)	URS0000007FBA_9606	UUAUAAAGCAAUGAGACUGAUU	[65]
mir-342	hsa-miR-342-3p	URS0000148B91_9606	UCUCACACAGAAAUCGCACCCGU	[65]
	hsa-miR-342-5p	URS00005A8080_9606	AGGGGUGCUAUCUGUGAUUGA	[65]
mir-345	hsa-miR-345-5p (hsa-miR-345)	URS000005D4F5_9606	GCUGACUCCUAGUCCAGGGCUC	[65]
mir-361	hsa-miR-361–3p	URS000031E6A1_9606	UCCCCCAGGUGUGAUUCUGAUUU	[65]
	hsa-miR-361-5p (hsa-miR-361) [65]	URS00000CF1D2_9606	UUAUCAGAAUCUCCAGGGGUAC	[65, 70]
mir-3613	hsa-miR-3613-3p	URS00004EAE33_9606	ACAAAAAAAAAAGCCCAACCCUUC	[12]
mir-3615	hsa-miR-3615	URS000011166D_9606	UCUCUCGGCUCCUCGCGGCUC	[54]
miR-362	hsa-miR-362-3p	URS00003A19A3_9606	AACACACCUAUUCAAGGAUUCA	[64, 65]
	hsa-miR-362-5p (hsa-miR-362)	URS0000085F64_9606	AAUCCUUGGAACCUAGGUGUGAGU	[65]
mir-3648	hsa-miR-3648	URS0000454FAB_9606	AGCCGCGGGGAUCGCCGAGGG	[54]
mir-365	hsa-miR-365a-3p (hsa-miR-365)	URS00003E7283_9606	UAAUGCCCCUAAAAAUCCUUAU	[65]
mir-3661	hsa-miR-3661	URS00002CCA6E_9606	UGACCUGGGACUCGGACAGCUG	[67]
mir-368	hsa-miR-376a-3p (hsa-miR-376a)	URS000041E11D_9606	AUCAUAGAGGAAAAUCCACGU	[65]
	hsa-miR-376a-5p (hsa-miR-376a*)	URS000032A93F_9606	GUAGAUUCUCCUUCUAUGAGUA	[65]
	hsa-miR-376b-3p (hsa-miR-376b)	URS00003AD231_9606	AUCAUAGAGGAAAAUCCAUGUU	[65]
	hsa-miR-376c-3p (hsa-miR-376c)	URS00005E651E_9606	AACAUAGAGGAAAUUCCACGU	[65]
mir-3687	hsa-miR-3687	URS0000420457_9606	CCCGGACAGGCGUUCGUGCGACGU	[54, 66]
mir-370	hsa-miR-370-3p	URS00004900F1_9606	GCCUGCUGGGGUGGAACCUGGU	[54]
mir-374	hsa-miR-374a-5p (hsa-miR-374)	URS000029E173_9606	UUAUAAUACAACCUGAUAAGUG	[65]
mir-375	hsa-miR-375-3p^#^	URS00000ED600_9606	UUUGUUCGUUCGGCUCGCGUGA	[54]
mir-378	hsa-miR-378a-3p (hsa-miR-378)[65]	URS00000451A1_9606	ACUGGACUUGGAGUCAGAAGGC	[54, 65]
mir-379	hsa-miR-380-5p	URS000075BE5F_9606	UGGUUGACCAUAGAACAUGCGC	[65]
	hsa-miR-411–3p (hsa-miR-411*)	URS000037DAEA_9606	UAUGUAACACGGUCCACUAACC	[65]
	hsa-miR-411-5p	URS00000C5BAA_9606	UAGUAGACCGUAUAGCGUACG	[54, 65]
	hsa-miR-758-3p (hsa-miR-758)	URS000024B619_9606	UUUGUGACCUGGUCCACUAACC	[65]
mir-384	hsa-miR-384	URS000075DD0E_9606	AUUCCUAGAAAUUGUUCAUA	[65]
mir-3934	hsa-miR-3934-5p	URS00003ACE11_9606	UCAGGUGUGGAAACUGAGGCAG	[72]
mir-3940	hsa-miR-3940-5p	URS00001E8DA7_9606	GUGGGUUGGGGCGGGCUCUG	[73]
mir-3960	hsa-miR-3960	URS00003783AB_9606	GGCGGCGGCGGAGGCGGGGG	[12, 54, 66]
mir-422	hsa-miR-422a	URS00003CC245_9606	ACUGGACUUAGGGUCAGAAGGC	[65]
mir-423	hsa-miR-423-3p	URS00000BE495_9606	AGCUCGGUCUGAGGCCCCUCAGU	[54]
	hsa-miR-423-5p	URS00001C8A86_9606	UGAGGGGCAGAGAGCGAGACUUU	[54, 65, 66, 69, 70]
mir-425	hsa-miR-425-3p (hsa-miR-425*)	URS000056B04E_9606	AUCGGGAAUGUCGUGUCCGCCC	[65]
	hsa-miR-425-5p	URS000048BA36_9606	AAUGACACGAUCACUCCCGUUGA	[65, 69]
mir-431	hsa-miR-431-5p (hsa-miR-431)	URS000043908D_9606	UGUCUUGCAGGCCGUCAUGCA	[65]
mir-432	hsa-miR-432-5p (hsa-miR-432)	URS00001C406A_9606	UCUUGGAGUAGGUCAUUGGGUGG	[65]
mir-4446	hsa-miR-4446-3p	URS000000EF0B_9606	CAGGGCUGGCAGUGACAUGGGU	[67]
mir-4449	hsa-miR-4449	URS00004DE2FC_9606	CGUCCCGGGGCUGCGCGAGGCA	[54, 67]
mir-4488	hsa-miR-4488	URS0000419B5A_9606	AGGGGGCGGGCUCCGGCG	[12, 54, 66]
mir-449	hsa-miR-449a (hsa-miR-449)	URS00001F5B39_9606	UGGCAGUGUAUUGUUAGCUGGU	[65]
	hsa-miR-449b-5p (hsa-miR-449b)	URS00003758F0_9606	AGGCAGUGUAUUGUUAGCUGGC	[65]
mir-450	hsa-miR-450a-5p (hsa-miR-450a)	URS00003E5ECC_9606	UUUUGCGAUGUGUUCCUAAUAU	[65]
	hsa-miR-450b-3p	URS00002FF522_9606	UUGGGAUCAUUUUGCAUCCAUA	[65]
	hsa-miR-450b-5p	URS0000422A99_9606	UUUUGCAAUAUGUUCCUGAAUA	[65]
mir-452	hsa-miR-452-5p (hsa-miR-452)	URS0000550C66_9606	AACUGUUUGCAGAGGAAACUGA	[65]
mir-454	hsa-miR-454-3p (hsa-miR-454)	URS00004F77ED_9606	UAGUGCAAUAUUGCUUAUAGGGU	[65]
	hsa-miR-454-5p (hsa-miR-454*)	URS000031602A_9606	ACCCUAUCAAUAUUGUCUCUGC	[65]
mir-455	hsa-miR-455-3p	URS000022A78C_9606	GCAGUCCAUGGGCAUAUACAC	[65]
	hsa-miR-455-5p (hsa-miR-455)	URS00000AD002_9606	UAUGUGCCUUUGGACUACAUCG	[65]
mir-483	hsa-miR-483-3p	URS00000EA063_9606	UCACUCCUCUCCUCCCGUCUU	[65]
	hsa-miR-483-5p	URS000003575B_9606	AAGACGGGAGGAAAGAAGGGAG	[65]
mir-484	hsa-miR-484	URS0000597BED_9606	UCAGGCUCAGUCCCCUCCCGAU	[54, 65, 67]
mir-485	hsa-miR-485-3p	URS000006372A_9606	GUCAUACACGGCUCUCCUCUCU	[65]
mir-485	hsa-miR-485-5p	URS00001935FA_9606	AGAGGCUGGCCGUGAUGAAUUC	[65]
mir-486	hsa-miR-486-5p (hsa-miR-486) [65]	URS00004BF1DC_9606	UCCUGUACUGAGCUGCCCCGAG	[11, 54, 65]
mir-488	hsa-miR-488-3p (hsa-miR-488)	URS00001BCAC5_9606	UUGAAAGGCUAUUUCUUGGUC	[65]
mir-492	hsa-miR-492	URS000032599B_9606	AGGACCUGCGGGACAAGAUUCUU	[65]
mir-493	hsa-miR-493-3p (hsa-miR-493)	URS00005E7CB2_9606	UGAAGGUCUACUGUGUGCCAGG	[65]
mir-497	hsa-miR-497-5p (hsa-miR-497)	URS00001BC212_9606	CAGCAGCACACUGUGGUUUGU	[65]
mir-500	hsa-miR-500a-5p (hsa-miR-500)	URS000039A052_9606	UAAUCCUUGCUACCUGGGUGAGA	[65]
	hsa-miR-501–3p	URS00000EEE35_9606	AAUGCACCCGGGCAAGGAUUCU	[65]
	hsa-miR-501-5p (hsa-miR-501)	URS00001E2DBC_9606	AAUCCUUUGUCCCUGGGUGAGA	[65]
	hsa-miR-502-3p	URS0000601CC4_9606	AAUGCACCUGGGCAAGGAUUCA	[65]
mir-503	hsa-miR-503-5p (hsa-miR-503)	URS00000F6E49_9606	UAGCAGCGGGAACAGUUCUGCAG	[65]
mir-505	hsa-miR-505-3p (hsa-miR-505)	URS00004A5A07_9606	CGUCAACACUUGCUGGUUUCCU	[65]
	hsa-miR-505-5p (hsa-miR-505*)	URS000017EA6A_9606	GGGAGCCAGGAAGUAUUGAUGU	[65]
mir-506	hsa-miR-508-3p (hsa-miR-508)	URS000044FE6A_9606	UGAUUGUAGCCUUUUGGAGUAGA	[65]
	hsa-miR-512-3p	URS000020F110_9606	AAGUGCUGUCAUAGCUGAGGUC	[65]
	hsa-miR-512-5p	URS0000062B37_9606	CACUCAGCCUUGAGGGCACUUUC	[65]
	hsa-miR-513a-5p (hsa-miR-513-5p)	URS0000357286_9606	UUCACAGGGAGGUGUCAU	[65]
mir-515	hsa-miR-517c-3p (hsa-miR-517c)	URS00003FBECA_9606	AUCGUGCAUCCUUUUAGAGUGU	[65]
	hsa-miR-520c-3p	URS000049A7EB_9606	AAAGUGCUUCCUUUUAGAGGGU	[65]
	hsa-miR-515-5p	URS00000A68B2_9606	UUCUCCAAAAGAAAGCACUUUCUG	[65]
	hsa-miR-516b-3p (hsa-miR-516-3p)	–	UGCUUCCUUUCAGAGGGU	[65]
	hsa-miR-517a-3p (hsa-miR-517a)	URS00000D4AB5_9606	AUCGUGCAUCCCUUUAGAGUGU	[65]
	hsa-miR-518a-3p	URS0000024ACC_9606	GAAAGCGCUUCCCUUUGCUGGA	[65]
	hsa-miR-518b	URS00003676C9_9606	CAAAGCGCUCCCCUUUAGAGGU	[65]
	hsa-miR-518d-3p (hsa-miR-518d)	URS00001B6361_9606	CAAAGCGCUUCCCUUUGGAGC	[65]
	hsa-miR-518f-3p (hsa-miR-518f)	URS000075E9BD_9606	GAAAGCGCUUCUCUUUAGAGG	[65]
	hsa-miR-519a-3p (hsa-miR-519a)	URS0000135E29_9606	AAAGUGCAUCCUUUUAGAGUGU	[65]
	hsa-miR-519b-3p	URS00003883FE_9606	AAAGUGCAUCCUUUUAGAGGUU	[65]
	hsa-miR-519e-5p (hsa-miR-519e*)	URS000075AC86_9606	UUCUCCAAAAGGGAGCACUUUC	[65]
	hsa-miR-520a-3p (hsa-miR-520a)	URS0000101689_9606	AAAGUGCUUCCCUUUGGACUGU	[65]
mir-541	hsa-miR-541–3p (hsa-miR-541)	URS000075A3AC_9606	UGGUGGGCACAGAAUCUGGACU	[65]
	hsa-miR-541-5p (hsa-miR-541*)	URS0000076E54_9606	AAAGGAUUCUGCUGUCGGUCCCACU	[65]
mir-542	hsa-miR-542-3p	URS00004F859B_9606	UGUGACAGAUUGAUAACUGAAA	[53, 64, 65]
	hsa-miR-542-5p	URS000050C722_9606	UCGGGGAUCAUCAUGUCACGAGA	[65]
mir-548	hsa-miR-548a-3p (hsa-miR-548a)	URS000038037E_9606	CAAAACUGGCAAUUACUUUUGC	[65]
	hsa-miR-548aa^##^ hsa-miR-548t-3p	URS000012930C_9606	AAAAACCACAAUUACUUUUGCACCA	[66]
	hsa-miR-548ap-5p	URS000054B69F_9606	AAAAGUAAUUGCGGUCUUU	[73]
	hsa-miR-548b-3p (hsa-miR-548b)	URS000039A25B_9606	CAAGAACCUCAGUUGCUUUUGU	[65]
	hsa-miR-548c-3p (hsa-miR-548c)	URS0000614A9B_9606	CAAAAAUCUCAAUUACUUUUGC	[65]
	hsa-miR-548d-5p	URS00005F2D64_9606	AAAAGUAAUUGUGGUUUUUGCC	[65]
	hsa-miR-570-3p (hsa-miR-570)	URS0000250A40_9606	CGAAAACAGCAAUUACCUUUGC	[65]
	hsa-miR-603	URS000075A6F1_9606	CACACACUGCAAUUACUUUUGC	[65]
mir-549	hsa-miR-549a-3p (hsa-miR-549)	URS00004C689A_9606	UGACAACUAUGGAUGAGCUCU	[65]
mir-550	hsa-miR-550a-5p (hsa-miR-550)	URS00003FFA6C_9606	AGUGCCUGAGGGAGUAAGAGCCC	[65]
mir-551	hsa-miR-551a	URS00002E99CB_9606	GCGACCCACUCUUGGUUUCCA	[65]
mir-556	hsa-miR-556-3p	URS00001D6605_9606	AUAUUACCAUUAGCUCAUCUUU	[65]
mir-561	hsa-miR-561–3p (hsa-miR-561)	URS000075D1DD_9606	CAAAGUUUAAGAUCCUUGAAGU	[65]
mir-564	hsa-miR-564	URS000075ED17_9606	AGGCACGGUGUCAGCAGGC	[65]
mir-571	hsa-miR-571	URS000075C61C_9606	UGAGUUGGCCAUCUGAGUGAG	[65]
mir-572	hsa-miR-572	URS000075CEB8_9606	GUCCGCUCGGCGGUGGCCCA	[65]
mir-574	hsa-miR-574-3p	URS00001CF056_9606	CACGCUCAUGCACACACCCACA	[65, 66, 72]
	hsa-miR-574-5p	URS000057466C_9606	UGAGUGUGUGUGUGUGAGUGUGU	[66, 72]
mir-582	hsa-miR-582-3p	URS00002573C3_9606	UAACUGGUUGAACAACUGAACC	[65]
mir-584	hsa-miR-584-5p (hsa-miR-584)	URS0000576F83_9606	UUAUGGUUUGCCUGGGACUGAG	[65]
mir-589	hsa-miR-589-5p (hsa-miR-589)	URS00004214BB_9606	UGAGAACCACGUCUGCUCUGAG	[65]
mir-590	hsa-miR-590-3P	URS0000272039_9606	UAAUUUUAUGUAUAAGCUAGU	[65]
	hsa-miR-590-5p	URS00005CACA0_9606	GAGCUUAUUCAUAAAAGUGCAG	[65, 70]
mir-592	hsa-miR-592	URS00004F507C_9606	UUGUGUCAAUAUGCGAUGAUGU	[65]
mir-593	hsa-miR-593-3p (hsa-miR-593)	URS000075D407_9606	UGUCUCUGCUGGGGUUUCU	[65]
mir-595	hsa-miR-595	URS000075B75E_9606	GAAGUGUGCCGUGGUGUGUCU	[65]
mir-596	hsa-miR-596	URS000075B35F_9606	AAGCCUGCCCGGCUCCUCGGG	[65]
mir-6089	hsa-miR-6089	URS000075B63F_9606	GGAGGCCGGGGUGGGGCGGGGCGG	[12]
mir-615	hsa-miR-615-3p	URS00003D5391_9606	UCCGAGCCUGGGUCUCCCUCUU	[53, 54]
	hsa-miR-615-5p	URS00004D8280_9606	GGGGGUCCCCGGUGCUCGGAUC	[65, 67]
mir-616	hsa-miR-616-3p (hsa-miR-616)	URS00005E3F32_9606	AGUCAUUGGAGGGUUUGAGCAG	[65]
mir-618	hsa-miR-618	URS0000450F92_9606	AAACUCUACUUGUCCUUCUGAGU	[65]
mir-619	hsa-miR-619-5p	URS000075B584_9606	GCUGGGAUUACAGGCAUGAGCC	[54, 66]
mir-622	hsa-miR-622	URS000075E944_9606	ACAGUCUGCUGAGGUUGGAGC	[65]
mir-623	hsa-miR-623	URS000075DCB1_9606	AUCCCUUGCAGGGGCUGUUGGGU	[65]
mir-625	hsa-miR-625-3p (hsa-miR-625*)	URS0000475E09_9606	GACUAUAGAACUUUCCCCCUCA	[65]
mir-628	hsa-miR-628-3p	URS000061BE3B_9606	UCUAGUAAGAGUGGCAGUCGA	[65]
mir-629	hsa-miR-629-5p (hsa-miR-629)	URS00002F3336_9606	UGGGUUUACGUUGGGAGAACU	[65]
mir-636	hsa-miR-636	URS000075A79D_9606	UGUGCUUGCUCGUCCCGCCCGCA	[65]
mir-638	hsa-miR-638	URS000075DB2F_9606	AGGGAUCGCGGGCGGGUGGCGGCCU	[12, 65, 70, 72]
mir-639	hsa-miR-639	URS000075B8B8_9606	AUCGCUGCGGUUGCGAGCGCUGU	[65]
mir-641	hsa-miR-641	URS000039D790_9606	AAAGACAUAGGAUAGAGUCACCUC	[65]
mir-642	hsa-miR-642a-5p (hsa-miR-642)	URS00000F2C33_9606	GUCCCUCUCCAAAUGUGUCUUG	[65]
	hsa-miR-642b-5p	URS000075B1CE_9606	GGUUCCCUCUCCAAAUGUGUCU	[73]
mir-649	hsa-miR-649	URS000075DD5B_9606	AAACCUGUGUUGUUCAAGAGUC	[65]
mir-650	hsa-miR-650	URS000075A00C_9606	AGGAGGCAGCGCUCUCAGGAC	[65]
mir-6511	hsa-miR-6511a-5p	URS000075C82B_9606	CAGGCAGAAGUGGGGCUGACAGG	[67]
	hsa-miR-6511b-3p	URS0000759CCE_9606	CCUCACCACCCCUUCUGCCUGCA	[67]
mir-652	hsa-miR-652-3p (hsa-miR-652)	URS0000013DD8_9606	AAUGGCGCCACUAGGGUUGUG	[64]
mir-654	hsa-miR-654-3p	URS00002F40E9_9606	UAUGUCUGCUGACCAUCACCUU	[65]
	hsa-miR-654-5p (hsa-miR-654)	URS00002B0B46_9606	UGGUGGGCCGCAGAACAUGUGC	[65]
mir-657	hsa-miR-657	URS000075C4C7_9606	GGCAGGUUCUCACCCUCUCUAGG	[65]
mir-661	hsa-miR-661	URS000075A4E8_9606	UGCCUGGGUCUCUGGCCUGCGCGU	[65]
mir-663	hsa-miR-663a	URS00004929F1_9606	AGGCGGGGCGCCGCGGGACCGC	[54, 66, 67]
	hsa-miR-663b	URS000075C3F6_9606	GGUGGCCCGGCCGUGCCUGAGG	[54, 65, 67]
mir-664	hsa-miR-664a-3p (hsa-miR-664) [65]	URS000029AE45_9606	UAUUCAUUUAUCCCCAGCCUACA	[65, 66]
mir-665	hsa-miR-665	URS0000355E82_9606	ACCAGGAGGCUGAGGCCCCU	[65, 67]
mir-671	hsa-miR-671–3p	URS00002B7450_9606	UCCGGUUCUCAGGGCUCCACC	[65, 67]
	hsa-miR-671-5p	URS00002FB368_9606	AGGAAGCCCUGGAGGGGCUGGAG	[67]
mir-6724	hsa-miR-6724-5p	URS00007777B8_9606	CUGGGCCCGCGGCGGGCGUGGGG	[67]
mir-675	hsa-miR-675-5p	URS00004E5112_9606	UGGUGCGGAGAGGGCCCACAGUG	[67]
mir-7	hsa-miR-7-2-3p (hsa-miR-7-2*)	URS0000572E11_9606	CAACAAAUCCCAGUCUACCUAA	[65]
mir-708	hsa-miR-708-5p (hsa-miR-708)	URS000019D79B_9606	AAGGAGCUUACAAUCUAGCUGGG	[65]
mir-743	hsa-miR-888-5p (hsa-miR-888)	URS000075D73F_9606	UACUCAAAAAGCUGUCAGUCA	[65]
	hsa-miR-892b	URS000075A42A_9606	CACUGGCUCCUUUCUGGGUAGA	[65]
mir-744	hsa-miR-744-3p (hsa-miR-744*)	URS00005FAA14_9606	CUGUUGCCACUAACCUCAACCU	[65]
	hsa-miR-744-5p (hsa-miR-744)	URS00002ED61F_9606	UGCGGGGCUAGGGCUAACAGCA	[65]
mir-760	hsa-miR-760	URS0000512C88_9606	CGGCUCUGGGUCUGUGGGGA	[67]
mir-762	hsa-miR-762	URS0000327AFF_9606	GGGGCUGGGGCCGGGGCCGAGC	[72]
mir-7641	hsa-miR-7641	URS000075B793_9606	UUGAUCUCGGAAGCUAAGC	[54, 66]
mir-766	hsa-miR-766-3p (hsa-miR-766)	URS00001012BC_9606	ACUCCAGCCCCACAGCCUCAGC	[65]
mir-769	hsa-miR-769-5p	URS00004E008F_9606	UGAGACCUCUGGGUUCUGAGCU	[54, 65]
mir-770	hsa-miR-770-5p	URS000075A169_9606	UCCAGUACCACGUGUCAGGGCCA	[65]
mir-8	hsa-miR-141–3p (hsa-miR-141)	URS000003E1A9_9606	UAACACUGUCUGGUAAAGAUGG	[65]
	hsa-miR-200a-3p (hsa-miR-200a)	URS000008DA94_9606	UAACACUGUCUGGUAACGAUGU	[65]
	hsa-miR-200a-5p (hsa-miR-200a*)	URS000023B77E_9606	CAUCUUACCGGACAGUGCUGGA	[65]
	hsa-miR-200b-3p (hsa-miR-200b)	URS000014D9C1_9606	UAAUACUGCCUGGUAAUGAUGA	[65]
	hsa-miR-200c-3p (hsa-miR-200c)	URS0000192F9C_9606	UAAUACUGCCGGGUAAUGAUGGA	[64, 65]
	hsa-miR-429	URS000055BBE5_9606	UAAUACUGUCUGGUAAAACCGU	[65]
mir-8069	hsa-miR-8069	URS000075E1C1_9606	GGAUGGUUGGGGGCGGUCGGCGU	[12]
mir-874	hsa-miR-874-3p	URS00005609ED_9606	CUGCCCUGGCCCGAGGGACCGA	[67]
mir-875	hsa-miR-875-5p	URS0000312ECD_9606	UAUACCUCAGUUUUAUCAGGUG	[65]
mir-876	hsa-miR-876-5p	URS0000470305_9606	UGGAUUUCUUUGUGAAUCACCA	[65]
mir-885	hsa-miR-885-5p	URS0000246356_9606	UCCAUUACACUACCCUGCCUCU	[65]
mir-9	hsa-miR-9-3p (hsa-miR-9*)	URS00003496BE_9606	AUAAAGCUAGAUAACCGAAAGU	[65]
	hsa-miR-9-5p (hsa-miR-9) [65]	URS00004208C5_9606	UCUUUGGUUAUCUAGCUGUAUGA	[54, 65]
miR-922	hsa-miR-922	URS000075D35F_9606	GCAGCAGAGAAUAGGACUACGUC	[65]
miR-935	hsa-miR-935	URS000033EBB8_9606	CCAGUUACCGCUUCCGCUACCGC	[65]
mir-937	hsa-miR-937-3p (hsa-miR-937)	URS0000553F51_9606	AUCCGCGCUCUGACUCUCUGCC	[65]
mir-938	hsa-miR-938	URS000075DF80_9606	UGCCCUUAAAGGUGAACCCAGU	[65]
mir-939	hsa-miR-939-5p (hsa-miR-939)	URS00005A31EB_9606	UGGGGAGCUGAGGCUCUGGGGGUG	[65]
mir-941	hsa-miR-941	URS000050E4BA_9606	CACCCGGCUGUGUGCACAUGUGC	[65]
mir-95	hsa-miR-545-3p (hsa-miR-545)	URS00002E1509_9606	UCAGCAAACAUUUAUUGUGUGC	[65]
	hsa-miR-545-5p (hsa-miR-545*)	URS00004C4520_9606	UCAGUAAAUGUUUAUUAGAUGA	[65]
	hsa-let-7c	–	–	[65]
	hsa-miR-1	–	–	[65]
	hsa-miR-10	URS00005D8C46_9606	UACCCUGUAGAACCGAAUUUG	[74]
	hsa-miR-10395-3p	URS0000D52042_9606	AUGUAUUCGUACUGUCUGAUG	[59]
	hsa-miR-10395-5p	URS0000D53F1E_9606	GUGAUGGAGAGCAAUACC	[59]
	hsa-miR-1180	–	–	[65]
	hsa-miR-1234-5p	–	–	[72]
	hsa-miR-1274a	–	–	[65]
	hsa-miR-1274b	–	–	[65]
	hsa-miR-1298	–	–	[65]
	hsa-miR-1300	–	–	[65]
	hsa-miR-133a	–	–	[65]
	hsa-miR-152	–	–	[65]
	hsa-miR-190b	–	–	[65]
	hsa-miR-199	URS000027FB26_9606	CCCAGUGUUUAGACUAUCUGU	[74]
	hsa-miR-210	–	–	[65]
	hsa-miR-215	–	–	[65]
	hsa-miR-219-2-3p	–	–	[65]
	hsa-miR-2277-5p	URS00000D6C3F_9606	AGCGCGGGCUGAGCGCUGCCAGUC	[67]
	hsa-miR-23-3p	–	–	[73]
	hsa-miR-26	–	–	[74]
	hsa-miR-3178	URS0000365675_9606	GGGGCGCGGCCGGAUCG	[12]
	hsa-miR-3195	URS000004DB7E_9606	CGCGCCGGGCCCGGGUU	[54]
	hsa-miR-3196	URS000033B548_9606	CGGGGCGGCAGGGGCCUC	[12]
	hsa-miR-328	–	–	[65]
	hsa-miR-329	–	–	[64, 65]
	hsa-miR-3614-5p	URS00003D4175_9606	CCACUUGGAUCUGAAGGCUGCCC	[54]
	hsa-miR-3653-3p	URS000009AF54_9606	CUAAGAAGUUGACUGAAG	[54]
	hsa-miR-3656	URS0000514CEC_9606	GGCGGGUGCGGGGGUGG	[12, 72]
	hsa-miR-3665	URS000075AFFF_9606	AGCAGGUGCGGGGCGGCG	[12]
	hsa-miR-370	–	–	[65]
	hsa-miR-375	–	–	[65]
	hsa-miR-378c	URS000025307A_9606	ACUGGACUUGGAGUCAGAAGAGUGG	[54]
	hsa-miR-383	–	–	[65]
	hsa-miR-3944-3p	URS0000446855_9606	UUCGGGCUGGCCUGCUGCUCCGG	[67]
	hsa-miR-410	–	–	[65]
	hsa-miR-412	–	–	[65]
	hsa-miR-4284	URS00001FC26E_9606	GGGCUCACAUCACCCCAU	[72]
	hsa-miR-433	–	–	[65]
	hsa-miR-4443	URS00004D84DB_9606	UUGGAGGCGUGGGUUUU	[72]
	hsa-miR-4448	URS00005F305A_9606	GGCUCCUUGGUCUAGGGGUA	[54]
	hsa-miR-4454	URS00005D12AC_9606	GGAUCCGAGUCACGGCACCA	[12, 54, 66]
	hsa-miR-4461	URS000028425A_9606	GAUUGAGACUAGUAGGGCUAGGC	[54]
	hsa-miR-4466	URS00001DC1D3_9606	GGGUGCGGGCCGGCGGGG	[12, 54, 72]
	hsa-miR-4485-3p (hsa-miR-4485)	URS000038446A_9606	UAACGGCCGCGGUACCCUAA	[11]
	hsa-miR-4492	URS000045ED38_9606	GGGGCUGGGCGCGCGCC	[54]
	hsa-miR-4497	URS00000A2C49_9606	CUCCGGGACGGCUGGGC	[12]
	hsa-miR-4505	URS000075EBEE_9606	AGGCUGGGCUGGGACGGA	[72]
	hsa-miR-4508	URS00004E78D3_9606	GCGGGGCUGGGCGCGCG	[12, 54]
	hsa-miR-4516	URS00000BF7F9_9606	GGGAGAAGGGUCGGGGC	[12, 54, 66]
	hsa-miR-4532	URS000013A349_9606	CCCCGGGGAGCCCGGCG	[54, 66, 67]
	hsa-miR-4649-5p	URS000044FB51_9606	UGGGCGAGGGGUGGGCUCUCAGAG	[67]
	hsa-miR-4665-5p	URS00000E9F44_9606	CUGGGGGACGCGUGAGCGCGAGC	[67]
	hsa-miR-4668-5p	URS00000A17E7_9606	AGGGAAAAAAAAAAGGAUUUGUC	[12]
	hsa-miR-4687-3p	URS000047456A_9606	UGGCUGUUGGAGGGGGCAGGC	[72]
	hsa-miR-4707-5p	URS00003EB443_9606	GCCCCGGCGCGGGCGGGUUCUGG	[67]
	hsa-miR-4708-3p	URS00004F4FFB_9606	AGCAAGGCGGCAUCUCUCUGAU	[73]
	hsa-miR-4722-5p	URS000047996E_9606	GGCAGGAGGGCUGUGCCAGGUUG	[67]
	hsa-miR-4741	URS0000547F6A_9606	CGGGCUGUCCGGAGGGGUCGGCU	[67]
	hsa-miR-4763-3p	URS00004A40D8_9606	AGGCAGGGGCUGGUGCUGGGCGGG	[67, 72]
	hsa-miR-4787-5p	URS0000521832_9606	GCGGGGGUGGCGGCGGCAUCCC	[12, 54, 72]
	hsa-miR-4792	URS00005B6542_9606	CGGUGAGCGCUCGCUGGC	[54, 66]
	hsa-miR-487a	–	–	[65]
	hsa-miR-487b	–	–	[65]
	hsa-miR-489	–	–	[65]
	hsa-miR-494	–	–	[65]
	hsa-miR-5088-5p	URS00002F0130_9606	CAGGGCUCAGGGAUUGGAUGGAGG	[67]
	hsa-miR-5095	URS00002E1785_9606	UUACAGGCGUGAACCACCGCG	[54]
	hsa-miR-5096	URS00001F8B82_9606	GUUUCACCAUGUUGGUCAGGC	[54, 66]
	hsa-miR-5100	URS0000079F78_9606	UUCAGAUCCCAGCGGUGCCUCU	[12]
	hsa-miR-5191	URS000075CB1C_9606	AGGAUAGGAAGAAUGAAGUGCU	[54]
	hsa-miR-520b	–	–	[65]
	hsa-miR-520f	–	–	[65]
	hsa-miR-520g	–	–	[65]
	hsa-miR-5585-3p	URS00003E6EFA_9606	CUGAAUAGCUGGGACUACAGGU	[54, 66]
	hsa-miR-566	URS00000FD5FE_9606	GGGCGCCUGUGAUCCCAAC	[65]
	hsa-miR-5787	URS000075CA3A_9606	GGGCUGGGGCGCGGGGAGGU	[12, 72]
	hsa-miR-597	–	–	[65]
	hsa-miR-598	–	–	[65]
	hsa-miR-605	–	–	[65]
	hsa-miR-6068	URS000075E142_9606	CCUGCGAGUCUCCGGCGGUGG	[72]
	hsa-miR-6087	URS000075EF8B_9606	UGAGGCGGGGGGGCGAGC	[12, 54, 66, 67]
	hsa-miR-6088	URS000075EC34_9606	AGAGAUGAAGCGGGGGGGCG	[12, 72]
	hsa-miR-6090	URS0000759F58_9606	GGGGAGCGAGGGGCGGGGC	[12]
	hsa-miR-6124	URS000075CC26_9606	GGGAAAAGGAAGGGGGAGGA	[72]
	hsa-miR-6125	URS000075F0F0_9606	GCGGAAGGCGGAGCGGCGGA	[12]
	hsa-miR-6126	URS000075D118_9606	GUGAAGGCCCGGCGGAGA	[66]
	hsa-miR-627	–	–	[65]
	hsa-miR-655	–	–	[65]
	hsa-miR-656	–	–	[65]
	hsa-miR-659-3p (hsa-miR-659)	URS000075C04A_9606	CUUGGUUCAGGGAGGGUCCCCA	[65]
	hsa-miR-668	–	–	[65]
	hsa-miR-672	–	–	[65]
	hsa-miR-6727-5p	URS000075A9AA_9606	CUCGGGGCAGGCGGCUGGGAGCG	[12, 67]
	hsa-miR-6729-5p	URS000075DD20_9606	UGGGCGAGGGCGGCUGAGCGGC	[12, 67]
	hsa-miR-6739-5p	URS000075C51C_9606	UGGGAAAGAGAAAGAACAAGUA	[66]
	hsa-miR-6746-5p	URS000075AF8F_9606	CCGGGAGAAGGAGGUGGCCUGG	[67]
	hsa-miR-6789-5p	URS000075DD04_9606	GUAGGGGCGUCCCGGGCGCGCGGG	[67]
	hsa-miR-6821-5p	URS000075EAF3_9606	GUGCGUGGUGGCUCGAGGCGGGG	[67]
	hsa-miR-6858-5p	URS000075C360_9606	GUGAGGAGGGGCUGGCAGGGAC	[67]
	hsa-miR-6869-5p	URS000075C3FC_9606	GUGAGUAGUGGCGCGCGGCGGC	[12]
	hsa-miR-6891-5p	URS000075BD73_9606	UAAGGAGGGGGAUGAGGGG	[67]
	hsa-miR-720	–	–	[65]
	hsa-miR-7704	URS000028F729_9606	CGGGGUCGGCGGCGACGUG	[12, 54, 66]
	hsa-miR-7977	URS000075A1F7_9606	UUCCCAGCCAACGCACCA	[12]
	hsa-miR-8061	URS000075E23B_9606	CUUAGAUUAGAGGAUAUUGUU	[54]
	hsa-miR-8485	URS000076B539_9606	CACACACACACACACACGUAU	[66]
	hsa-miR-874	–	–	[65]
	hsa-miR-886-3p	–	–	[65]
	hsa-miR-886-5p	–	–	[65]
	hsa-miR-887	–	–	[65]
	hsa-miR-889	–	–	[65]
	hsa-miR-891a	–	–	[65]
	hsa-miR-942	–	–	[65]
	hsa-miR-95	–	–	[65]

## The two names corresponded to the same sequence

# Identified by the sequence and the precursor. The referred article uses a name not found in the databases

In this review, we present a comprehensive analysis of miRNAs currently identified in human AT-MSC-EVs. 489 miRNAs from 255 gene families were classified. The mir-515 and mir-10 families have the greatest numbers of miRNAs (Table 2). However, there was no information available about which gene families the other 115 miRNAs belonged to. In addition, hsa-miR-320a-3p and hsa-miR-375-3p were identified by the sequence and the precursor reported by Reza et al. [54], since the actual names used in the reference, hsa-miR-320a and hsa-miR-375, respectively, were not found for mature miRNA in any of the databases. Hsa-miR-1273a [54, 66] was included in the miRBase database as a dead miRNA entry. It was eventually removed due to lack of consistency between the patterns of mapped reads from RNA-sequencing experiments and the gene being processed as a miRNA. hsa-miR-1274a, hsa-miR-1274b, hsa-miR-1300 and hsa-miR-720 [65] were also included in the miRBase database as dead miRNA entries. They were removed because it is likely that they are fragments of tRNAs and mRNA. This could be the reason for their absence from the RNAcentral database. 44 miRNAs were not found in any of the databases (Table 2). Other special cases included hsa-miR-548aa and hsa-miR-548 t-3p [66] – there is a specific entry for each one in the miRBase database, however, both entries showed the same sequence and RNAcentral link. Therefore, in the present review they are treated as the same miRNA. The same applies to hsa-miR-199b-3p and hsa-miR-199a-3p [53, 65, 66, 72].

The variety of miRNAs present in AT-MSC-EVs may play a role in the different therapeutic effects based on the paracrine properties of MSC [13]. Regardless, to confirm the involvement of miRNAs in these effects, it is necessary to take into consideration not only the presence of a specific miRNA, but also other factors such as concentration, structure, and availability of accessory proteins [13].

**Fig. 5:**
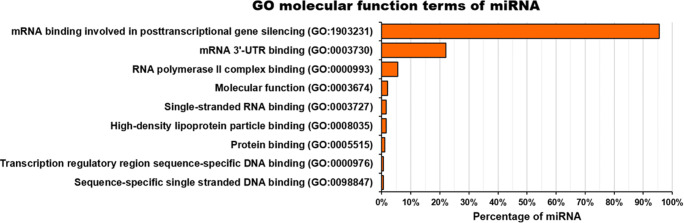
Gene ontology (GO) molecular function terms of the miRNA detected in human AT-MSC-EVs. Only 199 miRNAs showed GO molecular function annotations. The 95% of them enables the mRNA binding involved in post-transcriptional gene silencing

**Fig. 6:**
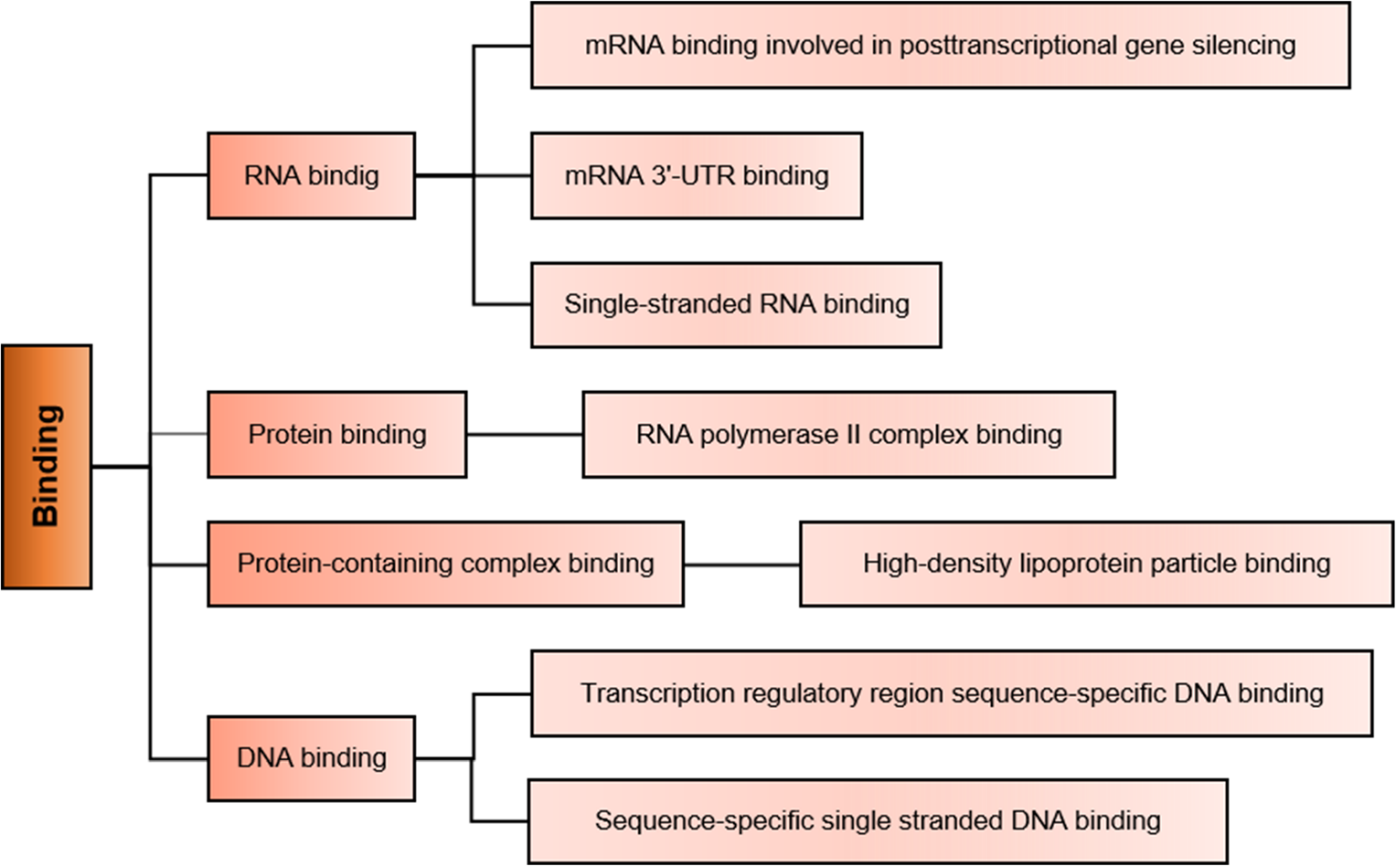
Simplified outline of the molecular functions enables by the miRNA detected in human AT-MSC-EVs. For a complete review of the relationships between gene ontology terms see the chart view in the web-based tool QuickGO (https://www.ebi.ac.uk/QuickGO/)

Only 199 miRNA showed GO annotations for molecular function when using the QuickGO database [55]. The molecular functions enabled by these miRNAs are mRNA binding involved in post-transcriptional gene silencing (95%), mRNA 3’-UTR binding (22%), RNA polymerase II complex binding (6%), single-stranded RNA binding and high-density lipoprotein particle binding (2% each), protein binding, transcription regulatory region sequence-specific DNA binding and sequence-specific single stranded DNA binding (1% each) (Fig. 5). All of these functions are specific child terms of the binding function (Fig. 6) which is also the most relevant molecular function of AT-MSC-EV proteins, as previously described. The specific molecular functions enabled by each miRNA are detailed in Table 3S.

**Fig. 7:**
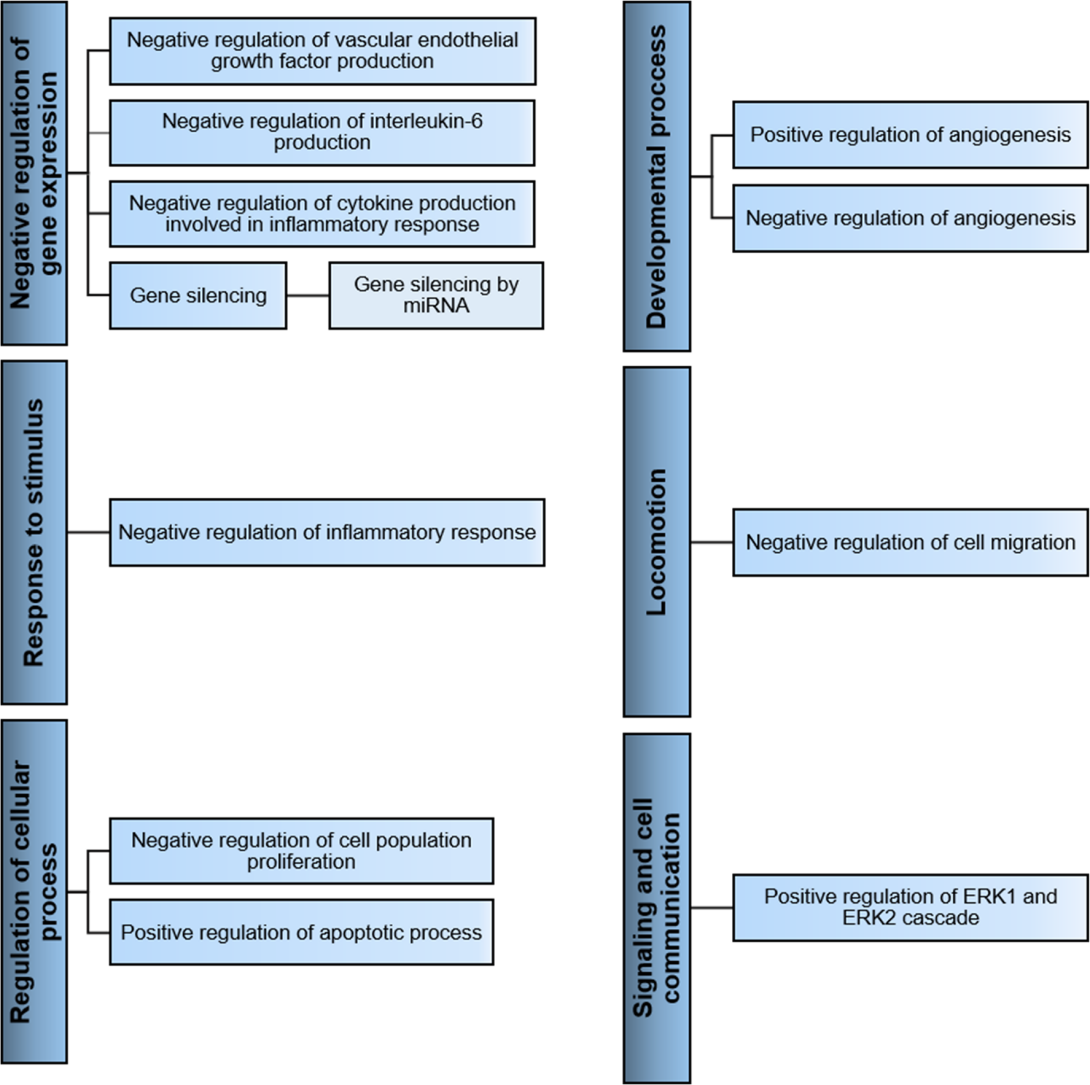
Simplified outline of the main biological processes in which the miRNA detected in EVs derived from human AT-MSC are involved. For a complete review of the relationships between gene ontology terms see the chart view in the web-based tool QuickGO (https://www.ebi.ac.uk/QuickGO/)

**Fig. 8:**
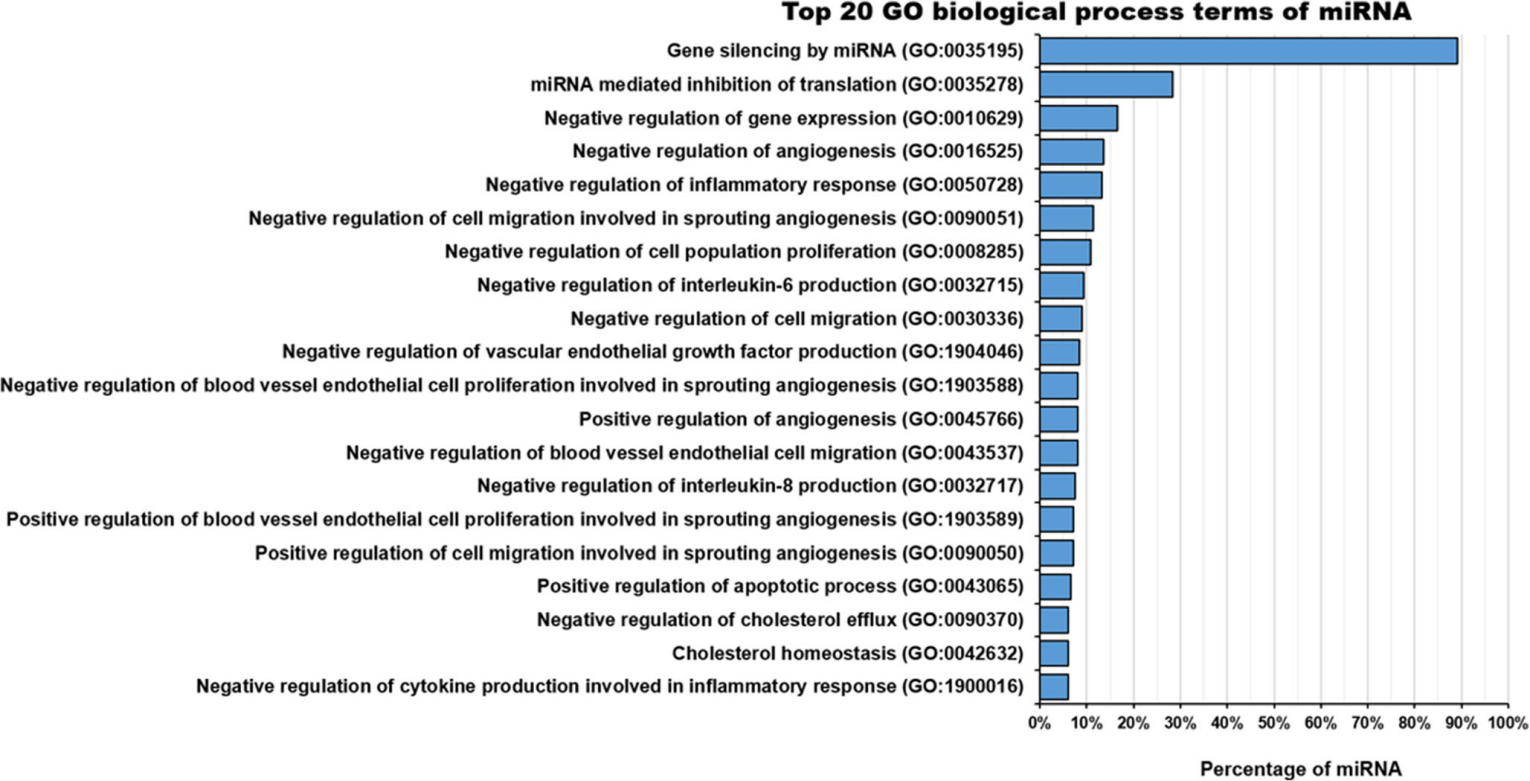
The top 20 gene ontology (GO) biological process terms of the 212 miRNA detected in human AT-MSC-EVs which presented annotations in this aspect. The 89% of them are involved in gene silencing

The number of miRNAs with GO annotations of biological processes in QuickGO [55] was 212. These miRNAs take part in biological processes described by 577 different GO terms. The biological processes in which the greatest number of miRNA are involved are: negative regulation of gene expression, response to stimulus, regulation of cellular process, developmental process, locomotion, signaling, and cell communication (Fig. 7). The specific miRNAs involved in each process are detailed in Table 4S. 89% of these miRNAs are involved in gene silencing (Fig. 8). Other relevant GO terms in which a large number of miRNAs are included are miRNA mediated inhibition of translation (28%) negative regulation of gene expression (17%), negative regulation of angiogenesis (14%), negative regulation of inflammatory response (13%) and negative regulation of cell migration involved in sprouting angiogenesis (11%) (Fig. 8).

### Therapeutic approaches of AT-MSC-EV miRNAs

Based on the data, miRNAs present in AT-MSC-EV cargo support their potential use as new treatments in various research fields. Similar to proteins, different miRNAs are involved in inflammatory response (hsa-let-7 g-5p, hsa-miR-16-5p, hsa-miR-92a-3p), negative regulation of macrophage activation (hsa-miR-124-3p), regulation of MAPK cascade (hsa-miR-126-3p, hsa-miR-21-5p, hsa-miR-26a-5p, hsa-miR-29b-3p), regulation of phosphatidylinositol 3-kinase signaling (hsa-miR-126-3p, hsa-miR-20a-5p, hsa-miR-21-5p), and positive regulation of cell migration (hsa-miR-1290, hsa-miR-181b-5p, hsa-miR-21-5p, hsa-miR-29b-3p) (Table 4S). Therefore, they can also be implicated in the positive effects observed after the injection of human AT-MSC-EVs in animal model of osteoarthritis [66], and in osteoarthritis chondrocytes [66] and osteoblasts [78] in vitro.

Regarding the use of AT-MSC-EVs for cardiology and vascular diseases, the rationale may be the role of the detected miRNAs in negative regulation of heart rate (hsa-miR-26a-5p), regulation of heart contraction (hsa-miR-92a-3p), positive regulation of cardiac muscle cell proliferation (hsa-miR-199b-3p, hsa-miR-19b-3p, hsa-miR-204-5p, hsa-miR-222-3p, hsa-miR-23b-3p), negative regulation of cardiac muscle cell apoptotic process (hsa-miR-145-5p, hsa-miR-199b-3p, hsa-miR-19b-3p, hsa-miR-21-5p, hsa-miR-30e-5p), regulation of cardiac muscle hypertrophy (hsa-miR-20a-5p), cell differentiation (hsa-miR-155-5p) and proliferation (hsa-miR-199a-5p), and regulation of cardiac conduction (hsa-miR-19a-3p), among others (Table 4S). AT-MSC-EV proteins are also involved in some of these biological processes. Therefore, both types of molecules, proteins and miRNAS, may present a synergistic action, supporting the cardioprotection observed in an in vivo model of myocardial infarction after the administration of AT-MSC-EVs [79].

Numerous miRNAs are involved in the positive regulation of angiogenesis, such as hsa-miR-126-3p, hsa-miR-143-3p, hsa-miR-1908-5p, hsa-miR-199a-5p, hsa-miR-199b-3p, hsa-miR-20a-5p, hsa-miR-21-5p, hsa-miR-27b-3p, hsa-miR-29a-3p and hsa-miR-31-5p, among others (Table 4S). They may play a role in the promotion of angiogenesis, as observed both in vitro and in vivo [60, 72, 80]. However, it should be noted that there are also numerous miRNAs involved in the negative regulation of angiogenesis (see Table 4S for a complete list).

Finally, although there are less miRNAs than proteins involved in regulation of cellular processes such as proliferation and apoptosis (Tables 2S and 4S), it should be noted that each miRNA targets more than one mRNA. Therefore, each one can show effects on numerous proteins.

### tRNA, mRNA, rRNA, snRNA, snoRNA and scRNA

According to Kaur et al. [53], the detected tRNA in AT-MSC-EVs represents 47% of all small RNAs observed. Although this percentage is slightly higher than that of miRNA, the available information about the presence of this type of RNA [11, 53, 54] is significantly less. The main tRNAs, in order of quantity detected in AT-MSC-EVs, are tRNA GCC (Gly), tRNA CTC (Glu) and tRNA TTC (Glu). Surprisingly, in AT-MSC the tRNA CTC (Glu) is the most abundant, while tRNA GCC (Gly) makes up a significantly lower percentage than in AT-MSC-EVs [11]. Other tRNAs present in lesser amounts in AT-MSC-EVs are tRNA GTC (Asp), tRNA CCC (Gly), tRNA GTG (His), tRNA CTT (Lys), tRNA AAC (Val) and tRNA CAC (Val) [11].

84 different mRNAs were detected in the AT-MSC-EVs. Their corresponding gene symbols, in order of quantity detected, are FN1, COL4A3, PGF, MMP2, PLG, HGF, IGF1, TEK, FGF2, HIF1A, VEGFA, EDN1, PF4, CXCL9, FGF1, TGFB2, ITGAV, PROK2, EGF, FLT1, IL8, IFNG, IFNA1, SERPINE1, FIGF, TIMP3, JAG1, CXCL10 ANGPT1, TIMP2, IL6, TIMP1, SERPINF1, AKT1, ANPEP, EFNB2, CXCL6, HPSE, THBS1, EPHB4, NRP1, THBS2, CCL11, TGFA, TIE1, TGFB1, COL18A1, PDGFA, KDR, F3, TGFBR1, BAI1, NRP2, ANGPT2, MMP9, CXCL1 ANGPTL4, ANG, ENG, PTGS1, CCL2, VEGFC, EFNA1, TNF, CTGF, NOS3, VEGFB, CXCL5, LECT1, CDH5, LEP, ITGB3, MMP14, IL1B, SPHK1, PLAU, FGFR3, ID1, S1PR1, ERBB2, PECAM1, NOTCH4, TYMP and MDK [52].

Other types of small RNA, such as rRNA [54], snRNA, snoRNA [53, 54] and scRNA [53], are present in AT-MSC-EVs, but the available information about these is even less than that of tRNA.

### Lipids

The third type of molecule transported by EVs is lipids [3, 4]. The lipid composition of EVs has been less studied than that of proteins or miRNAs [8]. Thus, the number of lipid entries (639) in the Vesiclepedia database [41] is notably lower than the number of protein and miRNA entries (349,988 and 10,520, respectively). None of these lipid entries are related to AT-MSC-EVs or any other MSC-EVs. The total lipid content of AT-MSC-EVs has been analysed by Bari et al. [58], using the Nile Red assay. However, to our knowledge, there is no detailed information about the different types of lipids present in AT-MSC-EVs.

## Modification of Cargo Components to Improve their Potential Effects

Different cell culture conditions and pre-treatments have been used to modify the profile of human AT-MSC-EV cargo, with the aim to improve its effects in skin flap survival [59, 86], angiogenesis [60, 61, 64, 80], immune response [71, 87], bone regeneration [77] and cancer [118, 119]. To this purpose, human AT-MSCs have been exposed to oxidative stress [59, 86], hypoxic [61, 80] or inflammatory culture conditions [71, 87], stimulation with platelet-derived growth factor (PDGF) [60, 65] and basic fibroblast growth factor (bFGF) [64] and transfected with lentiviral particles with different miRNAs [77, 118, 119].

Under oxidative stress conditions (50 μM H_2_O_2_), AT-MSC-EVs showed an enhanced effect on skin flap survival after ischemic injury in in vivo models [59, 86]. This improvement was associated with a promotion of angiogenesis, reduction of inflammation and apoptosis [86]. The proteomic analysis of these EVs showed an increase (>2-fold) of histone H4, beta ig-h3, ITI-HC2, FLG-2, periostin, thrombospondin-1, pentraxin-related protein PTX3 and annexin A5; and a decrease (>2-fold) of plakophilin-1, VDB, Apo B-100, lactotransferrin, serotransferrin, alpha-fetoprotein, fatty acid-binding protein 5, dermcidin, and hornerin [59]. The RNA sequencing analysis showed that hsa-miR-10,395-5p and hsa-miR-10,395-3p were increased in H_2_O_2_ AT-MSC-EVs, while hsa-miR-24-3p, hsa-miR-16-5p, hsa-miR-93-5p, hsa-miR-31-5p, hsa-miR-23a-3p, hsa-miR-152-3p, hsa-miR-122-5p, hsa-miR-134-5p, hsa-miR-221–3p, hsa-miR-196a-5p, hsa-miR-23b-3p, hsa-miR-222-3p were decreased [59]. Finally, the peak size of EV from H_2_O_2_-stimulated AT-MSC was larger than that of unstimulated cells [59].

Hypoxic culture conditions also induce the release of larger EVs according to Han et al. [61], although other authors claim that there are no significant differences in size [80]. The EVs collected from AT-MSC cultured under hypoxic conditions (5% O_2_) seemed to enhance angiogenic properties in cultured human umbilical vein endothelial cells and in an in vivo model of fat grafting [61, 80]. The results of these studies showed that the amount of the surface marker CD44 was significantly lower in hypoxic EVs [80], while VEGF-A, EGF, FGF-4, VEGFR-2, VEGFR-3, C-C motif chemokine 8 and 13 were increased under these culture conditions [61].

EVs contents are also different after AT-MSC exposure to inflammatory cytokines. In EVs secreted by INF-γ-stimulated AT-MSC, indoleamine 2,3-dioxygenase mRNA was detected, although its presence did not significantly improve their potential to control activated T cell proliferation, in comparison with those derived from unstimulated AT-MSC [87]. However, when AT-MSCs were pretreated with both INF-γ and TNF-α, the enriched EVs induced the polarization of macrophages to the M2 phenotype [71]. Under this pro-inflammatory culture condition, AT-MSC-EVs cause differences in the expression of 81 different miRNAs [71] (Table 3).

**Table 3:** miRNA detected in EVs derived from human AT-MSC treated with IFN-γ and TNFα, PDGF and bFGF (Modified tables from Domenis et al., 2018 [71], Lopatina et al., 2014 and 2018, [64, 65])

Stimulation with IFN-γ and TNFα				
miRNA under-expressed				
has-let-7b-5p	hsa-let-7c-5p	let-7f-5p	has-let-7i-5p	hsa-miR-10a-5p
hsa-miR-10b-5p	hsa-miR-125a-5p	hsa-miR-143-3p	hsa-miR-146b-5p	hsa-miR-148a-5p
hsa-miR-16-5p	hsa-miR-191-5p	hsa-miR-21-5p	hsa-miR-22-3p	hsa-miR-221–3p
hsa-miR-27a-3p	hsa-miR-28-3p	hsa-miR-381–3p	hsa-miR-423-5p	hsa-miR-486-5p
hsa-miR-92a-3p	hsa-miR-941	hsa-miR-99b-5p		
miRNA over-expressed				
hsa-let-7a-5p	hsa-let-7 g-5p	hsa-miR-100-5p	hsa-miR-125b-1-3p	hsa-miR-126a-5p
hsa-miR-146a-5p	hsa-miR-148a-3p	hsa-miR-151a-3p	hsa-miR-181a-5p	hsa-miR-192-5p
hsa-miR-199a-5p	hsa-miR-21–3p	hsa-miR-25-3p	hsa-miR-26a-5p	hsa-miR-30e-3p
hsa-miR-320a-3p	hsa-miR-340-5p	hsa-miR-378a-3p	hsa-miR-410-3p	hsa-miR-423-3p
hsa-miR-889-3p	hsa-miR-92b-3p	hsa-miR-99a-5p		
Lost miRNA				
hsa-let-7e-5p	hsa-miR-125b-5p	hsa-miR-134-5p	hsa-miR-136-3p	hsa-miR-148b-3p
hsa-miR-150-5p	hsa-miR-151a-5p	hsa-miR-181b-5p	hsa-miR-186-5p	hsa-miR-1910-5p
hsa-miR-193b-3p	hsa-miR-197-3p	hsa-miR-19b-3p	hsa-miR-19b-3p	hsa-miR-23b-3p
hsa-miR-27b-3p	hsa-miR-301a-3p	hsa-miR-30a-3p	hsa-miR-335-3p	hsa-miR-382-5p
hsa-miR-409-3p	hsa-miR-4677-3p	hsa-miR-532-5p	hsa-miR-6515-5p	hsa-miR-654-5p
hsa-miR-671–3p	hsa-miR-7706	hsa-miR-98-5p		
Gained miRNA				
hsa-miR-100-3p	hsa-miR-101–3p	hsa-miR-1246	hsa-miR-127-3p	
hsa-miR-155-5p	hsa-miR-361-5p	hsa-miR-411-5p	hsa-miR-493-3p	
Stimulation with PDGF				
miRNA under-expressed				
hsa-miR-1225-3p	hsa-miR-1226-5p			
miRNA over-expressed				
hsa-miR-125b	hsa-miR-195	hsa-miR-203a-3p	hsa-miR-99a-3p	
miRNA-expressed only in stimulated				
hsa-let-7e	hsa-let-7f-2	hsa-miR-122	hsa-miR-1269	hsa-miR-1276
hsa-miR-129	hsa-miR-1296	hsa-miR-133b	hsa-miR-147b	hsa-miR-154
hsa-miR-186	hsa-miR-202	hsa-miR-208b	hsa-miR-211	hsa-miR-216b
hsa-miR-221	hsa-miR-23b	hsa-miR-296-3p	hsa-miR-338-5P	hsa-miR-34b
hsa-miR-373	hsa-miR-380-3p	hsa-miR-381	hsa-miR-432	hsa-miR-502
hsa-miR-511	hsa-miR-518a-5p	hsa-miR-525-3p	hsa-miR-548c-5p	hsa-miR-548I
hsa-miR-550	hsa-miR-551b	hsa-miR-562	hsa-miR-567	hsa-miR-575
hsa-miR-579	hsa-miR-581	hsa-miR-582-5p	hsa-miR-604	hsa-miR-614
hsa-miR-621	hsa-miR-643	hsa-miR-708-3p	hsa-miR-765	hsa-miR-802
hsa-miR-872	hsa-miR-92a-1	hsa-miR-943	hsa-miR-944	
Stimulation with bFGF				
miRNA under-expressed				
hsa-let-7a	hsa-let-7b	hsa-let-7d	hsa-let-7e	hsa-let-7 g
hsa-miR-100	hsa-miR-101	hsa-miR-103	hsa-miR-106a	hsa-miR-10a
hsa-miR-10b	hsa-miR-125a-3p	hsa-miR-125b	hsa-miR-127	hsa-miR-130a
hsa-miR-138	hsa-miR-143	hsa-miR-15b	hsa-miR-17	hsa-miR-181a
hsa-miR-185	hsa-miR-192	hsa-miR-193a-5p	hsa-miR-194	hsa-miR-195
hsa-miR-199a	hsa-miR-199a-3p	hsa-miR-20a	hsa-miR-20b	hsa-miR-21
hsa-miR-210	hsa-miR-22	hsa-miR-221	hsa-miR-224	hsa-miR-26a
hsa-miR-27b				
miRNA-expressed only in stimulated				
hsa-let-7c	hsa-miR-130b	hsa-miR-133a	hsa-miR-184	hsa-miR-193a-3p
hsa-miR-199b	hsa-miR-223	hsa-miR-26b	hsa-miR-27a	hsa-miR-28-3p
hsa-miR-340	hsa-miR-381	hsa-miR-483-5p	hsa-miR-539	hsa-miR-542-5p
hsa-miR-545	hsa-miR-579	hsa-miR-654-3p	hsa-miR-885-5p	

Other methods used to alter the expression of cargo components are stimulation with PDGF [60, 65], with bFGF [64], and lentiviral transfection with the miRNA of interest [77, 118, 119]. In the former case, PDGF stimulation increased release of smaller AT-MSC-EVs, and improved their angiogenic potential, both in cultured human microvascular endothelial cells and in an in vivo model of severe combined immunodeficiency [60]. This stimulation also improved the AT-MSC-EVs anti-inflammatory and immunomodulatory potential both in vitro and in vivo in peripheral blood mononuclear cell and in a murine model of hindlimb ischemia, respectively [65]. Regarding protein composition, these EVs contained several proteins not observed in unstimulated AT-MSC-EVs: C-C motif chemokine 21, IL-17RD, IL-20RA, inhibin A, tyrosine-protein kinase Lck, LIF, SL-2, SL-3, MMP-14, OSM, kit ligand, IL-6RB (soluble form), TGF-beta 5 (not found in UniProtKB), thrombopoietin, metalloproteinase inhibitor 1, and TNF receptor superfamily member 10D [60]. In addition, 65 proteins were up-regulated and 15 proteins were down-regulated (Table 4). The miRNA composition of stimulated AT-MSC-EVs also showed variations in the expression of 55 different miRNAs [65] (Table 3).

**Table 4:** Protein detected in EVs derived from human AT-MSC treated with PDGF (Modified table from Lopatina et al., 2018, [65])

Stimulation with PDGF			
Proteins up-regulated			
Adenomatous polyposis coli protein*	ADP-ribosyl cyclase/cyclic ADP-ribose hydrolase 1*	Brain-derived neurotrophic factor*	Cadherin-1*
Calsyntenin-1	Calsyntenin-1	Cathepsin D	C-C chemokine receptor type 7*
C-C motif chemokine 1*	C-C motif chemokine 22*	C-C motif chemokine 5*	Ceruloplasmin
Coagulation factor XIII B chain	Complement C3*	Creatine kinase B-type*	C-X-C motif chemokine 10*
C-X-C motif chemokine 11*	Cystatin A	Growth/differentiation factor 2*	HLA class II histocompatibility antigen gamma chain*
Insulin-degrading enzyme*	Interleukin-13 receptor subunit alpha-2*	Interleukin-19*	Interleukin-21 receptor*
Interleukin-23 subunit alpha*	Keratin, type I cytoskeletal 19*	Kremen protein 2*	Low-density lipoprotein receptor*
Lymphotoxin beta	Macrophage migration inhibitory factor*	Matrilysin*	Matrix metalloproteinase-14*
Matrix metalloproteinase-9*	Metalloproteinase inhibitor 3*	MHC class I polypeptide-related sequence A*	Neural cell adhesion molecule 1*
Neurogenic differentiation factor 1*	Neurturin	Neutrophil-activating peptide 2	Orexin receptor type 1*
Platelet-derived growth factor D*	Polyubiquitin-B*	Progranulin	Protein S100-A10
Secreted frizzled-related protein 1*	Sialic acid-binding Ig-like lectin 5*	Stromelysin-2*	Thrombopoietin
Toll-like receptor 2*	Toll-like receptor 4*	Transferrin receptor protein 1*	Transforming growth factor beta receptor type 3*
Transforming growth factor beta-1	Triggering receptor expressed on myeloid cells 1*	Tumor necrosis factor ligand superfamily member 10*	Tumor necrosis factor ligand superfamily member 11*
Tumor necrosis factor ligand superfamily member 15*	Tumor necrosis factor ligand superfamily member 8*	Tumor necrosis factor receptor superfamily member 19*	Tumor necrosis factor receptor superfamily member 27*
Vascular endothelial growth factor A*	Vascular endothelial growth factor A*	Vascular endothelial growth factor C*	Vascular endothelial growth factor D*
Vascular endothelial growth factor receptor 2*			
Proteins down-regulated			
Activin receptor type-1*	CD166 antigen	Angiopoietin-related protein 2*	Apolipoprotein C-II*
SWI/SNF-related matrix-associated actin-dependent regulator of chromatin subfamily E member 1*	Tumor necrosis factor receptor superfamily member 13C*	Beta-2-microglobulin*	Bone morphogenetic protein 7*
Calbindin	Fibroblast growth factor receptor 3*	Interleukin-36 receptor antagonist protein*	pro-Glucagon
Receptor-interacting serine/threonine-protein kinase 1*	Transcription initiation factor TFIID subunit 4*	T lymphocyte activation antigen CD80*	

*The referred article used alternative or short names

The stimulation with bFGF did not affect the number or size of released AT-MSC-EVs but it reduced their antigenic properties, stimulating the stabilization of vessel growth, both in cultured human microvascular endothelial cells and in an in vivo model of severe combined immunodeficiency [64]. The analysis of these EVs showed that angiogenic and antiangiogenic proteins such as tumor necrosis factor ligand superfamily member 13, artemin, lactadherin, MMP-20, angiopoietin-related protein 7, thrombospondin, angiostatin and endostatin were lost, while new angiogenesis modulatory proteins, such as tumor necrosis factor ligand superfamily member 11 and matrilysin were gained. Regarding miRNA profile, differences in the expression of 55 different miRNAs were observed [64] (Table 3).

Finally, AT-MSC-EVs have been transfected with lentiviral particles to produce EVs enriched in miRNA 375 [77], miRNA-125b [119] and miRNA 101 [118]. The miRNA-375-enriched EVs promoted bone regeneration in an in vivo model of calvarial defects. AT-MSC-EVs enriched in miRNA-125b [119] and miRNA 101 [118] induced a reduction in cell proliferation of hepatocellular carcinoma cells and inhibited osteosarcoma cell invasion and migration in vitro*,* respectively. In addition, miRNA-101-enriched EVs also induced inhibition of osteosarcoma metastasis in a lung metastasis model in vivo [118].

## Conclusions

There is an increasing interest in the study of EVs as new therapeutic options in several research fields, due to their role in different biological processes, including cell proliferation, apoptosis, angiogenesis, inflammation and immune response, among others. Their potential is based upon the molecules transported inside these particles. Therefore, both molecule identification and an understanding of the molecular functions and biological processes in which they are involved are essential to advance this area of research. To the best of our knowledge, the presence of 591 proteins and 604 miRNAs in human AT-MSC-EVs has been described. The most important molecular function enabled by them is the binding function, which supports their role in cell communication. Regarding the biological processes, the proteins detected are mainly involved in signal transduction, while most miRNAs take part in negative regulation of gene expression. The involvement of both molecules in essential biological processes such as inflammation, angiogenesis, cell proliferation, apoptosis and migration, supports the beneficial effects of human AT-MSC-EVs observed in both in vitro and in vivo studies, in diseases of the musculoskeletal and cardiovascular systems, kidney, and skin.

Interestingly, the contents of AT-MSC-EVs can be modified by cell stimulation and different cell culture conditions, such as oxidative stress or hypoxia, to engineer a cargo selection with improved antigenic, anti-inflammatory or immunosuppressive effects. Moreover, it is also possible to enrich specific miRNAs in the cargo via transfection of AT-MSC with lentiviral particles. These modifications have enhanced the positive effects in skin flap survival, immune response, bone regeneration and cancer treatment. This phenomenon opens new avenues to examine the therapeutic potential of AT-MSC-EVs.

## Supplementary Information

Table 1S Proteins detected in human AT-MSC-EVs in alphabetical order: gene ontology annotations of molecular functions. (DOC 717 kb)

Table 2S Proteins detected in human AT-MSC-EVs in alphabetical order: gene ontology annotations of biological processes (XLSX 477 kb)

Table 3S miRNAs detected in human AT-MSC-EVs: gene ontology annotations of molecular functions. (DOC 251 kb)

Table 4S miRNAs detected in human AT-MSC-EVs: gene ontology annotations of biological processes. (DOC 1.87 mb)

## Abbreviations

Apo B-100: apolipoprotein B-100
AT: adipose tissue
AT-MSC-EVs: adipose mesenchymal cell–derived extracellular vesicles
Beta ig-h3: transforming growth factor-beta-induced protein ig-h3
bFGF: basic fibroblast growth factor
BMP-1: bone morphogenetic protein 1
BMPR-1A: bone morphogenetic protein receptor type-1A
BMPR-2: bone morphogenetic protein receptor type-2
BM: bone marrow
BM-MSC: bone marrow mesenchymal stem cells
EF-1-alpha-1: elongation factor 1-alpha 1
EF-2: elongation factor 2
EGF: epidermal growth factor
EMBL-EBI: the European Bioinformatics Institute
EV: extracellular vesicle
FGF-4: fibroblast growth factor 4
FGFR-1: fibroblast growth factor receptor 1
FGFR-4: fibroblast growth factor receptor 4
FLG-2: filaggrin-2
G alpha-13: guanine nucleotide-binding protein subunit alpha-13
GAPDH: glyceraldehyde 3-phosphate dehydrogenase
GO: gene ontology
IBP-7: insulin-like growth factor-binding protein 7
IL-1 alpha: interleukin-1 alpha
IL-4: interleukin-4
IL-6: interleukin-6
IL-6RB: interleukin-6 receptor subunit beta
IL-10: interleukin-10
IL-17RD: interleukin-17 receptor D
IL-20RA: interleukin-20 receptor subunit alpha
ISEV: International Society for Extracellular Vesicles
ITI-HC2: inter-alpha-trypsin inhibitor heavy chain H2
LIF: leukemia inhibitory factor
LTBP-1: latent-transforming growth factor beta-binding protein 1
MAP kinase 1: mitogen-activated protein kinase 1
MAP kinase 3: mitogen-activated protein kinase 3
miRNA: microRNA
MMP-9: matrix metalloproteinase-9
MMP-14: matrix metalloproteinase-14
MMP-20: matrix metalloproteinase-20
mRNA: messenger RNA
MSC: mesenchymal stem cells
OSM: oncostatin-M
PDGF: platelet-derived growth factor
PDGFR-alpha: platelet-derived growth factor receptor alpha.
PDGFR-beta: platelet-derived growth factor receptor beta
rRNA: small ribosomal RNA
SCFR: mast/stem cell growth factor receptor Kit
scRNA: small cytoplasmic RNA
SL-2: stromelysin-2
SL-3: stromelysin-3
snRNA: small nuclear RNA
snoRNA: small nucleolar RNA
TGFR-2: TGF-beta receptor type-2
tRNA: transfer RNA
UniProtKB: Universal Protein Knowledgebase
VDB: vitamin D-binding protein
VEGF-A: vascular endothelial growth factor A
VEGFR-2: vascular endothelial growth factor receptor 2
VEGFR-3: vascular endothelial growth factor receptor
