# Supplementary material for: Extracellular Vesicles from Human Adipose-Derived Mesenchymal Stem Cells: A Review of Common Cargos
Source: Stem Cell Rev Rep. 2021 Apr 26;18(3):854–901. doi: 10.1007/s12015-021-10155-5 (PMC8942954; doi:10.1007/s12015-021-10155-5)
Supplement: Supplementary file 1 — Proteins detected in human AT-MSC-EVs in alphabetical order: gene ontology annotations of molecular functions. (DOC 717 kb) [file 12015_2021_10155_MOESM1_ESM.doc]

| **Table 1S** Proteins detected in human AT-MSC-EVs in alphabetical order: gene ontology annotations of molecular functions | | |
| --- | --- | --- |
| **Protein** | **Gene ontology terms of molecular functions** | |
| 5'-AMP-activated protein kinase catalytic subunit alpha-1 | GO:0000166 nucleotide binding  GO:0003682 chromatin binding  GO:0004672 protein kinase activity  GO:0004674 protein serine/threonine kinase activity  GO:0004679 AMP-activated protein kinase activity  GO:0004691 cAMP-dependent protein kinase activity  GO:0005515 protein binding  GO:0005524 ATP binding  GO:0008022 protein C-terminus binding  GO:0016301 kinase activity  GO:0016740 transferase activity | GO:0035174 histone serine kinase activity  GO:0044877 protein-containing complex binding  GO:0046872 metal ion binding  GO:0047322 [hydroxymethylglutaryl-CoA reductase (NADPH)] kinase activity  GO:0048156 tau protein binding  GO:0050321 tau-protein kinase activity  GO:0050405 [acetyl-CoA carboxylase] kinase activity  GO:0106310 protein serine kinase activity  GO:0106311 protein threonine kinase activity |
| 72 kDa type IV collagenase | GO:0004175 endopeptidase activity  GO:0004222 metalloendopeptidase activity  GO:0004252 serine-type endopeptidase activity  GO:0005515 protein binding  GO:0008233 peptidase activity | GO:0008237 metallopeptidase activity  GO:0008270 zinc ion binding  GO:0016787 hydrolase activity  GO:0046872 metal ion binding |
| A disintegrin and metalloproteinase with thrombospondin motifs 1 | GO:0004222 metalloendopeptidase activity  GO:0005515 protein binding  GO:0008201 heparin binding  GO:0008233 peptidase activity | GO:0008237 metallopeptidase activity  GO:0008270 zinc ion binding  GO:0016787 hydrolase activity  GO:0046872 metal ion binding |
| A disintegrin and metalloproteinase with thrombospondin motifs 2 | GO:0004222 metalloendopeptidase activity  GO:0008233 peptidase activity  GO:0008237 metallopeptidase activity | GO:0008270 zinc ion binding  GO:0016787 hydrolase activity  GO:0046872 metal ion binding |
| A disintegrin and metalloproteinase with thrombospondin motifs 4 | GO:0002020 protease binding  GO:0004222 metalloendopeptidase activity  GO:0005515 protein binding  GO:0008233 peptidase activity | GO:0008237 metallopeptidase activity  GO:0016787 hydrolase activity  GO:0046872 metal ion binding |
| A disintegrin and metalloproteinase with thrombospondin motifs 17 | GO:0004222 metalloendopeptidase activity  GO:0008233 peptidase activity  GO:0008237 metallopeptidase activity | GO:0016787 hydrolase activity  GO:0046872 metal ion binding |
| A disintegrin and metalloproteinase with thrombospondin motifs 18 | GO:0004222 metalloendopeptidase activity  GO:0008233 peptidase activity  GO:0008237 metallopeptidase activity | GO:0016787 hydrolase activity  GO:0046872 metal ion binding |
| A disintegrin and metalloproteinase with thrombospondin motifs 19 | GO:0004222 metalloendopeptidase activity  GO:0008233 peptidase activity  GO:0008237 metallopeptidase activity | GO:0016787 hydrolase activity  GO:0046872 metal ion binding |
| Acidic fibroblast growth factor intracellular-binding protein | GO:0017134 fibroblast growth factor binding | |
| Activated CDC42 kinase 1 | GO:0000166 nucleotide binding  GO:0004672 protein kinase activity  GO:0004674 protein serine/threonine kinase activity  GO:0004712 protein serine/threonine/tyrosine kinase activity  GO:0004713 protein tyrosine kinase activity  GO:0004714 transmembrane receptor protein tyrosine kinase activity  GO:0004715 non-membrane spanning protein tyrosine kinase activity  GO:0005095 GTPase inhibitor activity  GO:0005102 signaling receptor binding | GO:0005154 epidermal growth factor receptor binding  GO:0005515 protein binding  GO:0005524 ATP binding  GO:0016301 kinase activity  GO:0016740 transferase activity  GO:0031625 ubiquitin protein ligase binding  GO:0042802 identical protein binding  GO:0046872 metal ion binding  GO:0050699 WW domain binding  GO:0106310 protein serine kinase activity  GO:0106311 protein threonine kinase activity |
| Activin receptor type-1B | GO:0000166 nucleotide binding  GO:0004672 protein kinase activity  GO:0004674 protein serine/threonine kinase activity  GO:0004675 transmembrane receptor protein serine/threonine kinase activity  GO:0005515 protein binding  GO:0005524 ATP binding  GO:0016301 kinase activity  GO:0016361 activin receptor activity, type I | GO:0016740 transferase activity  GO:0017002 activin-activated receptor activity  GO:0019838 growth factor binding  GO:0031625 ubiquitin protein ligase binding  GO:0034711 inhibin binding  GO:0046332 SMAD binding  GO:0046872 metal ion binding  GO:0048185 activin binding |
| Activin receptor type-2B | GO:0000166 nucleotide binding  GO:0004672 protein kinase activity  GO:0004674 protein serine/threonine kinase activity  GO:0004675 transmembrane receptor protein serine/threonine kinase activity  GO:0005515 protein binding  GO:0005524 ATP binding  GO:0016301 kinase activity | GO:0016362 activin receptor activity, type II  GO:0016740 transferase activity  GO:0017002 activin-activated receptor activity  GO:0019838 growth factor binding  GO:0046872 metal ion binding  GO:0048185 activin binding |
| Adenomatous polyposis coli protein | GO:0005515 protein binding  GO:0008013 beta-catenin binding  GO:0008017 microtubule binding  GO:0019887 protein kinase regulator activity  GO:0019901 protein kinase binding | GO:0031625 ubiquitin protein ligase binding  GO:0045295 gamma-catenin binding  GO:0045296 cadherin binding  GO:0051010 microtubule plus-end binding  GO:0070840 dynein complex binding |

| Adhesion G protein-coupled receptor B1 | GO:0001530 lipopolysaccharide binding  GO:0001786 phosphatidylserine binding  GO:0004888 transmembrane signaling receptor activity | GO:0004930 G protein-coupled receptor activity  GO:0005515 protein binding  GO:0030165 PDZ domain binding |
| --- | --- | --- |
| Adhesion G protein-coupled receptor E5 | GO:0004888 transmembrane signaling receptor activity  GO:0004930 G protein-coupled receptor activity | GO:0005509 calcium ion binding  GO:0005515 protein binding |
| ADP-ribosyl cyclase/cyclic ADP-ribose hydrolase 1 | GO:0003953 NAD+ nucleosidase activity  GO:0016740 transferase activity  GO:0016787 hydrolase activity  GO:0016798 hydrolase activity, acting on glycosyl bonds | GO:0016849 phosphorus-oxygen lyase activity  GO:0042802 identical protein binding  GO:0050135 NAD(P)+ nucleosidase activity  GO:0061809 NAD+ nucleotidase, cyclic ADP-ribose generating |
| Agouti-related protein | GO:0005102 signaling receptor binding  GO:0005184 neuropeptide hormone activity | GO:0031779 melanocortin receptor binding  GO:0070996 type 1 melanocortin receptor binding |
| Alkaline phosphatase, placental type | GO:0000287 magnesium ion binding  GO:0003824 catalytic activity  GO:0004035 alkaline phosphatase activity  GO:0005515 protein binding | GO:0008270 zinc ion binding  GO:0016787 hydrolase activity  GO:0016791 phosphatase activity  GO:0046872 metal ion binding |
| Alpha-1-acid glycoprotein 1 | GO:0005515 protein binding | |
| Alpha-1-antitrypsin | GO:0002020 protease binding  GO:0004867 serine-type endopeptidase inhibitor activity | GO:0005515 protein binding  GO:0030414 peptidase inhibitor activity  GO:0042802 identical protein binding |
| Alpha-1B-glycoprotein | GO:0003674 molecular_function | |
| Alpha-fetoprotein | GO:0005504 fatty acid binding  GO:0005515 protein binding | GO:0008270 zinc ion binding  GO:0046872 metal ion binding |
| Alpha-lactalbumin | GO:0004461 lactose synthase activity  GO:0005509 calcium ion binding | GO:0046872 metal ion binding |
| Aminopeptidase N | GO:0001618 virus receptor activity  GO:0004177 aminopeptidase activity  GO:0008233 peptidase activity  GO:0008237 metallopeptidase activity  GO:0008270 zinc ion binding | GO:0016787 hydrolase activity  GO:0038023 signaling receptor activity  GO:0042277 peptide binding  GO:0046872 metal ion binding  GO:0070006 metalloaminopeptidase activity |
| Amphiregulin | GO:0005154 epidermal growth factor receptor binding  GO:0005125 cytokine activity | GO:0005515 protein binding  GO:0008083 growth factor activity |
| Angiopoietin-1 | GO:0005102 signaling receptor binding | GO:0030971 receptor tyrosine kinase binding |
| Angiopoietin-1 receptor | GO:0000166 nucleotide binding  GO:0004672 protein kinase activity  GO:0004713 protein tyrosine kinase activity GO:0004714 transmembrane receptor protein tyrosine kinase activity  GO:0005515 protein binding | GO:0005524 ATP binding  GO:0016301 kinase activity  GO:0016740 transferase activity  GO:0019838 growth factor binding  GO:0038023 signaling receptor activity  GO:0042802 identical protein binding |
| Angiopoietin-4 | GO:0005102 signaling receptor binding  GO:0030297 transmembrane receptor protein tyrosine kinase activator activity | GO:0030971 receptor tyrosine kinase binding |
| Angiopoietin-related protein 1 | GO:0005102 signaling receptor binding | |
| Angiopoietin-related protein 2 | GO:0005102 signaling receptor binding | GO:0005515 protein binding |
| Angiopoietin-related protein 7 | GO:0005515 protein binding | GO:0042802 identical protein binding |
| Angiostatin (cleaved from plasminogen) | GO:0004175 endopeptidase activity  GO:0004252 serine-type endopeptidase activity  GO:0005102 signaling receptor binding  GO:0005515 protein binding  GO:0008233 peptidase activity  GO:0008236 serine-type peptidase activity  GO:0016787 hydrolase activity | GO:0019899 enzyme binding  GO:0019900 kinase binding  GO:0019904 protein domain specific binding  GO:0034185 apolipoprotein binding  GO:0051087 chaperone binding  GO:1990405 protein antigen binding |
| Annexin A5 | GO:0004859 phospholipase inhibitor activity  GO:0005509 calcium ion binding  GO:0005515 protein binding | GO:0005543 phospholipid binding  GO:0005544 calcium-dependent phospholipid binding |
| Annexin A7 | GO:0003723 RNA binding  GO:0005178 integrin binding  GO:0005509 calcium ion binding  GO:0005515 protein binding | GO:0005544 calcium-dependent phospholipid binding  GO:0048306 calcium-dependent protein binding |
| Antileukoproteinase | GO:0003677 DNA binding  GO:0003729 mRNA binding  GO:0004866 endopeptidase inhibitor activity  GO:0004867 serine-type endopeptidase inhibitor activity | GO:0005515 protein binding  GO:0019899 enzyme binding  GO:0030414 peptidase inhibitor activity |
| Apelin receptor | GO:0004930 G protein-coupled receptor activity  GO:0005515 protein binding | GO:0038023 signaling receptor activity  GO:0060182 apelin receptor activity |

| Apolipoprotein A-IV | GO:0005319 lipid transporter activity  GO:0005507 copper ion binding  GO:0005515 protein binding  GO:0005543 phospholipid binding  GO:0008289 lipid binding  GO:0015485 cholesterol binding | GO:0016209 antioxidant activity  GO:0031210 phosphatidylcholine binding  GO:0042802 identical protein binding  GO:0042803 protein homodimerization activity  GO:0060228 phosphatidylcholine-sterol O-acyltransferase activator activity  GO:0120020 cholesterol transfer activity | | | |  | |
| --- | --- | --- | --- | --- | --- | --- | --- |
| Apolipoprotein B-100 | GO:0005319 lipid transporter activity  GO:0005515 protein binding  GO:0005543 phospholipid binding  GO:0008201 heparin binding | GO:0035473 lipase binding  GO:0050750 low-density lipoprotein particle receptor binding  GO:0120020 cholesterol transfer activity | | | |  | |
| Apolipoprotein C-I | GO:0004859 phospholipase inhibitor activity  GO:0005504 fatty acid binding  GO:0005515 protein binding | GO:0031210 phosphatidylcholine binding  GO:0055102 lipase inhibitor activity  GO:0060228 phosphatidylcholine-sterol O-acyltransferase activator activity | | | |  | |
| Apolipoprotein C-II | GO:0005515 protein binding  GO:0008047 enzyme activator activity  GO:0008289 lipid binding  GO:0016004 phospholipase activator activity | GO:0042802 identical protein binding  GO:0043274 phospholipase binding  GO:0055102 lipase inhibitor activity  GO:0060230 lipoprotein lipase activator activity | | | |  | |
| Apolipoprotein E | GO:0001540 amyloid-beta binding  GO:0005102 signaling receptor binding  GO:0005198 structural molecule activity  GO:0005319 lipid transporter activity  GO:0005515 protein binding  GO:0005543 phospholipid binding  GO:0008201 heparin binding  GO:0008289 lipid binding  GO:0016209 antioxidant activity  GO:0042802 identical protein binding  GO:0042803 protein homodimerization activity | GO:0043395 heparan sulfate proteoglycan binding  GO:0044877 protein-containing complex binding  GO:0046911 metal chelating activity  GO:0046983 protein dimerization activity  GO:0048156 tau protein binding  GO:0050750 low-density lipoprotein particle receptor binding  GO:0060228 phosphatidylcholine-sterol O-acyltransferase activator activity  GO:0070326 very-low-density lipoprotein particle receptor binding  GO:0071813 lipoprotein particle binding  GO:0120020 cholesterol transfer activity | | | |  | |
| Apolipoprotein M | GO:0005319 lipid transporter activity  GO:0005543 phospholipid binding | GO:0016209 antioxidant activity | | | |  | |
| Apoptosis regulator BAX | GO:0005515 protein binding  GO:0008289 lipid binding  GO:0015267 channel activity  GO:0030544 Hsp70 protein binding  GO:0042802 identical protein binding | GO:0042803 protein homodimerization activity  GO:0046982 protein heterodimerization activity  GO:0051087 chaperone binding  GO:0051434 BH3 domain binding | | | |  | |
| Artemin | GO:0005102 signaling receptor binding GO:0005515 protein binding  GO:0008083 growth factor activity | GO:0030116 glial cell-derived neurotrophic factor receptor binding  GO:0030971 receptor tyrosine kinase binding | | | |  | |
| Aspartyl/asparaginyl beta-hydroxylase | GO:0004597 peptide-aspartate beta-dioxygenase activity  GO:0005198 structural molecule activity  GO:0005509 calcium ion binding  GO:0005515 protein binding  GO:0008307 structural constituent of muscle  GO:0009055 electron transfer activity | GO:0016491 oxidoreductase activity  GO:0044325 ion channel binding  GO:0046872 metal ion binding  GO:0051213 dioxygenase activity  GO:0062101 peptidyl-aspartic acid 3-dioxygenase activity | | | |  | |
| Basal cell adhesion molecule | GO:0004888 transmembrane signaling receptor activity  GO:0005055 laminin receptor activity  GO:0005515 protein binding | | | GO:0008022 protein C-terminus binding  GO:0043236 laminin binding |  | | |
| BCL2/adenovirus E1B 19 kDa protein-interacting protein 2 | GO:0004309 exopolyphosphatase activity  GO:0005096 GTPase activator activity | | GO:0005509 calcium ion binding  GO:0005515 protein binding | | | |  |
| Beta-2-microglobulin | GO:0005515 protein binding  GO:0042802 identical protein binding | | GO:0042803 protein homodimerization activity | | | |  |
| Beta-Ala-His dipeptidase | GO:0004180 carboxypeptidase activity  GO:0008233 peptidase activity  GO:0008237 metallopeptidase activity  GO:0016787 hydrolase activity | | GO:0016805 dipeptidase activity  GO:0046872 metal ion binding  GO:0070573 metallodipeptidase activity | | | |  |
| Beta-defensin 1 | GO:0005515 protein binding  GO:0031731 CCR6 chemokine receptor binding | | GO:0042802 identical protein binding | | | |  |
| Beta-defensin 4A | GO:0005515 protein binding  GO:0031731 CCR6 chemokine receptor binding | | GO:0042056 chemoattractant activity | | | |  |
| Beta-endorphin (Pro-opiomelanocortin) | GO:0001664 G protein-coupled receptor binding  GO:0005102 signaling receptor binding  GO:0005179 hormone activity  GO:0005515 protein binding | | GO:0031781 type 3 melanocortin receptor binding  GO:0031782 type 4 melanocortin receptor binding  GO:0070996 type 1 melanocortin receptor binding | | | |  |
| BMP-binding endothelial regulator protein | - | | | | |  | |
| Bone morphogenetic protein 1 | GO:0004222 metalloendopeptidase activity  GO:0005125 cytokine activity  GO:0005515 protein binding  GO:0005509 calcium ion binding  GO:0008083 growth factor activity  GO:0008233 peptidase activity | GO:0008237 metallopeptidase activity  GO:0008270 zinc ion binding  GO:0016787 hydrolase activity  GO:0042802 identical protein binding  GO:0046872 metal ion binding | | | |  | |

| Bone morphogenetic protein 3 | GO:0005102 signaling receptor binding  GO:0005125 cytokine activity | GO:0008083 growth factor activity  GO:0070700 BMP receptor binding |
| --- | --- | --- |
| Bone morphogenetic protein 4 | GO:0005125 cytokine activity  GO:0005515 protein binding  GO:0008083 growth factor activity  GO:0008201 heparin binding | GO:0039706 co-receptor binding  GO:0042056 chemoattractant activity  GO:0070700 BMP receptor binding |
| Bone morphogenetic protein 5 | GO:0005125 cytokine activity  GO:0005515 protein binding | GO:0008083 growth factor activity  GO:0070700 BMP receptor binding |
| Bone morphogenetic protein 6 | GO:0005125 cytokine activity  GO:0008083 growth factor activity | GO:0046982 protein heterodimerization activity  GO:0070700 BMP receptor binding |
| Bone morphogenetic protein 7 | GO:0005125 cytokine activity  GO:0005515 protein binding  GO:0008083 growth factor activity | GO:0008201 heparin binding  GO:0070700 BMP receptor binding |
| Bone morphogenetic protein 8B | GO:0005125 cytokine activity  GO:0008083 growth factor activity | GO:0070700 BMP receptor binding |
| Bone morphogenetic protein receptor type-1A | GO:0000166 nucleotide binding  GO:0000981 DNA-binding transcription factor activity, RNA polymerase II-specific  GO:0004672 protein kinase activity  GO:0004674 protein serine/threonine kinase activity GO:0004675 transmembrane receptor protein serine/threonine kinase activity  GO:0005025 transforming growth factor beta receptor activity, type I | GO:0005515 protein binding  GO:0005524 ATP binding  GO:0016301 kinase activity  GO:0016740 transferase activity  GO:0042803 protein homodimerization activity  GO:0046332 SMAD binding  GO:0046872 metal ion binding  GO:0098821 BMP receptor activity |
| Bone morphogenetic protein receptor type-1B | GO:0000166 nucleotide binding  GO:0004672 protein kinase activity  GO:0004674 protein serine/threonine kinase activity  GO:0004675 transmembrane receptor protein serine/threonine kinase activity  GO:0005025 transforming growth factor beta receptor activity, type I  GO:0005515 protein binding | GO:0005524 ATP binding  GO:0016301 kinase activity  GO:0016740 transferase activity  GO:0036122 BMP binding  GO:0046332 SMAD binding  GO:0046872 metal ion binding  GO:0098821 BMP receptor activity |
| Bone morphogenetic protein receptor type-2 | GO:0000166 nucleotide binding  GO:0004672 protein kinase activity  GO:0004674 protein serine/threonine kinase activity  GO:0004675 transmembrane receptor protein serine/threonine kinase activity  GO:0005024 transforming growth factor beta-activated receptor activity  GO:0005515 protein binding  GO:0005524 ATP binding | GO:0016301 kinase activity  GO:0016362 activin receptor activity, type II  GO:0016740 transferase activity  GO:0019838 growth factor binding  GO:0036122 BMP binding  GO:0045296 cadherin binding  GO:0046872 metal ion binding  GO:0098821 BMP receptor activity  GO:1990782 protein tyrosine kinase binding |
| Brain-derived neurotrophic factor | GO:0005102 signaling receptor binding  GO:0005163 nerve growth factor receptor binding | GO:0005515 protein binding  GO:0008083 growth factor activity |
| Cadherin-1 | GO:0005509 calcium ion binding  GO:0005515 protein binding  GO:0008013 beta-catenin binding  GO:0008092 cytoskeletal protein binding  GO:0030506 ankyrin binding  GO:0032794 GTPase activating protein binding | GO:0042802 identical protein binding  GO:0045295 gamma-catenin binding  GO:0045296 cadherin binding  GO:0046872 metal ion binding  GO:0050839 cell adhesion molecule binding |
| Cadherin-2 | GO:0005509 calcium ion binding  GO:0005515 protein binding  GO:0008013 beta-catenin binding  GO:0008092 cytoskeletal protein binding  GO:0019899 enzyme binding  GO:0019901 protein kinase binding | GO:0019903 protein phosphatase binding  GO:0042802 identical protein binding  GO:0045294 alpha-catenin binding  GO:0045295 gamma-catenin binding  GO:0045296 cadherin binding  GO:0046872 metal ion binding |
| Cadherin-5 | GO:0005102 signaling receptor binding  GO:0005509 calcium ion binding  GO:0005515 protein binding  GO:0008013 beta-catenin binding  GO:0008092 cytoskeletal protein binding  GO:0019903 protein phosphatase binding  GO:0043184 vascular endothelial growth factor receptor 2 binding | GO:0044325 ion channel binding  GO:0045296 cadherin binding  GO:0046872 metal ion binding  GO:0070051 fibrinogen binding  GO:0070700 BMP receptor binding  GO:1990782 protein tyrosine kinase binding |
| Cadherin-11 | GO:0005509 calcium ion binding  GO:0008092 cytoskeletal protein binding | GO:0045296 cadherin binding  GO:0046872 metal ion binding |
| Cadherin-13 | GO:0005509 calcium ion binding  GO:0008092 cytoskeletal protein binding  GO:0030169 low-density lipoprotein particle binding  GO:0042803 protein homodimerization activity | GO:0045296 cadherin binding  GO:0046872 metal ion binding  GO:0055100 adiponectin binding  GO:0071813 lipoprotein particle binding |
| Cadherin-related family member 2 | GO:0005509 calcium ion binding  GO:0005515 protein binding | GO:0050839 cell adhesion molecule binding |
| Cadherin-related family member 5 | GO:0005509 calcium ion binding  GO:0005515 protein binding | GO:0008013 beta-catenin binding  GO:0050839 cell adhesion molecule binding |
| Calbindin | GO:0005499 vitamin D binding  GO:0005509 calcium ion binding  GO:0005515 protein binding  GO:0008270 zinc ion binding | GO:0099534 calcium ion binding involved in regulation of presynaptic cytosolic calcium ion concentration  GO:0099567 calcium ion binding involved in regulation of postsynaptic cytosolic calcium ion concentration |
| Calcitonin | GO:0005102 signaling receptor binding  GO:0005179 hormone activity  GO:0005515 protein binding | GO:0031716 calcitonin receptor binding  GO:0042802 identical protein binding |
| Calreticulin | GO:0001849 complement component C1q complex binding  GO:0003677 DNA binding  GO:0003723 RNA binding  GO:0003729 mRNA binding  GO:0005178 integrin binding  GO:0005506 iron ion binding  GO:0005509 calcium ion binding  GO:0005515 protein binding  GO:0008270 zinc ion binding | GO:0030246 carbohydrate binding  GO:0031625 ubiquitin protein ligase binding  GO:0042277 peptide binding  GO:0042562 hormone binding  GO:0044183 protein folding chaperone  GO:0046872 metal ion binding  GO:0050681 androgen receptor binding  GO:0051082 unfolded protein binding  GO:0051087 chaperone binding |
| Calsyntenin-1 | GO:0001540 amyloid-beta binding  GO:0005509 calcium ion binding  GO:0005515 protein binding | GO:0019894 kinesin binding  GO:0042988 X11-like protein binding |
| Carboxypeptidase N subunit 2 | GO:0030234 enzyme regulator activity | |
| Carcinoembryonic antigen-related cell adhesion molecule 7 | - | |
| Caspase-3 | GO:0002020 protease binding  GO:0004190 aspartic-type endopeptidase activity  GO:0004197 cysteine-type endopeptidase activity  GO:0004861 cyclin-dependent protein serine/threonine kinase inhibitor activity  GO:0005123 death receptor binding  GO:0005515 protein binding  GO:0008233 peptidase activity  GO:0008234 cysteine-type peptidase activity | GO:0016005 phospholipase A2 activator activity  GO:0016787 hydrolase activity  GO:0044877 protein-containing complex binding  GO:0097153 cysteine-type endopeptidase activity involved in apoptotic process  GO:0097199 cysteine-type endopeptidase activity involved in apoptotic signaling pathway  GO:0097200 cysteine-type endopeptidase activity involved in execution phase of apoptosis |
| Caspase-8 | GO:0004197 cysteine-type endopeptidase activity  GO:0005123 death receptor binding  GO:0005164 tumor necrosis factor receptor binding  GO:0005515 protein binding  GO:0008233 peptidase activity  GO:0008234 cysteine-type peptidase activity  GO:0016787 hydrolase activity  GO:0031625 ubiquitin protein ligase binding  GO:0035877 death effector domain binding | GO:0042802 identical protein binding  GO:0044877 protein-containing complex binding  GO:0097110 scaffold protein binding  GO:0097153 cysteine-type endopeptidase activity involved in apoptotic process  GO:0097199 cysteine-type endopeptidase activity involved in apoptotic signaling pathway  GO:0097200 cysteine-type endopeptidase activity involved in execution phase of apoptosis |
| Cathepsin B | GO:0004197 cysteine-type endopeptidase activity  GO:0005515 protein binding  GO:0005518 collagen binding  GO:0008233 peptidase activity | GO:0008234 cysteine-type peptidase activity  GO:0016787 hydrolase activity  GO:0043394 proteoglycan binding |
| Cathepsin D | GO:0004190 aspartic-type endopeptidase activity  GO:0005515 protein binding  GO:0008233 peptidase activity | GO:0016787 hydrolase activity  GO:0070001 aspartic-type peptidase activity |
| C-C chemokine receptor type 1 | GO:0004435 phosphatidylinositol phospholipase C activity  GO:0004930 G protein-coupled receptor activity  GO:0004950 chemokine receptor activity  GO:0005515 protein binding | GO:0016493 C-C chemokine receptor activity  GO:0019957 C-C chemokine binding  GO:0035717 chemokine (C-C motif) ligand 7 binding  GO:0071791 chemokine (C-C motif) ligand 5 binding |
| C-C chemokine receptor type 2 | GO:0004930 G protein-coupled receptor activity  GO:0004950 chemokine receptor activity  GO:0005515 protein binding  GO:0016493 C-C chemokine receptor activity | GO:0019957 C-C chemokine binding  GO:0031727 CCR2 chemokine receptor binding  GO:0042802 identical protein binding |
| C-C chemokine receptor type 3 | GO:0004930 G protein-coupled receptor activity  GO:0004950 chemokine receptor activity  GO:0005515 protein binding | GO:0016493 C-C chemokine receptor activity  GO:0019957 C-C chemokine binding |
| C-C chemokine receptor type 4 | GO:0004930 G protein-coupled receptor activity  GO:0004950 chemokine receptor activity  GO:0005515 protein binding | GO:0016493 C-C chemokine receptor activity  GO:0019957 C-C chemokine binding |
| C-C chemokine receptor type 5 | GO:0001618 virus receptor activity  GO:0003779 actin binding  GO:0004435 phosphatidylinositol phospholipase C activity  GO:0004930 G protein-coupled receptor activity  GO:0004950 chemokine receptor activity  GO:0005515 protein binding | GO:0015026 coreceptor activity  GO:0016493 C-C chemokine receptor activity  GO:0019957 C-C chemokine binding  GO:0042802 identical protein binding  GO:0071791 chemokine (C-C motif) ligand 5 binding |
| C-C chemokine receptor type 6 | GO:0004930 G protein-coupled receptor activity  GO:0004950 chemokine receptor activity  GO:0005515 protein binding | GO:0016493 C-C chemokine receptor activity  GO:0019957 C-C chemokine binding  GO:0038023 signaling receptor activity |
| C-C chemokine receptor type 7 | GO:0004930 G protein-coupled receptor activity  GO:0004950 chemokine receptor activity  GO:0016493 C-C chemokine receptor activity  GO:0019957 C-C chemokine binding  GO:0035758 chemokine (C-C motif) ligand 21 binding | GO:0035758 chemokine (C-C motif) ligand 21 binding  GO:0038117 C-C motif chemokine 19 receptor activity  GO:0038121 C-C motif chemokine 21 receptor activity |
| C-C chemokine receptor type 9 | GO:0004930 G protein-coupled receptor activity  GO:0004950 chemokine receptor activity | GO:0016493 C-C chemokine receptor activity  GO:0019957 C-C chemokine binding |
| C-C motif chemokine 1 | GO:0005125 cytokine activity  GO:0008009 chemokine activity | GO:0048020 CCR chemokine receptor binding |
| C-C motif chemokine 2 | GO:0004672 protein kinase activity  GO:0005102 signaling receptor binding  GO:0005125 cytokine activity  GO:0005515 protein binding | GO:0008009 chemokine activity  GO:0031727 CCR2 chemokine receptor binding  GO:0048020 CCR chemokine receptor binding |
| C-C motif chemokine 3 | GO:0004672 protein kinase activity  GO:0004698 calcium-dependent protein kinase C activity  GO:0005125 cytokine activity  GO:0005515 protein binding  GO:0008009 chemokine activity  GO:0016004 phospholipase activator activity | GO:0016301 kinase activity  GO:0031726 CCR1 chemokine receptor binding  GO:0031730 CCR5 chemokine receptor binding  GO:0042056 chemoattractant activity  GO:0042802 identical protein binding  GO:0048020 CCR chemokine receptor binding |
| C-C motif chemokine 4 | GO:0005125 cytokine activity  GO:0005515 protein binding  GO:0008009 chemokine activity  GO:0031726 CCR1 chemokine receptor binding | GO:0031730 CCR5 chemokine receptor binding  GO:0042802 identical protein binding  GO:0048020 CCR chemokine receptor binding |
| C-C motif chemokine 5 | GO:0004435 phosphatidylinositol phospholipase C activity  GO:0004672 protein kinase activity  GO:0005125 cytokine activity  GO:0005515 protein binding  GO:0008009 chemokine activity  GO:0016004 phospholipase activator activity  GO:0030298 receptor signaling protein tyrosine kinase activator activity  GO:0031726 CCR1 chemokine receptor binding  GO:0031729 CCR4 chemokine receptor binding | GO:0031730 CCR5 chemokine receptor binding  GO:0042056 chemoattractant activity  GO:0042379 chemokine receptor binding  GO:0042802 identical protein binding  GO:0042803 protein homodimerization activity  GO:0043621 protein self-association  GO:0046817 chemokine receptor antagonist activity  GO:0048020 CCR chemokine receptor binding |
| C-C motif chemokine 7 | GO:0005125 cytokine activity  GO:0005515 protein binding  GO:0008009 chemokine activity  GO:0008201 heparin binding | GO:0031727 CCR2 chemokine receptor binding  GO:0031726 CCR1 chemokine receptor binding  GO:0048020 CCR chemokine receptor binding |
| C-C motif chemokine 8 | GO:0004672 protein kinase activity  GO:0005125 cytokine activity  GO:0005515 protein binding  GO:0008009 chemokine activity | GO:0008201 heparin binding  GO:0016004 phospholipase activator activity  GO:0048020 CCR chemokine receptor binding |
| C-C motif chemokine 13 | GO:0005102 signaling receptor binding  GO:0005125 cytokine activity  GO:0005515 protein binding | GO:0008009 chemokine activity  GO:0048020 CCR chemokine receptor binding |
| C-C motif chemokine 14 | GO:0005125 cytokine activity  GO:0008009 chemokine activity | GO:0048020 CCR chemokine receptor binding |
| C-C motif chemokine 16 | GO:0005125 cytokine activity  GO:0005515 protein binding  GO:0008009 chemokine activity | GO:0042056 chemoattractant activity  GO:0048020 CCR chemokine receptor binding |
| C-C motif chemokine 18 | GO:0005125 cytokine activity  GO:0005515 protein binding | GO:0008009 chemokine activity  GO:0048020 CCR chemokine receptor binding |
| C-C motif chemokine 19 | GO:0005125 cytokine activity  GO:0005515 protein binding  GO:0008009 chemokine activity  GO:0031732 CCR7 chemokine receptor binding | GO:0031735 CCR10 chemokine receptor binding  GO:0042379 chemokine receptor binding  GO:0048020 CCR chemokine receptor binding |
| C-C motif chemokine 21 | GO:0005125 cytokine activity  GO:0005515 protein binding  GO:0008009 chemokine activity | GO:0031732 CCR7 chemokine receptor binding  GO:0042379 chemokine receptor binding  GO:0048020 CCR chemokine receptor binding |
| C-C motif chemokine 22 | GO:0005125 cytokine activity  GO:0005515 protein binding | GO:0008009 chemokine activity  GO:0048020 CCR chemokine receptor binding |
| C-C motif chemokine 26 | GO:0005125 cytokine activity  GO:0005515 protein binding  GO:0008009 chemokine activity | GO:0031728 CCR3 chemokine receptor binding  GO:0048018 receptor ligand activity  GO:0048020 CCR chemokine receptor binding |
| C-C motif chemokine 27 | GO:0005125 cytokine activity  GO:0005515 protein binding | GO:0008009 chemokine activity  GO:0031728 CCR3 chemokine receptor binding |
| C-C motif chemokine 28 | GO:0005125 cytokine activity  GO:0005515 protein binding | GO:0008009 chemokine activity |
| CD166 antigen | GO:0005102 signaling receptor binding  GO:0005515 protein binding | GO:0042802 identical protein binding |
| CD27 antigen | GO:0004888 transmembrane signaling receptor activity  GO:0005515 protein binding | GO:0043027 cysteine-type endopeptidase inhibitor activity involved in apoptotic process |
| CD44 antigen | GO:0004888 transmembrane signaling receptor activity  GO:0004896 cytokine receptor activity | GO:0005515 protein binding  GO:0005518 collagen binding  GO:0005540 hyaluronic acid binding |
| CD59 glycoprotein | GO:0001848 complement binding | GO:0005515 protein binding |
| CD63 antigen | GO:0005515 protein binding | |
| Cdc42-interacting protein 4 | GO:0005515 protein binding  GO:0008289 lipid binding | GO:0042802 identical protein binding |

| Cell division control protein 42 homolog | GO:0000166 nucleotide binding  GO:0003924 GTPase activity  GO:0005515 protein binding  GO:0005525 GTP binding  GO:0016787 hydrolase activity  GO:0019901 protein kinase binding  GO:0030742 GTP-dependent protein binding | GO:0031435 mitogen-activated protein kinase kinase kinase binding  GO:0031996 thioesterase binding  GO:0032427 GBD domain binding  GO:0034191 apolipoprotein A-I receptor binding  GO:0042802 identical protein binding  GO:0061630 ubiquitin protein ligase activity |
| --- | --- | --- |
| Cerberus | GO:0005125 cytokine activity  GO:0016015 morphogen activity | GO:0036122 BMP binding  GO:0042803 protein homodimerization activity |
| Ceruloplasmin | GO:0004322 ferroxidase activity  GO:0005507 copper ion binding  GO:0016491 oxidoreductase activity | GO:0046872 metal ion binding  GO:0051087 chaperone binding |
| Chitinase-3-like protein 1 | GO:0004568 chitinase activity  GO:0005201 extracellular matrix structural constituent  GO:0005515 protein binding | GO:0008061 chitin binding  GO:0030246 carbohydrate binding |
| Chordin-like protein 2 | GO:0005515 protein binding | |
| Ciliary neurotrophic factor receptor subunit alpha | GO:0004896 cytokine receptor activity  GO:0004897 ciliary neurotrophic factor receptor activity  GO:0005102 signaling receptor binding | GO:0005515 protein binding  GO:0019955 cytokine binding |
| Ciliary neurotrophic factor | GO:0005125 cytokine activity  GO:0005127 ciliary neurotrophic factor receptor binding  GO:0005138 interleukin-6 receptor binding | GO:0005515 protein binding  GO:0008083 growth factor activity  GO:0044877 protein-containing complex binding |
| Clusterin | GO:0001540 amyloid-beta binding  GO:0005102 signaling receptor binding  GO:0005515 protein binding  GO:0016887 ATPase activity  GO:0031625 ubiquitin protein ligase binding  GO:0044877 protein-containing complex binding  GO:0046982 protein heterodimerization activity | GO:0048156 tau protein binding  GO:0050750 low-density lipoprotein particle receptor binding  GO:0051082 unfolded protein binding  GO:0051087 chaperone binding  GO:0051787 misfolded protein binding |
| Coagulation factor XIII A chain | GO:0003810 protein-glutamine gamma-glutamyltransferase activity  GO:0005515 protein binding  GO:0016740 transferase activity | GO:0016746 transferase activity, transferring acyl groups  GO:0046872 metal ion binding |
| Coagulation factor XIII B chain | - | |
| Collagen alpha-1(I) chain | GO:0002020 protease binding  GO:0005201 extracellular matrix structural constituent  GO:0005515 protein binding | GO:0030020 extracellular matrix structural constituent conferring tensile strength  GO:0042802 identical protein binding  GO:0046872 metal ion binding  GO:0048407 platelet-derived growth factor binding |
| Collagen alpha-1(III) chain | GO:0002020 protease binding  GO:0005178 integrin binding  GO:0005201 extracellular matrix structural constituent  GO:0005515 protein binding | GO:0030020 extracellular matrix structural constituent conferring tensile strength  GO:0046332 SMAD binding  GO:0046872 metal ion binding  GO:0048407 platelet-derived growth factor binding |
| Collagen alpha-1(IV) chain | GO:0005201 extracellular matrix structural constituent  GO:0005515 protein binding | GO:0030020 extracellular matrix structural constituent conferring tensile strength  GO:0048407 platelet-derived growth factor binding |
| Collagen alpha-1(V) chain | GO:0005178 integrin binding  GO:0005201 extracellular matrix structural constituent  GO:0005515 protein binding  GO:0008201 heparin binding | GO:0030020 extracellular matrix structural constituent conferring tensile strength  GO:0043394 proteoglycan binding  GO:0046872 metal ion binding  GO:0048407 platelet-derived growth factor binding |
| Collagen alpha-1(VI) chain | GO:0005518 collagen binding  GO:0030020 extracellular matrix structural constituent conferring tensile strength | GO:0048407 platelet-derived growth factor binding |
| Collagen alpha-1(VII) chain | GO:0004867 serine-type endopeptidase inhibitor activity  GO:0005515 protein binding | GO:0030020 extracellular matrix structural constituent conferring tensile strength  GO:0030414 peptidase inhibitor activity |
| Collagen alpha-1(XII) chain | GO:0030020 extracellular matrix structural constituent conferring tensile strength | |
| Collagen alpha-1(XV) chain | GO:0005198 structural molecule activity  GO:0005201 extracellular matrix structural constituent | GO:0030020 extracellular matrix structural constituent conferring tensile strength |
| Collagen alpha-2(I) chain | GO:0002020 protease binding  GO:0005201 extracellular matrix structural constituent  GO:0005515 protein binding  GO:0030020 extracellular matrix structural constituent conferring tensile strength | GO:0030674 protein-macromolecule adaptor activity  GO:0042802 identical protein binding  GO:0046332 SMAD binding  GO:0046872 metal ion binding  GO:0048407 platelet-derived growth factor binding |
| Collagen alpha-2(IV) chain | GO:0005201 extracellular matrix structural constituent  GO:0005515 protein binding | GO:0030020 extracellular matrix structural constituent conferring tensile strength |
| Collagen alpha-2(V) chain | GO:0005201 extracellular matrix structural constituent  GO:0030020 extracellular matrix structural constituent conferring tensile strength | GO:0046332 SMAD binding  GO:0046872 metal ion binding |
| Collagen alpha-2(VI) chain | GO:0005515 protein binding  GO:0005518 collagen binding | GO:0030020 extracellular matrix structural constituent conferring tensile strength |
| Collagen alpha-3(VI) chain | GO:0004867 serine-type endopeptidase inhibitor activity  GO:0030020 extracellular matrix structural constituent conferring tensile strength | GO:0030414 peptidase inhibitor activity |
| Collagenase 3 | GO:0004175 endopeptidase activity  GO:0004222 metalloendopeptidase activity  GO:0005509 calcium ion binding  GO:0005518 collagen binding  GO:0008233 peptidase activity | GO:0008237 metallopeptidase activity  GO:0008270 zinc ion binding  GO:0016787 hydrolase activity  GO:0046872 metal ion binding |
| Complement C2 | GO:0004252 serine-type endopeptidase activity  GO:0005515 protein binding  GO:0008233 peptidase activity | GO:0008236 serine-type peptidase activity  GO:0016787 hydrolase activity  GO:0046872 metal ion binding |
| Complement C3 | GO:0004866 endopeptidase inhibitor activity  GO:0005102 signaling receptor binding  GO:0005515 protein binding | GO:0031715 C5L2 anaphylatoxin chemotactic receptor binding |
| Complement C5 | GO:0004866 endopeptidase inhibitor activity  GO:0005102 signaling receptor binding | GO:0005515 protein binding  GO:0008009 chemokine activity |
| Complement factor H-related protein 2 | GO:0005515 protein binding | GO:0042802 identical protein binding |
| Corticosteroid 11-beta-dehydrogenase isozyme 1 | GO:0003845 11-beta-hydroxysteroid dehydrogenase [NAD(P)] activity  GO:0005496 steroid binding  GO:0016491 oxidoreductase activity | GO:0042803 protein homodimerization activity  GO:0050661 NADP binding  GO:0070524 11-beta-hydroxysteroid dehydrogenase (NADP+) activity |
| Corticosteroid-binding globulin | GO:0004867 serine-type endopeptidase inhibitor activity  GO:0005496 steroid binding | GO:0008289 lipid binding |
| C-reactive protein | GO:0001849 complement component C1q complex binding  GO:0005509 calcium ion binding  GO:0005515 protein binding  GO:0030169 low-density lipoprotein particle binding  GO:0033265 choline binding | GO:0042802 identical protein binding  GO:0046790 virion binding  GO:0046872 metal ion binding  GO:0050750 low-density lipoprotein particle receptor binding |
| Creatine kinase B-type | GO:0000166 nucleotide binding  GO:0003824 catalytic activity  GO:0004111 creatine kinase activity  GO:0005515 protein binding  GO:0005524 ATP binding | GO:0016301 kinase activity  GO:0016740 transferase activity  GO:0016772 transferase activity, transferring phosphorus-containing groups  GO:0031625 ubiquitin protein ligase binding |
| CREB-binding protein | GO:0001085 RNA polymerase II transcription factor binding  GO:0001102 RNA polymerase II activating transcription factor binding  GO:0002039 p53 binding  GO:0003682 chromatin binding  GO:0003684 damaged DNA binding  GO:0003712 transcription coregulator activity  GO:0003713 transcription coactivator activity  GO:0003714 transcription corepressor activity  GO:0004402 histone acetyltransferase activity  GO:0005515 protein binding | GO:0008134 transcription factor binding  GO:0008270 zinc ion binding  GO:0016407 acetyltransferase activity  GO:0016740 transferase activity  GO:0016746 transferase activity, transferring acyl groups  GO:0031490 chromatin DNA binding  GO:0043426 MRF binding  GO:0046872 metal ion binding  GO:0061733 peptide-lysine-N-acetyltransferase activity |
| Cryptic protein | GO:0003674 molecular_function  GO:0005102 signaling receptor binding | GO:0038100 nodal binding  GO:0070697 activin receptor binding |
| C-X-C chemokine receptor type 6 | GO:0004930 G protein-coupled receptor activity  GO:0004950 chemokine receptor activity  GO:0015026 coreceptor activity | GO:0016493 C-C chemokine receptor activity  GO:0016494 C-X-C chemokine receptor activity  GO:0019957 C-C chemokine binding |
| C-X-C motif chemokine 2 | GO:0005125 cytokine activity  GO:0005515 protein binding | GO:0008009 chemokine activity  GO:0045236 CXCR chemokine receptor binding |
| C-X-C motif chemokine 5 | GO:0005125 cytokine activity  GO:0005515 protein binding  GO:0008009 chemokine activity | GO:0042802 identical protein binding  GO:0045236 CXCR chemokine receptor binding |
| C-X-C motif chemokine 9 | GO:0005125 cytokine activity  GO:0005515 protein binding  GO:0008009 chemokine activity | GO:0045236 CXCR chemokine receptor binding  GO:0048248 CXCR3 chemokine receptor binding |
| C-X-C motif chemokine 10 | GO:0005102 signaling receptor binding  GO:0005125 cytokine activity  GO:0005515 protein binding  GO:0008009 chemokine activity  GO:0008201 heparin binding | GO:0008603 cAMP-dependent protein kinase regulator activity  GO:0042056 chemoattractant activity  GO:0045236 CXCR chemokine receptor binding  GO:0048248 CXCR3 chemokine receptor binding |
| C-X-C motif chemokine 11 | GO:0005125 cytokine activity  GO:0005515 protein binding  GO:0008009 chemokine activity | GO:0008201 heparin binding  GO:0045236 CXCR chemokine receptor binding GO:0048248 CXCR3 chemokine receptor binding |
| C-X-C motif chemokine 16 | GO:0005041 low-density lipoprotein particle receptor activity  GO:0005044 scavenger receptor activity | GO:0005125 cytokine activity  GO:0008009 chemokine activity |
| Cyclin-dependent kinase inhibitor 1 | GO:0004860 protein kinase inhibitor activity  GO:0004861 cyclin-dependent protein serine/threonine kinase inhibitor activity  GO:0005515 protein binding  GO:0019901 protein kinase binding  GO:0019912 cyclin-dependent protein kinase activating kinase activity | GO:0030332 cyclin binding  GO:0031625 ubiquitin protein ligase binding  GO:0044877 protein-containing complex binding  GO:0046872 metal ion binding  GO:0140311 protein sequestering activity |
| Cystatin A | GO:0002020 protease binding  GO:0004866 endopeptidase inhibitor activity  GO:0004869 cysteine-type endopeptidase inhibitor activity | GO:0005515 protein binding  GO:0030414 peptidase inhibitor activity |
| Cytokine receptor common subunit gamma | GO:0004896 cytokine receptor activity  GO:0004911 interleukin-2 receptor activity  GO:0004913 interleukin-4 receptor activity  GO:0004917 interleukin-7 receptor activity | GO:0005515 protein binding  GO:0019955 cytokine binding  GO:0019976 interleukin-2 binding  GO:0042010 interleukin-15 receptor activity |
| Cytoplasmic tyrosine-protein kinase BMX | GO:0000166 nucleotide binding  GO:0004672 protein kinase activity  GO:0004713 protein tyrosine kinase activity  GO:0004714 transmembrane receptor protein tyrosine kinase activity  GO:0004715 non-membrane spanning protein tyrosine kinase activity | GO:0005515 protein binding  GO:0005524 ATP binding  GO:0016301 kinase activity  GO:0016740 transferase activity  GO:0046872 metal ion binding |
| Cytotoxic and regulatory T-cell molecule | GO:0005102 signaling receptor binding  GO:0005515 protein binding | GO:0042802 identical protein binding |
| Cytotoxic T-lymphocyte protein 4 | GO:0005515 protein binding | |
| DAN domain family member 5 | GO:0016015 morphogen activity | |
| Decorin | GO:0003723 RNA binding  GO:0005515 protein binding  GO:0005518 collagen binding  GO:0005539 glycosaminoglycan binding | GO:0030021 extracellular matrix structural constituent conferring compression resistance  GO:0047485 protein N-terminus binding  GO:0050840 extracellular matrix binding |
| Dentin matrix acidic phosphoprotein 1 | GO:0005178 integrin binding  GO:0005509 calcium ion binding | GO:0050840 extracellular matrix binding |
| Dermcidin | GO:0003723 RNA binding  GO:0005515 protein binding | GO:0008233 peptidase activity  GO:0016787 hydrolase activity |
| Dickkopf-related protein 1 | GO:0005515 protein binding  GO:0008083 growth factor activity  GO:0039706 co-receptor binding | GO:0048019 receptor antagonist activity  GO:0050750 low-density lipoprotein particle receptor binding |
| Dickkopf-related protein 3 | GO:0005515 protein binding  GO:0039706 co-receptor binding | GO:0048019 receptor antagonist activity |
| Dickkopf-related protein 4 | GO:0003674 molecular_function  GO:0005515 protein binding | GO:0039706 co-receptor binding  GO:0048019 receptor antagonist activity |
| Discoidin domain-containing receptor 2 | GO:0000166 nucleotide binding  GO:0004672 protein kinase activity  GO:0004713 protein tyrosine kinase activity  GO:0004714 transmembrane receptor protein tyrosine kinase activity  GO:0005515 protein binding | GO:0005518 collagen binding  GO:0005524 ATP binding  GO:0016301 kinase activity  GO:0016740 transferase activity  GO:0038062 protein tyrosine kinase collagen receptor activity |
| Discoidin, CUB and LCCL domain-containing protein 2 | GO:0005515 protein binding | |
| Echinoderm microtubule-associated protein-like 2 | GO:0005102 signaling receptor binding  GO:0005515 protein binding  GO:0008017 microtubule binding | GO:0008022 protein C-terminus binding  GO:0015631 tubulin binding |
| Ectodysplasin-A | GO:0005102 signaling receptor binding  GO:0005123 death receptor binding  GO:0005164 tumor necrosis factor receptor binding | GO:0005515 protein binding  GO:0038177 death receptor agonist activity |
| Ectonucleotide pyrophosphatase/ phosphodiesterase family member 2 | GO:0003676 nucleic acid binding  GO:0003824 catalytic activity  GO:0004528 phosphodiesterase I activity  GO:0004551 nucleotide diphosphatase activity  GO:0004622 lysophospholipase activity  GO:0005044 scavenger receptor activity  GO:0005509 calcium ion binding | GO:0008134 transcription factor binding  GO:0008270 zinc ion binding  GO:0016787 hydrolase activity  GO:0030247 polysaccharide binding  GO:0046872 metal ion binding  GO:0047391 alkylglycerophosphoethanolamine phosphodiesterase activity |
| EGF-like repeat and discoidin I-like domain-containing protein 3 | GO:0005178 integrin binding  GO:0005201 extracellular matrix structural constituent | GO:0005509 calcium ion binding  GO:0046872 metal ion binding |
| Elongation factor 1-alpha 1 | GO:0000049 tRNA binding  GO:0000166 nucleotide binding  GO:0003723 RNA binding  GO:0003746 translation elongation factor activity GO:0003924 GTPase activity | GO:0005515 protein binding  GO:0005525 GTP binding  GO:0019901 protein kinase binding  GO:0019900 kinase binding |
| Elongation factor 2 | GO:0000166 nucleotide binding  GO:0002039 p53 binding  GO:0003723 RNA binding  GO:0003746 translation elongation factor activity  GO:0003924 GTPase activity  GO:0043022 ribosome binding | GO:0051015 actin filament binding  GO:0005515 protein binding  GO:0005525 GTP binding  GO:0008097 5S rRNA binding  GO:0019901 protein kinase binding  GO:0045296 cadherin binding |
| Embryonic growth/differentiation factor 1 | GO:0005125 cytokine activity | GO:0008083 growth factor activity |
| Endoglin | GO:0004888 transmembrane signaling receptor activity  GO:0005024 transforming growth factor beta-activated receptor activity  GO:0005114 type II transforming growth factor beta receptor binding  GO:0005515 protein binding  GO:0005534 galactose binding  GO:0005539 glycosaminoglycan binding  GO:0015026 coreceptor activity | GO:0034713 type I transforming growth factor beta receptor binding  GO:0036122 BMP binding  GO:0042802 identical protein binding  GO:0042803 protein homodimerization activity  GO:0048185 activin binding  GO:0050431 transforming growth factor beta binding |
| Endostatin (cleaved from Collagen alpha-1(XVIII) chain) | GO:0005201 extracellular matrix structural constituent  GO:0005515 protein binding | GO:0030020 extracellular matrix structural constituent conferring tensile strength  GO:0042802 identical protein binding  GO:0046872 metal ion binding |
| Endothelial cell-selective adhesion molecule | GO:0005515 protein binding | GO:0098632 cell-cell adhesion mediator activity |
| Endothelin-1 receptor | GO:0004435 phosphatidylinositol phospholipase C activity  GO:0004930 G protein-coupled receptor activity | GO:0004962 endothelin receptor activity  GO:0005515 protein binding |
| Eotaxin | GO:0005125 cytokine activity  GO:0005515 protein binding  GO:0008009 chemokine activity  GO:0031728 CCR3 chemokine receptor binding | GO:0046983 protein dimerization activity  GO:0048018 receptor ligand activity  GO:0048020 CCR chemokine receptor binding |
| Ephrin type-A receptor 4 | GO:0000166 nucleotide binding  GO:0001540 amyloid-beta binding  GO:0004672 protein kinase activity  GO:0004713 protein tyrosine kinase activity  GO:0004714 transmembrane receptor protein tyrosine kinase activity  GO:0005003 ephrin receptor activity  GO:0005004 GPI-linked ephrin receptor activity  GO:0005005 transmembrane-ephrin receptor activity  GO:0005515 protein binding | GO:0005524 ATP binding  GO:0016301 kinase activity  GO:0016740 transferase activity  GO:0042731 PH domain binding  GO:0042802 identical protein binding  GO:0046875 ephrin receptor binding  GO:0097161 DH domain binding  GO:1990782 protein tyrosine kinase binding |
| Ephrin type-A receptor 6 | GO:0000166 nucleotide binding  GO:0003674 molecular_function  GO:0004672 protein kinase activity  GO:0004713 protein tyrosine kinase activity  GO:0004714 transmembrane receptor protein tyrosine kinase activity | GO:0005003 ephrin receptor activity  GO:0005005 transmembrane-ephrin receptor activity  GO:0005524 ATP binding  GO:0016301 kinase activity  GO:0016740 transferase activity |
| Ephrin type-A receptor 8 | GO:0000166 nucleotide binding  GO:0004672 protein kinase activity  GO:0004713 protein tyrosine kinase activity  GO:0004714 transmembrane receptor protein tyrosine kinase activity  GO:0005003 ephrin receptor activity  GO:0005004 GPI-linked ephrin receptor activity | GO:0005005 transmembrane-ephrin receptor activity  GO:0005524 ATP binding  GO:0016301 kinase activity  GO:0016740 transferase activity  GO:0019838 growth factor binding |
| Ephrin type-B receptor 4 | GO:0000166 nucleotide binding  GO:0004672 protein kinase activity  GO:0004713 protein tyrosine kinase activity  GO:0004714 transmembrane receptor protein tyrosine kinase activity  GO:0005003 ephrin receptor activity | GO:0005005 transmembrane-ephrin receptor activity  GO:0005515 protein binding  GO:0005524 ATP binding  GO:0016301 kinase activity  GO:0016740 transferase activity |
| Epidermal growth factor receptor | GO:0000166 nucleotide binding  GO:0001618 virus receptor activity  GO:0003682 chromatin binding  GO:0003690 double-stranded DNA binding  GO:0004672 protein kinase activity  GO:0004709 MAP kinase kinase kinase activity  GO:0004713 protein tyrosine kinase activity  GO:0004714 transmembrane receptor protein tyrosine kinase activity  GO:0004888 transmembrane signaling receptor activity  GO:0005006 epidermal growth factor-activated receptor activity  GO:0042802 identical protein binding  GO:0044877 protein-containing complex binding  GO:0045296 cadherin binding | GO:0051015 actin filament binding  GO:0051117 ATPase binding  GO:0005102 signaling receptor binding  GO:0005178 integrin binding  GO:0005515 protein binding  GO:0005516 calmodulin binding  GO:0005524 ATP binding  GO:0016301 kinase activity  GO:0016740 transferase activity  GO:0019899 enzyme binding  GO:0019901 protein kinase binding  GO:0019903 protein phosphatase binding  GO:0030235 nitric-oxide synthase regulator activity  GO:0031625 ubiquitin protein ligase binding  GO:0048408 epidermal growth factor binding |
| Epidermal growth factor receptor substrate 15-like 1 | GO:0005509 calcium ion binding  GO:0005515 protein binding | GO:0045296 cadherin binding  GO:0046872 metal ion binding |
| Epithelial cell adhesion molecule | GO:0005515 protein binding  GO:0044877 protein-containing complex binding | GO:0098641 cadherin binding involved in cell-cell adhesion |
| Erythropoietin | GO:0005125 cytokine activity  GO:0005128 erythropoietin receptor binding  GO:0005179 hormone activity | GO:0005515 protein binding  GO:0030295 protein kinase activator activity |
| Erythropoietin receptor | GO:0004888 transmembrane signaling receptor activity  GO:0004896 cytokine receptor activity  GO:0004900 erythropoietin receptor activity | GO:0005515 protein binding  GO:0042802 identical protein binding |
| E-Selectin | GO:0004888 transmembrane signaling receptor activity  GO:0005515 protein binding  GO:0030246 carbohydrate binding  GO:0033691 sialic acid binding | GO:0043274 phospholipase binding  GO:0046872 metal ion binding  GO:0070492 oligosaccharide binding |
| EVI5-like protein | GO:0005096 GTPase activator activity  GO:0005515 protein binding | GO:0031267 small GTPase binding |
| FAS-associated death domain protein | GO:0002020 protease binding  GO:0005123 death receptor binding  GO:0005164 tumor necrosis factor receptor binding  GO:0005515 protein binding  GO:0032813 tumor necrosis factor receptor superfamily binding | GO:0033612 receptor serine/threonine kinase binding  GO:0035877 death effector domain binding  GO:0042802 identical protein binding  GO:0044877 protein-containing complex binding  GO:0089720 caspase binding |
| Fatty acid-binding protein 5 | GO:0001972 retinoic acid binding  GO:0005324 long-chain fatty acid transporter activity  GO:0005504 fatty acid binding | GO:0005515 protein binding  GO:0008289 lipid binding  GO:0042802 identical protein binding |
| Ferritin light chain | GO:0005506 iron ion binding  GO:0005515 protein binding  GO:0008198 ferrous iron binding | GO:0008199 ferric iron binding  GO:0042802 identical protein binding  GO:0046872 metal ion binding |
| Fetuin-B | GO:0003674 molecular_function  GO:0004857 enzyme inhibitor activity  GO:0004866 endopeptidase inhibitor activity  GO:0004869 cysteine-type endopeptidase inhibitor activity | GO:0005515 protein binding  GO:0008191 metalloendopeptidase inhibitor activity  GO:0030414 peptidase inhibitor activity |
| Fibrinogen-like protein 1 | GO:0005102 signaling receptor binding | GO:0005515 protein binding |
| Fibrinopeptide A (cleaved from Fibrinogen alpha chain) | GO:0005102 signaling receptor binding  GO:0005198 structural molecule activity  GO:0005201 extracellular matrix structural constituent | GO:0005515 protein binding  GO:0046872 metal ion binding  GO:0050839 cell adhesion molecule binding |
| Fibroblast growth factor 2 | GO:0005104 fibroblast growth factor receptor binding  GO:0005125 cytokine activity  GO:0005178 integrin binding  GO:0005515 protein binding  GO:0008083 growth factor activity  GO:0008201 heparin binding | GO:0019956 chemokine binding  GO:0030374 nuclear receptor transcription coactivator activity  GO:0042056 chemoattractant activity  GO:0042802 identical protein binding  GO:0090722 receptor-receptor interaction |
| Fibroblast growth factor 4 | GO:0005104 fibroblast growth factor receptor binding | GO:0008083 growth factor activity  GO:0008201 heparin binding |
| Fibroblast growth factor 5 | GO:0005104 fibroblast growth factor receptor binding | GO:0008083 growth factor activity |
| Fibroblast growth factor 6 | GO:0005104 fibroblast growth factor receptor binding  GO:0005515 protein binding | GO:0008083 growth factor activity |
| Fibroblast growth factor 8 | GO:0005104 fibroblast growth factor receptor binding  GO:0005105 type 1 fibroblast growth factor receptor binding  GO:0005111 type 2 fibroblast growth factor receptor binding | GO:0008083 growth factor activity  GO:0042056 chemoattractant activity |
| Fibroblast growth factor 10 | GO:0005104 fibroblast growth factor receptor binding  GO:0005111 type 2 fibroblast growth factor receptor binding  GO:0005515 protein binding | GO:0008083 growth factor activity  GO:0008201 heparin binding  GO:0042056 chemoattractant activity |
| Fibroblast growth factor 11 | GO:0005515 protein binding  GO:0008083 growth factor activity | GO:0017080 sodium channel regulator activity |
| Fibroblast growth factor 12 | GO:0005104 fibroblast growth factor receptor binding  GO:0005515 protein binding  GO:0008083 growth factor activity | GO:0008201 heparin binding  GO:0017080 sodium channel regulator activity  GO:0044325 ion channel binding |
| Fibroblast growth factor 13 | GO:0005515 protein binding  GO:0008017 microtubule binding  GO:0008083 growth factor activity  GO:0017080 sodium channel regulator activity | GO:0030295 protein kinase activator activity  GO:0044325 ion channel binding  GO:0048487 beta-tubulin binding |
| Fibroblast growth factor 16 | GO:0005104 fibroblast growth factor receptor binding  GO:0005515 protein binding | GO:0008083 growth factor activity |
| Fibroblast growth factor 17 | GO:0005104 fibroblast growth factor receptor binding  GO:0005105 type 1 fibroblast growth factor receptor binding  GO:0005111 type 2 fibroblast growth factor receptor binding | GO:0005515 protein binding  GO:0008083 growth factor activity |
| Fibroblast growth factor 18 | GO:0005104 fibroblast growth factor receptor binding  GO:0005105 type 1 fibroblast growth factor receptor binding | GO:0005111 type 2 fibroblast growth factor receptor binding  GO:0008083 growth factor activity |

| Fibroblast growth factor 20 | GO:0005102 signaling receptor binding  GO:0005104 fibroblast growth factor receptor binding  GO:0008083 growth factor activity | GO:0043395 heparan sulfate proteoglycan binding  GO:0090722 receptor-receptor interaction |
| --- | --- | --- |
| Fibroblast growth factor 21 | GO:0005104 fibroblast growth factor receptor binding  GO:0005515 protein binding | GO:0008083 growth factor activity |
| Fibroblast growth factor receptor 1 | GO:0000166 nucleotide binding  GO:0004672 protein kinase activity  GO:0004713 protein tyrosine kinase activity  GO:0004714 transmembrane receptor protein  GO:0005007 fibroblast growth factor-activated receptor activity  GO:0005515 protein binding  GO:0005524 ATP binding  GO:0008201 heparin binding | GO:0016301 kinase activity  GO:0016740 transferase activity  GO:0017134 fibroblast growth factor binding  tyrosine kinase activity  GO:0042169 SH2 domain binding  GO:0042802 identical protein binding  GO:0042803 protein homodimerization activity  GO:0090722 receptor-receptor interaction |
| Fibroblast growth factor receptor 3 | GO:0000166 nucleotide binding  GO:0004672 protein kinase activity  GO:0004713 protein tyrosine kinase activity  GO:0004714 transmembrane receptor protein tyrosine kinase activity  GO:0005007 fibroblast growth factor-activated receptor activity  GO:0005515 protein binding | GO:0005524 ATP binding  GO:0016301 kinase activity  GO:0016740 transferase activity  GO:0017134 fibroblast growth factor binding  GO:0042802 identical protein binding |
| Fibroblast growth factor receptor 4 | GO:0000166 nucleotide binding  GO:0004713 protein tyrosine kinase activity  GO:0004714 transmembrane receptor protein tyrosine kinase activity  GO:0004672 protein kinase activity  GO:0005007 fibroblast growth factor-activated receptor activity | GO:0005515 protein binding  GO:0005524 ATP binding  GO:0008201 heparin binding  GO:0016301 kinase activity  GO:0016740 transferase activity  GO:0017134 fibroblast growth factor binding |
| Fibroblast growth factor-binding protein 1 | GO:0005515 protein binding  GO:0008201 heparin binding | GO:0017134 fibroblast growth factor binding  GO:0019838 growth factor binding |
| Fibronectin | GO:0002020 protease binding  GO:0005102 signaling receptor binding  GO:0005178 integrin binding  GO:0005515 protein binding  GO:0005518 collagen binding  GO:0005201 extracellular matrix structural constituent  GO:0008022 protein C-terminus binding | GO:0008201 heparin binding  GO:0016504 peptidase activator activity  GO:0019899 enzyme binding  GO:0042802 identical protein binding  GO:0043394 proteoglycan binding  GO:0051087 chaperone binding  GO:0097718 disordered domain specific binding |
| Filaggrin-2 | GO:0005198 structural molecule activity  GO:0005509 calcium ion binding | GO:0046872 metal ion binding  GO:0046914 transition metal ion binding |
| Follistatin | GO:0005515 protein binding  GO:0038102 activin receptor antagonist activity | GO:0043395 heparan sulfate proteoglycan binding  GO:0048185 activin binding |
| Follistatin-related protein 3 | GO:0001968 fibronectin binding  GO:0005515 protein binding | GO:0048185 activin binding |
| Forkhead box protein N3 | GO:0000981 DNA-binding transcription factor activity, RNA polymerase II-specific  GO:0000987 cis-regulatory region sequence-specific DNA binding  GO:0003677 DNA binding  GO:0003700 DNA-binding transcription factor activity | GO:0005515 protein binding  GO:0008022 protein C-terminus binding  GO:0043565 sequence-specific DNA binding |
| Frizzled-1 | GO:0004888 transmembrane signaling receptor activity  GO:0004930 G protein-coupled receptor activity  GO:0005102 signaling receptor binding  GO:0005109 frizzled binding | GO:0005515 protein binding  GO:0017147 Wnt-protein binding  GO:0030165 PDZ domain binding  GO:0042813 Wnt-activated receptor activity |
| Frizzled-3 | GO:0004888 transmembrane signaling receptor activity  GO:0004930 G protein-coupled receptor activity  GO:0005515 protein binding | GO:0017147 Wnt-protein binding  GO:0030165 PDZ domain binding  GO:0042813 Wnt-activated receptor activity |
| Frizzled-6 | GO:0001540 amyloid-beta binding  GO:0004888 transmembrane signaling receptor activity  GO:0004930 G protein-coupled receptor activity | GO:0005515 protein binding  GO:0017147 Wnt-protein binding  GO:0031625 ubiquitin protein ligase binding GO:0042813 Wnt-activated receptor activity |
| Frizzled-7 | GO:0004888 transmembrane signaling receptor activity  GO:0004930 G protein-coupled receptor activity  GO:0005109 frizzled binding  GO:0005515 protein binding | GO:0005546 phosphatidylinositol-4,5-bisphosphate binding  GO:0017147 Wnt-protein binding  GO:0030165 PDZ domain binding  GO:0042813 Wnt-activated receptor activity |
| Fructose-bisphosphate aldolase A | GO:0003723 RNA binding  GO:0003779 actin binding  GO:0003824 catalytic activity  GO:0004332 fructose-bisphosphate aldolase activity  GO:0005515 protein binding  GO:0008092 cytoskeletal protein binding | GO:0015631 tubulin binding  GO:0016829 lyase activity  GO:0042802 identical protein binding  GO:0045296 cadherin binding  GO:0070061 fructose binding |

| Fructose-bisphosphate aldolase B | GO:0003824 catalytic activity  GO:0004332 fructose-bisphosphate aldolase activity  GO:0005515 protein binding  GO:0008092 cytoskeletal protein binding  GO:0016829 lyase activity | GO:0042802 identical protein binding  GO:0051117 ATPase binding  GO:0061609 fructose-1-phosphate aldolase activity  GO:0070061 fructose binding |
| --- | --- | --- |
| Fructose-bisphosphate aldolase C | GO:0003824 catalytic activity  GO:0004332 fructose-bisphosphate aldolase activity  GO:0005515 protein binding | GO:0008092 cytoskeletal protein binding  GO:0016829 lyase activity |
| Furin | GO:0002020 protease binding  GO:0004175 endopeptidase activity  GO:0004252 serine-type endopeptidase activity  GO:0004867 serine-type endopeptidase inhibitor activity  GO:0005515 protein binding  GO:0008233 peptidase activity | GO:0008236 serine-type peptidase activity  GO:0016787 hydrolase activity  GO:0042277 peptide binding  GO:0046872 metal ion binding  GO:0048406 nerve growth factor binding |
| Galanin peptides | GO:0004966 galanin receptor activity  GO:0005179 hormone activity  GO:0005184 neuropeptide hormone activity  GO:0005515 protein binding | GO:0031763 galanin receptor binding  GO:0031764 type 1 galanin receptor binding  GO:0031765 type 2 galanin receptor binding  GO:0031766 type 3 galanin receptor binding |
| Galectin-10 | GO:0004622 lysophospholipase activity  GO:0005515 protein binding  GO:0030246 carbohydrate binding | GO:0042802 identical protein binding  GO:0097153 cysteine-type endopeptidase activity involved in apoptotic process |
| Galectin-3 | GO:0003723 RNA binding  GO:0004864 protein phosphatase inhibitor activity  GO:0005515 protein binding  GO:0019863 IgE binding  GO:0019903 protein phosphatase binding | GO:0030246 carbohydrate binding  GO:0042056 chemoattractant activity  GO:0043236 laminin binding  GO:0070492 oligosaccharide binding |
| Gamma-Thrombin (cleaved from prothrombin) | GO:0001530 lipopolysaccharide binding  GO:0004252 serine-type endopeptidase activity  GO:0005102 signaling receptor binding  GO:0005509 calcium ion binding  GO:0005515 protein binding  GO:0008047 enzyme activator activity | GO:0008083 growth factor activity  GO:0008201 heparin binding  GO:0008233 peptidase activity  GO:0008236 serine-type peptidase activity  GO:0016787 hydrolase activity  GO:0070053 thrombospondin receptor activity |
| GATA-type zinc finger protein 1 | GO:0003677 DNA binding  GO:0008270 zinc ion binding | GO:0043565 sequence-specific DNA binding  GO:0046872 metal ion binding |
| GDNF family receptor alpha-3 | GO:0005102 signaling receptor binding  GO:0005515 protein binding  GO:0008046 axon guidance receptor activity | GO:0016167 glial cell-derived neurotrophic factor receptor activity  GO:0038023 signaling receptor activity |
| Geminin | GO:0003682 chromatin binding  GO:0003714 transcription corepressor activity  GO:0005515 protein binding | GO:0042826 histone deacetylase binding  GO:0070491 repressing transcription factor binding |
| Glial cell line-derived neurotrophic factor | GO:0005102 signaling receptor binding  GO:0005515 protein binding  GO:0008083 growth factor activity  GO:0030116 glial cell-derived neurotrophic factor receptor binding | GO:0030971 receptor tyrosine kinase binding  GO:0042803 protein homodimerization activity  GO:1902379 chemoattractant activity involved in axon guidance |
| Glutathione peroxidase 1 | GO:0004601 peroxidase activity  GO:0004602 glutathione peroxidase activity  GO:0016491 oxidoreductase activity | GO:0017124 SH3 domain binding  GO:0047066 phospholipid-hydroperoxide glutathione peroxidase activity |
| Glutathione peroxidase 3 | GO:0004601 peroxidase activity  GO:0004602 glutathione peroxidase activity  GO:0005515 protein binding  GO:0008134 transcription factor binding | GO:0008430 selenium binding  GO:0016491 oxidoreductase activity  GO:0042802 identical protein binding |
| Glyceraldehyde 3-phosphate dehydrogenase | GO:0004365 glyceraldehyde-3-phosphate dehydrogenase (NAD+) (phosphorylating) activity  GO:0005515 protein binding  GO:0008017 microtubule binding  GO:0016491 oxidoreductase activity  GO:0016620 oxidoreductase activity, acting on the aldehyde or oxo group of donors, NAD or NADP as acceptor | GO:0016740 transferase activity  GO:0019828 aspartic-type endopeptidase inhibitor activity  GO:0035605 peptidyl-cysteine S-nitrosylase activity  GO:0042802 identical protein binding GO:0050661 NADP binding  GO:0051287 NAD binding  GO:0097718 disordered domain specific binding |
| Glycogen phosphorylase, brain form | GO:0003824 catalytic activity  GO:0004645 1,4-alpha-oligoglucan phosphorylase activity  GO:0005515 protein binding  GO:0008184 glycogen phosphorylase activity  GO:0016740 transferase activity | GO:0016757 transferase activity, transferring glycosyl groups  GO:0030170 pyridoxal phosphate binding  GO:0102250 linear malto-oligosaccharide phosphorylase activity  GO:0102499 SHG alpha-glucan phosphorylase activity |
| Glycoprotein hormones alpha chain | GO:0005179 hormone activity  GO:0005515 protein binding | GO:0016913 follicle-stimulating hormone activity |
| Glypican-3 | GO:0005515 protein binding  GO:0030414 peptidase inhibitor activity | GO:0060422 peptidyl-dipeptidase inhibitor activity |
| Glypican-5 | GO:0005515 protein binding | |
| Granulocyte colony-stimulating factor | GO:0005125 cytokine activity  GO:0005130 granulocyte colony-stimulating factor receptor binding | GO:0008083 growth factor activity  GO:0019899 enzyme binding |
| Granulocyte-macrophage colony-stimulating factor receptor subunit alpha | GO:0004896 cytokine receptor activity  GO:0005515 protein binding | GO:0019955 cytokine binding  GO:0038023 signaling receptor activity |
| Granulocyte-macrophage colony-stimulating factor | GO:0005125 cytokine activity  GO:0005129 granulocyte macrophage colony-stimulating factor receptor binding | GO:0005515 protein binding  GO:0008083 growth factor activity |
| Granzyme A | GO:0004252 serine-type endopeptidase activity  GO:0005515 protein binding  GO:0008233 peptidase activity | GO:0008236 serine-type peptidase activity  GO:0016787 hydrolase activity  GO:0042803 protein homodimerization activity |
| Gremlin-1 | GO:0005125 cytokine activity  GO:0005515 protein binding  GO:0016015 morphogen activity  GO:0030297 transmembrane receptor protein tyrosine kinase activator activity | GO:0036122 BMP binding  GO:0042803 protein homodimerization activity  GO:0043184 vascular endothelial growth factor receptor 2 binding  GO:0048018 receptor ligand activity |
| Growth arrest and DNA damage-inducible protein GADD45 alpha | GO:0005515 protein binding  GO:0019900 kinase binding  GO:0042803 protein homodimerization activity | GO:0046982 protein heterodimerization activity  GO:0047485 protein N-terminus binding  GO:1990841 promoter-specific chromatin binding |
| Growth factor receptor-bound protein 2 | GO:0001784 phosphotyrosine residue binding  GO:0003723 RNA binding  GO:0005154 epidermal growth factor receptor binding  GO:0005168 neurotrophin TRKA receptor binding  GO:0005515 protein binding  GO:0017124 SH3 domain binding  GO:0019899 enzyme binding | GO:0019901 protein kinase binding  GO:0019903 protein phosphatase binding  GO:0019904 protein domain specific binding  GO:0042802 identical protein binding  GO:0043560 insulin receptor substrate binding GO:0044877 protein-containing complex binding  GO:0046875 ephrin receptor binding  GO:0051219 phosphoprotein binding |
| Growth/differentiation factor 2 | GO:0005125 cytokine activity  GO:0005515 protein binding | GO:0008083 growth factor activity |
| Growth/differentiation factor 3 | GO:0005125 cytokine activity  GO:0008083 growth factor activity | GO:0019901 protein kinase binding |
| Growth/differentiation factor 5 | GO:0005102 signaling receptor binding  GO:0005125 cytokine activity  GO:0005515 protein binding | GO:0008083 growth factor activity  GO:0036122 BMP binding  GO:0042802 identical protein binding |
| Growth/differentiation factor 8 | GO:0005102 signaling receptor binding  GO:0005125 cytokine activity  GO:0005515 protein binding  GO:0008083 growth factor activity | GO:0008201 heparin binding  GO:0042802 identical protein binding  GO:0042803 protein homodimerization activity |
| Growth/differentiation factor 9 | GO:0005125 cytokine activity  GO:0008083 growth factor activity | GO:0070699 type II activin receptor binding |
| Growth/diﬀerentiation factor 11 | GO:0005125 cytokine activity  GO:0005515 protein binding | GO:0008083 growth factor activity |
| Guanine nucleotide-binding protein G(I)/G(S)/G(O) subunit gamma-12 | GO:0003924 GTPase activity  GO:0005515 protein binding  GO:0030165 PDZ domain binding | GO:0031681 G-protein beta-subunit binding  GO:0042301 phosphate ion binding |
| Guanine nucleotide-binding protein subunit alpha-13 | GO:0000166 nucleotide binding  GO:0001664 G protein-coupled receptor binding  GO:0003924 GTPase activity  GO:0005515 protein binding  GO:0005525 GTP binding | GO:0019001 guanyl nucleotide binding  GO:0031683 G-protein beta/gamma-subunit complex binding  GO:0031752 D5 dopamine receptor binding GO:0046872 metal ion binding |
| Haptoglobin | GO:0004252 serine-type endopeptidase activity  GO:0005515 protein binding | GO:0016209 antioxidant activity  GO:0030492 hemoglobin binding |
| Heat shock protein 105 kDa | GO:0000166 nucleotide binding  GO:0000774 adenyl-nucleotide exchange factor activity | GO:0005515 protein binding  GO:0005524 ATP binding  GO:0043014 alpha-tubulin binding |
| Heat shock protein beta-1 | GO:0003723 RNA binding  GO:0005080 protein kinase C binding  GO:0005515 protein binding  GO:0008426 protein kinase C inhibitor activity  GO:0019901 protein kinase binding | GO:0042802 identical protein binding  GO:0042803 protein homodimerization activity  GO:0043130 ubiquitin binding  GO:0044183 protein folding chaperone |
| Hepatocyte growth factor activator | GO:0004252 serine-type endopeptidase activity  GO:0008233 peptidase activity | GO:0008236 serine-type peptidase activity  GO:0016787 hydrolase activity |
| Hepatocyte growth factor receptor | GO:0000166 nucleotide binding  GO:0004672 protein kinase activity  GO:0004713 protein tyrosine kinase activity  GO:0004714 transmembrane receptor protein tyrosine kinase activity  GO:0005008 hepatocyte growth factor-activated receptor activity  GO:0005515 protein binding | GO:0005524 ATP binding  GO:0016301 kinase activity  GO:0016740 transferase activity  GO:0017154 semaphorin receptor activity  GO:0019903 protein phosphatase binding  GO:0042802 identical protein binding |
| Hepatocyte growth factor-like protein alpha chain (cleaved from hepatocyte growth factor-like protein) | GO:0004252 serine-type endopeptidase activity  GO:0005515 protein binding  GO:0030971 receptor tyrosine kinase binding | |
| Hepatocyte growth factor-regulated tyrosine kinase substrate | GO:0005515 protein binding  GO:0019904 protein domain specific binding | GO:0044389 ubiquitin-like protein ligase binding  GO:0046872 metal ion binding |
| Hepcidin | GO:0005102 signaling receptor binding  GO:0005179 hormone activity  GO:0005507 copper ion binding | GO:0097690 iron ion transmembrane transporter inhibitor activity |
| Histone H4 | GO:0003677 DNA binding  GO:0003723 RNA binding  GO:0005515 protein binding | GO:0019904 protein domain specific binding  GO:0046982 protein heterodimerization activity |

| HLA class II histocompatibility antigen gamma chain | GO:0001540 amyloid-beta binding  GO:0004896 cytokine receptor activity  GO:0005515 protein binding  GO:0019955 cytokine binding  GO:0023026 MHC class II protein complex binding  GO:0035718 macrophage migration inhibitory factor binding | GO:0042289 MHC class II protein binding  GO:0042609 CD4 receptor binding  GO:0042658 MHC class II protein binding, via antigen binding groove  GO:0042802 identical protein binding  GO:0044183 protein folding chaperone  GO:0050998 nitric-oxide synthase binding |
| --- | --- | --- |
| Homeobox protein NANOG | GO:0000976 transcription regulatory region sequence-specific DNA binding  GO:0000977 RNA polymerase II transcription regulatory region sequence-specific DNA binding  GO:0000978 RNA polymerase II cis-regulatory region sequence-specific DNA binding  GO:0000981 DNA-binding transcription factor activity, RNA polymerase II-specific  GO:0001227 DNA-binding transcription repressor activity, RNA polymerase II-specific | GO:0003677 DNA binding  GO:0003700 DNA-binding transcription factor activity  GO:0005515 protein binding  GO:1990837 sequence-specific double-stranded DNA binding |
| Hornerin | GO:0005509 calcium ion binding  GO:0046872 metal ion binding | GO:0046914 transition metal ion binding |
| Inhibin beta A chain | GO:0005102 signaling receptor binding  GO:0005125 cytokine activity  GO:0005179 hormone activity  GO:0005515 protein binding  GO:0008083 growth factor activity | GO:0017046 peptide hormone binding  GO:0034711 inhibin binding  GO:0042802 identical protein binding  GO:0044877 protein-containing complex binding  GO:0070699 type II activin receptor binding |
| Inhibin beta B chain | GO:0005125 cytokine activity  GO:0005179 hormone activity  GO:0005515 protein binding | GO:0008083 growth factor activity  GO:0042803 protein homodimerization activity  GO:0046789 host cell surface receptor binding |
| Inhibin beta C chain | GO:0005125 cytokine activity  GO:0005160 transforming growth factor beta receptor binding | GO:0005179 hormone activity  GO:0008083 growth factor activity |
| Insulin receptor | GO:0000166 nucleotide binding  GO:0001540 amyloid-beta binding  GO:0004672 protein kinase activity  GO:0004713 protein tyrosine kinase activity  GO:0004714 transmembrane receptor protein tyrosine kinase activity  GO:0005009 insulin-activated receptor activity  GO:0005159 insulin-like growth factor receptor binding  GO:0005198 structural molecule activity  GO:0005515 protein binding  GO:0005524 ATP binding  GO:0005525 GTP binding  GO:0016301 kinase activity | GO:0016740 transferase activity  GO:0019904 protein domain specific binding  GO:0031994 insulin-like growth factor I binding  GO:0031995 insulin-like growth factor II binding  GO:0038024 cargo receptor activity  GO:0042802 identical protein binding  GO:0043548 phosphatidylinositol 3-kinase binding  GO:0043559 insulin binding  GO:0043560 insulin receptor substrate binding  GO:0044877 protein-containing complex binding  GO:0051425 PTB domain binding |
| Insulin-degrading enzyme | GO:0000166 nucleotide binding  GO:0001540 amyloid-beta binding  GO:0001618 virus receptor activity  GO:0003824 catalytic activity  GO:0004175 endopeptidase activity  GO:0004222 metalloendopeptidase activity  GO:0005515 protein binding  GO:0005524 ATP binding  GO:0008233 peptidase activity  GO:0008237 metallopeptidase activity  GO:0008270 zinc ion binding | GO:0016787 hydrolase activity  GO:0016887 ATPase activity  GO:0017046 peptide hormone binding  GO:0031626 beta-endorphin binding  GO:0042277 peptide binding  GO:0042802 identical protein binding  GO:0042803 protein homodimerization activity  GO:0043559 insulin binding  GO:0044877 protein-containing complex binding  GO:0046872 metal ion binding  GO:0140036 ubiquitin-dependent protein binding |
| Insulin-like growth factor 1 receptor | GO:0000166 nucleotide binding  GO:0004672 protein kinase activity  GO:0004713 protein tyrosine kinase activity  GO:0004714 transmembrane receptor protein tyrosine kinase activity  GO:0005009 insulin-activated receptor activity  GO:0005010 insulin-like growth factor-activated receptor activity  GO:0005158 insulin receptor binding  GO:0005198 structural molecule activity  GO:0005515 protein binding | GO:0005520 insulin-like growth factor binding  GO:0005524 ATP binding  GO:0016301 kinase activity  GO:0016740 transferase activity  GO:0031994 insulin-like growth factor I binding  GO:0042802 identical protein binding  GO:0043548 phosphatidylinositol 3-kinase binding  GO:0043559 insulin binding  GO:0043560 insulin receptor substrate binding  GO:0140318 protein transporter activity |
| Insulin-like growth factor I | GO:0005158 insulin receptor binding  GO:0005159 insulin-like growth factor receptor binding  GO:0005178 integrin binding | GO:0005179 hormone activity  GO:0005515 protein binding  GO:0008083 growth factor activity |
| Insulin-like growth factor-binding protein 1 | GO:0005102 signaling receptor binding  GO:0005515 protein binding  GO:0005520 insulin-like growth factor binding | GO:0019838 growth factor binding  GO:0031994 insulin-like growth factor I binding  GO:0031995 insulin-like growth factor II binding |
| Insulin-like growth factor-binding protein 3 | GO:0001968 fibronectin binding  GO:0005515 protein binding GO:0005520 insulin-like growth factor binding GO:0008160 protein tyrosine phosphatase activator activity | GO:0019838 growth factor binding  GO:0031994 insulin-like growth factor I binding GO:0031995 insulin-like growth factor II binding GO:0046872 metal ion binding |

| Insulin-like growth factor-binding protein 4 | GO:0005102 signaling receptor binding  GO:0005515 protein binding  GO:0005520 insulin-like growth factor binding | GO:0019838 growth factor binding  GO:0031994 insulin-like growth factor I binding  GO:0031995 insulin-like growth factor II binding |
| --- | --- | --- |
| Insulin-like growth factor-binding protein 5 | GO:0001968 fibronectin binding  GO:0005515 protein binding  GO:0005520 insulin-like growth factor binding | GO:0019838 growth factor binding  GO:0031994 insulin-like growth factor I binding  GO:0031995 insulin-like growth factor II binding |
| Insulin-like growth factor-binding protein 7 | GO:0005201 extracellular matrix structural constituent GO:0005515 protein binding | GO:0005520 insulin-like growth factor binding GO:0019838 growth factor binding |
| Insulin-like growth factor-binding protein complex acid labile subunit | GO:0005520 insulin-like growth factor binding |  |
| Integrin alpha-1 | GO:0005102 signaling receptor binding  GO:0005515 protein binding GO:0005518 collagen binding GO:0019903 protein phosphatase binding | GO:0046872 metal ion binding GO:0098639 collagen binding involved in cell-matrix adhesion |
| Integrin alpha-2 | GO:0001540 amyloid-beta binding  GO:0001618 virus receptor activity  GO:0005178 integrin binding  GO:0005515 protein binding GO:0005518 collagen binding  GO:0038064 collagen receptor activity | GO:0043236 laminin binding GO:0043395 heparan sulfate proteoglycan binding  GO:0044877 protein-containing complex binding GO:0046872 metal ion binding GO:0098639 collagen binding involved in cell-matrix adhesión |
| Integrin alpha-3 | GO:0001968 fibronectin binding GO:0002020 protease binding  GO:0005178 integrin binding  GO:0005515 protein binding GO:0005518 collagen binding | GO:0019904 protein domain specific binding GO:0043236 laminin binding GO:0044877 protein-containing complex binding GO:0046872 metal ion binding GO:0046982 protein heterodimerization activity |
| Integrin alpha-4 | GO:0001968 fibronectin binding GO:0003823 antigen binding GO:0005178 integrin binding  GO:0005515 protein binding  GO:0015026 coreceptor activity | GO:0019960 C-X3-C chemokine binding GO:0046872 metal ion binding  GO:0050839 cell adhesion molecule binding GO:1990405 protein antigen binding |
| Integrin alpha-5 | GO:0001618 virus receptor activity  GO:0005154 epidermal growth factor receptor binding  GO:0005161 platelet-derived growth factor receptor binding  GO:0005178 integrin binding | GO:0005515 protein binding GO:0043184 vascular endothelial growth factor receptor 2 binding GO:0046872 metal ion binding  GO:0050839 cell adhesion molecule binding |
| Integrin alpha-6 | GO:0005515 protein binding GO:0031994 insulin-like growth factor I binding GO:0038132 neuregulin binding GO:0043236 laminin binding | GO:0044877 protein-containing complex binding GO:0045296 cadherin binding GO:0046872 metal ion binding |
| Integrin alpha-7 | GO:0005515 protein binding | GO:0046872 metal ion binding |
| Integrin alpha-10 | GO:0005518 collagen binding GO:0046872 metal ion binding | GO:0098639 collagen binding involved in cell-matrix adhesion |
| Integrin alpha-11 | GO:0005518 collagen binding GO:0038064 collagen receptor activity  GO:0046872 metal ion binding | GO:0098639 collagen binding involved in cell-matrix adhesion |
| Integrin alpha-M | GO:0001540 amyloid-beta binding  GO:0001851 complement component C3b binding  GO:0005178 integrin binding  GO:0005515 protein binding | GO:0031072 heat shock protein binding  GO:0038024 cargo receptor activity  GO:0044877 protein-containing complex binding  GO:0046872 metal ion binding |
| Integrin alpha-V | GO:0001618 virus receptor activity GO:0001846 opsonin binding  GO:0001968 fibronectin binding GO:0002020 protease binding GO:0005080 protein kinase C binding  GO:0005102 signaling receptor binding GO:0005178 integrin binding GO:0005245 voltage-gated calcium channel activity  GO:0005515 protein binding | GO:0015026 coreceptor activity GO:0017134 fibroblast growth factor binding GO:0019960 C-X3-C chemokine binding GO:0031994 insulin-like growth factor I binding GO:0038132 neuregulin binding GO:0046872 metal ion binding GO:0050431 transforming growth factor beta binding GO:0050840 extracellular matrix binding GO:1990430 extracellular matrix protein binding |
| Integrin beta-1 | GO:0001618 virus receptor activity  GO:0001968 fibronectin binding GO:0002020 protease binding  GO:0003779 actin binding  GO:0005178 integrin binding  GO:0005515 protein binding GO:0015026 coreceptor activity  GO:0019960 C-X3-C chemokine binding GO:0038023 signaling receptor activity | GO:0043236 laminin binding GO:0044877 protein-containing complex binding GO:0045296 cadherin binding GO:0046872 metal ion binding GO:0046982 protein heterodimerization activity GO:0050839 cell adhesion molecule binding  GO:0098639 collagen binding involved in cell-matrix adhesión  GO:1990782 protein tyrosine kinase binding |
| Integrin beta-1-binding protein 1 | GO:0005178 integrin binding GO:0005092 GDP-dissociation inhibitor activity  GO:0005515 protein binding | GO:0019900 kinase binding GO:0019901 protein kinase binding |

| Integrin beta-3 | GO:0001618 virus receptor activity  GO:0001968 fibronectin binding GO:0002020 protease binding  GO:0003756 protein disulfide isomerase activity GO:0005161 platelet-derived growth factor receptor binding  GO:0005178 integrin binding  GO:0005515 protein binding  GO:0015026 coreceptor activity GO:0017134 fibroblast growth factor binding | GO:0019899 enzyme binding GO:0019960 C-X3-C chemokine binding GO:0031994 insulin-like growth factor I binding GO:0038132 neuregulin binding  GO:0042802 identical protein binding GO:0043184 vascular endothelial growth factor receptor 2 binding GO:0050839 cell adhesion molecule binding GO:0050840 extracellular matrix binding |
| --- | --- | --- |
| Integrin beta-5 | GO:0001618 virus receptor activity  GO:0005178 integrin binding | GO:0005515 protein binding GO:0038023 signaling receptor activity |
| Integrin-linked protein kinase | GO:0000166 nucleotide binding  GO:0004672 protein kinase activity  GO:0004674 protein serine/threonine kinase activity  GO:0005178 integrin binding  GO:0005515 protein binding  GO:0005524 ATP binding | GO:0016301 kinase activity GO:0016740 transferase activity GO:0017124 SH3 domain binding GO:0019901 protein kinase binding GO:0019904 protein domain specific binding GO:0044877 protein-containing complex binding |
| Inter-alpha-trypsin inhibitor heavy chain H2 | GO:0004866 endopeptidase inhibitor activity  GO:0004867 serine-type endopeptidase inhibitor activity | GO:0030414 peptidase inhibitor activity |
| Intercellular adhesion molecule 1 | GO:0001618 virus receptor activity  GO:0004888 transmembrane signaling receptor activity  GO:0005178 integrin binding | GO:0005515 protein binding  GO:0038023 signaling receptor activity GO:0044877 protein-containing complex binding |
| Intercellular adhesion molecule 2 | GO:0005178 integrin binding |  |
| Interferon beta | GO:0005125 cytokine activity  GO:0005126 cytokine receptor binding  GO:0005132 type I interferon receptor binding | GO:0005515 protein binding  GO:0008811 chloramphenicol O-acetyltransferase activity |
| Interferon gamma | GO:0005125 cytokine activity  GO:0005133 interferon-gamma receptor binding | GO:0005515 protein binding |
| Interferon lambda-1 | GO:0005102 signaling receptor binding  GO:0005125 cytokine activity | GO:0032003 interleukin-28 receptor binding |
| Interferon lambda-2 | GO:0005102 signaling receptor binding | GO:0005125 cytokine activity |
| Interferon regulatory factor 6 | GO:0000976 transcription regulatory region sequence-specific DNA binding  GO:0000978 RNA polymerase II cis-regulatory region sequence-specific DNA binding  GO:0000981 DNA-binding transcription factor activity, RNA polymerase II-specific  GO:0001228 DNA-binding transcription activator activity, RNA polymerase II-specific  GO:0003677 DNA binding | GO:0003700 DNA-binding transcription factor activity  GO:0005515 protein binding  GO:0043565 sequence-specific DNA binding  GO:1990837 sequence-specific double-stranded DNA binding |
| Interleukin-1 alpha | GO:0005125 cytokine activity  GO:0005149 interleukin-1 receptor binding | GO:0005507 copper ion binding  GO:0005515 protein binding |
| Interleukin-1 beta | GO:0005125 cytokine activity GO:0005149 interleukin-1 receptor binding GO:0005178 integrin binding | GO:0005515 protein binding GO:0019904 protein domain specific binding |
| Interleukin-1 family member 10 | GO:0005125 cytokine activity  GO:0005149 interleukin-1 receptor binding | GO:0005515 protein binding |
| Interleukin-1 receptor accessory protein-like 1 | GO:0003953 NAD+ nucleosidase activity  GO:0005102 signaling receptor binding  GO:0005515 protein binding  GO:0016787 hydrolase activity | GO:0019966 interleukin-1 binding  GO:0050135 NAD(P)+ nucleosidase activity  GO:0061809 NAD+ nucleotidase, cyclic ADP-ribose generating |
| Interleukin-1 receptor type 1 | GO:0002020 protease binding  GO:0003953 NAD+ nucleosidase activity  GO:0004888 transmembrane signaling receptor activity  GO:0004908 interleukin-1 receptor activity  GO:0004909 interleukin-1, type I, activating receptor activity  GO:0005161 platelet-derived growth factor receptor binding | GO:0005515 protein binding  GO:0016787 hydrolase activity  GO:0019966 interleukin-1 binding  GO:0050135 NAD(P)+ nucleosidase activity  GO:0061809 NAD+ nucleotidase, cyclic ADP-ribose generating |
| Interleukin-1 receptor type 2 | GO:0004908 interleukin-1 receptor activity  GO:0004910 interleukin-1, type II, blocking receptor activity | GO:0005515 protein binding  GO:0019966 interleukin-1 binding |
| Interleukin-1 receptor-like 1 | GO:0002113 interleukin-33 binding  GO:0002114 interleukin-33 receptor activity  GO:0003953 NAD+ nucleosidase activity  GO:0004896 cytokine receptor activity  GO:0004908 interleukin-1 receptor activity | GO:0005515 protein binding  GO:0016787 hydrolase activity  GO:0050135 NAD(P)+ nucleosidase activity  GO:0061809 NAD+ nucleotidase, cyclic ADP-ribose generating |
| Interleukin-1 receptor-like 2 | GO:0003953 NAD+ nucleosidase activity  GO:0004908 interleukin-1 receptor activity  GO:0004909 interleukin-1, type I, activating receptor activity | GO:0016787 hydrolase activity  GO:0050135 NAD(P)+ nucleosidase activity  GO:0061809 NAD+ nucleotidase, cyclic ADP-ribose generating |
| Interleukin-2 | GO:0005125 cytokine activity  GO:0005134 interleukin-2 receptor binding  GO:0005515 protein binding  GO:0008083 growth factor activity | GO:0019209 kinase activator activity  GO:0030246 carbohydrate binding  GO:0031851 kappa-type opioid receptor binding  GO:0043208 glycosphingolipid binding |
| Interleukin-2 receptor subunit alpha | GO:0004911 interleukin-2 receptor activity  GO:0005515 protein binding | GO:0019976 interleukin-2 binding |
| Interleukin-2 receptor subunit beta | GO:0005515 protein binding  GO:0019976 interleukin-2 binding  GO:0004911 interleukin-2 receptor activity | GO:0004896 cytokine receptor activity  GO:0042010 interleukin-15 receptor activity |
| Interleukin-4 | GO:0005125 cytokine activity GO:0005126 cytokine receptor binding GO:0005136 interleukin-4 receptor binding | GO:0005515 protein binding  GO:0008083 growth factor activity |
| Interleukin-5 | GO:0005125 cytokine activity  GO:0005137 interleukin-5 receptor binding | GO:0005515 protein binding  GO:0008083 growth factor activity |
| Interleukin-6 | GO:0005125 cytokine activity  GO:0005138 interleukin-6 receptor binding | GO:0005515 protein binding GO:0008083 growth factor activity |
| Interleukin-7 | GO:0005125 cytokine activity  GO:0005126 cytokine receptor binding  GO:0005139 interleukin-7 receptor binding | GO:0005515 protein binding  GO:0008083 growth factor activity |
| Interleukin-7 receptor subunit alpha | GO:0003823 antigen binding  GO:0004896 cytokine receptor activity | GO:0004917 interleukin-7 receptor activity  GO:0005515 protein binding |
| Interleukin-8 | GO:0005125 cytokine activity  GO:0005153 interleukin-8 receptor binding  GO:0005515 protein binding | GO:0008009 chemokine activity  GO:0045236 CXCR chemokine receptor binding |
| Interleukin-9 | GO:0005125 cytokine activity  GO:0005126 cytokine receptor binding | GO:0005140 interleukin-9 receptor binding  GO:0008083 growth factor activity |
| Interleukin-10 | GO:0005125 cytokine activity GO:0005141 interleukin-10 receptor binding  GO:0005515 protein binding | GO:0008083 growth factor activity GO:0046983 protein dimerization activity |
| Interleukin-10 receptor subunit alpha | GO:0004896 cytokine receptor activity  GO:0004920 interleukin-10 receptor activity  GO:0005515 protein binding | GO:0019969 interleukin-10 binding  GO:0038023 signaling receptor activity |
| Interleukin-11 | GO:0005125 cytokine activity  GO:0005142 interleukin-11 receptor binding | GO:0005515 protein binding  GO:0008083 growth factor activity |
| Interleukin-12 subunit alpha | GO:0005125 cytokine activity  GO:0005143 interleukin-12 receptor binding  GO:0005515 protein binding GO:0008083 growth factor activity | GO:0042163 interleukin-12 beta subunit binding  GO:0046982 protein heterodimerization activity GO:0045513 interleukin-27 binding |
| Interleukin-12 subunit beta | GO:0004896 cytokine receptor activity GO:0005143 interleukin-12 receptor binding  GO:0005125 cytokine activity  GO:0005126 cytokine receptor binding  GO:0005515 protein binding  GO:0008083 growth factor activity | GO:0019955 cytokine binding GO:0042802 identical protein binding  GO:0042164 interleukin-12 alpha subunit binding GO:0044877 protein-containing complex binding GO:0045519 interleukin-23 receptor binding  GO:0046982 protein heterodimerization activity |
| Interleukin-13 receptor subunit alpha-1 | GO:0004896 cytokine receptor activity  GO:0004923 leukemia inhibitory factor receptor activity  GO:0004924 oncostatin-M receptor activity | GO:0005127 ciliary neurotrophic factor receptor binding  GO:0005515 protein binding  GO:0019955 cytokine binding |
| Interleukin-13 receptor subunit alpha-2 | GO:0004896 cytokine receptor activity  GO:0005515 protein binding | GO:0019955 cytokine binding |
| Interleukin-13 | GO:0005125 cytokine activity  GO:0005126 cytokine receptor binding | GO:0005144 interleukin-13 receptor binding  GO:0005515 protein binding |
| Interleukin-15 | GO:0005125 cytokine activity  GO:0005126 cytokine receptor binding | GO:0005515 protein binding |
| Interleukin-17 receptor B | GO:0004896 cytokine receptor activity  GO:0005515 protein binding | GO:0030368 interleukin-17 receptor activity |
| Interleukin-17 receptor C | GO:0005102 signaling receptor binding  GO:0005515 protein binding | GO:0030368 interleukin-17 receptor activity |
| Interleukin-17A | GO:0005125 cytokine activity  GO:0005515 protein binding | GO:0042803 protein homodimerization activity  GO:0046982 protein heterodimerization activity |
| Interleukin-17C | GO:0005125 cytokine activity | |
| Interleukin-19 | GO:0005125 cytokine activity | GO:0005515 protein binding |
| Interleukin-20 receptor subunit alpha | GO:0004896 cytokine receptor activity  GO:0005515 protein binding | GO:0042015 interleukin-20 binding |
| Interleukin-21 receptor | GO:0001532 interleukin-21 receptor activity  GO:0004888 transmembrane signaling receptor activity | GO:0004896 cytokine receptor activity  GO:0005515 protein binding |
| Interleukin-21 | GO:0005125 cytokine activity  GO:0005126 cytokine receptor binding | GO:0005134 interleukin-2 receptor binding  GO:0005515 protein binding |
| Interleukin-23 receptor | GO:0004896 cytokine receptor activity  GO:0005143 interleukin-12 receptor binding  GO:0005515 protein binding | GO:0019955 cytokine binding  GO:0042019 interleukin-23 binding  GO:0042020 interleukin-23 receptor activity |
| Interleukin-23 subunit alpha | GO:0005125 cytokine activity  GO:0005515 protein binding | GO:0045519 interleukin-23 receptor binding |
| Interleukin-24 | GO:0005125 cytokine activity | GO:0005515 protein binding |
| Interleukin-27 subunit alpha | GO:0005102 signaling receptor binding  GO:0005125 cytokine activity | GO:0005515 protein binding  GO:0045523 interleukin-27 receptor binding |
| Interleukin-36 gamma | GO:0005125 cytokine activity | GO:0005149 interleukin-1 receptor binding |
| Interleukin-36 receptor antagonist protein | GO:0005125 cytokine activity  GO:0005149 interleukin-1 receptor binding | GO:0005152 interleukin-1 receptor antagonist activity  GO:0005515 protein binding |
| Interstitial collagenase | GO:0004175 endopeptidase activity  GO:0004222 metalloendopeptidase activity  GO:0004252 serine-type endopeptidase activity GO:0008233 peptidase activity | GO:0008237 metallopeptidase activity GO:0008270 zinc ion binding  GO:0016787 hydrolase activity GO:0046872 metal ion binding |
| Islet amyloid polypeptide | GO:0001540 amyloid-beta binding  GO:0005102 signaling receptor binding  GO:0005179 hormone activity | GO:0005515 protein binding  GO:0042802 identical protein binding |
| Junctional adhesion molecule C | GO:0005178 integrin binding GO:0005515 protein binding GO:0042802 identical protein binding | GO:0042803 protein homodimerization activity GO:0046982 protein heterodimerization activity  GO:0098632 cell-cell adhesion mediator activity |
| Junctional adhesion molecule-like | GO:0005178 integrin binding  GO:0005515 protein binding | GO:0042803 protein homodimerization activity  GO:0050839 cell adhesion molecule binding |
| Kallikrein 2 | GO:0004252 serine-type endopeptidase activity  GO:0008233 peptidase activity | GO:0008236 serine-type peptidase activity  GO:0016787 hydrolase activity |
| Kallikrein 11 | GO:0004252 serine-type endopeptidase activity  GO:0008233 peptidase activity | GO:0008236 serine-type peptidase activity  GO:0016787 hydrolase activity |
| Keratin, type I cytoskeletal 19 | GO:0005198 structural molecule activity  GO:0005200 structural constituent of cytoskeleton  GO:0005515 protein binding | GO:0008307 structural constituent of muscle  GO:0044877 protein-containing complex binding |
| Kremen protein 1 | GO:0005515 protein binding | |
| Kremen protein 2 | - | |
| Lactadherin | GO:0001786 phosphatidylserine binding  GO:0005178 integrin binding  GO:0005201 extracellular matrix structural constituent | GO:0008429 phosphatidylethanolamine binding |
| Lactotransferrin | GO:0001530 lipopolysaccharide binding  GO:0003677 DNA binding  GO:0004252 serine-type endopeptidase activity  GO:0004869 cysteine-type endopeptidase inhibitor activity GO:0005506 iron ion binding  GO:0005515 protein binding | GO:0008201 heparin binding GO:0008233 peptidase activity  GO:0008236 serine-type peptidase activity GO:0016787 hydrolase activity GO:0043539 protein serine/threonine kinase activator activity GO:0046872 metal ion binding |
| Lactoylglutathione lyase | GO:0004462 lactoylglutathione lyase activity  GO:0005515 protein binding  GO:0008270 zinc ion binding | GO:0016829 lyase activity  GO:0046872 metal ion binding |
| Laminin subunit alpha-1 | GO:0005102 signaling receptor binding  GO:0005201 extracellular matrix structural constituent | GO:0005515 protein binding  GO:0008022 protein C-terminus binding GO:0043208 glycosphingolipid binding |
| Laminin subunit alpha-2 | GO:0005102 signaling receptor binding  GO:0005198 structural molecule activity | GO:0005201 extracellular matrix structural constituent |
| Laminin subunit alpha-4 | GO:0005102 signaling receptor binding  GO:0005201 extracellular matrix structural constituent | GO:0005515 protein binding |
| Laminin subunit alpha-5 | GO:0005102 signaling receptor binding  GO:0005178 integrin binding | GO:0005201 extracellular matrix structural constituent |
| Laminin subunit beta-1 | GO:0005178 integrin binding  GO:0005198 structural molecule activity | GO:0005201 extracellular matrix structural constituent  GO:0005515 protein binding |
| Laminin subunit beta-2 | GO:0005178 integrin binding  GO:0005198 structural molecule activity | GO:0005201 extracellular matrix structural constituent |
| Laminin subunit gamma-1 | GO:0005201 extracellular matrix structural constituent | GO:0030023 extracellular matrix constituent conferring elasticity |
| Latent-transforming growth factor beta-binding protein 1 | GO:0005024 transforming growth factor beta-activated receptor activity  GO:0005201 extracellular matrix structural constituent GO:0005509 calcium ion binding | GO:0005515 protein binding GO:0019838 growth factor binding  GO:0050431 transforming growth factor beta binding  GO:0050436 microfibril binding |
| Layilin | GO:0005515 protein binding  GO:0005540 hyaluronic acid binding | GO:0030246 carbohydrate binding |
| Leucine-rich alpha-2-glycoprotein | GO:0003674 molecular_function  GO:0005160 transforming growth factor beta receptor binding | GO:0005515 protein binding |
| Leukocyte surface antigen CD47 | GO:0005515 protein binding GO:0070053 thrombospondin receptor activity | GO:0086080 protein binding involved in heterotypic cell-cell adhesion GO:0098632 cell-cell adhesion mediator activity |
| Lipopolysaccharide-binding protein | GO:0001530 lipopolysaccharide binding  GO:0005102 signaling receptor binding  GO:0005515 protein binding | GO:0008289 lipid binding  GO:0070891 lipoteichoic acid binding  GO:0071723 lipopeptide binding |

| L-lactate dehydrogenase A chain | GO:0003824 catalytic activity GO:0004457 lactate dehydrogenase activity  GO:0004459 L-lactate dehydrogenase activity  GO:0005515 protein binding  GO:0016491 oxidoreductase activity | GO:0016616 oxidoreductase activity, acting on the CH-OH group of donors, NAD or NADP as acceptor  GO:0019900 kinase binding  GO:0042802 identical protein binding GO:0045296 cadherin binding GO:0051287 NAD binding |  |
| --- | --- | --- | --- |
| Low affinity immunoglobulin epsilon Fc receptor | GO:0005178 integrin binding  GO:0005515 protein binding  GO:0019863 IgE binding  GO:0030246 carbohydrate binding  GO:0046872 metal ion binding | |  |
| Low-density lipoprotein receptor | GO:0001540 amyloid-beta binding  GO:0001618 virus receptor activity  GO:0002020 protease binding  GO:0005041 low-density lipoprotein particle receptor activity  GO:0005509 calcium ion binding | GO:0005515 protein binding  GO:0030169 low-density lipoprotein particle binding  GO:0030229 very-low-density lipoprotein particle receptor activity  GO:0032050 clathrin heavy chain binding  GO:0042802 identical protein binding |  |
| Low-density lipoprotein receptor-related protein 6 | GO:0005041 low-density lipoprotein particle receptor activity  GO:0005102 signaling receptor binding GO:0005109 frizzled binding  GO:0005515 protein binding  GO:0015026 coreceptor activity  GO:0017147 Wnt-protein binding  GO:0019210 kinase inhibitor activity  GO:0019534 toxin transmembrane transporter activity | GO:0034185 apolipoprotein binding  GO:0042802 identical protein binding GO:0042803 protein homodimerization activity GO:0042813 Wnt-activated receptor activity GO:0071936 coreceptor activity involved in Wnt signaling pathway GO:1904928 coreceptor activity involved in canonical Wnt signaling pathway |  |
| L-Selectin | GO:0002020 protease binding  GO:0005509 calcium ion binding  GO:0005515 protein binding  GO:0008201 heparin binding | GO:0030246 carbohydrate binding  GO:0043208 glycosphingolipid binding  GO:0046872 metal ion binding  GO:0070492 oligosaccharide binding |  |
| Lutropin-choriogonadotropic hormone receptor | GO:0004930 G protein-coupled receptor activity  GO:0004964 luteinizing hormone receptor activity  GO:0008528 G protein-coupled peptide receptor activity | GO:0016500 protein-hormone receptor activity  GO:0035472 choriogonadotropin hormone receptor activity  GO:0038106 choriogonadotropin hormone binding |  |
| Lymphocyte activation gene 3 protein | GO:0003823 antigen binding  GO:0004888 transmembrane signaling receptor activity | GO:0005515 protein binding  GO:0042289 MHC class II protein binding |  |
| Lymphotoxin-alpha | GO:0005102 signaling receptor binding  GO:0005125 cytokine activity | GO:0005164 tumor necrosis factor receptor binding  GO:0005515 protein binding |  |
| Lymphotoxin-beta | GO:0005102 signaling receptor binding  GO:0005125 cytokine activity | GO:0005164 tumor necrosis factor receptor binding |  |
| Lysosome membrane protein 2 | GO:0001618 virus receptor activity  GO:0001786 phosphatidylserine binding  GO:0004888 transmembrane signaling receptor activity  GO:0005044 scavenger receptor activity  GO:0005515 protein binding | GO:0015485 cholesterol binding  GO:0019899 enzyme binding  GO:0031210 phosphatidylcholine binding  GO:0038024 cargo receptor activity  GO:0042803 protein homodimerization activity |  |
| Lysosome-associated membrane glycoprotein 2 | GO:0005515 protein binding GO:0019899 enzyme binding | GO:0019904 protein domain specific binding |  |
| Macrophage migration inhibitory factor | GO:0004175 endopeptidase activity  GO:0005125 cytokine activity  GO:0005126 cytokine receptor binding  GO:0005515 protein binding | GO:0016853 isomerase activity  GO:0042056 chemoattractant activity  GO:0042802 identical protein binding  GO:0050178 phenylpyruvate tautomerase activity |  |
| Mammaglobin A | GO:0003674 molecular_function | GO:0005515 protein binding |  |
| Mast/stem cell growth factor receptor Kit | GO:0000166 nucleotide binding GO:0002020 protease binding  GO:0004672 protein kinase activity  GO:0004713 protein tyrosine kinase activity GO:0004714 transmembrane receptor protein tyrosine kinase activity  GO:0005020 stem cell factor receptor activity  GO:0005515 protein binding | GO:0005524 ATP binding  GO:0016301 kinase activity GO:0016740 transferase activity GO:0019838 growth factor binding  GO:0019955 cytokine binding GO:0042169 SH2 domain binding GO:0042803 protein homodimerization activity GO:0046872 metal ion binding |  |
| Matrilysin | GO:0004175 endopeptidase activity  GO:0004222 metalloendopeptidase activity  GO:0005515 protein binding  GO:0008201 heparin binding  GO:0008233 peptidase activity | GO:0008237 metallopeptidase activity  GO:0008270 zinc ion binding  GO:0016787 hydrolase activity  GO:0046872 metal ion binding |  |
| Matrix metalloproteinase-9 | GO:0004222 metalloendopeptidase activity GO:0005515 protein binding  GO:0005518 collagen binding GO:0008233 peptidase activity  GO:0008237 metallopeptidase activity GO:0008270 zinc ion binding | GO:0004175 endopeptidase activity GO:0004252 serine-type endopeptidase activity  GO:0016787 hydrolase activity  GO:0042802 identical protein binding GO:0046872 metal ion binding |  |
| Matrix metalloproteinase-14 | GO:0004175 endopeptidase activity  GO:0004222 metalloendopeptidase activity  GO:0005178 integrin binding  GO:0005515 protein binding  GO:0008233 peptidase activity | GO:0008237 metallopeptidase activity  GO:0008270 zinc ion binding  GO:0016787 hydrolase activity  GO:0046872 metal ion binding  GO:0070006 metalloaminopeptidase activity |  |
| Matrix metalloproteinase-19 | GO:0004222 metalloendopeptidase activity  GO:0008233 peptidase activity  GO:0008237 metallopeptidase activity | GO:0008270 zinc ion binding  GO:0016787 hydrolase activity  GO:0046872 metal ion binding |  |
| Matrix metalloproteinase-20 | GO:0004222 metalloendopeptidase activity  GO:0005515 protein binding  GO:0008233 peptidase activity GO:0008237 metallopeptidase activity | GO:0008270 zinc ion binding GO:0016787 hydrolase activity GO:0046872 metal ion binding |  |
| Matrix metalloproteinase-24 | GO:0004222 metalloendopeptidase activity  GO:0008047 enzyme activator activity  GO:0008233 peptidase activity  GO:0008237 metallopeptidase activity | GO:0008270 zinc ion binding  GO:0016787 hydrolase activity  GO:0045296 cadherin binding  GO:0046872 metal ion binding |  |
| Megakaryocyte-associated tyrosine-protein kinase | GO:0000166 nucleotide binding  GO:0004672 protein kinase activity  GO:0004713 protein tyrosine kinase activity  GO:0004714 transmembrane receptor protein tyrosine kinase activity  GO:0004715 non-membrane spanning protein tyrosine kinase activity | GO:0005515 protein binding  GO:0005524 ATP binding  GO:0016301 kinase activity  GO:0016740 transferase activity |  |
| Metalloproteinase inhibitor 2 | GO:0002020 protease binding  GO:0004857 enzyme inhibitor activity  GO:0005178 integrin binding  GO:0005515 protein binding | GO:0008191 metalloendopeptidase inhibitor activity  GO:0008270 zinc ion binding GO:0030414 peptidase inhibitor activity  GO:0046872 metal ion binding |  |
| Metalloproteinase inhibitor 3 | GO:0002020 protease binding  GO:0004857 enzyme inhibitor activity  GO:0005515 protein binding | GO:0008191 metalloendopeptidase inhibitor activity  GO:0030414 peptidase inhibitor activity  GO:0046872 metal ion binding |  |
| MHC class I polypeptide-related sequence A | GO:0005515 protein binding  GO:0030881 beta-2-microglobulin binding | GO:0046703 natural killer cell lectin-like receptor binding |  |
| Microtubule-associated tumor suppressor 1 | GO:0005515 protein binding | GO:0008017 microtubule binding |  |
| Mitogen-activated protein kinase 1 | GO:0000166 nucleotide binding  GO:0001784 phosphotyrosine residue binding  GO:0003677 DNA binding GO:0003690 double-stranded DNA binding  GO:0004672 protein kinase activity  GO:0004674 protein serine/threonine kinase activity GO:0004707 MAP kinase activity  GO:0004708 MAP kinase kinase activity  GO:0005515 protein binding  GO:0005524 ATP binding | GO:0008134 transcription factor binding GO:0008353 RNA polymerase II CTD heptapeptide repeat kinase activity  GO:0016301 kinase activity  GO:0016740 transferase activity  GO:0019901 protein kinase binding GO:0019902 phosphatase binding  GO:0031435 mitogen-activated protein kinase kinase kinase binding  GO:0042802 identical protein binding |  |
| Mitogen-activated protein kinase 3 | GO:0000166 nucleotide binding GO:0001784 phosphotyrosine residue binding  GO:0004672 protein kinase activity  GO:0004674 protein serine/threonine kinase activity  GO:0004707 MAP kinase activity GO:0004708 MAP kinase kinase activity  GO:0005515 protein binding | GO:0005524 ATP binding  GO:0016301 kinase activity GO:0016740 transferase activity GO:0019902 phosphatase binding  GO:0042802 identical protein binding GO:0097110 scaffold protein binding |  |
| Monocyte differentiation antigen CD14 | GO:0001530 lipopolysaccharide binding  GO:0001847 opsonin receptor activity  GO:0005515 protein binding | GO:0016019 peptidoglycan immune receptor activity  GO:0070891 lipoteichoic acid binding  GO:0071723 lipopeptide binding |  |
| Mucin-1 | GO:0000978 RNA polymerase II cis-regulatory region sequence-specific DNA binding  GO:0002039 p53 binding | GO:0003712 transcription coregulator activity  GO:0005515 protein binding |  |
| Mucin-16 | GO:0005515 protein binding | |  |
| Mucosal addressin cell adhesion molecule 1 | GO:0098640 integrin binding involved in cell-matrix adhesion | |  |
| Muscle, skeletal receptor tyrosine-protein kinase | GO:0000166 nucleotide binding  GO:0004672 protein kinase activity  GO:0004713 protein tyrosine kinase activity  GO:0004714 transmembrane receptor protein tyrosine kinase activity  GO:0005515 protein binding  GO:0005518 collagen binding | GO:0005524 ATP binding  GO:0016301 kinase activity  GO:0016740 transferase activity  GO:0038062 protein tyrosine kinase collagen receptor activity  GO:0046872 metal ion binding |  |
| Myeloid-derived growth factor | GO:0005515 protein binding |  |  |
| Natriuretic peptides B | GO:0005102 signaling receptor binding  GO:0005179 hormone activity  GO:0005515 protein binding | GO:0008613 diuretic hormone activity  GO:0051427 hormone receptor binding |  |
| Natural killer cell receptor 2B4 | GO:0005515 protein binding  GO:0038023 signaling receptor activity | GO:0042289 MHC class II protein binding |  |
| Neprilysin | GO:0001786 phosphatidylserine binding  GO:0004175 endopeptidase activity GO:0004222 metalloendopeptidase activity  GO:0005515 protein binding  GO:0008233 peptidase activity GO:0008237 metallopeptidase activity  GO:0008238 exopeptidase activity | GO:0008270 zinc ion binding  GO:0016787 hydrolase activity GO:0042277 peptide binding  GO:0042803 protein homodimerization activity GO:0046872 metal ion binding GO:0070012 oligopeptidase activity GO:1901612 cardiolipin binding |  |
| Netrin-1 | GO:0005515 protein binding | |  |
| Netrin-G2 | GO:0003674 molecular_function | GO:0005515 protein binding |  |
| Neural cell adhesion molecule 1 | GO:0001618 virus receptor activity  GO:0005515 protein binding | GO:0042802 identical protein binding |  |
| Neural cell adhesion molecule L1-like protein | GO:0002020 protease binding | |  |
| Neuregulin-1 (cleaved form pro-neuregulin-1, membrane-bound isoform) | GO:0003712 transcription coregulator activity  GO:0005102 signaling receptor binding  GO:0005125 cytokine activity  GO:0005178 integrin binding  GO:0005515 protein binding  GO:0008083 growth factor activity | GO:0030296 protein tyrosine kinase activator activity  GO:0030297 transmembrane receptor protein tyrosine kinase activator activity  GO:0030971 receptor tyrosine kinase binding  GO:0043125 ErbB-3 class receptor binding  GO:0045499 chemorepellent activity |  |
| Neuregulin-2 (cleaved pro-neuregulin-2, membrane-bound isoform) | GO:0005102 signaling receptor binding | GO:0008083 growth factor activity |  |
| Neuregulin-3 (cleaved pro-neuregulin-3, membrane-bound isoform) | GO:0005102 signaling receptor binding  GO:0008083 growth factor activity  GO:0030297 transmembrane receptor protein tyrosine kinase activator activity | GO:0030971 receptor tyrosine kinase binding  GO:0045499 chemorepellent activity |  |
| Neurofibromin | GO:0005096 GTPase activator activity  GO:0005515 protein binding  GO:0008289 lipid binding | GO:0008429 phosphatidylethanolamine binding  GO:0031210 phosphatidylcholine binding |  |
| Neurogenic differentiation factor 1 | GO:0000978 RNA polymerase II cis-regulatory region sequence-specific DNA binding  GO:0000981 DNA-binding transcription factor activity, RNA polymerase II-specific  GO:0001102 RNA polymerase II activating transcription factor binding  GO:0001228 DNA-binding transcription activator activity, RNA polymerase II-specific  GO:0003677 DNA binding  GO:0003682 chromatin binding  GO:0003690 double-stranded DNA binding  GO:0003700 DNA-binding transcription factor activity | GO:0005515 protein binding  GO:0008134 transcription factor binding  GO:0043565 sequence-specific DNA binding  GO:0046982 protein heterodimerization activity  GO:0046983 protein dimerization activity  GO:0070888 E-box binding  GO:1990837 sequence-specific double-stranded DNA binding |  |
| Neuronal pentraxin-1 | GO:0046872 metal ion binding | |  |
| Neuropeptide Y (cleaved form pro-neuropeptide Y) | GO:0001664 G protein-coupled receptor binding  GO:0004930 G protein-coupled receptor activity  GO:0005102 signaling receptor binding  GO:0005179 hormone activity | GO:0005184 neuropeptide hormone activity  GO:0005246 calcium channel regulator activity  GO:0005515 protein binding  GO:0031841 neuropeptide Y receptor binding |  |
| Neurosecretory protein VGF | GO:0003674 molecular_function  GO:0005179 hormone activity | GO:0005184 neuropeptide hormone activity  GO:0008083 growth factor activity |  |
| Neuroserpin | GO:0004867 serine-type endopeptidase inhibitor activity | GO:0030414 peptidase inhibitor activity |  |
| Neurturin | GO:0005102 signaling receptor binding  GO:0008083 growth factor activity | GO:0030116 glial cell-derived neurotrophic factor receptor binding  GO:0030971 receptor tyrosine kinase binding |  |
| Neutrophil collagenase | GO:0004175 endopeptidase activity  GO:0004222 metalloendopeptidase activity  GO:0004252 serine-type endopeptidase activity  GO:0008233 peptidase activity | GO:0008237 metallopeptidase activity  GO:0008270 zinc ion binding  GO:0016787 hydrolase activity  GO:0046872 metal ion binding |  |
| Neutrophil-activating peptide 2  (cleaved from Platelet basic protein) | GO:0005125 cytokine activity  GO:0005355 glucose transmembrane transporter activity  GO:0005515 protein binding | GO:0008009 chemokine activity  GO:0008083 growth factor activity  GO:0045236 CXCR chemokine receptor binding |  |
| Non-receptor tyrosine-protein kinase TYK2 | GO:0000166 nucleotide binding  GO:0004672 protein kinase activity  GO:0004713 protein tyrosine kinase activity  GO:0004714 transmembrane receptor protein tyrosine kinase activity  GO:0004715 non-membrane spanning protein tyrosine kinase activity  GO:0005131 growth hormone receptor binding | GO:0005515 protein binding  GO:0005524 ATP binding  GO:0016301 kinase activity  GO:0016740 transferase activity  GO:0031702 type 1 angiotensin receptor binding |  |
| Nucleoside diphosphate kinase A | GO:0000166 nucleotide binding  GO:0000287 magnesium ion binding  GO:0000977 RNA polymerase II transcription regulatory region sequence-specific DNA binding  GO:0003697 single-stranded DNA binding  GO:0003723 RNA binding  GO:0004536 deoxyribonuclease activity  GO:0004550 nucleoside diphosphate kinase activity  GO:0005515 protein binding  GO:0005524 ATP binding | GO:0005525 GTP binding  GO:0016301 kinase activity  GO:0016740 transferase activity  GO:0019215 intermediate filament binding  GO:0019899 enzyme binding  GO:0042802 identical protein binding  GO:0043015 gamma-tubulin binding  GO:0043024 ribosomal small subunit binding  GO:0046872 metal ion binding |  |
| Orexin receptor type 1 | GO:0004930 G protein-coupled receptor activity  GO:0016499 orexin receptor activity | GO:0017046 peptide hormone binding |  |
| OX-2 membrane glycoprotein | GO:0005515 protein binding  GO:0086080 protein binding involved in heterotypic cell-cell adhesion | GO:0140081 glycosylated region protein binding |  |
| Pentraxin-related protein PTX3 | GO:0001849 complement component C1q complex binding  GO:0001872 (1->3)-beta-D-glucan binding | GO:0005515 protein binding  GO:0042802 identical protein binding GO:0046790 virion binding |  |
| Peptide YY | GO:0001664 G protein-coupled receptor binding  GO:0005179 hormone activity  GO:0005184 neuropeptide hormone activity | GO:0005515 protein binding  GO:0031841 neuropeptide Y receptor binding |  |
| Periostin | GO:0005201 extracellular matrix structural constituent  GO:0005515 protein binding | GO:0008201 heparin binding GO:0046872 metal ion binding GO:0050839 cell adhesion molecule binding |  |
| Phosphatidylinositol 3-kinase regulatory subunit beta | GO:0001784 phosphotyrosine residue binding  GO:0005515 protein binding  GO:0019903 protein phosphatase binding  GO:0030971 receptor tyrosine kinase binding | GO:0046935 1-phosphatidylinositol-3-kinase regulator activity  GO:0046982 protein heterodimerization activity |  |
| Phosphoglycerate Kinase 1 | GO:0000166 nucleotide binding  GO:0004618 phosphoglycerate kinase activity GO:0005515 protein binding GO:0005524 ATP binding | GO:0016301 kinase activity GO:0016740 transferase activity GO:0043531 ADP binding GO:0047134 protein-disulfide reductase activity |  |
| Plakophilin-1 | GO:0005515 protein binding GO:0005521 lamin binding GO:0019215 intermediate filament binding | GO:0030280 structural constituent of skin epidermis GO:0045296 cadherin binding |  |
| Plasma protease C1 inhibitor | GO:0004867 serine-type endopeptidase inhibitor activity  GO:0005515 protein binding | GO:0030414 peptidase inhibitor activity |  |
| Platelet endothelial cell adhesion molecule | GO:0005515 protein binding | GO:0042803 protein homodimerization activity |  |
| Platelet glycoprotein 4 | GO:0001540 amyloid-beta binding  GO:0005041 low-density lipoprotein particle receptor activity  GO:0005044 scavenger receptor activity  GO:0005324 long-chain fatty acid transporter activity  GO:0005515 protein binding  GO:0008035 high-density lipoprotein particle binding  GO:0008289 lipid binding  GO:0015636 short-chain fatty acid transmembrane transporter activity  GO:0030169 low-density lipoprotein particle binding | GO:0035325 Toll-like receptor binding  GO:0044877 protein-containing complex binding  GO:0050431 transforming growth factor beta binding  GO:0070053 thrombospondin receptor activity  GO:0070892 lipoteichoic acid immune receptor activity  GO:0071813 lipoprotein particle binding  GO:0150025 oxidised low-density lipoprotein particle receptor activity  GO:1901480 oleate transmembrane transporter activity |  |
| Platelet-derived growth factor D | GO:0005161 platelet-derived growth factor receptor binding  GO:0008083 growth factor activity | GO:0070851 growth factor receptor binding |  |
| Platelet-derived growth factor receptor alpha | GO:0000166 nucleotide binding  GO:0004672 protein kinase activity GO:0004713 protein tyrosine kinase activity  GO:0004714 transmembrane receptor protein tyrosine kinase activity  GO:0005018 platelet-derived growth factor alpha-receptor activity  GO:0005021 vascular endothelial growth factor-activated receptor activity  GO:0005161 platelet-derived growth factor receptor binding | GO:0005515 protein binding GO:0005524 ATP binding  GO:0016301 kinase activity GO:0016740 transferase activity GO:0019838 growth factor binding GO:0038085 vascular endothelial growth factor binding GO:0042803 protein homodimerization activity GO:0044877 protein-containing complex binding  GO:0048407 platelet-derived growth factor binding |  |
| Platelet-derived growth factor receptor beta | GO:0000166 nucleotide binding  GO:0004672 protein kinase activity GO:0004713 protein tyrosine kinase activity  GO:0004714 transmembrane receptor protein tyrosine kinase activity  GO:0004992 platelet activating factor receptor activity GO:0005017 platelet-derived growth factor-activated receptor activity  GO:0005019 platelet-derived growth factor beta-receptor activity  GO:0005102 signaling receptor binding | GO:0005161 platelet-derived growth factor receptor binding  GO:0005515 protein binding GO:0005524 ATP binding  GO:0016301 kinase activity  GO:0016740 transferase activity GO:0019838 growth factor binding GO:0019899 enzyme binding  GO:0019901 protein kinase binding GO:0038085 vascular endothelial growth factor binding GO:0043548 phosphatidylinositol 3-kinase binding  GO:0048407 platelet-derived growth factor binding |  |
| Platelet-derived growth factor subunit B | GO:0005161 platelet-derived growth factor receptor binding  GO:0005515 protein binding  GO:0005518 collagen binding GO:0008083 growth factor activity GO:0016176 superoxide-generating NADPH oxidase activator activity | GO:0042056 chemoattractant activity  GO:0042802 identical protein binding GO:0042803 protein homodimerization activity GO:0046982 protein heterodimerization activity GO:0048407 platelet-derived growth factor binding GO:0070851 growth factor receptor binding |  |
| Polyubiquitin-B | GO:0005515 protein binding  GO:0031386 protein tag | GO:0031625 ubiquitin protein ligase binding |  |
| Probetacellulin | GO:0005154 epidermal growth factor receptor binding  GO:0005515 protein binding | GO:0008083 growth factor activity |  |
| Pro-epidermal growth factor | GO:0005088 Ras guanyl-nucleotide Exchange factor activity  GO:0005154 epidermal growth factor receptor binding  GO:0005509 calcium ion binding  GO:0005515 protein binding | | GO:0008083 growth factor activity GO:0017147 Wnt-protein binding GO:0030297 transmembrane receptor protein tyrosine kinase activator activity GO:0042813 Wnt-activated receptor activity |
| Progesterone receptor | GO:0000978 RNA polymerase II cis-regulatory region sequence-specific DNA binding  GO:0000981 DNA-binding transcription factor activity, RNA polymerase II-specific  GO:0001225 RNA polymerase II transcription coactivator binding  GO:0001228 DNA-binding transcription activator activity, RNA polymerase II-specific  GO:0003677 DNA binding  GO:0003700 DNA-binding transcription factor activity  GO:0003707 steroid hormone receptor activity  GO:0004879 nuclear receptor activity  GO:0005102 signaling receptor binding | GO:0005496 steroid binding  GO:0005515 protein binding  GO:0008270 zinc ion binding  GO:0008289 lipid binding  GO:0019899 enzyme binding  GO:0042802 identical protein binding  GO:0043565 sequence-specific DNA binding  GO:0046872 metal ion binding  GO:0051117 ATPase binding |  |
| pro-Glucagon | GO:0005102 signaling receptor binding  GO:0005179 hormone activity  GO:0005515 protein binding | GO:0031769 glucagon receptor binding  GO:0042802 identical protein binding |  |
| Progranulin | GO:0003723 RNA binding  GO:0005125 cytokine activity  GO:0005515 protein binding | GO:0008083 growth factor activity  GO:0051087 chaperone binding |  |
| Proheparin-binding EGF-like growth factor | GO:0005154 epidermal growth factor receptor binding  GO:0005515 protein binding | GO:0008083 growth factor activity  GO:0008201 heparin binding |  |
| Prokineticin-1 | GO:0001664 G protein-coupled receptor binding | GO:0008083 growth factor activity |  |
| ProSAAS | GO:0004866 endopeptidase inhibitor activity  GO:0004867 serine-type endopeptidase inhibitor activity | GO:0005102 signaling receptor binding |  |
| Prostaglandin D2 receptor 2 | GO:0001785 prostaglandin J receptor activity  GO:0004930 G protein-coupled receptor activity  GO:0004956 prostaglandin D receptor activity | GO:0004958 prostaglandin F receptor activity  GO:0042277 peptide binding  GO:0042923 neuropeptide binding |  |
| Protein AMBP | GO:0004867 serine-type endopeptidase inhibitor activity  GO:0005515 protein binding  GO:0019855 calcium channel inhibitor activity  GO:0019862 IgA binding | GO:0020037 heme binding  GO:0030414 peptidase inhibitor activity  GO:0042803 protein homodimerization activity  GO:0046904 calcium oxalate binding |  |
| Protein FAM3B | GO:0005125 cytokine activity | GO:0005515 protein binding |  |
| Protein S100-A6 | GO:0005509 calcium ion binding  GO:0005515 protein binding  GO:0005523 tropomyosin binding  GO:0008270 zinc ion binding  GO:0015075 ion transmembrane transporter activity | GO:0042803 protein homodimerization activity  GO:0044548 S100 protein binding  GO:0046872 metal ion binding  GO:0048306 calcium-dependent protein binding |  |
| Protein S100-A8 | GO:0005509 calcium ion binding  GO:0005515 protein binding  GO:0008017 microtubule binding  GO:0008270 zinc ion binding  GO:0035662 Toll-like receptor 4 binding | GO:0046872 metal ion binding  GO:0048306 calcium-dependent protein binding  GO:0050544 arachidonic acid binding  GO:0050786 RAGE receptor binding |  |
| Protein S100-A10 | GO:0005509 calcium ion binding  GO:0005515 protein binding  GO:0008289 lipid binding | GO:0042803 protein homodimerization activity  GO:0044325 ion channel binding  GO:0048306 calcium-dependent protein binding |  |
| Protein S100-A12 | GO:0005507 copper ion binding  GO:0005509 calcium ion binding  GO:0005515 protein binding  GO:0008270 zinc ion binding | GO:0046872 metal ion binding  GO:0048306 calcium-dependent protein binding  GO:0050786 RAGE receptor binding |  |
| Pro-epidermal growth factor | GO:0005088 Ras guanyl-nucleotide Exchange factor activity  GO:0005154 epidermal growth factor receptor binding  GO:0005509 calcium ion binding  GO:0005515 protein binding | GO:0008083 growth factor activity GO:0017147 Wnt-protein binding GO:0030297 transmembrane receptor protein tyrosine kinase activator activity GO:0042813 Wnt-activated receptor activity |  |
| Protein Wnt-5a | GO:0003700 DNA-binding transcription factor activity  GO:0005102 signaling receptor binding  GO:0005109 frizzled binding  GO:0005115 receptor tyrosine kinase-like orphan receptor binding GO:0005125 cytokine activity | GO:0005515 protein binding  GO:0005543 phospholipid binding GO:0019904 protein domain specific binding GO:0044212 transcription regulatory region sequence-specific DNA binding GO:0048018 receptor ligand activity GO:1902379 chemoattractant activity involved in axon guidance |  |
| Protein Wnt-5b | GO:0005102 signaling receptor binding GO:0005109 frizzled binding | GO:0005125 cytokine activity |  |
| Protein wntless homolog | GO:0005515 protein binding GO:0017147 Wnt-protein binding | GO:0031852 mu-type opioid receptor binding |  |
| Protocadherin Fat 1 | GO:0005509 calcium ion binding | GO:0005515 protein binding |  |
| Protocadherin Fat 4 | GO:0005509 calcium ion binding | GO:0005515 protein binding |  |
| Protocadherin gamma-C3 | GO:0005509 calcium ion binding |  |  |
| Protocadherin-7 | GO:0005509 calcium ion binding |  |  |
| Protocadherin-9 | GO:0005509 calcium ion binding |  |  |
| Protocadherin-18 | GO:0005509 calcium ion binding |  |  |
| Proto-oncogene tyrosine-protein kinase receptor Ret | GO:0000166 nucleotide binding  GO:0004672 protein kinase activity  GO:0004713 protein tyrosine kinase activity  GO:0004714 transmembrane receptor protein tyrosine kinase activity  GO:0005509 calcium ion binding | GO:0005515 protein binding  GO:0005524 ATP binding  GO:0016301 kinase activity  GO:0016740 transferase activity  GO:0038023 signaling receptor activity |  |
| P-selectin | GO:0001530 lipopolysaccharide binding  GO:0005509 calcium ion binding  GO:0005515 protein binding  GO:0008201 heparin binding  GO:0030246 carbohydrate binding  GO:0033691 sialic acid binding | GO:0042806 fucose binding  GO:0043208 glycosphingolipid binding  GO:0046872 metal ion binding  GO:0048306 calcium-dependent protein binding  GO:0070492 oligosaccharide binding |  |
| Ras-related protein R-Ras | GO:0000166 nucleotide binding  GO:0003924 GTPase activity  GO:0005515 protein binding | GO:0005525 GTP binding GO:0019003 GDP binding GO:0044877 protein-containing complex binding |  |
| Ras-related protein R-Ras2 | GO:0000166 nucleotide binding GO:0003924 GTPase activity  GO:0005515 protein binding | GO:0005525 GTP binding GO:0016787 hydrolase activity GO:0019003 GDP binding |  |
| Receptor tyrosine-protein kinase erbB-2 | GO:0000166 nucleotide binding  GO:0001042 RNA polymerase I core binding  GO:0004672 protein kinase activity  GO:0004713 protein tyrosine kinase activity  GO:0004714 transmembrane receptor protein tyrosine kinase activity  GO:0004888 transmembrane signaling receptor activity  GO:0005515 protein binding  GO:0005524 ATP binding | GO:0008022 protein C-terminus binding  GO:0016301 kinase activity  GO:0016740 transferase activity  GO:0019838 growth factor binding  GO:0019903 protein phosphatase binding  GO:0042802 identical protein binding  GO:0043125 ErbB-3 class receptor binding  GO:0046982 protein heterodimerization activity |  |
| Receptor tyrosine-protein kinase erbB-4 | GO:0000166 nucleotide binding  GO:0000976 transcription regulatory region sequence-specific DNA binding  GO:0004672 protein kinase activity  GO:0004713 protein tyrosine kinase activity  GO:0004714 transmembrane receptor protein tyrosine kinase activity  GO:0005154 epidermal growth factor receptor binding | GO:0005515 protein binding  GO:0005524 ATP binding  GO:0016301 kinase activity  GO:0016740 transferase activity  GO:0042803 protein homodimerization activity |  |
| Receptor-interacting serine/ threonine-protein kinase 1 | GO:0000166 nucleotide binding  GO:0004672 protein kinase activity  GO:0004674 protein serine/threonine kinase activity  GO:0004706 JUN kinase kinase kinase activity  GO:0005123 death receptor binding  GO:0005515 protein binding  GO:0005524 ATP binding  GO:0016301 kinase activity | GO:0016740 transferase activity  GO:0031625 ubiquitin protein ligase binding  GO:0042802 identical protein binding  GO:0042803 protein homodimerization activity  GO:0044877 protein-containing complex binding  GO:0070513 death domain binding  GO:0106310 protein serine kinase activity  GO:0106311 protein threonine kinase activity |  |
| Receptor-type tyrosine-protein kinase FLT3 | GO:0000166 nucleotide binding  GO:0004672 protein kinase activity  GO:0004713 protein tyrosine kinase activity  GO:0004714 transmembrane receptor protein tyrosine kinase activity  GO:0004896 cytokine receptor activity  GO:0005021 vascular endothelial growth factor-activated receptor activity  GO:0005515 protein binding | GO:0005524 ATP binding  GO:0016301 kinase activity  GO:0016740 transferase activity  GO:0019838 growth factor binding  GO:0035259 glucocorticoid receptor binding  GO:0043621 protein self-association  GO:0044877 protein-containing complex binding |  |
| Receptor-type tyrosine-protein phosphatase delta | GO:0004721 phosphoprotein phosphatase activity  GO:0004725 protein tyrosine phosphatase activity  GO:0005001 transmembrane receptor protein tyrosine phosphatase activity  GO:0005102 signaling receptor binding | GO:0005515 protein binding  GO:0016787 hydrolase activity  GO:0016791 phosphatase activity  GO:0050839 cell adhesion molecule binding |  |
| Rho family-interacting cell polarization regulator 1 | GO:0005515 protein binding | GO:0071889 14-3-3 protein binding |  |
| Rho GTPase-activating protein 1 | GO:0005096 GTPase activator activity  GO:0005515 protein binding GO:0017124 SH3 domain binding | GO:0017137 Rab GTPase binding GO:0045296 cadherin binding |  |
| Rho guanine nucleotide exchange factor 1 | GO:0001664 G protein-coupled receptor binding GO:0003723 RNA binding  GO:0005085 guanyl-nucleotide exchange factor activity | GO:0005089 Rho guanyl-nucleotide exchange factor activity  GO:0005096 GTPase activator activity  GO:0005515 protein binding |  |
| Rho guanine nucleotide exchange factor 7 | GO:0005085 guanyl-nucleotide exchange factor activity | GO:0005515 protein binding  GO:0019901 protein kinase binding |  |
| Rho-associated protein kinase 1 | GO:0000166 nucleotide binding  GO:0004672 protein kinase activity  GO:0004674 protein serine/threonine kinase activity  GO:0005515 protein binding GO:0005524 ATP binding  GO:0016301 kinase activity GO:0016740 transferase activity  GO:0017048 Rho GTPase binding | GO:0017049 GTP-Rho binding GO:0019828 aspartic-type endopeptidase inhibitor activity GO:0046872 metal ion binding GO:0048156 tau protein binding GO:0050321 tau-protein kinase activity  GO:0072518 Rho-dependent protein serine/threonine kinase activity |  |

| Rho-associated protein kinase 2 | GO:0000166 nucleotide binding GO:0003723 RNA binding GO:0004672 protein kinase activity  GO:0004674 protein serine/threonine kinase activity  GO:0005198 structural molecule activity GO:0005515 protein binding GO:0005524 ATP binding  GO:0016301 kinase activity | GO:0016740 transferase activity GO:0017048 Rho GTPase binding GO:0017049 GTP-Rho binding GO:0046872 metal ion binding GO:0048156 tau protein binding GO:0050321 tau-protein kinase activity  GO:0072518 Rho-dependent protein serine/threonine kinase activity |
| --- | --- | --- |
| Rho-related GTP-binding protein RhoB | GO:0000166 nucleotide binding  GO:0003924 GTPase activity GO:0005515 protein binding | GO:0005525 GTP binding GO:0019003 GDP binding |
| Rho-related GTP-binding protein RhoE | GO:0000166 nucleotide binding  GO:0003924 GTPase activity | GO:0005515 protein binding GO:0005525 GTP binding |
| Rho-related GTP-binding protein RhoG | GO:0000166 nucleotide binding  GO:0003924 GTPase activity GO:0005515 protein binding | GO:0005525 GTP binding GO:0019901 protein kinase binding |
| Ribosomal oxygenase 2 | GO:0003714 transcription corepressor activity  GO:0005515 protein binding  GO:0016491 oxidoreductase activity  GO:0016706 2-oxoglutarate-dependent dioxygenase activity  GO:0032453 histone demethylase activity (H3-K4 specific) | GO:0042802 identical protein binding  GO:0046872 metal ion binding  GO:0051213 dioxygenase activity  GO:0051864 histone demethylase activity (H3-K36 specific) |
| Scavenger receptor cysteine-rich type 1 protein M130 | GO:0005044 scavenger receptor activity | GO:0005515 protein binding |
| Sclerostin | GO:0005515 protein binding  GO:0008134 transcription factor binding | GO:0008201 heparin binding  GO:0036122 BMP binding |
| Secreted frizzled-related protein 1 | GO:0004197 cysteine-type endopeptidase activity  GO:0005109 frizzled binding  GO:0005515 protein binding | GO:0008201 heparin binding  GO:0017147 Wnt-protein binding  GO:0042802 identical protein binding |
| Secreted frizzled-related protein 3 | GO:0005515 protein binding | GO:0017147 Wnt-protein binding |
| Serum amyloid A-1 protein | GO:0001664 G protein-coupled receptor binding | GO:0008201 heparin binding |
| Serine/threonine-protein kinase MRCK alpha | GO:0000166 nucleotide binding  GO:0000287 magnesium ion binding  GO:0004672 protein kinase activity  GO:0004674 protein serine/threonine kinase activity GO:0005515 protein binding | GO:0005524 ATP binding GO:0016301 kinase activity GO:0016740 transferase activity  GO:0042802 identical protein binding  GO:0046872 metal ion binding |
| Serine/threonine-protein kinase MRCK beta | GO:0000166 nucleotide binding GO:0000287 magnesium ion binding  GO:0004672 protein kinase activity  GO:0004674 protein serine/threonine kinase activity GO:0005524 ATP binding | GO:0016301 kinase activity GO:0016740 transferase activity GO:0017048 Rho GTPase binding GO:0044877 protein-containing complex binding GO:0046872 metal ion binding |
| Serotransferrin | GO:0005515 protein binding GO:0008198 ferrous iron binding  GO:0008199 ferric iron binding | GO:0034986 iron chaperone activity GO:0046872 metal ion binding  GO:1990459 transferrin receptor binding |
| Sex hormone-binding globulin | GO:0005496 steroid binding  GO:0005497 androgen binding | GO:0005515 protein binding  GO:0008289 lipid binding |
| Sialic acid-binding Ig-like lectin 5 | GO:0005515 protein binding  GO:0030246 carbohydrate binding | GO:0033691 sialic acid binding |
| Sialic acid-binding Ig-like lectin 9 | GO:0005515 protein binding  GO:0030246 carbohydrate binding | GO:0033691 sialic acid binding |
| Signal peptide, CUB and EGF-like domain-containing protein 3 | GO:0005509 calcium ion binding  GO:0005515 protein binding | GO:0042802 identical protein binding |
| Signal transducer CD24 | GO:0005515 protein binding  GO:0019901 protein kinase binding | GO:0030296 protein tyrosine kinase activator activity |
| SLIT-ROBO Rho GTPase-activating protein 1 | GO:0005096 GTPase activator activity  GO:0005515 protein binding | GO:0048365 Rac GTPase binding |
| SLIT-ROBO Rho GTPase-activating protein 2 | GO:0005096 GTPase activator activity  GO:0005515 protein binding GO:0042802 identical protein binding | GO:0042803 protein homodimerization activity  GO:0048365 Rac GTPase binding |
| Solute carrier family 2, facilitated glucose transporter member 1 | GO:0005324 long-chain fatty acid transporter activity  GO:0005355 glucose transmembrane transporter activity  GO:0005515 protein binding  GO:0019900 kinase binding  GO:0022857 transmembrane transporter activity  GO:0033300 dehydroascorbic acid transmembrane transporter activity | GO:0042802 identical protein binding  GO:0042910 xenobiotic transmembrane transporter activity  GO:0043621 protein self-association  GO:0055056 D-glucose transmembrane transporter activity |
| Solute carrier family 2, facilitated glucose transporter member 2 | GO:0005353 fructose transmembrane transporter activity  GO:0005355 glucose transmembrane transporter activity  GO:0022857 transmembrane transporter activity | GO:0033300 dehydroascorbic acid transmembrane transporter activity  GO:0055056 D-glucose transmembrane transporter activity |
| Solute carrier family 2, facilitated glucose transporter member 3 | GO:0005355 glucose transmembrane transporter activity  GO:0005515 protein binding | GO:0005536 glucose binding  GO:0022857 transmembrane transporter activity |
| Solute carrier family 2, facilitated glucose transporter member 5 | GO:0005353 fructose transmembrane transporter activity  GO:0005355 glucose transmembrane transporter activity | GO:0005515 protein binding  GO:0022857 transmembrane transporter activity  GO:0070061 fructose binding |
| Somatotropin | GO:0005131 growth hormone receptor binding  GO:0005148 prolactin receptor binding  GO:0005179 hormone activity | GO:0005515 protein binding  GO:0008083 growth factor activity  GO:0046872 metal ion binding |
| Sonic hedgehog protein | GO:0005113 patched binding  GO:0005509 calcium ion binding  GO:0005515 protein binding  GO:0005539 glycosaminoglycan binding  GO:0008233 peptidase activity | GO:0008270 zinc ion binding  GO:0016015 morphogen activity  GO:0016787 hydrolase activity  GO:0043237 laminin-1 binding  GO:0046872 metal ion binding |
| SPARC | GO:0005201 extracellular matrix structural constituent  GO:0005509 calcium ion binding GO:0005515 protein binding | GO:0005518 collagen binding GO:0046872 metal ion binding  GO:0050840 extracellular matrix binding |
| Sphingosine 1-phosphate receptor 1 | GO:0001664 G protein-coupled receptor binding  GO:0004930 G protein-coupled receptor activity  GO:0005515 protein binding | GO:0038036 sphingosine-1-phosphate receptor activity  GO:0046625 sphingolipid binding |
| Stromal cell-derived factor 1 | GO:0005102 signaling receptor binding  GO:0005125 cytokine activity  GO:0005178 integrin binding  GO:0005515 protein binding | GO:0008009 chemokine activity  GO:0008083 growth factor activity  GO:0042379 chemokine receptor binding  GO:0045236 CXCR chemokine receptor binding |
| Stromelysin-2 | GO:0004222 metalloendopeptidase activity  GO:0008233 peptidase activity  GO:0008237 metallopeptidase activity | GO:0008270 zinc ion binding  GO:0016787 hydrolase activity  GO:0046872 metal ion binding |
| Stromelysin-3 | GO:0004222 metalloendopeptidase activity  GO:0008233 peptidase activity  GO:0008237 metallopeptidase activity | GO:0008270 zinc ion binding  GO:0016787 hydrolase activity  GO:0046872 metal ion binding |
| SWI/SNF-related matrix-associated actin-dependent regulator of chromatin subfamily E member 1 | GO:0003677 DNA binding  GO:0003682 chromatin binding  GO:0003713 transcription coactivator activity  GO:0003723 RNA binding  GO:0005515 protein binding | GO:0008080 N-acetyltransferase activity  GO:0016922 nuclear receptor binding  GO:0031492 nucleosomal DNA binding  GO:0047485 protein N-terminus binding |
| TGF-beta receptor type-2 | GO:0000166 nucleotide binding  GO:0004672 protein kinase activity  GO:0004674 protein serine/threonine kinase activity GO:0004675 transmembrane receptor protein serine/threonine kinase activity  GO:0005026 transforming growth factor beta receptor activity, type II GO:0005024 transforming growth factor beta-activated receptor activity  GO:0005515 protein binding  GO:0005524 ATP binding  GO:0005539 glycosaminoglycan binding GO:0016301 kinase activity | GO:0016740 transferase activity GO:0017002 activin-activated receptor activity GO:0031435 mitogen-activated protein kinase kinase kinase binding  GO:0034713 type I transforming growth factor beta receptor binding  GO:0034714 type III transforming growth factor beta receptor binding GO:0038023 signaling receptor activity  GO:0046332 SMAD binding  GO:0046872 metal ion binding GO:0048185 activin binding  GO:0050431 transforming growth factor beta binding |
| Thioredoxin-interacting protein | GO:0004857 enzyme inhibitor activity  GO:0005515 protein binding | GO:0031625 ubiquitin protein ligase binding |
| Thrombopoietin | GO:0005102 signaling receptor binding  GO:0005125 cytokine activity | GO:0005179 hormone activity  GO:0008083 growth factor activity |
| Thrombospondin-1 | GO:0001786 phosphatidylserine binding GO:0001968 fibronectin binding  GO:0005178 integrin binding GO:0005201 extracellular matrix structural constituent  GO:0005509 calcium ion binding  GO:0005515 protein binding  GO:0008201 heparin binding  GO:0017134 fibroblast growth factor binding | GO:0030169 low-density lipoprotein particle binding GO:0042802 identical protein binding GO:0043236 laminin binding GO:0043394 proteoglycan binding  GO:0050431 transforming growth factor beta binding GO:0050840 extracellular matrix binding GO:0070051 fibrinogen binding GO:0070052 collagen V binding |
| Thrombospondin-2 | GO:0005201 extracellular matrix structural constituent  GO:0005509 calcium ion binding | GO:0005515 protein binding  GO:0008201 heparin binding |
| Thyroid peroxidase | GO:0004447 iodide peroxidase activity  GO:0004601 peroxidase activity  GO:0005509 calcium ion binding | GO:0016491 oxidoreductase activity  GO:0020037 heme binding  GO:0046872 metal ion binding |
| Thyrotropin subunit beta | GO:0005179 hormone activity | |
| T-lymphocyte activation antigen CD80 | GO:0001618 virus receptor activity  GO:0005515 protein binding | GO:0015026 coreceptor activity |
| Toll-like receptor 2 | GO:0001530 lipopolysaccharide binding  GO:0001540 amyloid-beta binding  GO:0001875 lipopolysaccharide immune receptor activity  GO:0003953 NAD+ nucleosidase activity  GO:0004888 transmembrane signaling receptor activity  GO:0005515 protein binding  GO:0016787 hydrolase activity  GO:0035325 Toll-like receptor binding | GO:0038023 signaling receptor activity  GO:0038187 pattern recognition receptor activity  GO:0042497 triacyl lipopeptide binding  GO:0042802 identical protein binding  GO:0042834 peptidoglycan binding  GO:0044877 protein-containing complex binding  GO:0050135 NAD(P)+ nucleosidase activity  GO:0061809 NAD+ nucleotidase, cyclic ADP-ribose generating  GO:0071723 lipopeptide binding |
| Toll-like receptor 4 | GO:0001530 lipopolysaccharide binding  GO:0001540 amyloid-beta binding  GO:0001875 lipopolysaccharide immune receptor activity  GO:0003953 NAD+ nucleosidase activity  GO:0004888 transmembrane signaling receptor activity  GO:0005102 signaling receptor binding  GO:0005515 protein binding | GO:0016787 hydrolase activity  GO:0038023 signaling receptor activity  GO:0042802 identical protein binding  GO:0046982 protein heterodimerization activity  GO:0050135 NAD(P)+ nucleosidase activity  GO:0061809 NAD+ nucleotidase, cyclic ADP-ribose generating |
| Transcription factor SOX-2 | GO:0000976 transcription regulatory region sequence-specific DNA binding  GO:0000978 RNA polymerase II cis-regulatory region sequence-specific DNA binding  GO:0000981 DNA-binding transcription factor activity, RNA polymerase II-specific  GO:0001228 DNA-binding transcription activator activity, RNA polymerase II-specific  GO:0003677 DNA binding | GO:0003700 DNA-binding transcription factor activity  GO:0005515 protein binding  GO:0035198 miRNA binding  GO:0043565 sequence-specific DNA binding |
| Transcription initiation factor TFIID subunit 4 | GO:0001046 core promoter sequence-specific DNA binding  GO:0003677 DNA binding  GO:0005515 protein binding | GO:0016251 RNA polymerase II general transcription initiation factor activity  GO:0017162 aryl hydrocarbon receptor binding  GO:0046982 protein heterodimerization activity |
| Transferrin receptor protein 1 | GO:0001618 virus receptor activity  GO:0003723 RNA binding  GO:0003725 double-stranded RNA binding  GO:0004998 transferrin receptor activity  GO:0005515 protein binding | GO:0019901 protein kinase binding  GO:0042802 identical protein binding  GO:0042803 protein homodimerization activity  GO:0044877 protein-containing complex binding |
| Transforming growth factor alpha (cleaved from Protransforming growth factor alpha) | GO:0005154 epidermal growth factor receptor binding | GO:0005515 protein binding GO:0008083 growth factor activity |
| Transforming growth factor beta receptor type 3 | GO:0005024 transforming growth factor beta-activated receptor activity  GO:0005114 type II transforming growth factor beta receptor binding  GO:0005160 transforming growth factor beta receptor binding  GO:0005515 protein binding  GO:0005539 glycosaminoglycan binding  GO:0008201 heparin binding  GO:0015026 coreceptor activity | GO:0017134 fibroblast growth factor binding  GO:0030165 PDZ domain binding  GO:0046332 SMAD binding  GO:0048185 activin binding  GO:0050431 transforming growth factor beta binding  GO:0070123 transforming growth factor beta receptor activity, type III |
| Transforming growth factor beta-1 (cleaved from Transforming growth factor beta-1 proprotein) | GO:0003823 antigen binding  GO:0005114 type II transforming growth factor beta receptor binding  GO:0005125 cytokine activity  GO:0005160 transforming growth factor beta receptor binding  GO:0005515 protein binding | GO:0008083 growth factor activity  GO:0019899 enzyme binding  GO:0034713 type I transforming growth factor beta receptor binding GO:0034714 type III transforming growth factor beta receptor binding GO:0042802 identical protein binding |
| Transforming growth factor beta-3 (cleaved form Transforming growth factor beta-3 proprotein) | GO:0005114 type II transforming growth factor beta receptor binding  GO:0005125 cytokine activity  GO:0005160 transforming growth factor beta receptor binding GO:0005515 protein binding  GO:0008083 growth factor activity | GO:0034713 type I transforming growth factor beta receptor binding  GO:0034714 type III transforming growth factor beta receptor binding  GO:0042802 identical protein binding GO:0044877 protein-containing complex binding GO:0050431 transforming growth factor beta binding |
| Transforming growth factor-beta-induced protein ig-h3 | GO:0005178 integrin binding  GO:0005201 extracellular matrix structural constituent GO:0005515 protein binding | GO:0005518 collagen binding GO:0050839 cell adhesion molecule binding  GO:0050840 extracellular matrix binding |
| Transforming protein RhoA | GO:0000166 nucleotide binding  GO:0003924 GTPase activity  GO:0005515 protein binding  GO:0005525 GTP binding GO:0016787 hydrolase activity | GO:0017022 myosin binding GO:0019003 GDP binding GO:0019904 protein domain specific binding GO:0051022 Rho GDP-dissociation inhibitor binding |
| Transient receptor potential cation channel subfamily M member 7 | GO:0000166 nucleotide binding  GO:0003779 actin binding  GO:0004674 protein serine/threonine kinase activity  GO:0005216 ion channel activity  GO:0005261 cation channel activity  GO:0005262 calcium channel activity  GO:0005524 ATP binding | GO:0016301 kinase activity  GO:0016740 transferase activity  GO:0017022 myosin binding  GO:0046872 metal ion binding  GO:0106310 protein serine kinase activity  GO:0106311 protein threonine kinase activity |
| Triggering receptor expressed on myeloid cells 1 | GO:0038023 signaling receptor activity  GO:0097110 scaffold protein binding | |
| Troponin C, slow skeletal and cardiac muscles | GO:0005509 calcium ion binding  GO:0005515 protein binding  GO:0031013 troponin I binding  GO:0031014 troponin T binding | GO:0042803 protein homodimerization activity  GO:0046872 metal ion binding  GO:0048306 calcium-dependent protein binding  GO:0051015 actin filament binding |
| Tumor necrosis factor ligand superfamily member 10 | GO:0005102 signaling receptor binding  GO:0005125 cytokine activity  GO:0005164 tumor necrosis factor receptor binding  GO:0005515 protein binding  GO:0008270 zinc ion binding | GO:0032813 tumor necrosis factor receptor superfamily binding  GO:0042802 identical protein binding  GO:0045569 TRAIL binding  GO:0046872 metal ion binding |
| Tumor necrosis factor ligand superfamily member 11 | GO:0005125 cytokine activity  GO:0005164 tumor necrosis factor receptor binding  GO:0005515 protein binding | GO:0032813 tumor necrosis factor receptor superfamily binding  GO:0042802 identical protein binding |
| Tumor necrosis factor ligand superfamily member 13 | GO:0005102 signaling receptor binding GO:0005125 cytokine activity | GO:0005164 tumor necrosis factor receptor binding |
| Tumor necrosis factor ligand superfamily member 15 | GO:0005102 signaling receptor binding  GO:0005125 cytokine activity | GO:0005164 tumor necrosis factor receptor binding  GO:0005515 protein binding |
| Tumor necrosis factor ligand superfamily member 4 | GO:0005102 signaling receptor binding  GO:0005125 cytokine activity  GO:0005164 tumor necrosis factor receptor binding | GO:0005515 protein binding  GO:0032813 tumor necrosis factor receptor superfamily binding |
| Tumor necrosis factor ligand superfamily member 6 | GO:0005102 signaling receptor binding  GO:0005123 death receptor binding  GO:0005125 cytokine activity | GO:0005164 tumor necrosis factor receptor binding  GO:0005515 protein binding |
| Tumor necrosis factor ligand superfamily member 8 | GO:0005102 signaling receptor binding  GO:0005125 cytokine activity | GO:0005164 tumor necrosis factor receptor binding  GO:0005515 protein binding |
| Tumor necrosis factor receptor superfamily member 10A | GO:0002020 protease binding  GO:0005035 death receptor activity  GO:0005515 protein binding  GO:0008134 transcription factor binding | GO:0038023 signaling receptor activity  GO:0042802 identical protein binding  GO:0045569 TRAIL binding |
| Tumor necrosis factor receptor superfamily member 10B | GO:0005515 protein binding  GO:0036463 TRAIL receptor activity | GO:0038023 signaling receptor activity  GO:0045569 TRAIL binding |
| Tumor necrosis factor receptor superfamily member 11B | GO:0005125 cytokine activity GO:0005515 protein binding | GO:0038023 signaling receptor activity |
| Tumor necrosis factor receptor superfamily member 13B | GO:0005515 protein binding  GO:0038023 signaling receptor activity | |
| Tumor necrosis factor receptor superfamily member 13C | GO:0038023 signaling receptor activity |  |
| Tumor necrosis factor receptor superfamily member 6B | GO:0005515 protein binding  GO:0038023 signaling receptor activity | |
| Tumor necrosis factor receptor superfamily member 14 | GO:0001618 virus receptor activity  GO:0005031 tumor necrosis factor-activated receptor activity  GO:0005515 protein binding | GO:0019955 cytokine binding  GO:0031625 ubiquitin protein ligase binding |
| Tumor necrosis factor receptor superfamily member 17 | GO:0005515 protein binding  GO:0038023 signaling receptor activity | |
| Tumor necrosis factor receptor superfamily member 19 | GO:0005031 tumor necrosis factor-activated receptor activity | GO:0005515 protein binding  GO:0038023 signaling receptor activity |
| Tumor necrosis factor receptor superfamily member 25 | GO:0005031 tumor necrosis factor-activated receptor activity  GO:0038023 signaling receptor activity | |
| Tumor necrosis factor receptor superfamily member 27 | GO:0005031 tumor necrosis factor-activated receptor activity  GO:0005515 protein binding | GO:0038023 signaling receptor activity |
| Tumor necrosis factor receptor type 1-associated DEATH domain protein | GO:0005068 transmembrane receptor protein tyrosine kinase adaptor activity  GO:0005164 tumor necrosis factor receptor binding  GO:0005515 protein binding  GO:0019900 kinase binding | GO:0042802 identical protein binding  GO:0044877 protein-containing complex binding  GO:0060090 molecular adaptor activity  GO:0070513 death domain binding |
| Tumor necrosis factor | GO:0000976 transcription regulatory region sequence-specific DNA binding  GO:0002020 protease binding  GO:0005125 cytokine activity | GO:0005164 tumor necrosis factor receptor binding  GO:0005515 protein binding  GO:0042802 identical protein binding |
| Tyrosine-protein kinase ABL1 | GO:0000166 nucleotide binding  GO:0000287 magnesium ion binding  GO:0000400 four-way junction DNA binding  GO:0000405 bubble DNA binding  GO:0001784 phosphotyrosine residue binding  GO:0003677 DNA binding  GO:0003713 transcription coactivator activity  GO:0003785 actin monomer binding  GO:0004515 nicotinate-nucleotide adenylyltransferase activity  GO:0004672 protein kinase activity  GO:0004713 protein tyrosine kinase activity  GO:0004714 transmembrane receptor protein tyrosine kinase activity  GO:0004715 non-membrane spanning protein tyrosine kinase activity  GO:0005080 protein kinase C binding  GO:0005515 protein binding  GO:0005524 ATP binding | GO:0008022 protein C-terminus binding  GO:0016301 kinase activity  GO:0016740 transferase activity  GO:0017124 SH3 domain binding  GO:0019904 protein domain specific binding  GO:0019905 syntaxin binding  GO:0030145 manganese ion binding  GO:0038191 neuropilin binding  GO:0042169 SH2 domain binding  GO:0046872 metal ion binding  GO:0046875 ephrin receptor binding  GO:0051015 actin filament binding  GO:0051019 mitogen-activated protein kinase binding  GO:0070064 proline-rich region binding  GO:0097100 supercoiled DNA binding  GO:1990837 sequence-specific double-stranded DNA binding |
| Tyrosine-protein kinase BTK | GO:0000166 nucleotide binding  GO:0004672 protein kinase activity  GO:0004713 protein tyrosine kinase activity  GO:0004714 transmembrane receptor protein tyrosine kinase activity  GO:0004715 non-membrane spanning protein tyrosine kinase activity  GO:0005515 protein binding | GO:0005524 ATP binding  GO:0005547 phosphatidylinositol-3,4,5-trisphosphate binding  GO:0008289 lipid binding  GO:0016301 kinase activity  GO:0016740 transferase activity  GO:0042802 identical protein binding  GO:0046872 metal ion binding |
| Tyrosine-protein kinase Fer | GO:0000166 nucleotide binding  GO:0004672 protein kinase activity  GO:0004713 protein tyrosine kinase activity  GO:0004714 transmembrane receptor protein tyrosine kinase activity  GO:0004715 non-membrane spanning protein tyrosine kinase activity  GO:0005102 signaling receptor binding  GO:0005154 epidermal growth factor receptor binding | GO:0005515 protein binding  GO:0005524 ATP binding  GO:0008157 protein phosphatase 1 binding  GO:0008289 lipid binding  GO:0016301 kinase activity  GO:0016740 transferase activity |
| Tyrosine-protein kinase FRK | GO:0000166 nucleotide binding  GO:0004672 protein kinase activity  GO:0004713 protein tyrosine kinase activity  GO:0004714 transmembrane receptor protein tyrosine kinase activity  GO:0004715 non-membrane spanning protein tyrosine kinase activity | GO:0005102 signaling receptor binding  GO:0005515 protein binding  GO:0005524 ATP binding  GO:0016301 kinase activity  GO:0016740 transferase activity |
| Tyrosine-protein kinase Fyn | GO:0000166 nucleotide binding  GO:0001664 G protein-coupled receptor binding  GO:0004672 protein kinase activity  GO:0004713 protein tyrosine kinase activity  GO:0004714 transmembrane receptor protein tyrosine kinase activity  GO:0004715 non-membrane spanning protein tyrosine kinase activity  GO:0005102 signaling receptor binding  GO:0005515 protein binding  GO:0005524 ATP binding  GO:0015631 tubulin binding  GO:0016301 kinase activity  GO:0016740 transferase activity  GO:0019899 enzyme binding  GO:0031802 type 5 metabotropic glutamate receptor binding  GO:0042608 T cell receptor binding | GO:0042609 CD4 receptor binding  GO:0042610 CD8 receptor binding  GO:0042802 identical protein binding  GO:0043014 alpha-tubulin binding  GO:0043548 phosphatidylinositol 3-kinase binding  GO:0044325 ion channel binding  GO:0044877 protein-containing complex binding  GO:0046872 metal ion binding  GO:0046875 ephrin receptor binding  GO:0048156 tau protein binding  GO:0050321 tau-protein kinase activity  GO:0051428 peptide hormone receptor binding  GO:0070851 growth factor receptor binding  GO:0097718 disordered domain specific binding |
| Tyrosine-protein kinase HCK | GO:0000166 nucleotide binding  GO:0001784 phosphotyrosine residue binding  GO:0004672 protein kinase activity  GO:0004713 protein tyrosine kinase activity  GO:0004714 transmembrane receptor protein tyrosine kinase activity  GO:0004715 non-membrane spanning protein tyrosine kinase activity | GO:0005102 signaling receptor binding  GO:0005515 protein binding  GO:0005524 ATP binding  GO:0016301 kinase activity  GO:0016740 transferase activity |
| Tyrosine-protein kinase ITK/TSK | GO:0000166 nucleotide binding  GO:0004672 protein kinase activity  GO:0004713 protein tyrosine kinase activity  GO:0004714 transmembrane receptor protein tyrosine kinase activity  GO:0004715 non-membrane spanning protein tyrosine kinase activity | GO:0005515 protein binding  GO:0005524 ATP binding  GO:0016301 kinase activity  GO:0016740 transferase activity  GO:0046872 metal ion binding |
| Tyrosine-protein kinase Lck | GO:0000166 nucleotide binding  GO:0001784 phosphotyrosine residue binding  GO:0004672 protein kinase activity  GO:0004713 protein tyrosine kinase activity  GO:0004714 transmembrane receptor protein tyrosine kinase activity  GO:0004715 non-membrane spanning protein tyrosine kinase activity  GO:0004722 protein serine/threonine phosphatase activity  GO:0005102 signaling receptor binding  GO:0005515 protein binding  GO:0005524 ATP binding  GO:0008022 protein C-terminus binding | GO:0016301 kinase activity  GO:0016740 transferase activity  GO:0019901 protein kinase binding  GO:0019903 protein phosphatase binding  GO:0042169 SH2 domain binding  GO:0042608 T cell receptor binding  GO:0042609 CD4 receptor binding  GO:0042610 CD8 receptor binding  GO:0042802 identical protein binding  GO:0043548 phosphatidylinositol 3-kinase binding  GO:0051117 ATPase binding |
| Tyrosine-protein kinase Lyn | GO:0000166 nucleotide binding  GO:0004672 protein kinase activity  GO:0004713 protein tyrosine kinase activity  GO:0004714 transmembrane receptor protein tyrosine kinase activity  GO:0004715 non-membrane spanning protein tyrosine kinase activity  GO:0005102 signaling receptor binding  GO:0005161 platelet-derived growth factor receptor binding  GO:0005178 integrin binding  GO:0005515 protein binding  GO:0005524 ATP binding  GO:0016301 kinase activity | GO:0016740 transferase activity  GO:0017124 SH3 domain binding  GO:0019899 enzyme binding  GO:0031625 ubiquitin protein ligase binding  GO:0043015 gamma-tubulin binding  GO:0043208 glycosphingolipid binding  GO:0044325 ion channel binding  GO:0044877 protein-containing complex binding  GO:0046875 ephrin receptor binding  GO:0051219 phosphoprotein binding  GO:0140031 phosphorylation-dependent protein binding |

| Tyrosine-protein kinase receptor Tie-1 | GO:0000166 nucleotide binding  GO:0004672 protein kinase activity  GO:0004713 protein tyrosine kinase activity  GO:0004714 transmembrane receptor protein tyrosine kinase activity | GO:0005515 protein binding GO:0005524 ATP binding GO:0016301 kinase activity GO:0016740 transferase activity |
| --- | --- | --- |
| Tyrosine-protein kinase receptor UFO | GO:0000166 nucleotide binding  GO:0001618 virus receptor activity  GO:0001786 phosphatidylserine binding  GO:0004672 protein kinase activity GO:0004713 protein tyrosine kinase activity GO:0004714 transmembrane receptor protein tyrosine kinase activity | GO:0005515 protein binding GO:0005524 ATP binding  GO:0016301 kinase activity GO:0016740 transferase activity GO:0032036 myosin heavy chain binding GO:0043548 phosphatidylinositol 3-kinase binding |
| Tyrosine-protein kinase Tec | GO:0000166 nucleotide binding  GO:0004672 protein kinase activity  GO:0004713 protein tyrosine kinase activity  GO:0004714 transmembrane receptor protein tyrosine kinase activity  GO:0004715 non-membrane spanning protein tyrosine kinase activity  GO:0005515 protein binding | GO:0005524 ATP binding  GO:0005543 phospholipid binding  GO:0008289 lipid binding  GO:0016301 kinase activity  GO:0016740 transferase activity  GO:0046872 metal ion binding |
| Tyrosine-protein kinase TXK | GO:0000166 nucleotide binding  GO:0004672 protein kinase activity  GO:0004713 protein tyrosine kinase activity  GO:0004714 transmembrane receptor protein tyrosine kinase activity  GO:0004715 non-membrane spanning protein tyrosine kinase activity | GO:0005515 protein binding  GO:0005524 ATP binding  GO:0016301 kinase activity  GO:0016740 transferase activity |
| Tyrosine-protein kinase ZAP-70 | GO:0000166 nucleotide binding  GO:0001784 phosphotyrosine residue binding  GO:0004672 protein kinase activity  GO:0004713 protein tyrosine kinase activity  GO:0004714 transmembrane receptor protein tyrosine kinase activity  GO:0004715 non-membrane spanning protein tyrosine kinase activity | GO:0005102 signaling receptor binding  GO:0005515 protein binding  GO:0005524 ATP binding  GO:0016301 kinase activity  GO:0016740 transferase activity |
| Urokinase plasminogen activator surface receptor | GO:0005102 signaling receptor binding  GO:0005515 protein binding GO:0019899 enzyme binding GO:0019904 protein domain specific binding | GO:0030377 urokinase plasminogen activator receptor activity GO:0038023 signaling receptor activity |
| Vascular endothelial growth factor A | GO:0001968 fibronectin binding  GO:0005125 cytokine activity GO:0005161 platelet-derived growth factor receptor binding  GO:0005172 vascular endothelial growth factor receptor binding  GO:0005515 protein binding GO:0008083 growth factor activity GO:0038191 neuropilin binding  GO:0008201 heparin binding | GO:0042056 chemoattractant activity  GO:0042802 identical protein binding GO:0042803 protein homodimerization activity  GO:0043183 vascular endothelial growth factor receptor 1 binding  GO:0043184 vascular endothelial growth factor receptor 2 binding  GO:0048018 receptor ligand activity  GO:0050840 extracellular matrix binding |
| Vascular endothelial growth factor C | GO:0005172 vascular endothelial growth factor receptor binding  GO:0005515 protein binding  GO:0008083 growth factor activity | GO:0042056 chemoattractant activity  GO:0043185 vascular endothelial growth factor receptor 3 binding |
| Vascular endothelial growth factor D | GO:0005161 platelet-derived growth factor receptor binding  GO:0005172 vascular endothelial growth factor receptor binding GO:0005515 protein binding | GO:0008083 growth factor activity GO:0042056 chemoattractant activity  GO:0042802 identical protein binding  GO:0043185 vascular endothelial growth factor receptor 3 binding |
| Vascular endothelial growth factor receptor 1 | GO:0000166 nucleotide binding  GO:0004672 protein kinase activity  GO:0004713 protein tyrosine kinase activity  GO:0004714 transmembrane receptor protein tyrosine kinase activity  GO:0005021 vascular endothelial growth factor-activated receptor activity  GO:0005515 protein binding | GO:0005524 ATP binding  GO:0016301 kinase activity  GO:0016740 transferase activity  GO:0019838 growth factor binding  GO:0036332 placental growth factor-activated receptor activity |
| Vascular endothelial growth factor receptor 2 | GO:0000166 nucleotide binding  GO:0004672 protein kinase activity  GO:0004713 protein tyrosine kinase activity  GO:0004714 transmembrane receptor protein tyrosine kinase activity GO:0005021 vascular endothelial growth factor-activated receptor activity  GO:0005178 integrin binding  GO:0005515 protein binding | GO:0005524 ATP binding GO:0016301 kinase activity GO:0016740 transferase activity  GO:0019838 growth factor binding  GO:0038085 vascular endothelial growth factor binding  GO:0042802 identical protein binding  GO:0045296 cadherin binding GO:0051879 Hsp90 protein binding |

| Vascular endothelial growth factor receptor 3 | GO:0000166 nucleotide binding  GO:0004672 protein kinase activity GO:0004713 protein tyrosine kinase activity  GO:0004714 transmembrane receptor protein tyrosine kinase activity GO:0005021 vascular endothelial growth factor-activated receptor activity GO:0005515 protein binding  GO:0005524 ATP binding | GO:0016301 kinase activity GO:0016740 transferase activity GO:0019838 growth factor binding GO:0019903 protein phosphatase binding GO:0036328 VEGF-C-activated receptor activity GO:0038085 vascular endothelial growth factor binding GO:0042803 protein homodimerization activity |
| --- | --- | --- |
| Vinculin | GO:0002162 dystroglycan binding  GO:0003779 actin binding  GO:0005198 structural molecule activity  GO:0005515 protein binding | GO:0008013 beta-catenin binding GO:0031625 ubiquitin protein ligase binding GO:0045294 alpha-catenin binding  GO:0045296 cadherin binding |
| Vitamin D-binding protein | GO:0003779 actin binding  GO:0005499 vitamin D binding | GO:0090482 vitamin transmembrane transporter activity GO:1902118 calcidiol binding |
| Vitronectin | GO:0005044 scavenger receptor activity  GO:0005178 integrin binding  GO:0005201 extracellular matrix structural constituent  GO:0005515 protein binding | GO:0005518 collagen binding  GO:0008201 heparin binding GO:0030247 polysaccharide binding GO:0042802 identical protein binding GO:0050840 extracellular matrix binding |
| WAP, Kazal, immunoglobulin, Kunitz and NTR domain-containing protein 1 | GO:0004857 enzyme inhibitor activity  GO:0004867 serine-type endopeptidase inhibitor activity  GO:0005515 protein binding | GO:0008191 metalloendopeptidase inhibitor activity  GO:0030414 peptidase inhibitor activity  GO:0048019 receptor antagonist activity  GO:0050431 transforming growth factor beta binding |
| X-linked interleukin-1 receptor accessory protein-like 2 | GO:0003953 NAD+ nucleosidase activity  GO:0004908 interleukin-1 receptor activity  GO:0004910 interleukin-1, type II, blocking receptor activity | GO:0016787 hydrolase activity  GO:0050135 NAD(P)+ nucleosidase activity  GO:0061809 NAD+ nucleotidase, cyclic ADP-ribose generating |
