# Supplementary material for: Extracellular Vesicles from Human Adipose-Derived Mesenchymal Stem Cells: A Review of Common Cargos
Source: Stem Cell Rev Rep. 2021 Apr 26;18(3):854–901. doi: 10.1007/s12015-021-10155-5 (PMC8942954; doi:10.1007/s12015-021-10155-5)
Supplement: Supplementary file 3 — miRNAs detected in human AT-MSC-EVs: gene ontology annotations of molecular functions. (DOC 251 kb) [file 12015_2021_10155_MOESM3_ESM.doc]

| **Table 3S** miRNAs detected in human AT-MSC-EVs: gene ontology annotations of molecular functions | | |
| --- | --- | --- |
| **miRNA** | **Gene ontology terms of molecular functions** | |
| hsa-let-7a-5p | GO:1903231 | mRNA binding involved in posttranscriptional gene silencing |
| hsa-let-7b-5p | GO:1903231 | mRNA binding involved in posttranscriptional gene silencing |
| hsa-let-7c-5p | GO:1903231 | mRNA binding involved in posttranscriptional gene silencing |
| hsa-let-7e-5p | GO:1903231 | mRNA binding involved in posttranscriptional gene silencing |
| hsa-let-7f-5p  (Previous ID: hsa-let-7f) | GO:1903231 | mRNA binding involved in posttranscriptional gene silencing |
| hsa-let-7g-5p | GO:1903231 | mRNA binding involved in posttranscriptional gene silencing |
| hsa-let-7i-5p | GO:1903231 | mRNA binding involved in posttranscriptional gene silencing |
|  | GO:0000993 | RNA polymerase II complex binding |
|  | GO:0003730 | mRNA 3'-UTR binding |
|  | GO:0098847 | sequence-specific single stranded DNA binding |
| hsa-miR-100-3p  (Previous ID: hsa-miR-100*) | GO:1903231 | mRNA binding involved in posttranscriptional gene silencing |
| hsa-miR-101-3p | GO:1903231 | mRNA binding involved in posttranscriptional gene silencing |
|  | GO:0003730 | mRNA 3'-UTR binding |
| hsa-miR-103a-3p | GO:1903231 | mRNA binding involved in posttranscriptional gene silencing |
| hsa-miR-106a-5p  (Previous ID: hsa-miR-106a) | GO:1903231 | mRNA binding involved in posttranscriptional gene silencing |
|  | GO:0003730 | mRNA 3'-UTR binding |
| hsa-miR-106b-5p  (Previous ID:hsa-miR-106b) | GO:1903231 | mRNA binding involved in posttranscriptional gene silencing |
|  | GO:0003730 | mRNA 3'-UTR binding |
| hsa-miR-107 | GO:1903231 | mRNA binding involved in posttranscriptional gene silencing |
| hsa-miR-10a-5p  (Previous ID: hsa-miR-10a) | GO:1903231 | mRNA binding involved in posttranscriptional gene silencing |
| hsa-miR-10b-5p  (Previous ID: hsa-miR-10b) | GO:1903231 | mRNA binding involved in posttranscriptional gene silencing |
| hsa-miR-1207-5p | GO:1903231 | mRNA binding involved in posttranscriptional gene silencing |
| hsa-miR-124-3p | GO:1903231 | mRNA binding involved in posttranscriptional gene silencing |
|  | GO:0008035 | high-density lipoprotein particle binding |
| hsa-miR-125a-5p | GO:1903231 | mRNA binding involved in posttranscriptional gene silencing |
|  | GO:0000993 | RNA polymerase II complex binding |
|  | GO:0003730 | mRNA 3'-UTR binding |
| hsa-miR-125b-1-3p | GO:1903231 | mRNA binding involved in posttranscriptional gene silencing |
| hsa-miR-125b-5p | GO:1903231 | mRNA binding involved in posttranscriptional gene silencing |
| hsa-miR-126-3p | GO:1903231 | mRNA binding involved in posttranscriptional gene silencing |
| hsa-miR-126-5p | GO:0000993 | RNA polymerase II complex binding |
|  | GO:1903231 | mRNA binding involved in posttranscriptional gene silencing |
| hsa-miR-128-3p | GO:1903231 | mRNA binding involved in posttranscriptional gene silencing |
| hsa-miR-129-5p | GO:1903231 | mRNA binding involved in posttranscriptional gene silencing |
| hsa-miR-130a-3p  (Previous ID:hsa-miR-130a) | GO:1903231 | mRNA binding involved in posttranscriptional gene silencing |
|  | GO:0003730 | mRNA 3'-UTR binding |
| hsa-miR-130b-3p  (Previous ID:hsa-miR-130b) | GO:1903231 | mRNA binding involved in posttranscriptional gene silencing |
|  | GO:0003730 | mRNA 3'-UTR binding |
| hsa-miR-132-3p  (Previous ID:hsa-miR-132) | GO:1903231 | mRNA binding involved in posttranscriptional gene silencing |
| hsa-miR-134-5p | GO:1903231 | mRNA binding involved in posttranscriptional gene silencing |
| hsa-miR-135b-5p | GO:1903231 | mRNA binding involved in posttranscriptional gene silencing |
| hsa-miR-136-5p | GO:1903231 | mRNA binding involved in posttranscriptional gene silencing |
| hsa-miR-138-5p | GO:1903231 | mRNA binding involved in posttranscriptional gene silencing |
| hsa-miR-139-5p | GO:0000993 | RNA polymerase II complex binding |
| hsa-miR-140-3p | GO:0000993 | RNA polymerase II complex binding |
| hsa-miR-141-3p  (Previous ID:hsa-miR-141) | GO:1903231 | mRNA binding involved in posttranscriptional gene silencing |
|  | GO:0003730 | mRNA 3'-UTR binding |
| hsa-miR-142-3p | GO:1903231 | mRNA binding involved in posttranscriptional gene silencing |
| hsa-miR-142-5p | GO:0003730 | mRNA 3'-UTR binding |
|  | GO:1903231 | mRNA binding involved in posttranscriptional gene silencing |
| hsa-miR-143-3p | GO:1903231 | mRNA binding involved in posttranscriptional gene silencing |
| hsa-miR-144-3p | GO:1903231 | mRNA binding involved in posttranscriptional gene silencing |
|  | GO:0003730 | mRNA 3'-UTR binding |
| hsa-miR-145-3p  (Previous ID:hsa-miR-145*) | GO:1903231 | mRNA binding involved in posttranscriptional gene silencing |
| hsa-miR-145-5p | GO:1903231 | mRNA binding involved in posttranscriptional gene silencing |
|  | GO:0000993 | RNA polymerase II complex binding |
| hsa-miR-146a-5p | GO:1903231 | mRNA binding involved in posttranscriptional gene silencing |
|  | GO:0003730 | mRNA 3'-UTR binding |
| hsa-miR-146b-5p | GO:1903231 | mRNA binding involved in posttranscriptional gene silencing |
| hsa-miR-148a-3p | GO:1903231 | mRNA binding involved in posttranscriptional gene silencing |
| hsa-miR-148b-3p  (Previous ID:hsa-miR-148b) | GO:1903231 | mRNA binding involved in posttranscriptional gene silencing |
| hsa-miR-148b-5p  (Previous ID:hsa-miR-148b*) | GO:1903231 | mRNA binding involved in posttranscriptional gene silencing |
| hsa-miR-149-3p | GO:1903231 | mRNA binding involved in posttranscriptional gene silencing |
| hsa-miR-149-5p  (Previous ID:hsa-miR-149) | GO:1903231 | mRNA binding involved in posttranscriptional gene silencing |
| hsa-miR-150-5p  (Previous ID:hsa-miR-150) | GO:1903231 | mRNA binding involved in posttranscriptional gene silencing |
| hsa-miR-152-3p | GO:1903231 | mRNA binding involved in posttranscriptional gene silencing |
|  | GO:0003730 | mRNA 3'-UTR binding |
|  | GO:1903231 | mRNA binding involved in posttranscriptional gene silencing |
| hsa-miR-153-3p | GO:1903231 | mRNA binding involved in posttranscriptional gene silencing |
|  | GO:0003730 | mRNA 3'-UTR binding |
| hsa-miR-155-5p | GO:1903231 | mRNA binding involved in posttranscriptional gene silencing |
| hsa-miR-15a-3p  (Previous ID: hsa-miR-15a*) | GO:1903231 | mRNA binding involved in posttranscriptional gene silencing |
| hsa-miR-15a-5p  (Previous ID: hsa-miR-15a) | GO:1903231 | mRNA binding involved in posttranscriptional gene silencing |
|  | GO:0003730 | mRNA 3'-UTR binding |
| hsa-miR-15b-5p | GO:1903231 | mRNA binding involved in posttranscriptional gene silencing |
|  | GO:0003730 | mRNA 3'-UTR binding |
| hsa-miR-16-5p | GO:1903231 | mRNA binding involved in posttranscriptional gene silencing |
|  | GO:0000993 | RNA polymerase II complex binding |
| hsa-miR-17-5p  (Previous ID: hsa-miR-17) | GO:1903231 | mRNA binding involved in posttranscriptional gene silencing |
|  | GO:0003730 | mRNA 3'-UTR binding |
| hsa-miR-181a-5p | GO:1903231 | mRNA binding involved in posttranscriptional gene silencing |
| hsa-miR-181b-5p | GO:1903231 | mRNA binding involved in posttranscriptional gene silencing |
| hsa-miR-181c-5p | GO:1903231 | mRNA binding involved in posttranscriptional gene silencing |
|  | GO:0003727 | single-stranded RNA binding |
| hsa-miR-1825 | GO:0003674 | molecular_function |
| hsa-miR-182-5p  (Previous ID: hsa-miR-182) | GO:1903231 | mRNA binding involved in posttranscriptional gene silencing |
| hsa-miR-183-5p  (Previous ID: hsa-miR-183) | GO:1903231 | mRNA binding involved in posttranscriptional gene silencing |
|  | GO:0003730 | mRNA 3'-UTR binding |
| hsa-miR-185-3p | GO:1903231 | mRNA binding involved in posttranscriptional gene silencing |
| hsa-miR-185-5p  (Previous ID: hsa-miR-185) | GO:1903231 | mRNA binding involved in posttranscriptional gene silencing |
| hsa-miR-18a-5p  (Previous ID: hsa-miR-18a) | GO:1903231 | mRNA binding involved in posttranscriptional gene silencing |
| hsa-miR-18b-5p  (Previous ID: hsa-miR-18b) | GO:1903231 | mRNA binding involved in posttranscriptional gene silencing |
| hsa-miR-1908-5p | GO:1903231 | mRNA binding involved in posttranscriptional gene silencing |
|  | GO:0003730 | mRNA 3'-UTR binding |
| hsa-miR-191-5p  (Previous ID: hsa-miR-191) | GO:1903231 | mRNA binding involved in posttranscriptional gene silencing |
| hsa-miR-192-5p | GO:1903231 | mRNA binding involved in posttranscriptional gene silencing |
| hsa-miR-193a-3p | GO:1903231 | mRNA binding involved in posttranscriptional gene silencing |
|  | GO:0003730 | mRNA 3'-UTR binding |
| hsa-miR-193b-3p  (Previous ID: hsa-miR-193b) | GO:1903231 | mRNA binding involved in posttranscriptional gene silencing |
| hsa-miR-194-5p  (Previous ID: hsa-miR-194) | GO:1903231 | mRNA binding involved in posttranscriptional gene silencing |
| hsa-miR-195-5p  (Previous ID: hsa-miR-195) | GO:1903231 | mRNA binding involved in posttranscriptional gene silencing |
|  | GO:0003730 | mRNA 3'-UTR binding |
| hsa-miR-196a-5p | GO:1903231 | mRNA binding involved in posttranscriptional gene silencing |
| hsa-miR-198 | GO:0003730 | mRNA 3'-UTR binding |
|  | GO:1903231 | mRNA binding involved in posttranscriptional gene silencing |
| hsa-miR-199a-5p | GO:1903231 | mRNA binding involved in posttranscriptional gene silencing |
| hsa-miR-199b-3p /  hsa-miR-199a-3p | GO:1903231 | mRNA binding involved in posttranscriptional gene silencing |
| hsa-miR-199b-5p  (Previous ID: hsa-miR-199b) | GO:1903231 | mRNA binding involved in posttranscriptional gene silencing |
| hsa-miR-19a-3p  (Previous ID: hsa-miR-19a) | GO:1903231 | mRNA binding involved in posttranscriptional gene silencing |
| hsa-miR-19b-3p  (Previous ID: hsa-miR-19b) | GO:1903231 | mRNA binding involved in posttranscriptional gene silencing |
| hsa-miR-200a-3p  (Previous ID: hsa-miR-200a) | GO:1903231 | mRNA binding involved in posttranscriptional gene silencing |
| hsa-miR-200b-3p  (Previous ID: hsa-miR-200b) | GO:1903231 | mRNA binding involved in posttranscriptional gene silencing |
|  | GO:0003730 | mRNA 3'-UTR binding |
| hsa-miR-200c-3p  (Previous ID: hsa-miR-200c) | GO:1903231 | mRNA binding involved in posttranscriptional gene silencing |
|  | GO:0003730 | mRNA 3'-UTR binding |
| hsa-miR-203a-3p | GO:1903231 | mRNA binding involved in posttranscriptional gene silencing |
| hsa-miR-204-5p | GO:1903231 | mRNA binding involved in posttranscriptional gene silencing |
| hsa-miR-205-5p  (Previous ID: hsa-miR-205) | GO:1903231 | mRNA binding involved in posttranscriptional gene silencing |
| hsa-miR-206 | GO:1903231 | mRNA binding involved in posttranscriptional gene silencing |
|  | GO:0003730 | mRNA 3'-UTR binding |
|  | GO:0005515 | protein binding |
| hsa-miR-20a-5p | GO:1903231 | mRNA binding involved in posttranscriptional gene silencing |
|  | GO:0000993 | RNA polymerase II complex binding |
|  | GO:0003730 | mRNA 3'-UTR binding |
| hsa-miR-20b-5p  (Previous ID: hsa-miR-20b) | GO:1903231 | mRNA binding involved in posttranscriptional gene silencing |
|  | GO:0003730 | mRNA 3'-UTR binding |
| hsa-miR-210-5p | GO:1903231 | mRNA binding involved in posttranscriptional gene silencing |
| hsa-miR-21-3p | GO:1903231 | mRNA binding involved in posttranscriptional gene silencing |
| hsa-miR-212-3p  (Previous ID: hsa-miR-212) | GO:1903231 | mRNA binding involved in posttranscriptional gene silencing |
| hsa-miR-214-3p | GO:1903231 | mRNA binding involved in posttranscriptional gene silencing |
|  |  |  |
|  |  |  |
| hsa-miR-21-5p  (Previous ID: hsa-miR-21) | GO:1903231 | mRNA binding involved in posttranscriptional gene silencing |
|  | GO:0003730 | mRNA 3'-UTR binding |
| hsa-miR-218-5p | GO:1903231 | mRNA binding involved in posttranscriptional gene silencing |
| hsa-miR-219a-5p  (Previous ID: hsa-miR-219) | GO:1903231 | mRNA binding involved in posttranscriptional gene silencing |
| hsa-miR-221-3p | GO:1903231 | mRNA binding involved in posttranscriptional gene silencing |
| hsa-miR-222-3p  (Previous ID: hsa-miR-222) | GO:1903231 | mRNA binding involved in posttranscriptional gene silencing |
| hsa-miR-223-3p  (Previous ID: hsa-miR-223) | GO:1903231 | mRNA binding involved in posttranscriptional gene silencing |
| hsa-miR-22-3p | GO:1903231 | mRNA binding involved in posttranscriptional gene silencing |
| hsa-miR-224-5p  (Previous ID: hsa-miR-224) | GO:1903231 | mRNA binding involved in posttranscriptional gene silencing |
| hsa-miR-23a-3p | GO:1903231 | mRNA binding involved in posttranscriptional gene silencing |
| hsa-miR-23b-3p | GO:1903231 | mRNA binding involved in posttranscriptional gene silencing |
| hsa-miR-24-3p | GO:1903231 | mRNA binding involved in posttranscriptional gene silencing |
| hsa-miR-25-3p | GO:1903231 | mRNA binding involved in posttranscriptional gene silencing |
| hsa-miR-25-5p  (Previous ID: hsa-miR-25*) | GO:1903231 | mRNA binding involved in posttranscriptional gene silencing |
| hsa-miR-26a-5p | GO:1903231 | mRNA binding involved in posttranscriptional gene silencing |
| hsa-miR-26b-5p  (Previous ID: hsa-miR-26b) | GO:1903231 | mRNA binding involved in posttranscriptional gene silencing |
| hsa-miR-27a-3p | GO:1903231 | mRNA binding involved in posttranscriptional gene silencing |
|  | GO:0000976 | transcription regulatory region sequence-specific DNA binding |
| hsa-miR-27a-5p | GO:1903231 | mRNA binding involved in posttranscriptional gene silencing |
| hsa-miR-27b-3p | GO:1903231 | mRNA binding involved in posttranscriptional gene silencing |
| hsa-miR-27b-5p  (Previous ID: hsa-miR-27b*) | GO:1903231 | mRNA binding involved in posttranscriptional gene silencing |
| hsa-miR-28-5p  (Previous ID: hsa-miR-28) | GO:1903231 | mRNA binding involved in posttranscriptional gene silencing |
|  | GO:0003730 | mRNA 3'-UTR binding |
| hsa-miR-296-5p | GO:1903231 | mRNA binding involved in posttranscriptional gene silencing |
| hsa-miR-299-5p | GO:1903231 | mRNA binding involved in posttranscriptional gene silencing |
| hsa-miR-29a-3p | GO:1903231 | mRNA binding involved in posttranscriptional gene silencing |
| hsa-miR-29b-3p | GO:1903231 | mRNA binding involved in posttranscriptional gene silencing |
|  | GO:0003730 | mRNA 3'-UTR binding |
| hsa-miR-29c-3p | GO:1903231 | mRNA binding involved in posttranscriptional gene silencing |
| hsa-miR-301a-3p  (Previous ID: hsa-miR-301) | GO:1903231 | mRNA binding involved in posttranscriptional gene silencing |
| hsa-miR-301b-3p  (Previous ID: hsa-miR-301b) | GO:1903231 | mRNA binding involved in posttranscriptional gene silencing |
| hsa-miR-302a-3p  (Previous ID: hsa-miR-302a) | GO:1903231 | mRNA binding involved in posttranscriptional gene silencing |
| hsa-miR-302c-3p  (Previous ID: hsa-miR-302c ) | GO:1903231 | mRNA binding involved in posttranscriptional gene silencing |
| hsa-miR-302d-3p  (Previous ID: hsa-miR-302d) | GO:1903231 | mRNA binding involved in posttranscriptional gene silencing |
| hsa-miR-30a-3p | GO:1903231 | mRNA binding involved in posttranscriptional gene silencing |
|  | GO:0003730 | mRNA 3'-UTR binding |
| hsa-miR-30a-5p | GO:1903231 | mRNA binding involved in posttranscriptional gene silencing |
| hsa-miR-30b-5p  (Previous ID: hsa-miR-30b) | GO:1903231 | mRNA binding involved in posttranscriptional gene silencing |
| hsa-miR-30c-5p | GO:1903231 | mRNA binding involved in posttranscriptional gene silencing |
|  | GO:0008035 | high-density lipoprotein particle binding |
| hsa-miR-30e-5p | GO:1903231 | mRNA binding involved in posttranscriptional gene silencing |
| hsa-miR-31-5p | GO:1903231 | mRNA binding involved in posttranscriptional gene silencing |
|  | GO:0005515 | protein binding |
| hsa-miR-320a-3p | GO:1903231 | mRNA binding involved in posttranscriptional gene silencing |
| hsa-miR-323a-3p  (Previous ID: hsa-miR-323-3p) | GO:0003730 | mRNA 3'-UTR binding |
|  | GO:1903231 | mRNA binding involved in posttranscriptional gene silencing |
| hsa-miR-330-3p  (Previous ID: hsa-miR-330) | GO:1903231 | mRNA binding involved in posttranscriptional gene silencing |
| hsa-miR-338-3p | GO:1903231 | mRNA binding involved in posttranscriptional gene silencing |
| hsa-miR-33a-3p  (Previous ID: hsa-miR-33a*) | GO:0000993 | RNA polymerase II complex binding |
| hsa-miR-33a-5p  (Previous ID: hsa-miR-33a) | GO:1903231 | mRNA binding involved in posttranscriptional gene silencing |
| hsa-miR-342-5p | GO:1903231 | mRNA binding involved in posttranscriptional gene silencing |
| hsa-miR-345-5p  (Previous ID: hsa-miR-345) | GO:1903231 | mRNA binding involved in posttranscriptional gene silencing |
| hsa-miR-34a-5p | GO:1903231 | mRNA binding involved in posttranscriptional gene silencing |
| hsa-miR-361-3p | GO:0003730 | mRNA 3'-UTR binding |
|  | GO:1903231 | mRNA binding involved in posttranscriptional gene silencing |
| hsa-miR-361-5p  (Previous ID: hsa-miR-361) | GO:1903231 | mRNA binding involved in posttranscriptional gene silencing |
| hsa-miR-362-3p | GO:1903231 | mRNA binding involved in posttranscriptional gene silencing |
| hsa-miR-365a-3p  (Previous ID: hsa-miR-365) | GO:1903231 | mRNA binding involved in posttranscriptional gene silencing |
| hsa-miR-374a-5p  (Previous ID: hsa-miR-374) | GO:0003730 | mRNA 3'-UTR binding |
|  | GO:1903231 | mRNA binding involved in posttranscriptional gene silencing |
| hsa-miR-375-3p | GO:0000993 | RNA polymerase II complex binding |
| hsa-miR-378a-3p | GO:1903231 | mRNA binding involved in posttranscriptional gene silencing |
| hsa-miR-384 | GO:1903231 | mRNA binding involved in posttranscriptional gene silencing |
| hsa-miR-409-3p | GO:1903231 | mRNA binding involved in posttranscriptional gene silencing |
| hsa-miR-410-3p | GO:1903231 | mRNA binding involved in posttranscriptional gene silencing |
| hsa-miR-411-5p | GO:1903231 | mRNA binding involved in posttranscriptional gene silencing |
| hsa-miR-423-3p | GO:1903231 | mRNA binding involved in posttranscriptional gene silencing |
| hsa-miR-424-5p  (Previous ID: hsa-miR-424) | GO:1903231 | mRNA binding involved in posttranscriptional gene silencing |
|  | GO:0003730 | mRNA 3'-UTR binding |
| hsa-miR-425-5p | GO:1903231 | mRNA binding involved in posttranscriptional gene silencing |
| hsa-miR-429 | GO:1903231 | mRNA binding involved in posttranscriptional gene silencing |
|  | GO:0003730 | mRNA 3'-UTR binding |
| hsa-miR-449a  (Previous ID: hsa-miR-449) | GO:1903231 | mRNA binding involved in posttranscriptional gene silencing |
| hsa-miR-455-3p | GO:1903231 | mRNA binding involved in posttranscriptional gene silencing |
|  | GO:0003730 | mRNA 3'-UTR binding |
| hsa-miR-455-5p  (Previous ID: hsa-miR-455) | GO:0003730 | mRNA 3'-UTR binding |
|  | GO:1903231 | mRNA binding involved in posttranscriptional gene silencing |
| hsa-miR-483-3p | GO:1903231 | mRNA binding involved in posttranscriptional gene silencing |
| hsa-miR-483-5p | GO:1903231 | mRNA binding involved in posttranscriptional gene silencing |
| hsa-miR-485-5p | GO:1903231 | mRNA binding involved in posttranscriptional gene silencing |
| hsa-miR-488-3p  (Previous ID: hsa-miR-488) | GO:1903231 | mRNA binding involved in posttranscriptional gene silencing |
| hsa-miR-492 | GO:1903231 | mRNA binding involved in posttranscriptional gene silencing |
| hsa-miR-497-5p  (Previous ID: hsa-miR-497) | GO:0003730 | mRNA 3'-UTR binding |
|  | GO:1903231 | mRNA binding involved in posttranscriptional gene silencing |
| hsa-miR-503-5p  (Previous ID: hsa-miR-503) | GO:1903231 | mRNA binding involved in posttranscriptional gene silencing |
| hsa-miR-505-3p  (Previous ID: hsa-miR-505) | GO:1903231 | mRNA binding involved in posttranscriptional gene silencing |
| hsa-miR-518a-3p | GO:0003674 | molecular_function |
| hsa-miR-518b | GO:1903231 | mRNA binding involved in posttranscriptional gene silencing |
| hsa-miR-518d-3p  (Previous ID: hsa-miR-518d) | GO:0003674 | molecular_function |
| hsa-miR-518f-3p  (Previous ID: hsa-miR-518f) | GO:0003674 | molecular_function |
| hsa-miR-519b-3p | GO:1903231 | mRNA binding involved in posttranscriptional gene silencing |
| hsa-miR-520a-3p  (Previous ID: hsa-miR-520a) | GO:1903231 | mRNA binding involved in posttranscriptional gene silencing |
| hsa-miR-520c-3p | GO:1903231 | mRNA binding involved in posttranscriptional gene silencing |
|  | GO:0003730 | mRNA 3'-UTR binding |
| hsa-miR-543 | GO:1903231 | mRNA binding involved in posttranscriptional gene silencing |
| hsa-miR-548c-3p  (Previous ID: hsa-miR-548c) | GO:1903231 | mRNA binding involved in posttranscriptional gene silencing |
| hsa-miR-548d-5p | GO:0003730 | mRNA 3'-UTR binding |
|  | GO:1903231 | mRNA binding involved in posttranscriptional gene silencing |
| hsa-miR-572 | GO:1903231 | mRNA binding involved in posttranscriptional gene silencing |
| hsa-miR-584-5p  (Previous ID: hsa-miR-584) | GO:0000993 | RNA polymerase II complex binding |
| hsa-miR-590-3P | GO:1903231 | mRNA binding involved in posttranscriptional gene silencing |
| hsa-miR-590-5p | GO:1903231 | mRNA binding involved in posttranscriptional gene silencing |
| hsa-miR-638 | GO:1903231 | mRNA binding involved in posttranscriptional gene silencing |
| hsa-miR-639 | GO:1903231 | mRNA binding involved in posttranscriptional gene silencing |
| hsa-miR-654-3p | GO:1903231 | mRNA binding involved in posttranscriptional gene silencing |
| hsa-miR-657 | GO:1903231 | mRNA binding involved in posttranscriptional gene silencing |
| hsa-miR-659-3p  (Previous ID: hsa-miR-659) | GO:1903231 | mRNA binding involved in posttranscriptional gene silencing |
| hsa-miR-661 | GO:1903231 | mRNA binding involved in posttranscriptional gene silencing |
| hsa-miR-663a | GO:1903231 | mRNA binding involved in posttranscriptional gene silencing |
| hsa-miR-665 | GO:1903231 | mRNA binding involved in posttranscriptional gene silencing |
|  | GO:0003727 | single-stranded RNA binding |
| hsa-miR-675-5p | GO:1903231 | mRNA binding involved in posttranscriptional gene silencing |
| hsa-miR-6869-5p | GO:1903231 | mRNA binding involved in posttranscriptional gene silencing |
| hsa-miR-708-5p  (Previous ID: hsa-miR-708) | GO:0003730 | mRNA 3'-UTR binding |
|  | GO:1903231 | mRNA binding involved in posttranscriptional gene silencing |
| hsa-miR-758-3p  (Previous ID: hsa-miR-758) | GO:1903231 | mRNA binding involved in posttranscriptional gene silencing |
| hsa-miR-892b | GO:1903231 | mRNA binding involved in posttranscriptional gene silencing |
| hsa-miR-92a-3p | GO:1903231 | mRNA binding involved in posttranscriptional gene silencing |
| hsa-miR-935 | GO:1903231 | mRNA binding involved in posttranscriptional gene silencing |
| hsa-miR-93-5p | GO:1903231 | mRNA binding involved in posttranscriptional gene silencing |
|  | GO:0003730 | mRNA 3'-UTR binding |
| hsa-miR-939-5p  (Previous ID: hsa-miR-939) | GO:1903231 | mRNA binding involved in posttranscriptional gene silencing |
| hsa-miR-9-3p  (Previous ID: hsa-miR-9*) | GO:0003730 | mRNA 3'-UTR binding |
|  | GO:1903231 | mRNA binding involved in posttranscriptional gene silencing |
| hsa-miR-9-5p | GO:0003727 | single-stranded RNA binding |
|  | GO:0008035 | high-density lipoprotein particle binding |
|  | GO:1903231 | mRNA binding involved in posttranscriptional gene silencing |
| hsa-miR-98-5p | GO:1903231 | mRNA binding involved in posttranscriptional gene silencing |
|  | GO:0003730 | mRNA 3'-UTR binding |
| hsa-miR-99a-5p | GO:1903231 | mRNA binding involved in posttranscriptional gene silencing |
