# Supplementary material for: Extracellular Vesicles from Human Adipose-Derived Mesenchymal Stem Cells: A Review of Common Cargos
Source: Stem Cell Rev Rep. 2021 Apr 26;18(3):854–901. doi: 10.1007/s12015-021-10155-5 (PMC8942954; doi:10.1007/s12015-021-10155-5)
Supplement: Supplementary file 4 — miRNAs detected in human AT-MSC-EVs: gene ontology annotations of biological processes. (DOC 1.87 mb) [file 12015_2021_10155_MOESM4_ESM.doc]

| **Table 4S** miRNAs detected in human AT-MSC-EVs: gene ontology annotations of biological processes |
| --- |
| **GO:0000320 re-entry into mitotic cell cycle** |
| hsa-miR-199b-3p / hsa-miR-199a-3p |
| **GO:0001569 branching involved in blood vessel morphogenesis** |
| hsa-miR-15b-5p |
| hsa-miR-16-5p |
| **GO:0001678 cellular glucose homeostasis** |
| hsa-miR-103a-3p |
| **GO:0001774 microglial cell activation** |
| hsa-miR-181c-5p |
| **GO:0001818 negative regulation of cytokine production** |
| hsa-miR-155-5p |
| hsa-miR-200c-3p (Previous ID: hsa-miR-200c) |
| **GO:0001819 positive regulation of cytokine production** |
| hsa-miR-182-5p (Previous ID: hsa-miR-182) |
| **GO:0001934 positive regulation of protein phosphorylation** |
| hsa-miR-125b-1-3p |
| hsa-miR-128-3p |
| hsa-miR-21-5p (Previous ID: hsa-miR-21) |
| hsa-miR-30b-5p (Previous ID: hsa-miR-30b) |
| **GO:0001937 negative regulation of endothelial cell proliferation** |
| hsa-miR-146a-5p |
| hsa-miR-16-5p |
| hsa-miR-21-5p (Previous ID: hsa-miR-21) |
| hsa-miR-22-3p |
| hsa-miR-424-5p (Previous ID: hsa-miR-424) |
| hsa-miR-503-5p (Previous ID: hsa-miR-503) |
| hsa-miR-92a-3p |
| **GO:0001953 negative regulation of cell-matrix adhesion** |
| hsa-miR-192-5p |
| hsa-miR-29c-3p |
| hsa-miR-939-5p (Previous ID: hsa-miR-939) |
| **GO:0001960 negative regulation of cytokine-mediated signaling pathway** |
| hsa-miR-125a-5p |
| hsa-miR-21-5p (Previous ID: hsa-miR-21) |
| hsa-miR-29b-3p |
| hsa-miR-520c-3p |
| **GO:0002091 negative regulation of receptor internalization** |
| hsa-miR-199a-5p |
| **GO:0002675 positive regulation of acute inflammatory response** |
| hsa-miR-92a-3p |
| **GO:0002696 positive regulation of leukocyte activation** |
| hsa-miR-155-5p |
| **GO:0002718 regulation of cytokine production involved in immune response** |
| hsa-miR-155-5p |
| **GO:0002862 negative regulation of inflammatory response to antigenic stimulus** |
| hsa-miR-19a-3p (Previous ID: hsa-miR-19a) |
| hsa-miR-19b-3p (Previous ID: hsa-miR-19b) |
| hsa-miR-6869-5p |
| **GO:0002931 response to ischemia** |
| hsa-miR-195-5p (Previous ID: hsa-miR-195) |
| **GO:0003085 negative regulation of systemic arterial blood pressure** |
| hsa-miR-17-5p (Previous ID: hsa-miR-17) |
| **GO:0003151 outflow tract morphogenesis** |
| hsa-miR-17-5p (Previous ID: hsa-miR-17) |
| hsa-miR-20a-5p |
| **GO:0003245 cardiac muscle tissue growth involved in heart morphogenesis** |
| hsa-miR-195-5p (Previous ID: hsa-miR-195) |
|  |
|  |
| **GO:0003300 cardiac muscle hypertrophy** |
| hsa-miR-15b-5p |
| hsa-miR-195-5p (Previous ID: hsa-miR-195) |
| **GO:0006469 negative regulation of protein kinase activity** |
| hsa-miR-20a-5p |
| **GO:0006919 activation of cysteine-type endopeptidase activity involved in apoptotic process** |
| hsa-miR-15a-3p (Previous ID: hsa-miR-15a*) |
| **GO:0006940 regulation of smooth muscle contraction** |
| hsa-miR-143-3p |
| hsa-miR-145-5p |
| **GO:0006974 cellular response to DNA damage stimulus** |
| hsa-miR-193a-3p |
| hsa-miR-34a-5p |
| **GO:0007162 negative regulation of cell adhesion** |
| hsa-miR-138-5p |
| hsa-miR-675-5p |
| hsa-miR-9-5p |
| **GO:0007179 transforming growth factor beta receptor signaling pathway** |
| hsa-miR-183-5p (Previous ID: hsa-miR-183) |
| hsa-miR-21-5p (Previous ID: hsa-miR-21) |
| hsa-miR-212-3p (Previous ID: hsa-miR-212) |
| hsa-miR-26a-5p |
| hsa-miR-30a-3p |
| **GO:0007204 positive regulation of cytosolic calcium ion concentration** |
| hsa-miR-199a-5p |
| **GO:0007566 embryo implantation** |
| hsa-miR-21-5p (Previous ID: hsa-miR-21) |
| **GO:0008016 regulation of heart contraction** |
| hsa-miR-92a-3p |
| **GO:0008150 biological process** |
| hsa-miR-1825 |
| hsa-miR-518a-3p |
| hsa-miR-518d-3p (Previous ID: hsa-miR-518d) |
| hsa-miR-518f-3p (Previous ID: hsa-miR-518f) |
| **GO:0008284 positive regulation of cell population proliferation** |
| hsa-miR-1290 |
| hsa-miR-132-3p (Previous ID: hsa-miR-132) |
| hsa-miR-141-3p (Previous ID: hsa-miR-141) |
| hsa-miR-19b-3p (Previous ID: hsa-miR-19b Ragni 2019) |
| hsa-miR-200b-3p (Previous ID: hsa-miR-200b) |
| hsa-miR-21-5p (Previous ID: hsa-miR-21 Chen 2019, Mayo 2019) |
| hsa-miR-520a-3p (Previous ID: hsa-miR-520a) |
| hsa-miR-590-5p |
| hsa-miR-657 |
| **GO:0008285 negative regulation of cell population proliferation** |
| hsa-miR-138-5p |
| hsa-miR-15a-5p (Previous ID: hsa-miR-15a) |
| hsa-miR-15b-5p |
| hsa-miR-16-5p |
| hsa-miR-195-5p (Previous ID: hsa-miR-195) |
| hsa-miR-204-5p |
| hsa-miR-21-3p |
| hsa-miR-214-3p |
| hsa-miR-21-5p (Previous ID: hsa-miR-21) |
| hsa-miR-218-5p |
| hsa-miR-221-3p |
| hsa-miR-26a-5p |
| hsa-miR-29a-3p |
| hsa-miR-29b-3p |
| hsa-miR-29c-3p |
| hsa-miR-320a-3p |
| hsa-miR-378a-3p |
| hsa-miR-449a (Previous ID: hsa-miR-449) |
| hsa-miR-518b |
| hsa-miR-892b |
| hsa-miR-93-5p |
| hsa-miR-9-5p |
| hsa-miR-98-5p |
| **GO:0008360 regulation of cell shape** |
| hsa-miR-21-5p (Previous ID: hsa-miR-21) |
| **GO:0009749 response to glucose** |
| hsa-miR-221-3p |
| **GO:0009968 negative regulation of signal transduction** |
| hsa-miR-181b-5p |
| **GO:0010459 negative regulation of heart rate** |
| hsa-miR-26a-5p |
| **GO:0010455 positive regulation of cell fate commitment** |
| hsa-miR-206 |
| **GO:0010507 negative regulation of autophagy** |
| hsa-let-7b-5p |
| hsa-miR-199a-5p |
| **GO:0010561 negative regulation of glycoprotein biosynthetic process** |
| hsa-miR-144-3p |
| hsa-miR-520c-3p |
| **GO:0010575 positive regulation of vascular endothelial growth factor production** |
| hsa-miR-132-3p (Previous ID: hsa-miR-132) |
| **GO:0010593 negative regulation of lamellipodium assembly** |
| hsa-miR-196a-5p |
| hsa-miR-214-3p |
| **GO:0010595 positive regulation of endothelial cell migration** |
| hsa-miR-1908-5p |
| hsa-miR-199a-5p |
| hsa-miR-199b-3p / hsa-miR-199a-3p |
| hsa-miR-21-3p |
| hsa-miR-29a-3p |
| **GO:0010596 negative regulation of endothelial cell migration** |
| hsa-miR-21-5p (Previous ID: hsa-miR-21) |
| hsa-miR-503-5p (Previous ID: hsa-miR-503) |
| **GO:0010611 regulation of cardiac muscle hypertrophy** |
| hsa-miR-20a-5p |
| **GO:0010613 positive regulation of cardiac muscle hypertrophy** |
| hsa-miR-155-5p |
| hsa-miR-21-5p (Previous ID: hsa-miR-21) |
| **GO:0010614 negative regulation of cardiac muscle hypertrophy** |
| hsa-miR-145-5p |
| hsa-miR-21-5p (Previous ID: hsa-miR-21) |
| **GO:0010628 positive regulation of gene expression** |
| hsa-miR-101-3p |
| hsa-miR-132-3p (Previous ID: hsa-miR-132) |
| hsa-miR-144-3p |
| hsa-miR-155-5p |
| hsa-miR-182-5p (Previous ID: hsa-miR-182) |
| hsa-miR-20b-5p (Previous ID: hsa-miR-20b) |
| hsa-miR-212-3p (Previous ID: hsa-miR-212) |
| hsa-miR-223-3p (Previous ID: hsa-miR-223) |
| hsa-miR-33b-5p (Previous ID: hsa-miR-33b) |
| hsa-miR-520c-3p |
| hsa-miR-548c-3p (Previous ID: hsa-miR-548c) |
|  |
|  |
| **GO:0010629 negative regulation of gene expression** |
| hsa-miR-101-3p |
| hsa-miR-106b-5p (Previous ID: hsa-miR-106b) |
| hsa-miR-132-3p (Previous ID: hsa-miR-132) |
| hsa-miR-144-3p |
| hsa-miR-146a-5p |
| hsa-miR-155-5p |
| hsa-miR-17-5p (Previous ID: hsa-miR-17) |
| hsa-miR-181a-5p |
| hsa-miR-183-5p (Previous ID: hsa-miR-183) |
| hsa-miR-19b-3p (Previous ID: hsa-miR-19b) |
| hsa-miR-200b-3p (Previous ID: hsa-miR-200b) |
| hsa-miR-206 |
| hsa-miR-20a-5p |
| hsa-miR-212-3p (Previous ID: hsa-miR-212) |
| hsa-miR-21-3p |
| hsa-miR-214-3p |
| hsa-miR-21-5p (Previous ID: hsa-miR-21) |
| hsa-miR-23b-3p |
| hsa-miR-27a-3p |
| hsa-miR-27a-5p |
| hsa-miR-27b-3p |
| hsa-miR-29a-3p |
| hsa-miR-29b-3p |
| hsa-miR-29c-3p |
| hsa-miR-338-3p |
| hsa-miR-33a-5p (Previous ID: hsa-miR-33a) |
| hsa-miR-449a (Previous ID: hsa-miR-449) |
| hsa-miR-492 |
| hsa-miR-520c-3p |
| hsa-miR-548c-3p (Previous ID: hsa-miR-548c) |
| hsa-miR-708-5p (Previous ID: hsa-miR-708) |
| hsa-miR-92a-3p |
| hsa-miR-93-5p |
| hsa-miR-98-5p |
| **GO:0010634 positive regulation of epithelial cell migration** |
| hsa-miR-221-3p |
| hsa-miR-222-3p (Previous ID: hsa-miR-222) |
| **GO:0010641 positive regulation of platelet-derived growth factor receptor signaling pathway** |
| hsa-miR-296-5p |
| **GO:0010666 positive regulation of cardiac muscle cell apoptotic process** |
| hsa-miR-16-5p |
| hsa-miR-17-5p (Previous ID: hsa-miR-17) |
| hsa-miR-195-5p (Previous ID: hsa-miR-195) |
| hsa-miR-320a-3p |
| hsa-miR-34a-5p |
| **GO:0010667 negative regulation of cardiac muscle cell apoptotic process** |
| hsa-miR-106b-5p (Previous ID: hsa-miR-106b) |
| hsa-miR-145-5p |
| hsa-miR-199b-3p / hsa-miR-199a-3p |
| hsa-miR-19a-3p (Previous ID: hsa-miR-19a) |
| hsa-miR-19b-3p (Previous ID: hsa-miR-19b) |
| hsa-miR-20a-5p |
| hsa-miR-21-5p (Previous ID: hsa-miR-21) |
| hsa-miR-24-3p |
| hsa-miR-30e-5p |
| hsa-miR-590-3p |
| **GO:0010668 ectodermal cell differentiation** |
| hsa-miR-145-5p |
|  |
|  |
| **GO:0010716 negative regulation of extracellular matrix disassembly** |
| hsa-miR-98-5p |
| **GO:0010718 positive regulation of epithelial to mesenchymal transition** |
| hsa-miR-21-5p (Previous ID: hsa-miR-21) |
| hsa-miR-221-3p |
| hsa-miR-222-3p (Previous ID: hsa-miR-222) |
| **GO:0010719 negative regulation of epithelial to mesenchymal transition** |
| hsa-miR-144-3p |
| hsa-miR-146b-5p |
| hsa-miR-149-5p (Previous ID: hsa-miR-149) |
| hsa-miR-29b-3p |
| hsa-miR-590-5p |
| **GO:0010744 positive regulation of macrophage derived foam cell differentiation** |
| hsa-miR-185-5p (Previous ID: hsa-miR-185) |
| **GO:0010763 positive regulation of fibroblast migration** |
| hsa-miR-145-5p |
| **GO:0010764 negative regulation of fibroblast migration** |
| hsa-miR-124-3p |
| hsa-miR-146b-5p |
| **GO:0010801 negative regulation of peptidyl-threonine phosphorylation** |
| hsa-miR-103a-3p |
| hsa-miR-15a-5p (Previous ID: hsa-miR-15a) |
| **GO:0010804 negative regulation of tumor necrosis factor-mediated signaling pathway** |
| hsa-miR-152-3p |
| hsa-miR-24-3p |
| hsa-miR-27b-5p (Previous ID: hsa-miR-27b*) |
| **GO:0010812 negative regulation of cell-substrate adhesion** |
| hsa-miR-183-5p (Previous ID: hsa-miR-183) |
| hsa-miR-503-5p (Previous ID: hsa-miR-503) |
| **GO:0010823 negative regulation of mitochondrion organization** |
| hsa-miR-155-5p |
| **GO:0010831 positive regulation of myotube differentiation** |
| hsa-miR-206 |
| **GO:0010832 negative regulation of myotube differentiation** |
| hsa-miR-200b-3p (Previous ID: hsa-miR-200b) |
| **GO:0010838 positive regulation of keratinocyte proliferation** |
| hsa-miR-21-5p (Previous ID: hsa-miR-21) |
| **GO:0010839 negative regulation of keratinocyte proliferation** |
| hsa-miR-125b-5p |
| hsa-miR-181b-5p |
| **GO:0010867 positive regulation of triglyceride biosynthetic process** |
| hsa-miR-29b-3p |
| **GO:0010868 negative regulation of triglyceride biosynthetic process** |
| hsa-miR-30c-5p |
| **GO:0010871negative regulation of receptor biosynthetic process** |
| hsa-let-7e-5p |
| hsa-miR-125a-5p |
| hsa-miR-23b-3p |
| **GO:0010872 regulation of cholesterol esterification** |
| hsa-miR-27a-3p |
| hsa-miR-27b-3p |
| **GO:0010886 positive regulation of cholesterol storage** |
| hsa-miR-144-3p |
| **GO:0010887 negative regulation of cholesterol storage** |
| hsa-miR-146a-5p |
| **GO:0010917 negative regulation of mitochondrial membrane potential** |
| hsa-miR-181b-5p |
|  |
|  |
| **GO:0010940 positive regulation of necrotic cell death** |
| hsa-miR-155-5p |
| hsa-miR-92a-3p |
| **GO:0010942 positive regulation of cell death** |
| hsa-miR-155-5p |
| hsa-miR-34a-5p |
| **GO:0010972 negative regulation of G2/M transition of mitotic cell cycle** |
| hsa-miR-195-5p (Previous ID: hsa-miR-195) |
| hsa-miR-19b-3p (Previous ID: hsa-miR-19b) |
| **GO:0010976 positive regulation of neuron projection development** |
| hsa-miR-200c-3p (Previous ID: hsa-miR-200c) |
| hsa-miR-431-5p (Previous ID: hsa-miR-431) |
| **GO:0010977 negative regulation of neuron projection development** |
| hsa-miR-219a-5p (Previous ID: hsa-miR-219) |
| **GO:0010983 positive regulation of high-density lipoprotein particle clearance** |
| hsa-miR-144-3p |
| hsa-miR-302a-3p (Previous ID: hsa-miR-302a) |
| hsa-miR-33a-5p (Previous ID: hsa-miR-33a) |
| hsa-miR-33b-5p (Previous ID: hsa-miR-33b) |
| **GO:0010985 negative regulation of lipoprotein particle clearance** |
| hsa-miR-128-3p |
| hsa-miR-148a-3p |
| **GO:0010989 negative regulation of low-density lipoprotein particle clearance** |
| hsa-miR-148a-3p |
| hsa-miR-155-5p |
| hsa-miR-17-5p (Previous ID: hsa-miR-17) |
| hsa-miR-185-5p (Previous ID: hsa-miR-185) |
| hsa-miR-199a-5p |
| hsa-miR-27a-3p |
| hsa-miR-27b-3p |
| **GO:0014067 negative regulation of phosphatidylinositol 3-kinase signaling** |
| hsa-miR-206 |
| hsa-miR-20a-5p |
| hsa-miR-449a (Previous ID: hsa-miR-449) |
| **GO:0014068 positive regulation of phosphatidylinositol 3-kinase signaling** |
| hsa-miR-126-3p |
| hsa-miR-21-5p (Previous ID: hsa-miR-21) |
| **GO:0016525 negative regulation of angiogenesis** |
| hsa-miR-106b-5p (Previous ID: hsa-miR-106b) |
| hsa-miR-10a-3p (Previous ID: hsa-miR-10a*) |
| hsa-miR-125a-5p |
| hsa-miR-125b-5p |
| hsa-miR-143-3p |
| hsa-miR-145-5p |
| hsa-miR-146a-5p |
| hsa-miR-15a-5p (Previous ID: hsa-miR-15a) |
| hsa-miR-15b-5p |
| hsa-miR-16-5p |
| hsa-miR-185-5p (Previous ID: hsa-miR-185) |
| hsa-miR-193a-5p |
| hsa-miR-200b-3p (Previous ID: hsa-miR-200b) |
| hsa-miR-20b-5p (Previous ID: hsa-miR-20b) |
| hsa-miR-212-3p (Previous ID: hsa-miR-212) |
| hsa-miR-214-3p |
| hsa-miR-21-5p (Previous ID: hsa-miR-21) |
| hsa-miR-222-3p (Previous ID: hsa-miR-222) |
| hsa-miR-24-3p |
| hsa-miR-29a-3p |
| hsa-miR-30b-5p (Previous ID: hsa-miR-30b) |
| hsa-miR-34a-5p |
| hsa-miR-361-5p (Previous ID: hsa-miR-361) |
| hsa-miR-424-5p (Previous ID: hsa-miR-424) |
| hsa-miR-492 |
| hsa-miR-503-5p (Previous ID: hsa-miR-503) |
| hsa-miR-505-3p (Previous ID: hsa-miR-505) |
| hsa-miR-92a-3p |
| hsa-miR-939-5p (Previous ID: hsa-miR-939) |
| **GO:0030036 actin cytoskeleton organization** |
| hsa-miR-143-3p |
| hsa-miR-145-5p |
| **GO:0030334 regulation of cell migration** |
| hsa-miR-21-5p (Previous ID: hsa-miR-21) |
| **GO:0030335 positive regulation of cell migration** |
| hsa-miR-1290 |
| hsa-miR-181a-5p |
| hsa-miR-181b-5p |
| hsa-miR-182-5p (Previous ID: hsa-miR-182) |
| hsa-miR-21-5p (Previous ID: hsa-miR-21) |
| hsa-miR-29a-3p |
| hsa-miR-29b-3p |
| hsa-miR-590-5p |
| **GO:0030336 negative regulation of cell migration** |
| hsa-let-7g-5p |
| hsa-miR-138-5p |
| hsa-miR-149-5p (Previous ID: hsa-miR-149) |
| hsa-miR-204-5p |
| hsa-miR-214-3p |
| hsa-miR-21-5p (Previous ID: hsa-miR-21) |
| hsa-miR-218-5p |
| hsa-miR-26a-5p |
| hsa-miR-29a-3p |
| hsa-miR-29b-3p |
| hsa-miR-29c-3p |
| hsa-miR-320a-3p |
| hsa-miR-338-3p |
| hsa-miR-34a-5p |
| hsa-miR-449a (Previous ID: hsa-miR-449) |
| hsa-miR-518b |
| hsa-miR-892b |
| hsa-miR-93-5p |
| hsa-miR-9-5p |
| **GO:0030509 BMP signaling pathway** |
| hsa-miR-21-5p (Previous ID: hsa-miR-21) |
| **GO:0030511 positive regulation of transforming growth factor beta receptor signaling pathway** |
| hsa-miR-30b-5p (Previous ID: hsa-miR-30b) |
| **GO:0030512 negative regulation of transforming growth factor beta receptor signaling pathway** |
| hsa-let-7f-5p (Previous ID: hsa-let-7f) |
| hsa-let-7g-5p |
| hsa-miR-15b-5p |
| hsa-miR-29b-3p |
| hsa-miR-342-5p |
| **GO:0030514 negative regulation of BMP signaling pathway** |
| hsa-miR-20a-5p |
| hsa-miR-26a-5p |
| **GO:0030837 negative regulation of actin filament polymerization** |
| hsa-miR-214-3p |
| **GO:0030857 negative regulation of epithelial cell differentiation** |
| hsa-miR-18b-5p (Previous ID: hsa-miR-18b) |
| hsa-miR-518b |
| **GO:0030948 negative regulation of vascular endothelial growth factor receptor signaling pathway** |
| hsa-miR-200c-3p (Previous ID: hsa-miR-200c) |
| **GO:0030949 positive regulation of vascular endothelial growth factor receptor signaling pathway** |
| hsa-miR-10a-5p (Previous ID: hsa-miR-10a) |
| hsa-miR-10b-5p (Previous ID: hsa-miR-10b) |
| hsa-miR-296-5p |
| **GO:0031047 gene silencing by RNA** |
| hsa-miR-181c-5p |
| hsa-miR-665 |
| hsa-miR-9-5p |
| **GO:0031327 negative regulation of cellular biosynthetic process** |
| hsa-miR-520c-3p |
| **GO:0031346 positive regulation of cell projection organization** |
| hsa-miR-21-5p (Previous ID: hsa-miR-21) |
| **GO:0031393 negative regulation of prostaglandin biosynthetic process** |
| hsa-miR-132-3p (Previous ID: hsa-miR-132) |
| hsa-miR-204-5p |
| hsa-miR-766-3p (Previous ID: hsa-miR-766) |
| **GO:0031397 negative regulation of protein ubiquitination** |
| hsa-miR-101-3p |
| hsa-miR-21-5p (Previous ID: hsa-miR-21) |
| **GO:0032007 negative regulation of TOR signaling** |
| hsa-let-7f-5p (Previous ID: hsa-let-7f) |
| **GO:0032008 positive regulation of TOR signaling** |
| hsa-miR-199a-5p |
| **GO:0032088 negative regulation of NF-kappaB transcription factor activity** |
| hsa-miR-138-5p |
| hsa-miR-155-5p |
| hsa-miR-15a-5p (Previous ID: hsa-miR-15a) |
| hsa-miR-16-5p |
| hsa-miR-21-3p |
| hsa-miR-27a-5p |
| **GO:0032148 activation of protein kinase B activity** |
| hsa-miR-143-3p |
| hsa-miR-21-5p (Previous ID: hsa-miR-21) |
| **GO:0032286 central nervous system myelin maintenance** |
| hsa-miR-26a-5p |
| **GO:0032480 negative regulation of type I interferon production** |
| hsa-miR-21-5p (Previous ID: hsa-miR-21) |
| **GO:0032496 response to lipopolysaccharide** |
| hsa-miR-142-3p |
| hsa-miR-155-5p |
| **GO:0032675 regulation of interleukin-6 production** |
| hsa-miR-361-5p (Previous ID: hsa-miR-361) |
| **GO:0032682 negative regulation of chemokine production** |
| hsa-miR-26b-5p (Previous ID: hsa-miR-26b) |
| hsa-miR-590-3p |
| **GO:0032686 negative regulation of hepatocyte growth factor production** |
| hsa-miR-199b-3p / hsa-miR-199a-3p |
| **GO:0032688 negative regulation of interferon-beta production** |
| hsa-miR-26b-5p (Previous ID: hsa-miR-26b) |
| **GO:0032689 negative regulation of interferon-gamma production** |
| hsa-miR-708-5p (Previous ID: hsa-miR-708) |
| **GO:0032690 negative regulation of interleukin-1 alpha production** |
| hsa-miR-142-3p |
| **GO:0032691 negative regulation of interleukin-1 beta production** |
| hsa-miR-101-3p |
| hsa-miR-132-3p (Previous ID: hsa-miR-132) |
| hsa-miR-155-5p |
| hsa-miR-181a-5p |
| hsa-miR-195-5p (Previous ID: hsa-miR-195) |
| hsa-miR-23b-3p |
| hsa-miR-27b-5p (Previous ID: hsa-miR-27b*) |
| hsa-miR-488-3p (Previous ID: hsa-miR-488) |
| hsa-miR-708-5p (Previous ID: hsa-miR-708) |
| hsa-miR-766-3p (Previous ID: hsa-miR-766) |
| hsa-miR-98-5p |
| **GO:0032693 negative regulation of interleukin-10 production** |
| hsa-let-7c-5p |
| hsa-miR-106a-5p (Previous ID: hsa-miR-106a) |
| hsa-miR-194-5p (Previous ID: hsa-miR-194) |
| hsa-miR-98-5p |
| **GO:0032694 negative regulation of interleukin-11 production** |
| hsa-miR-124-3p |
| hsa-miR-23b-3p |
| **GO:0032695 negative regulation of interleukin-12 production** |
| hsa-miR-21-5p (Previous ID: hsa-miR-21) |
| **GO:0032699 negative regulation of interleukin-16 production** |
| hsa-miR-125a-5p |
| hsa-miR-145-3p (Previous ID: hsa-miR-145*) |
| **GO:0032700 negative regulation of interleukin-17 production** |
| hsa-let-7f-5p (Previous ID: hsa-let-7f) |
| hsa-miR-136-5p |
| hsa-miR-146b-5p |
| hsa-miR-181c-5p |
| hsa-miR-20a-5p |
| hsa-miR-26a-5p |
| **GO:0032701 negative regulation of interleukin-18 production** |
| hsa-miR-197-3p (Previous ID: hsa-miR-197) |
| hsa-miR-411-5p |
| **GO:0032703 negative regulation of interleukin-2 production** |
| hsa-miR-181c-5p |
| **GO:0032705 negative regulation of interleukin-21 production** |
| hsa-miR-192-5p |
| hsa-miR-21-5p (Previous ID: hsa-miR-21) |
| hsa-miR-221-3p |
| hsa-miR-222-3p (Previous ID: hsa-miR-222) |
| **GO:0032707 negative regulation of interleukin-23 production** |
| hsa-miR-155-5p |
| **GO:0032711 negative regulation of interleukin-27 production** |
| hsa-miR-935 |
| **GO:0032713 negative regulation of interleukin-4 production** |
| hsa-miR-320a-3p |
| **GO:0032715 negative regulation of interleukin-6 production** |
| hsa-let-7e-5p |
| hsa-miR-101-3p |
| hsa-miR-125a-5p |
| hsa-miR-132-3p (Previous ID: hsa-miR-132) |
| hsa-miR-146a-5p |
| hsa-miR-149-5p (Previous ID: hsa-miR-149) |
| hsa-miR-155-5p |
| hsa-miR-181a-5p |
| hsa-miR-195-5p (Previous ID: hsa-miR-195) |
| hsa-miR-19a-3p (Previous ID: hsa-miR-19a) |
| hsa-miR-19b-3p (Previous ID: hsa-miR-19b) |
| hsa-miR-204-5p |
| hsa-miR-206 |
| hsa-miR-26a-5p |
| hsa-miR-27b-3p |
| hsa-miR-365a-3p (Previous ID: hsa-miR-365) |
| hsa-miR-6869-5p |
| hsa-miR-708-5p (Previous ID: hsa-miR-708) |
| hsa-miR-766-3p (Previous ID: hsa-miR-766) |
| hsa-miR-98-5p |
| **GO:0032716 negative regulation of interleukin-7 production** |
| hsa-miR-181c-5p |
| **GO:0032717 negative regulation of interleukin-8 production** |
| hsa-miR-100-3p (Previous ID: hsa-miR-100*) |
| hsa-miR-106a-5p (Previous ID: hsa-miR-106a) |
| hsa-miR-106b-5p (Previous ID: hsa-miR-106b) |
| hsa-miR-129-5p |
| hsa-miR-132-3p (Previous ID: hsa-miR-132) |
| hsa-miR-146a-5p |
| hsa-miR-155-5p |
| hsa-miR-203a-3p |
| hsa-miR-204-5p |
| hsa-miR-302c-3p (Previous ID: hsa-miR-302c ) |
| hsa-miR-302d-3p (Previous ID: hsa-miR-302d) |
| hsa-miR-488-3p (Previous ID: hsa-miR-488) |
| hsa-miR-520c-3p |
| hsa-miR-766-3p (Previous ID: hsa-miR-766) |
| hsa-miR-93-5p |
| hsa-miR-98-5p |
| **GO:0032720 negative regulation of tumor necrosis factor production** |
| hsa-miR-125b-5p |
| hsa-miR-130a-3p (Previous ID: hsa-miR-130a) |
| hsa-miR-132-3p (Previous ID: hsa-miR-132) |
| hsa-miR-149-5p (Previous ID: hsa-miR-149) |
| hsa-miR-181a-5p |
| hsa-miR-195-5p (Previous ID: hsa-miR-195) |
| hsa-miR-488-3p (Previous ID: hsa-miR-488) |
| hsa-miR-708-5p (Previous ID: hsa-miR-708) |
| **GO:0032731 positive regulation of interleukin-1 beta production** |
| hsa-miR-206 |
| **GO:0032733 positive regulation of interleukin-10 production** |
| hsa-miR-145-3p (Previous ID: hsa-miR-145*) |
| **GO:0032735 positive regulation of interleukin-12 production** |
| hsa-miR-155-5p |
| **GO:0032755 positive regulation of interleukin-6 production** |
| hsa-miR-144-3p |
| hsa-miR-657 |
| hsa-miR-92a-3p |
| **GO:0032760 positive regulation of tumor necrosis factor production** |
| hsa-miR-206 |
| hsa-miR-657 |
| **GO:0032769 negative regulation of monooxygenase activity** |
| hsa-miR-27b-3p |
| hsa-miR-130b-3p (Previous ID: hsa-miR-130b) |
| **GO:0032869 cellular response to insulin stimulus** |
| hsa-miR-103a-3p |
| hsa-miR-107 |
| **GO:0032916 positive regulation of transforming growth factor beta3 production** |
| hsa-miR-149-5p (Previous ID: hsa-miR-149) |
| **GO:0032963 collagen metabolic process** |
| hsa-miR-21-5p (Previous ID: hsa-miR-21) |
| **GO:0032965 regulation of collagen biosynthetic process** |
| hsa-miR-145-5p |
|  |
|  |
|  |
| **GO:0032966 negative regulation of collagen biosynthetic process** |
| hsa-miR-218-5p |
| hsa-miR-29a-3p |
| hsa-miR-29b-3p |
| hsa-miR-92a-3p |
| **GO:0032967 positive regulation of collagen biosynthetic process** |
| hsa-miR-149-5p (Previous ID: hsa-miR-149) |
| **GO:0033137 negative regulation of peptidyl-serine phosphorylation** |
| hsa-miR-132-3p (Previous ID: hsa-miR-132) |
| hsa-miR-212-3p (Previous ID: hsa-miR-212) |
| **GO:0033209 tumor necrosis factor-mediated signaling pathway** |
| hsa-miR-34a-5p |
| **GO:0033689 negative regulation of osteoblast proliferation** |
| hsa-miR-138-5p |
| hsa-miR-675-5p |
| hsa-miR-9-5p |
| **GO:0034115 negative regulation of heterotypic cell-cell adhesion** |
| hsa-miR-221-3p |
| **GO:0034121 regulation of toll-like receptor signaling pathway** |
| hsa-miR-146a-5p |
| hsa-miR-146b-5p |
| **GO:0034122 negative regulation of toll-like receptor signaling pathway** |
| hsa-miR-17-5p (Previous ID: hsa-miR-17) |
| hsa-miR-19a-3p (Previous ID: hsa-miR-19a) |
| **GO:0034144 negative regulation of toll-like receptor 4 signaling pathway** |
| hsa-miR-146a-5p |
| hsa-miR-181b-5p |
| hsa-miR-708-5p (Previous ID: hsa-miR-708) |
| **GO:0034260 negative regulation of GTPase activity** |
| hsa-miR-21-5p (Previous ID: hsa-miR-21) |
| hsa-miR-223-3p (Previous ID: hsa-miR-223) |
| **GO:0034345 negative regulation of type III interferon production** |
| hsa-miR-29b-3p |
| **GO:0034392 negative regulation of smooth muscle cell apoptotic process** |
| hsa-miR-21-5p (Previous ID: hsa-miR-21) |
| hsa-miR-92a-3p |
| **GO:0034612 response to tumor necrosis factor** |
| hsa-miR-142-3p |
| **GO:0034614 cellular response to reactive oxygen species** |
| hsa-let-7b-5p |
| **GO:0034976 response to endoplasmic reticulum stress** |
| hsa-miR-200c-3p (Previous ID: hsa-miR-200c) |
| **GO:0035195 gene silencing by miRNA** |
| hsa-let-7a-5p |
| hsa-let-7b-5p |
| hsa-let-7c-5p |
| hsa-let-7e-5p |
| hsa-let-7f-5p (Previous ID: hsa-let-7f) |
| hsa-let-7g-5p |
| hsa-let-7i-5p |
| hsa-miR-101-3p |
| hsa-miR-103a-3p |
| hsa-miR-106a-5p (Previous ID: hsa-miR-106a) |
| hsa-miR-106b-5p (Previous ID: hsa-miR-106b) |
| hsa-miR-107 |
| hsa-miR-10a-5p (Previous ID: hsa-miR-10a) |
| hsa-miR-10b-5p (Previous ID: hsa-miR-10b) |
| hsa-miR-1207-5p |
| hsa-miR-124-3p |
| hsa-miR-125a-5p |
| hsa-miR-125b-1-3p |
| hsa-miR-125b-5p |
| hsa-miR-126-3p |
| hsa-miR-126-5p |
| hsa-miR-128-3p |
| hsa-miR-129-5p |
| hsa-miR-130a-3p (Previous ID: hsa-miR-130a) |
| hsa-miR-130b-3p (Previous ID: hsa-miR-130b) |
| hsa-miR-132-3p (Previous ID: hsa-miR-132) |
| hsa-miR-134-5p |
| hsa-miR-135b-5p |
| hsa-miR-136-5p |
| hsa-miR-138-5p |
| hsa-miR-141-3p (Previous ID: hsa-miR-141) |
| hsa-miR-142-3p |
| hsa-miR-142-5p |
| hsa-miR-143-3p |
| hsa-miR-144-3p |
| hsa-miR-145-3p (Previous ID: hsa-miR-145*) |
| hsa-miR-145-5p |
| hsa-miR-146a-5p |
| hsa-miR-146b-5p |
| hsa-miR-148a-3p |
| hsa-miR-148b-3p (Previous ID: hsa-miR-148b) |
| hsa-miR-148b-5p (Previous ID: hsa-miR-148b*) |
| hsa-miR-149-3p |
| hsa-miR-149-5p (Previous ID: hsa-miR-149) |
| hsa-miR-150-5p (Previous ID: hsa-miR-150) |
| hsa-miR-152-3p |
| hsa-miR-153-3p |
| hsa-miR-155-5p |
| hsa-miR-15a-3p (Previous ID: hsa-miR-15a*) |
| hsa-miR-15a-5p (Previous ID: hsa-miR-15a) |
| hsa-miR-15b-5p |
| hsa-miR-16-5p |
| hsa-miR-17-5p (Previous ID: hsa-miR-17) |
| hsa-miR-181a-5p |
| hsa-miR-181b-5p |
| hsa-miR-181c-5p |
| hsa-miR-182-5p (Previous ID: hsa-miR-182) |
| hsa-miR-183-5p (Previous ID: hsa-miR-183) |
| hsa-miR-185-3p |
| hsa-miR-185-5p (Previous ID: hsa-miR-185) |
| hsa-miR-18a-5p (Previous ID: hsa-miR-18a) |
| hsa-miR-18b-5p (Previous ID: hsa-miR-18b) |
| hsa-miR-1908-5p |
| hsa-miR-191-5p (Previous ID: hsa-miR-191) |
| hsa-miR-192-5p |
| hsa-miR-193a-3p |
| hsa-miR-193b-3p (Previous ID: hsa-miR-193b) |
| hsa-miR-194-5p (Previous ID: hsa-miR-194) |
| hsa-miR-195-5p (Previous ID: hsa-miR-195) |
| hsa-miR-196a-5p |
| hsa-miR-197-3p (Previous ID: hsa-miR-197) |
| hsa-miR-198 |
| hsa-miR-199a-5p |
| hsa-miR-199b-3p / hsa-miR-199a-3p |
| hsa-miR-199b-5p (Previous ID: hsa-miR-199b) |
| hsa-miR-19a-3p (Previous ID: hsa-miR-19a) |
| hsa-miR-19b-3p (Previous ID: hsa-miR-19b) |
| hsa-miR-200a-3p (Previous ID: hsa-miR-200a) |
| hsa-miR-200b-3p (Previous ID: hsa-miR-200b) |
| hsa-miR-200c-3p (Previous ID: hsa-miR-200c) |
| hsa-miR-203a-3p |
| hsa-miR-204-5p |
| hsa-miR-205-5p (Previous ID: hsa-miR-205) |
| hsa-miR-206 |
| hsa-miR-20a-5p |
| hsa-miR-20b-5p (Previous ID: hsa-miR-20b) |
| hsa-miR-210-5p |
| hsa-miR-212-3p (Previous ID: hsa-miR-212) |
| hsa-miR-21-3p |
| hsa-miR-214-3p |
| hsa-miR-21-5p (Previous ID: hsa-miR-21) |
| hsa-miR-218-5p |
| hsa-miR-219a-5p (Previous ID: hsa-miR-219) |
| hsa-miR-221-3p |
| hsa-miR-222-3p (Previous ID: hsa-miR-222) |
| hsa-miR-223-3p (Previous ID: hsa-miR-223) |
| hsa-miR-22-3p |
| hsa-miR-224-5p (Previous ID: hsa-miR-224) |
| hsa-miR-23a-3p |
| hsa-miR-23b-3p |
| hsa-miR-24-3p |
| hsa-miR-25-3p |
| hsa-miR-25-5p (Previous ID: hsa-miR-25*) |
| hsa-miR-26a-5p |
| hsa-miR-26b-5p (Previous ID: hsa-miR-26b) |
| hsa-miR-27a-3p |
| hsa-miR-27a-5p |
| hsa-miR-27b-3p |
| hsa-miR-27b-5p (Previous ID: hsa-miR-27b*) |
| hsa-miR-28-5p (Previous ID: hsa-miR-28) |
| hsa-miR-296-5p |
| hsa-miR-29a-3p |
| hsa-miR-29b-3p |
| hsa-miR-29c-3p |
| hsa-miR-301a-3p (Previous ID: hsa-miR-301) |
| hsa-miR-301b-3p (Previous ID: hsa-miR-301b) |
| hsa-miR-302a-3p (Previous ID: hsa-miR-302a) |
| hsa-miR-302c-3p (Previous ID: hsa-miR-302c ) |
| hsa-miR-302d-3p (Previous ID: hsa-miR-302d) |
| hsa-miR-30a-3p |
| hsa-miR-30a-5p |
| hsa-miR-30b-5p (Previous ID: hsa-miR-30b) |
| hsa-miR-30c-5p |
| hsa-miR-30e-5p |
| hsa-miR-31-5p |
| hsa-miR-320a-3p |
| hsa-miR-323a-3p (Previous ID: hsa-miR-323-3p) |
| hsa-miR-330-3p (Previous ID: hsa-miR-330) |
| hsa-miR-338-3p |
| hsa-miR-33a-5p (Previous ID: hsa-miR-33a) |
| hsa-miR-33b-5p (Previous ID: hsa-miR-33b) |
| hsa-miR-342-5p |
| hsa-miR-34a-5p |
| hsa-miR-34c-5p (Previous ID: hsa-miR-34c) |
| hsa-miR-361-3p |
| hsa-miR-361-5p (Previous ID: hsa-miR-361) |
| hsa-miR-362-3p |
| hsa-miR-365a-3p (Previous ID: hsa-miR-365) |
| hsa-miR-374a-5p (Previous ID: hsa-miR-374) |
| hsa-miR-378a-3p |
| hsa-miR-384 |
| hsa-miR-409-3p |
| hsa-miR-410-3p |
| hsa-miR-411-5p |
| hsa-miR-423-3p |
| hsa-miR-424-5p (Previous ID: hsa-miR-424) |
| hsa-miR-425-5p |
| hsa-miR-429 |
| hsa-miR-449a (Previous ID: hsa-miR-449) |
| hsa-miR-455-3p |
| hsa-miR-455-5p (Previous ID: hsa-miR-455) |
| hsa-miR-483-3p |
| hsa-miR-485-5p |
| hsa-miR-488-3p (Previous ID: hsa-miR-488) |
| hsa-miR-492 |
| hsa-miR-497-5p (Previous ID: hsa-miR-497) |
| hsa-miR-503-5p (Previous ID: hsa-miR-503) |
| hsa-miR-505-3p (Previous ID: hsa-miR-505) |
| hsa-miR-518b |
| hsa-miR-519b-3p |
| hsa-miR-520a-3p (Previous ID: hsa-miR-520a) |
| hsa-miR-520c-3p |
| hsa-miR-543 |
| hsa-miR-548c-3p (Previous ID: hsa-miR-548c) |
| hsa-miR-548d-5p |
| hsa-miR-572 |
| hsa-miR-590-3p |
| hsa-miR-590-5p |
| hsa-miR-595 |
| hsa-miR-638 |
| hsa-miR-639 |
| hsa-miR-654-3p |
| hsa-miR-657 |
| hsa-miR-661 |
| hsa-miR-663a |
| hsa-miR-665 |
| hsa-miR-675-5p |
| hsa-miR-6869-5p |
| hsa-miR-708-5p (Previous ID: hsa-miR-708) |
| hsa-miR-758-3p (Previous ID: hsa-miR-758) |
| hsa-miR-766-3p (Previous ID: hsa-miR-766) |
| hsa-miR-892b |
| hsa-miR-92a-3p |
| hsa-miR-935 |
| hsa-miR-93-5p |
| hsa-miR-9-3p (Previous ID: hsa-miR-9*) |
| hsa-miR-9-5p |
| hsa-miR-98-5p |
| hsa-miR-99a-5p |
| **GO:0035278 miRNA mediated inhibition of translation** |
| hsa-let-7a-5p |
| hsa-let-7e-5p |
| hsa-let-7i-5p |
| hsa-miR-100-3p (Previous ID: hsa-miR-100*) |
| hsa-miR-101-3p |
| hsa-miR-103a-3p |
| hsa-miR-106a-5p (Previous ID: hsa-miR-106a) |
| hsa-miR-106b-5p (Previous ID: hsa-miR-106b) |
| hsa-miR-107 |
| hsa-miR-124-3p |
| hsa-miR-125b-5p |
| hsa-miR-132-3p (Previous ID: hsa-miR-132) |
| hsa-miR-134-5p |
| hsa-miR-135b-5p |
| hsa-miR-138-5p |
| hsa-miR-145-5p |
| hsa-miR-146a-5p |
| hsa-miR-148a-3p |
| hsa-miR-155-5p |
| hsa-miR-15a-5p (Previous ID: hsa-miR-15a) |
| hsa-miR-15b-5p |
| hsa-miR-16-5p |
| hsa-miR-17-5p (Previous ID: hsa-miR-17) |
| hsa-miR-181a-5p |
| hsa-miR-181b-5p |
| hsa-miR-181c-5p |
| hsa-miR-182-5p (Previous ID: hsa-miR-182) |
| hsa-miR-19b-3p (Previous ID: hsa-miR-19b) |
| hsa-miR-200b-3p (Previous ID: hsa-miR-200b) |
| hsa-miR-200c-3p (Previous ID: hsa-miR-200c) |
| hsa-miR-204-5p |
| hsa-miR-205-5p (Previous ID: hsa-miR-205) |
| hsa-miR-20a-5p |
| hsa-miR-212-3p (Previous ID: hsa-miR-212) |
| hsa-miR-21-3p |
| hsa-miR-21-5p (Previous ID: hsa-miR-21) |
| hsa-miR-218-5p |
| hsa-miR-221-3p |
| hsa-miR-222-3p (Previous ID: hsa-miR-222) |
| hsa-miR-27a-3p |
| hsa-miR-27a-5p |
| hsa-miR-27b-3p |
| hsa-miR-28-5p (Previous ID: hsa-miR-28) |
| hsa-miR-299-5p |
| hsa-miR-29a-3p |
| hsa-miR-29b-3p |
| hsa-miR-31-5p |
| hsa-miR-345-5p (Previous ID: hsa-miR-345) |
| hsa-miR-365a-3p (Previous ID: hsa-miR-365) |
| hsa-miR-483-5p |
| hsa-miR-503-5p (Previous ID: hsa-miR-503) |
| hsa-miR-518b |
| hsa-miR-520c-3p |
| hsa-miR-590-3p |
| hsa-miR-590-5p |
| hsa-miR-659-3p (Previous ID: hsa-miR-659) |
| hsa-miR-92a-3p |
| hsa-miR-939-5p (Previous ID: hsa-miR-939) |
| hsa-miR-9-5p |
| hsa-miR-98-5p |
| **GO:0035308 negative regulation of protein dephosphorylation** |
| hsa-miR-124-3p |
| **GO:0035886 vascular associated smooth muscle cell differentiation** |
| hsa-miR-145-5p |
| **GO:0035924 cellular response to vascular endothelial growth factor stimulus** |
| hsa-miR-196a-5p |
| hsa-miR-20a-5p |
| hsa-miR-23a-3p |
| hsa-miR-23b-3p |
| hsa-miR-27a-3p |
| hsa-miR-27b-3p |
| **GO:0036166 phenotypic switching** |
| hsa-miR-21-5p (Previous ID: hsa-miR-21) |
| **GO:0036446 myofibroblast differentiation** |
| hsa-miR-145-5p |
| **GO:0038027 apolipoprotein A-I-mediated signaling pathway** |
| hsa-miR-33a-5p (Previous ID: hsa-miR-33a) |
| **GO:0038061 NIK/NF-kappaB signaling** |
| hsa-miR-125b-5p |
| hsa-miR-130a-3p (Previous ID: hsa-miR-130a) |
| hsa-miR-146a-5p |
| hsa-miR-27a-3p |
| hsa-miR-27b-3p |
| **GO:0038084 vascular endothelial growth factor signaling pathway** |
| hsa-miR-26a-5p |
| **GO:0038166 angiotensin-activated signaling pathway** |
| hsa-miR-143-3p |
| hsa-miR-145-5p |
| **GO:0039531 regulation of viral-induced cytoplasmic pattern recognition receptor signaling pathway** |
| hsa-miR-708-5p (Previous ID: hsa-miR-708) |
| **GO:0040037 negative regulation of fibroblast growth factor receptor signaling pathway** |
| hsa-miR-149-3p |
| hsa-miR-149-5p (Previous ID: hsa-miR-149) |
| hsa-miR-16-5p |
| hsa-miR-424-5p (Previous ID: hsa-miR-424) |
| hsa-miR-503-5p (Previous ID: hsa-miR-503) |
| **GO:0042058 regulation of epidermal growth factor receptor signaling pathway** |
| hsa-miR-21-5p (Previous ID: hsa-miR-21) |
| **GO:0042104 positive regulation of activated T cell proliferation** |
| hsa-miR-155-5p |
| hsa-miR-21-5p (Previous ID: hsa-miR-21) |
| hsa-miR-30b-5p (Previous ID: hsa-miR-30b) |
| **GO:0042113 B cell activation** |
| hsa-miR-19a-3p (Previous ID: hsa-miR-19a) |
| **GO:0042127 regulation of cell population proliferation** |
| hsa-miR-155-5p |
| **GO:0042531 positive regulation of tyrosine phosphorylation of STAT protein** |
| hsa-miR-19a-3p (Previous ID: hsa-miR-19a) |
| hsa-miR-19b-3p (Previous ID: hsa-miR-19b) |
| hsa-miR-221-3p |
| **GO:0042532 negative regulation of tyrosine phosphorylation of STAT protein** |
| hsa-let-7e-5p |
| hsa-miR-125a-5p |
| hsa-miR-146a-5p |
| hsa-miR-98-5p |
| **GO:0042534 regulation of tumor necrosis factor biosynthetic process** |
| hsa-miR-361-5p (Previous ID: hsa-miR-361) |
| **GO:0042536 negative regulation of tumor necrosis factor biosynthetic process** |
| hsa-miR-98-5p |
| **GO:0042593 glucose homeostasis** |
| hsa-miR-33a-5p (Previous ID: hsa-miR-33a) |
| **GO:0042632 cholesterol homeostasis** |
| hsa-miR-128-3p |
| hsa-miR-144-3p |
| hsa-miR-148a-3p |
| hsa-miR-155-5p |
| hsa-miR-182-5p (Previous ID: hsa-miR-182) |
| hsa-miR-185-5p (Previous ID: hsa-miR-185) |
| hsa-miR-19b-3p (Previous ID: hsa-miR-19b) |
| hsa-miR-27b-3p |
| hsa-miR-302a-3p (Previous ID: hsa-miR-302a) |
| hsa-miR-30c-5p |
| hsa-miR-33b-5p (Previous ID: hsa-miR-33b) |
| hsa-miR-34a-5p |
| hsa-miR-590-3p |
| **GO:0042742 defense response to bacterium** |
| hsa-miR-155-5p |
| hsa-miR-223-3p (Previous ID: hsa-miR-223) |
| **GO:0042832 defense response to protozoan** |
| hsa-let-7i-5p |
| **GO:0042953 lipoprotein transport** |
| hsa-miR-130b-3p (Previous ID: hsa-miR-130b) |
| hsa-miR-301b-3p (Previous ID: hsa-miR-301b) |
| **GO:0042981 regulation of apoptotic process** |
| hsa-miR-155-5p |
| **GO:0042985 negative regulation of amyloid precursor protein biosynthetic process** |
| hsa-miR-101-3p |
| hsa-miR-106a-5p (Previous ID: hsa-miR-106a) |
| hsa-miR-17-5p (Previous ID: hsa-miR-17) |
| hsa-miR-29a-3p |
| hsa-miR-323a-3p (Previous ID: hsa-miR-323-3p) |
| hsa-miR-455-3p |
| hsa-miR-520c-3p |
| **GO:0043031 negative regulation of macrophage activation** |
| hsa-miR-124-3p |
| hsa-miR-130a-3p (Previous ID: hsa-miR-130a) |
| **GO:0043032 positive regulation of macrophage activation** |
| hsa-miR-145-3p (Previous ID: hsa-miR-145*) |
| hsa-miR-155-5p |
| **GO:0043065 positive regulation of apoptotic process** |
| hsa-miR-146a-5p |
| hsa-miR-15a-5p (Previous ID: hsa-miR-15a) |
| hsa-miR-15b-5p |
| hsa-miR-16-5p |
| hsa-miR-204-5p |
| hsa-miR-195-5p (Previous ID: hsa-miR-195) |
| hsa-miR-200b-3p (Previous ID: hsa-miR-200b) |
| hsa-miR-21-3p |
| hsa-miR-221-3p |
| hsa-miR-29a-3p |
| hsa-miR-29b-3p |
| hsa-miR-29c-3p |
| hsa-miR-518b |
| hsa-miR-92a-3p |
| **GO:0043066 negative regulation of apoptotic process** |
| hsa-let-7f-5p (Previous ID: hsa-let-7f) |
| hsa-miR-19a-3p (Previous ID: hsa-miR-19a) |
| hsa-miR-200b-3p (Previous ID: hsa-miR-200b) |
| hsa-miR-21-5p (Previous ID: hsa-miR-21) |
| hsa-miR-221-3p |
| hsa-miR-222-3p (Previous ID: hsa-miR-222) |
| hsa-miR-361-3p |
| **GO:0043116 negative regulation of vascular permeability** |
| hsa-miR-23a-3p |
| **GO:0043117 positive regulation of vascular permeability** |
| hsa-miR-23b-3p |
|  |
|  |
|  |
| **GO:0043124 negative regulation of I-kappaB kinase/NF-kappaB signaling** |
| hsa-miR-15a-5p (Previous ID: hsa-miR-15a) |
| hsa-miR-15b-5p |
| hsa-miR-16-5p |
| hsa-miR-195-5p (Previous ID: hsa-miR-195) |
| hsa-miR-497-5p (Previous ID: hsa-miR-497) |
| **GO:0043154 negative regulation of cysteine-type endopeptidase activity involved in apoptotic process** |
| hsa-let-7b-5p |
| hsa-miR-17-5p (Previous ID: hsa-miR-17) |
| **GO:0043280 positive regulation of cysteine-type endopeptidase activity involved in apoptotic process** |
| hsa-miR-27b-5p (Previous ID: hsa-miR-27b*) |
| **GO:0043407 negative regulation of MAP kinase activity** |
| hsa-miR-218-5p |
| **GO:0043409 negative regulation of MAPK cascade** |
| hsa-miR-21-5p (Previous ID: hsa-miR-21) |
| hsa-miR-26a-5p |
| hsa-miR-29b-3p |
| **GO:0043410 positive regulation of MAPK cascade** |
| hsa-miR-126-3p |
| **GO:0043433 negative regulation of DNA-binding transcription factor activity** |
| hsa-let-7e-5p |
| hsa-miR-101-3p |
| hsa-miR-125a-5p |
| hsa-miR-144-3p |
| hsa-miR-26a-5p |
| hsa-miR-29b-3p |
| hsa-miR-892b |
| **GO:0043507 positive regulation of JUN kinase activity** |
| hsa-miR-92a-3p |
| **GO:0043508 negative regulation of JUN kinase activity** |
| hsa-miR-92a-3p |
| **GO:0043524 negative regulation of neuron apoptotic process** |
| hsa-miR-124-3p |
| hsa-miR-15b-5p |
| hsa-miR-181c-5p |
| **GO:0043525 positive regulation of neuron apoptotic process** |
| hsa-miR-125b-1-3p |
| hsa-miR-200a-3p (Previous ID: hsa-miR-200a) |
| hsa-miR-98-5p |
| **GO:0043536 positive regulation of blood vessel endothelial cell migration** |
| hsa-miR-135b-5p |
| hsa-miR-143-3p |
| hsa-miR-200a-3p (Previous ID: hsa-miR-200a) |
| hsa-miR-30a-3p |
| hsa-miR-221-3p |
| hsa-miR-31-5p |
| hsa-miR-342-5p |
| hsa-miR-939-5p (Previous ID: hsa-miR-939) |
| **GO:0043537 negative regulation of blood vessel endothelial cell migration** |
| hsa-miR-101-3p |
| hsa-miR-10a-3p (Previous ID: hsa-miR-10a*) |
| hsa-miR-129-5p |
| hsa-miR-152-3p |
| hsa-miR-155-5p |
| hsa-miR-15b-5p |
| hsa-miR-193a-5p |
| hsa-miR-200b-3p (Previous ID: hsa-miR-200b) |
| hsa-miR-204-5p |
| hsa-miR-212-3p (Previous ID: hsa-miR-212) |
| hsa-miR-24-3p |
| hsa-miR-30b-5p (Previous ID: hsa-miR-30b) |
| hsa-miR-424-5p (Previous ID: hsa-miR-424) |
| hsa-miR-492 |
| hsa-miR-503-5p (Previous ID: hsa-miR-503) |
| hsa-miR-505-3p (Previous ID: hsa-miR-505) |
| hsa-miR-92a-3p |
| **GO:0043553 negative regulation of phosphatidylinositol 3-kinase activity** |
| hsa-miR-138-5p |
| **GO:0044030 regulation of DNA methylation** |
| hsa-miR-29a-3p |
| hsa-miR-29b-3p |
| hsa-miR-29c-3p |
| **GO:0044344 cellular response to fibroblast growth factor stimulus** |
| hsa-miR-149-3p |
| hsa-miR-149-5p (Previous ID: hsa-miR-149) |
| hsa-miR-339-5p |
| **GO:0044663 establishment or maintenance of cell type involved in phenotypic switching** |
| hsa-miR-143-3p |
| hsa-miR-145-5p |
| **GO:0044828 negative regulation by host of viral genome replication** |
| hsa-miR-155-5p |
| hsa-miR-221-3p |
| hsa-miR-222-3p (Previous ID: hsa-miR-222) |
| **GO:0044854 plasma membrane raft assembly** |
| hsa-miR-138-5p |
| **GO:0045019 negative regulation of nitric oxide biosynthetic process** |
| hsa-miR-132-3p (Previous ID: hsa-miR-132) |
| hsa-miR-212-3p (Previous ID: hsa-miR-212) |
| hsa-miR-199a-5p |
| hsa-miR-92a-3p |
| **GO:0045071 negative regulation of viral genome replication** |
| hsa-miR-141-3p (Previous ID: hsa-miR-141) |
| **GO:0045429 positive regulation of nitric oxide biosynthetic process** |
| hsa-miR-181a-2-3p (Previous ID: hsa-miR-181a-2*) |
| hsa-miR-99b-3p (Previous ID: hsa-miR-99b*) |
| **GO:0045541 negative regulation of cholesterol biosynthetic process** |
| hsa-miR-30c-5p |
| **GO:0045542 positive regulation of cholesterol biosynthetic process** |
| hsa-miR-182-5p (Previous ID: hsa-miR-182) |
| **GO:0045590 negative regulation of regulatory T cell differentiation** |
| hsa-miR-155-5p |
| hsa-miR-21-5p (Previous ID: hsa-miR-21) |
| hsa-miR-30b-5p (Previous ID: hsa-miR-30b) |
| **GO:0045598 regulation of fat cell differentiation** |
| hsa-miR-181a-5p |
| **GO:0045599 negative regulation of fat cell differentiation** |
| hsa-miR-103a-3p |
| hsa-miR-107 |
| hsa-miR-27b-3p |
| hsa-miR-483-3p |
| hsa-miR-548d-5p |
| **GO:0045600 positive regulation of fat cell differentiation** |
| hsa-miR-21-5p (Previous ID: hsa-miR-21) |
| hsa-miR-29b-3p |
| **GO:0045603 positive regulation of endothelial cell differentiation** |
| hsa-miR-150-5p (Previous ID: hsa-miR-150) |
| hsa-miR-181a-2-3p (Previous ID: hsa-miR-181a-2*) |
| hsa-miR-199b-5p (Previous ID: hsa-miR-199b) |
| hsa-miR-200c-3p (Previous ID: hsa-miR-200c) |
| hsa-miR-21-5p (Previous ID: hsa-miR-21) |
| hsa-miR-99b-3p (Previous ID: hsa-miR-99b*) |
| **GO:0045618 positive regulation of keratinocyte differentiation** |
| hsa-miR-125b-5p |
| **GO:0045648 positive regulation of erythrocyte differentiation** |
| hsa-miR-221-3p |
| hsa-miR-222-3p (Previous ID: hsa-miR-222) |
| **GO:0045649 regulation of macrophage differentiation** |
| hsa-miR-223-3p (Previous ID: hsa-miR-223) |
| **GO:0045651 positive regulation of macrophage differentiation** |
| hsa-miR-145-3p (Previous ID: hsa-miR-145*) |
| **GO:0045656 negative regulation of monocyte differentiation** |
| hsa-miR-125b-5p |
| **GO:0045666 positive regulation of neuron differentiation** |
| hsa-miR-124-3p |
| hsa-miR-146a-5p |
| **GO:0045668 negative regulation of osteoblast differentiation** |
| hsa-miR-138-5p |
| hsa-miR-665 |
| hsa-miR-675-5p |
| hsa-miR-9-5p |
| **GO:0045669 positive regulation of osteoblast differentiation** |
| hsa-miR-200c-3p (Previous ID: hsa-miR-200c) |
| hsa-miR-20b-5p (Previous ID: hsa-miR-20b) |
| hsa-miR-21-5p (Previous ID: hsa-miR-21) |
| hsa-miR-548d-5p |
| **GO:0045717 negative regulation of fatty acid biosynthetic process** |
| hsa-miR-30c-5p |
| hsa-miR-33a-5p (Previous ID: hsa-miR-33a) |
| **GO:0045722 positive regulation of gluconeogenesis** |
| hsa-miR-103a-3p |
| hsa-miR-107 |
| **GO:0045723 positive regulation of fatty acid biosynthetic process** |
| hsa-miR-182-5p (Previous ID: hsa-miR-182) |
| **GO:0045727 positive regulation of translation** |
| hsa-miR-15b-5p |
| hsa-miR-16-5p |
| **GO:0045736 negative regulation of cyclin-dependent protein serine/threonine kinase activity** |
| hsa-miR-103a-3p |
| hsa-miR-15a-5p (Previous ID: hsa-miR-15a) |
| **GO:0045743 positive regulation of fibroblast growth factor receptor signaling pathway** |
| hsa-miR-146a-5p |
| **GO:0045747 positive regulation of Notch signaling pathway** |
| hsa-miR-126-5p |
| hsa-miR-212-3p (Previous ID: hsa-miR-212) |
| **GO:0045765 regulation of angiogenesis** |
| hsa-miR-199a-5p |
| hsa-miR-199b-5p (Previous ID: hsa-miR-199b) |
| **GO:0045766 positive regulation of angiogenesis** |
| hsa-let-7b-5p |
| hsa-let-7g-5p |
| hsa-miR-126-3p |
| hsa-miR-130a-3p (Previous ID: hsa-miR-130a) |
| hsa-miR-132-3p (Previous ID: hsa-miR-132) |
| hsa-miR-143-3p |
| hsa-miR-1908-5p |
| hsa-miR-199a-5p |
| hsa-miR-199b-3p / hsa-miR-199a-3p |
| hsa-miR-199b-5p (Previous ID: hsa-miR-199b) |
| hsa-miR-20a-5p |
| hsa-miR-21-5p (Previous ID: hsa-miR-21) |
| hsa-miR-27b-3p |
| hsa-miR-29a-3p |
| hsa-miR-30a-3p |
| hsa-miR-31-5p |
| hsa-miR-99b-3p (Previous ID: hsa-miR-99b*) |
| **GO:0045777 positive regulation of blood pressure** |
| hsa-miR-17-5p (Previous ID: hsa-miR-17) |
| **GO:0045787 positive regulation of cell cycle** |
| hsa-miR-590-5p |
| hsa-miR-26b-5p (Previous ID: hsa-miR-26b) |
| **GO:0045824 negative regulation of innate immune response** |
| hsa-miR-155-5p |
| hsa-miR-181b-5p |
| hsa-miR-21-5p (Previous ID: hsa-miR-21) |
| **GO:0045893 positive regulation of transcription, DNA-templated** |
| hsa-miR-27a-3p |
| **GO:0045899 positive regulation of RNA polymerase II transcription preinitiation complex assembly** |
| hsa-let-7i-5p |
| **GO:0045930 negative regulation of mitotic cell cycle** |
| hsa-miR-134-5p |
| hsa-miR-15b-5p |
| hsa-miR-16-5p |
| hsa-miR-195-5p (Previous ID: hsa-miR-195) |
| **GO:0045944 positive regulation of transcription by RNA polymerase II** |
| hsa-let-7c-5p |
| hsa-let-7i-5p |
| hsa-miR-138-5p |
| **GO:0046007 negative regulation of activated T cell proliferation** |
| hsa-miR-181c-5p |
| **GO:0046325 negative regulation of glucose import** |
| hsa-miR-103a-3p |
| hsa-miR-107 |
| hsa-miR-143-3p |
| **GO:0046326 positive regulation of glucose import** |
| hsa-miR-223-3p (Previous ID: hsa-miR-223) |
| **GO:0046329 negative regulation of JNK cascade** |
| hsa-miR-92a-3p |
| **GO:0046426 negative regulation of receptor signaling pathway via JAK-STAT** |
| hsa-miR-155-5p |
| hsa-miR-9-5p |
| **GO:0046427 positive regulation of receptor signaling pathway via JAK-STAT** |
| hsa-miR-221-3p |
| **GO:0046627 negative regulation of insulin receptor signaling pathway** |
| hsa-miR-103a-3p |
| hsa-miR-107 |
| **GO:0046716 muscle cell cellular homeostasis** |
| hsa-miR-155-5p |
| **GO:0048008 platelet-derived growth factor receptor signaling pathway** |
| hsa-miR-221-3p |
| hsa-miR-638 |
| **GO:0048026 positive regulation of mRNA splicing, via spliceosome** |
| hsa-miR-124-3p |
| **GO:0048146 positive regulation of fibroblast proliferation** |
| hsa-miR-155-5p |
| hsa-miR-17-3p (Previous ID: hsa-miR-17*) |
| **GO:0048147 negative regulation of fibroblast proliferation** |
| hsa-miR-124-3p |
|  |
|  |
| **GO:0048261 negative regulation of receptor-mediated endocytosis** |
| hsa-miR-205-5p (Previous ID: hsa-miR-205) |
| **GO:0048333 mesodermal cell differentiation** |
| hsa-miR-145-5p |
| **GO:0048662 negative regulation of smooth muscle cell proliferation** |
| hsa-miR-143-3p |
| hsa-miR-145-5p |
| **GO:0048678 response to axon injury** |
| hsa-miR-34a-5p |
| **GO:0048680 positive regulation of axon regeneration** |
| hsa-miR-221-3p |
| hsa-miR-222-3p (Previous ID: hsa-miR-222) |
| hsa-miR-431-5p (Previous ID: hsa-miR-431) |
| **GO:0050680 negative regulation of epithelial cell proliferation** |
| hsa-miR-29b-3p |
| **GO:0050687 negative regulation of defense response to virus** |
| hsa-miR-26b-5p (Previous ID: hsa-miR-26b) |
| **GO:0050709 negative regulation of protein secretion** |
| hsa-miR-29b-3p |
| hsa-miR-30c-5p |
| hsa-miR-93-5p |
| **GO:0050714 positive regulation of protein secretion** |
| hsa-miR-199b-5p (Previous ID: hsa-miR-199b) |
| **GO:0050715 positive regulation of cytokine secretion** |
| hsa-miR-21-5p (Previous ID: hsa-miR-21) |
| **GO:0050718 positive regulation of interleukin-1 beta production** |
| hsa-miR-144-3p |
| **GO:0050722 regulation of interleukin-1 beta production** |
| hsa-miR-361-5p (Previous ID: hsa-miR-361) |
| **GO:0050723 negative regulation of interleukin-1 alpha production** |
| hsa-miR-181a-5p |
| hsa-miR-181c-5p |
| **GO:0050724 negative regulation of interleukin-1 beta production** |
| hsa-miR-204-5p |
| **GO:0050727 regulation of inflammatory response** |
| hsa-miR-361-5p (Previous ID: hsa-miR-361) |
| hsa-miR-657 |
| **GO:0050728 negative regulation of inflammatory response** |
| hsa-let-7g-5p |
| hsa-miR-124-3p |
| hsa-miR-126-3p |
| hsa-miR-138-5p |
| hsa-miR-141-3p (Previous ID: hsa-miR-141) |
| hsa-miR-142-3p |
| hsa-miR-145-3p (Previous ID: hsa-miR-145*) |
| hsa-miR-146a-5p |
| hsa-miR-149-3p |
| hsa-miR-149-5p (Previous ID: hsa-miR-149) |
| hsa-miR-155-5p |
| hsa-miR-15a-5p (Previous ID: hsa-miR-15a) |
| hsa-miR-16-5p |
| hsa-miR-181a-5p |
| hsa-miR-181b-5p |
| hsa-miR-181c-5p |
| hsa-miR-19a-3p (Previous ID: hsa-miR-19a) |
| hsa-miR-204-5p |
| hsa-miR-20a-5p |
| hsa-miR-221-3p |
| hsa-miR-222-3p (Previous ID: hsa-miR-222) |
| hsa-miR-223-3p (Previous ID: hsa-miR-223) |
| hsa-miR-223-5p (Previous ID: hsa-miR-223*) |
| hsa-miR-31-5p |
| hsa-miR-488-3p (Previous ID: hsa-miR-488) |
| hsa-miR-590-3p |
| hsa-miR-766-3p (Previous ID: hsa-miR-766) |
| hsa-miR-92a-3p |
| **GO:0050729 positive regulation of inflammatory response** |
| hsa-miR-126-3p |
| hsa-miR-144-3p |
| hsa-miR-155-5p |
| hsa-miR-181b-5p |
| hsa-miR-21-5p (Previous ID: hsa-miR-21) |
| hsa-miR-22-3p |
| hsa-miR-92a-3p |
| **GO:0050732 negative regulation of peptidyl-tyrosine phosphorylation** |
| hsa-miR-106b-5p **(Previous ID: hsa-miR-**106b) |
| **GO:0050765 negative regulation of phagocytosis** |
| hsa-miR-181b-5p |
| **GO:0050766 positive regulation of phagocytosis** |
| hsa-miR-17-5p (Previous ID: hsa-miR-17) |
| hsa-miR-183-5p (Previous ID: hsa-miR-183) |
| hsa-miR-20a-5p |
| **GO:0050778 positive regulation of immune response** |
| hsa-miR-136-5p |
| **GO:0050819 negative regulation of coagulation** |
| hsa-miR-19b-3p (Previous ID: hsa-miR-19b) |
| **GO:0050821 protein stabilization** |
| hsa-miR-101-3p |
| hsa-miR-34a-5p |
| **GO:0050859 negative regulation of B cell receptor signaling pathway** |
| hsa-miR-34a-5p |
| **GO:0050861 positive regulation of B cell receptor signaling pathway** |
| hsa-miR-18a-5p (Previous ID: hsa-miR-18a) |
| hsa-miR-19a-3p (Previous ID: hsa-miR-19a) |
| **GO:0050868 negative regulation of T cell activation** |
| hsa-miR-181c-5p |
| hsa-miR-27a-3p |
| **GO:0050870 positive regulation of T cell activation** |
| hsa-miR-155-5p |
| **GO:0051001 negative regulation of nitric-oxide synthase activity** |
| hsa-miR-132-3p (Previous ID: hsa-miR-132) |
| hsa-miR-138-5p |
| hsa-miR-212-3p (Previous ID: hsa-miR-212) |
| hsa-miR-26a-5p |
| **GO:0051005 negative regulation of lipoprotein lipase activity** |
| hsa-miR-590-3p |
| **GO:0051006 positive regulation of lipoprotein lipase activity** |
| hsa-miR-182-5p (Previous ID: hsa-miR-182) |
| **GO:0051091 positive regulation of DNA-binding transcription factor activity** |
| hsa-miR-19b-3p (Previous ID: hsa-miR-19b) |
| **GO:0051151 negative regulation of smooth muscle cell differentiation** |
| hsa-miR-199b-5p (Previous ID: hsa-miR-199b) |
| **GO:0051152 positive regulation of smooth muscle cell differentiation** |
| hsa-miR-22-3p |
| hsa-miR-34a-5p |
|  |
|  |
|  |
| **GO:0051497 negative regulation of stress fiber assembly** |
| hsa-miR-138-5p |
| hsa-miR-149-5p (Previous ID: hsa-miR-149) |
| hsa-miR-20a-5p |
| hsa-miR-21-5p (Previous ID: hsa-miR-21) |
| **GO:0051607 defense response to virus** |
| hsa-miR-155-5p |
| hsa-miR-26b-5p (Previous ID: hsa-miR-26b) |
| **GO:0051771 negative regulation of nitric-oxide synthase biosynthetic process** |
| hsa-miR-132-3p (Previous ID: hsa-miR-132) |
| **GO:0051896 regulation of protein kinase B signaling** |
| hsa-miR-21-5p (Previous ID: hsa-miR-21) |
| **GO:0051897 positive regulation of protein kinase B signaling** |
| hsa-miR-126-3p |
| hsa-miR-132-3p (Previous ID: hsa-miR-132) |
| hsa-miR-138-5p |
| hsa-miR-143-3p |
| hsa-miR-199b-3p / hsa-miR-199a-3p |
| hsa-miR-21-5p (Previous ID: hsa-miR-21) |
| hsa-miR-221-3p |
| hsa-miR-222-3p (Previous ID: hsa-miR-222) |
| hsa-miR-29a-3p |
| **GO:0051898 negative regulation of protein kinase B signaling** |
| hsa-let-7f-5p (Previous ID: hsa-let-7f) |
| hsa-miR-145-5p |
| hsa-miR-146a-5p |
| hsa-miR-200c-3p (Previous ID: hsa-miR-200c) |
| hsa-miR-20a-5p |
| hsa-miR-29a-3p |
| hsa-miR-29b-3p |
| hsa-miR-29c-3p |
| hsa-miR-342-5p |
| hsa-miR-34a-5p |
| hsa-miR-34c-5p (Previous ID: hsa-miR-34c) |
| hsa-miR-375-3p |
| hsa-miR-449a (Previous ID: hsa-miR-449) |
| **GO:0051966 regulation of synaptic transmission, glutamatergic** |
| hsa-miR-142-3p |
| **GO:0055022 negative regulation of cardiac muscle tissue growth** |
| hsa-miR-25-3p |
| **GO:0055088 lipid homeostasis** |
| hsa-miR-128-3p |
| hsa-miR-148a-3p |
| **GO:0055118 negative regulation of cardiac muscle contraction** |
| hsa-miR-30e-5p |
| **GO:0060043 regulation of cardiac muscle cell proliferation** |
| hsa-miR-199a-5p |
| **GO:0060044 negative regulation of cardiac muscle cell proliferation** |
| hsa-miR-199a-5p |
| hsa-miR-199b-5p (Previous ID: hsa-miR-199b) |
| hsa-miR-200b-3p (Previous ID: hsa-miR-200b) |
| **GO:0060045 positive regulation of cardiac muscle cell proliferation** |
| hsa-miR-199b-3p / hsa-miR-199a-3p |
| hsa-miR-19b-3p (Previous ID: hsa-miR-19b) |
| hsa-miR-204-5p |
| hsa-miR-222-3p (Previous ID: hsa-miR-222) |
| hsa-miR-23b-3p |
| hsa-miR-548c-3p (Previous ID: hsa-miR-548c) |
| hsa-miR-590-3p |
| **GO:0060087 relaxation of vascular associated smooth muscle** |
| hsa-miR-153-3p |
| **GO:0060252 positive regulation of glial cell proliferation** |
| hsa-miR-125b-5p |
| **GO:0060253 negative regulation of glial cell proliferation** |
| hsa-miR-146a-5p |
| **GO:0060312 regulation of blood vessel remodeling** |
| hsa-miR-29b-3p |
| **GO:0060339 negative regulation of type I interferon-mediated signaling pathway** |
| hsa-miR-21-5p (Previous ID: hsa-miR-21) |
| **GO:0060354 negative regulation of cell adhesion molecule production** |
| hsa-miR-101-3p |
| hsa-miR-125a-5p |
| hsa-miR-155-5p |
| hsa-miR-206 |
| hsa-miR-20a-5p |
| hsa-miR-221-3p |
| hsa-miR-222-3p (Previous ID: hsa-miR-222) |
| hsa-miR-374a-5p (Previous ID: hsa-miR-374) |
| **GO:0060355 positive regulation of cell adhesion molecule production** |
| hsa-miR-144-3p |
| hsa-miR-520c-3p |
| **GO:0060371 regulation of atrial cardiac muscle cell membrane depolarization** |
| hsa-miR-26a-5p |
| **GO:0060394 negative regulation of pathway-restricted SMAD protein phosphorylation** |
| hsa-let-7g-5p |
| **GO:0060414 aorta smooth muscle tissue morphogenesis** |
| hsa-miR-143-3p |
| hsa-miR-145-5p |
| **GO:0060545 positive regulation of necroptotic process** |
| hsa-miR-103a-3p |
| hsa-miR-107 |
| hsa-miR-155-5p |
| **GO:0060546 negative regulation of necroptotic process** |
| hsa-miR-101-3p |
| hsa-miR-155-5p |
| hsa-miR-214-3p |
| hsa-miR-221-3p |
| hsa-miR-22-3p |
| hsa-miR-485-5p |
| hsa-miR-92a-3p |
| **GO:0060547 negative regulation of necrotic cell death** |
| hsa-miR-155-5p |
| hsa-miR-223-3p (Previous ID: hsa-miR-223) |
| hsa-miR-223-5p (Previous ID: hsa-miR-223*) |
| **GO:0060761 negative regulation of response to cytokine stimulus** |
| hsa-miR-138-5p |
| **GO:0060940 epithelial to mesenchymal transition involved in cardiac fibroblast development** |
| hsa-miR-21-5p (Previous ID: hsa-miR-21) |
| **GO:0061037 negative regulation of cartilage development** |
| hsa-miR-21-5p (Previous ID: hsa-miR-21) |
| **GO:0061044 negative regulation of vascular wound healing** |
| hsa-miR-155-5p |
| hsa-miR-200b-3p (Previous ID: hsa-miR-200b) |
| hsa-miR-34a-5p |
| **GO:0061045 negative regulation of wound healing** |
| hsa-miR-15b-5p |
| hsa-miR-892b |
|  |
|  |
|  |
| **GO:0061049 cell growth involved in cardiac muscle cell development** |
| hsa-miR-195-5p (Previous ID: hsa-miR-195) |
| hsa-miR-199a-5p |
| hsa-miR-23a-3p |
| hsa-miR-23b-3p |
| hsa-miR-24-3p |
| **GO:0061051 positive regulation of cell growth involved in cardiac muscle cell development** |
| hsa-miR-199a-5p |
| hsa-miR-19a-3p (Previous ID: hsa-miR-19a) |
| hsa-miR-19b-3p (Previous ID: hsa-miR-19b) |
| **GO:0061052 negative regulation of cell growth involved in cardiac muscle cell development** |
| hsa-miR-199a-5p |
| hsa-miR-199b-5p (Previous ID: hsa-miR-199b) |
| **GO:0061154 endothelial tube morphogenesis** |
| hsa-miR-21-5p (Previous ID: hsa-miR-21) |
| **GO:0061754 negative regulation of circulating fibrinogen levels** |
| hsa-miR-29a-3p |
| hsa-miR-29b-3p |
| hsa-miR-29c-3p |
| hsa-miR-409-3p |
| **GO:0061766 positive regulation of lung blood pressure** |
| hsa-miR-199a-5p |
| **GO:0061886 negative regulation of mini excitatory postsynaptic potential** |
| hsa-miR-30b-5p (Previous ID: hsa-miR-30b) |
| **GO:0061889 negative regulation of astrocyte activation** |
| hsa-miR-181b-5p |
| hsa-miR-181c-5p |
| **GO:0061890 positive regulation of astrocyte activation** |
| hsa-miR-142-3p |
| **GO:0062000 positive regulation of cardiac endothelial to mesenchymal transition** |
| hsa-miR-342-5p |
| **GO:0070102 interleukin-6-mediated signaling pathway** |
| hsa-miR-98-5p |
| **GO:0070104 negative regulation of interleukin-6-mediated signaling pathway** |
| hsa-let-7a-5p |
| hsa-let-7c-5p |
| hsa-let-7e-5p |
| hsa-miR-125a-5p |
| hsa-miR-125b-5p |
| hsa-miR-26a-5p |
| hsa-miR-98-5p |
| hsa-miR-99a-5p |
| **GO:0070301 cellular response to hydrogen peroxide** |
| hsa-miR-103a-3p |
| hsa-miR-107 |
| **GO:0070317 negative regulation of G0 to G1 transition** |
| hsa-miR-424-5p (Previous ID: hsa-miR-424) |
| hsa-miR-503-5p (Previous ID: hsa-miR-503) |
| **GO:0070328 triglyceride homeostasis** |
| hsa-miR-33a-5p (Previous ID: hsa-miR-33a) |
| hsa-miR-34a-5p |
| **GO:0070372 regulation of ERK1 and ERK2 cascade** |
| hsa-miR-145-5p |
| **GO:0070373 negative regulation of ERK1 and ERK2 cascade** |
| hsa-miR-185-3p |
| hsa-miR-200c-3p (Previous ID: hsa-miR-200c) |
| hsa-miR-21-5p (Previous ID: hsa-miR-21) |
| hsa-miR-221-3p |
|  |
|  |
| hsa-miR-424-5p (Previous ID: hsa-miR-424) |
| hsa-miR-503-5p (Previous ID: hsa-miR-503) |
| **GO:0070374 positive regulation of ERK1 and ERK2 cascade** |
| hsa-let-7b-5p |
| hsa-miR-126-3p |
| hsa-miR-21-5p (Previous ID: hsa-miR-21) |
| hsa-miR-221-3p |
| hsa-miR-222-3p (Previous ID: hsa-miR-222) |
| hsa-miR-23a-3p |
| hsa-miR-23b-3p |
| hsa-miR-24-3p |
| hsa-miR-27a-3p |
| hsa-miR-27b-3p |
| **GO:0070498 interleukin-1-mediated signaling pathway** |
| hsa-miR-146a-5p |
| hsa-miR-155-5p |
| **GO:0070555 response to interleukin-1** |
| hsa-let-7i-5p |
| hsa-miR-101-3p |
| hsa-miR-142-3p |
| hsa-miR-204-5p |
| **GO:0070858 negative regulation of bile acid biosynthetic process** |
| hsa-miR-33a-5p (Previous ID: hsa-miR-33a**)** |
| **GO:0070885 negative regulation of calcineurin-NFAT signaling cascade** |
| hsa-miR-124-3p |
| **GO:0071072 negative regulation of phospholipid biosynthetic process** |
| hsa-miR-30c-5p |
| **GO:0071158 positive regulation of cell cycle arrest** |
| hsa-miR-200b-3p (Previous ID: hsa-miR-200b) |
| hsa-miR-34a-5p |
| **GO:0071221 cellular response to bacterial lipopeptide** |
| hsa-miR-19a-3p (Previous ID: hsa-miR-19a) |
| hsa-miR-19b-3p (Previous ID: hsa-miR-19b) |
| **GO:0071222 cellular response to lipopolysaccharide** |
| hsa-miR-146a-5p |
| hsa-miR-146b-5p |
| hsa-miR-17-5p (Previous ID: hsa-miR-17) |
| hsa-miR-20a-5p |
| hsa-miR-21-5p (Previous ID: hsa-miR-21) |
| hsa-miR-6869-5p |
| hsa-miR-766-3p (Previous ID: hsa-miR-766) |
| **GO:0071305 cellular response to vitamin D** |
| hsa-miR-125b-5p |
| **GO:0071333 cellular response to glucose stimulus** |
| hsa-let-7g-5p |
| hsa-miR-146a-5p |
| hsa-miR-15a-5p (Previous ID: hsa-miR-15a) |
| hsa-miR-16-5p |
| **GO:0071345 cellular response to cytokine stimulus** |
| hsa-miR-146a-5p |
| **GO:0071347 cellular response to interleukin-1** |
| hsa-miR-766-3p (Previous ID: hsa-miR-766) |
| **GO:0071354 cellular response to interleukin-6** |
| hsa-miR-125b-5p |
| hsa-miR-149-3p |
| **GO:0071356 cellular response to tumor necrosis factor** |
| hsa-miR-181b-5p |
| hsa-miR-20b-5p (Previous ID: hsa-miR-20b) |
| hsa-miR-31-5p |
| hsa-miR-766-3p (Previous ID: hsa-miR-766) |
| **GO:0071397 cellular response to cholesterol** |
| hsa-miR-182-5p (Previous ID: hsa-miR-182) |
| **GO:0071398 cellular response to fatty acid** |
| hsa-miR-92a-3p |
| **GO:0071404 cellular response to low-density lipoprotein particle stimulus** |
| hsa-miR-155-5p |
| hsa-miR-302a-3p (Previous ID: hsa-miR-302a) |
| hsa-miR-758-3p (Previous ID: hsa-miR-758) |
| hsa-miR-92a-3p |
| **GO:0071456 cellular response to hypoxia** |
| hsa-miR-106b-5p (Previous ID: hsa-miR-106b) |
| hsa-miR-124-3p |
| hsa-miR-126-3p |
| hsa-miR-146a-5p |
| hsa-miR-155-5p |
| hsa-miR-17-5p (Previous ID: hsa-miR-17) |
| hsa-miR-214-3p |
| hsa-miR-34a-5p |
| **GO:0071499 cellular response to laminar fluid shear stress** |
| hsa-miR-126-5p |
| **GO:0071560 cellular response to transforming growth factor beta stimulus** |
| hsa-miR-27a-5p |
| **GO:0071639 positive regulation of monocyte chemotactic protein-1 production** |
| hsa-miR-92a-3p |
| **GO:0071644 negative regulation of chemokine (C-C motif) ligand 4 production** |
| hsa-miR-155-5p |
| **GO:0071672 negative regulation of smooth muscle cell chemotaxis** |
| hsa-miR-34a-5p |
| **GO:0071864 positive regulation of cell proliferation in bone marrow** |
| hsa-miR-27b-3p |
| **GO:0071901 negative regulation of protein serine/threonine kinase activity** |
| hsa-miR-20a-5p |
| hsa-miR-24-3p |
| **GO:0071902 positive regulation of protein serine/threonine kinase activity** |
| hsa-miR-26b-5p (Previous ID: hsa-miR-26b) |
| **GO:0072125 negative regulation of glomerular mesangial cell proliferation** |
| hsa-miR-125a-5p |
| **GO:0090024 negative regulation of neutrophil chemotaxis** |
| hsa-miR-223-3p (Previous ID: hsa-miR-223) |
| **GO:0090050 positive regulation of cell migration involved in sprouting angiogenesis** |
| hsa-let-7f-5p (Previous ID: hsa-let-7f) |
| hsa-miR-101-3p |
| hsa-miR-10a-5p (Previous ID: hsa-miR-10a) |
| hsa-miR-10b-5p (Previous ID: hsa-miR-10b) |
| hsa-miR-126-3p |
| hsa-miR-126-5p |
| hsa-miR-132-3p (Previous ID: hsa-miR-132) |
| hsa-miR-146a-5p |
| hsa-miR-150-5p (Previous ID: hsa-miR-150) |
| hsa-miR-23a-3p |
| hsa-miR-23b-3p |
| hsa-miR-27a-3p |
| hsa-miR-27b-3p |
| hsa-miR-296-5p |
| hsa-miR-31-5p |
| **GO:0090051 negative regulation of cell migration involved in sprouting angiogenesis** |
| hsa-miR-146a-5p |
| hsa-miR-146b-5p |
| hsa-miR-149-3p |
| hsa-miR-149-5p (Previous ID: hsa-miR-149) |
| hsa-miR-155-5p |
| hsa-miR-15a-5p (Previous ID: hsa-miR-15a) |
| hsa-miR-16-5p |
| hsa-miR-193a-3p |
| hsa-miR-196a-5p |
| hsa-miR-199b-3p / hsa-miR-199a-3p |
| hsa-miR-19b-3p (Previous ID: hsa-miR-19b) |
| hsa-miR-200c-3p (Previous ID: hsa-miR-200c) |
| hsa-miR-206 |
| hsa-miR-20a-5p |
| hsa-miR-221-3p |
| hsa-miR-22-3p |
| hsa-miR-26a-5p |
| hsa-miR-320a-3p |
| hsa-miR-361-5p (Previous ID: hsa-miR-361) |
| hsa-miR-410-3p |
| hsa-miR-424-5p (Previous ID: hsa-miR-424) |
| hsa-miR-483-5p |
| hsa-miR-497-5p (Previous ID: hsa-miR-497) |
| hsa-miR-503-5p (Previous ID: hsa-miR-503) |
| **GO:0090090 negative regulation of canonical Wnt signaling pathway** |
| hsa-miR-29b-3p |
| hsa-miR-665 |
| **GO:0090107 regulation of high-density lipoprotein particle assembly** |
| hsa-miR-144-3p |
| **GO:0090263 positive regulation of canonical Wnt signaling pathway** |
| hsa-miR-145-5p |
| hsa-miR-222-3p (Previous ID: hsa-miR-222) |
| **GO:0090272 negative regulation of fibroblast growth factor production** |
| hsa-miR-146b-5p |
| hsa-miR-152-3p |
| hsa-miR-195-5p (Previous ID: hsa-miR-195) |
| hsa-miR-205-5p (Previous ID: hsa-miR-205) |
| **GO:0090281 negative regulation of calcium ion import** |
| hsa-miR-34a-5p |
| **GO:0090298 negative regulation of mitochondrial DNA replication** |
| hsa-miR-155-5p |
| **GO:0090322 regulation of superoxide metabolic process** |
| hsa-miR-27b-3p |
| **GO:0090370 negative regulation of cholesterol efflux** |
| hsa-miR-128-3p |
| hsa-miR-130b-3p (Previous ID: hsa-miR-130b) |
| hsa-miR-144-3p |
| hsa-miR-148a-3p |
| hsa-miR-19b-3p (Previous ID: hsa-miR-19b) |
| hsa-miR-26a-5p |
| hsa-miR-27a-3p |
| hsa-miR-27b-3p |
| hsa-miR-301b-3p (Previous ID: hsa-miR-301b) |
| hsa-miR-302a-3p (Previous ID: hsa-miR-302a) |
| hsa-miR-33a-5p (Previous ID: hsa-miR-33a) |
| hsa-miR-33b-5p (Previous ID: hsa-miR-33b) |
| hsa-miR-758-3p (Previous ID: hsa-miR-758) |
| **GO:0097006 regulation of plasma lipoprotein particle levels** |
| hsa-miR-19b-3p (Previous ID: hsa-miR-19b) |
| **GO:0097028 dendritic cell differentiation** |
| hsa-miR-155-5p |
| **GO:0097699 vascular endothelial cell response to fluid shear stress** |
| hsa-miR-92a-3p |
| **GO:0097756 negative regulation of blood vessel diameter** |
| hsa-miR-92a-3p |
| **GO:0098586 cellular response to virus** |
| hsa-miR-130a-3p (Previous ID: hsa-miR-130a) |
| hsa-miR-146a-5p |
| hsa-miR-21-5p (Previous ID: hsa-miR-21) |
| hsa-miR-29b-3p |
| hsa-miR-30c-5p |
| **GO:0098806 deadenylation involved in gene silencing by miRNA** |
| hsa-let-7a-5p |
| **GO:0106016 positive regulation of inflammatory response to wounding** |
| hsa-miR-21-5p (Previous ID: hsa-miR-21) |
| **GO:0106090 positive regulation of cell adhesion involved in sprouting angiogenesis** |
| hsa-miR-27b-3p |
| **GO:0106128 negative regulation of store-operated calcium entry** |
| hsa-miR-424-5p (Previous ID: hsa-miR-424) |
| **GO:0110015 positive regulation of elastin catabolic process** |
| hsa-miR-181b-5p |
| **GO:0110023 negative regulation of cardiac muscle myoblast proliferation** |
| hsa-miR-10a-5p (Previous ID: hsa-miR-10a) |
| hsa-miR-134-5p |
| hsa-miR-204-5p |
| **GO:0110058 positive regulation of blood vessel endothelial cell differentiation** |
| hsa-miR-34a-5p |
| **GO:0110059 negative regulation of blood vessel endothelial cell differentiation** |
| hsa-miR-10a-3p (Previous ID: hsa-miR-10a*) |
| **GO:0110081 negative regulation of placenta blood vessel development** |
| hsa-miR-16-5p |
| **GO:0110114 negative regulation of lipid transporter activity** |
| hsa-miR-30c-5p |
| hsa-miR-34a-5p |
| **GO:0120041 positive regulation of macrophage proliferation** |
| hsa-miR-181b-5p |
| **GO:0120132 positive regulation of apoptotic process in bone marrow cell** |
| hsa-miR-146a-5p |
| **GO:0120190 negative regulation of bile acid secretion** |
| hsa-miR-33a-5p (Previous ID: hsa-miR-33a) |
| **GO:0140052 cellular response to oxidised low-density lipoprotein particle stimulus** |
| hsa-miR-146a-5p |
| hsa-miR-155-5p |
| **GO:0140076 negative regulation of lipoprotein transport** |
| hsa-miR-128-3p |
| hsa-miR-148a-3p |
| **GO:0140193 regulation of adenylate cyclase-inhibiting adrenergic receptor signaling pathway involved in heart process** |
| hsa-miR-30e-5p |
| **GO:0150078 positive regulation of neuroinflammatory response** |
| hsa-miR-128-3p |
| hsa-miR-142-3p |
| hsa-miR-206 |
| **GO:0150079 negative regulation of neuroinflammatory response** |
| hsa-miR-124-3p |
| hsa-miR-195-5p (Previous ID: hsa-miR-195) |
| hsa-miR-26a-5p |
| **GO:0150128 negative regulation of interleukin-33 production** |
| hsa-miR-378a-3p |
| hsa-miR-200b-3p (Previous ID: hsa-miR-200b) |
| hsa-miR-200c-3p (Previous ID: hsa-miR-200c) |
|  |
|  |
|  |
| **GO:0150138 negative regulation of interleukin-37 production** |
| hsa-miR-657 |
| **GO:0150159 negative regulation of interleukin-34 production** |
| hsa-miR-28-5p (Previous ID: hsa-miR-28) |
| hsa-miR-31-5p |
| **GO:0150190 negative regulation of interleukin-32 production** |
| hsa-miR-29b-3p |
| **GO:1900004 negative regulation of serine-type endopeptidase activity** |
| hsa-miR-19b-3p (Previous ID: hsa-miR-19b) |
| **GO:1900015 regulation of cytokine production involved in inflammatory response** |
| hsa-miR-197-3p (Previous ID: hsa-miR-197) |
| hsa-miR-98-5p |
| **GO:1900016 negative regulation of cytokine production involved in inflammatory response** |
| hsa-miR-125a-5p |
| hsa-miR-129-5p |
| hsa-miR-136-5p |
| hsa-miR-146a-5p |
| hsa-miR-155-5p |
| hsa-miR-16-5p |
| hsa-miR-203a-3p |
| hsa-miR-221-3p |
| hsa-miR-222-3p (Previous ID: hsa-miR-222) |
| hsa-miR-26a-5p |
| hsa-miR-378a-3p |
| hsa-miR-93-5p |
| hsa-miR-98-5p |
| **GO:1900017 positive regulation of cytokine production involved in inflammatory response** |
| hsa-miR-17-5p (Previous ID: hsa-miR-17) |
| hsa-miR-21-5p (Previous ID: hsa-miR-21) |
| **GO:1900039 positive regulation of cellular response to hypoxia** |
| hsa-miR-21-5p (Previous ID: hsa-miR-21) |
| **GO:1900045 negative regulation of protein K63-linked ubiquitination** |
| hsa-miR-138-5p |
| **GO:1900082 negative regulation of arginine catabolic process** |
| hsa-miR-21-5p (Previous ID: hsa-miR-21) |
| **GO:1900087 positive regulation of G1/S transition of mitotic cell cycle** |
| hsa-miR-214-3p |
| hsa-miR-221-3p |
| hsa-miR-222-3p (Previous ID: hsa-miR-222) |
| hsa-miR-29a-3p |
| hsa-miR-520a-3p (Previous ID: hsa-miR-520a) |
| hsa-miR-590-3p |
| **GO:1900131 negative regulation of lipid binding** |
| hsa-miR-27a-3p |
| hsa-miR-27b-3p |
| **GO:1900149 positive regulation of Schwann cell migration** |
| hsa-miR-221-3p |
| hsa-miR-222-3p (Previous ID: hsa-miR-222) |
| **GO:1900181 negative regulation of protein localization to nucleus** |
| hsa-miR-155-5p |
| **GO:1900222 negative regulation of amyloid-beta clearance** |
| hsa-miR-1908-5p |
| hsa-miR-34a-5p |
| **GO:1900239 regulation of phenotypic switching** |
| hsa-miR-143-3p |
| hsa-miR-145-5p |
| hsa-miR-199b-5p (Previous ID: hsa-miR-199b) |
| **GO:1900272 negative regulation of long-term synaptic potentiation** |
| hsa-miR-30b-5p (Previous ID: hsa-miR-30b) |
| **GO:1900408 negative regulation of cellular response to oxidative stress** |
| hsa-miR-132-3p (Previous ID: hsa-miR-132) |
| **GO:1900425 negative regulation of defense response to bacterium** |
| hsa-miR-181b-5p |
| **GO:1900745 positive regulation of p38MAPK cascade** |
| hsa-miR-181a-5p |
| hsa-miR-181b-5p |
| **GO:1900747 negative regulation of vascular endothelial growth factor signaling pathway** |
| hsa-miR-16-5p |
| hsa-miR-199b-3p / hsa-miR-199a-3p |
| hsa-miR-342-5p |
| hsa-miR-424-5p (Previous ID: hsa-miR-424) |
| **GO:1900748 positive regulation of vascular endothelial growth factor signaling pathway** |
| hsa-miR-21-5p (Previous ID: hsa-miR-21) |
| **GO:1901164 negative regulation of trophoblast cell migration** |
| hsa-miR-15b-5p |
| hsa-miR-16-5p |
| **GO:1901202 negative regulation of extracellular matrix assembly** |
| hsa-miR-29b-3p |
| **GO:1901215 negative regulation of neuron death** |
| hsa-miR-200c-3p (Previous ID: hsa-miR-200c) |
| **GO:1901223 negative regulation of NIK/NF-kappaB signaling** |
| hsa-miR-132-3p (Previous ID: hsa-miR-132) |
| hsa-miR-146a-5p |
| hsa-miR-149-5p  (Previous ID: hsa-miR-149) |
| hsa-miR-204-5p |
| hsa-miR-21-5p (Previous ID: hsa-miR-21) |
| hsa-miR-223-3p (Previous ID: hsa-miR-223) |
| hsa-miR-27b-5p (Previous ID: hsa-miR-27b*) |
| hsa-miR-29b-3p |
| hsa-miR-590-3p |
| hsa-miR-766-3p (Previous ID: hsa-miR-766) |
| **GO:1901224 positive regulation of NIK/NF-kappaB signaling** |
| hsa-miR-182-5p (Previous ID: hsa-miR-182) |
| **GO:1901295 regulation of canonical Wnt signaling pathway involved in cardiac muscle cell fate commitment** |
| hsa-miR-19b-3p (Previous ID: hsa-miR-19b) |
| **GO:1901299 negative regulation of hydrogen peroxide-mediated programmed cell death** |
| hsa-let-7b-5p |
| hsa-miR-21-5p (Previous ID: hsa-miR-21) |
| **GO:1901300 positive regulation of hydrogen peroxide-mediated programmed cell death** |
| hsa-miR-17-5p (Previous ID: hsa-miR-17) |
| **GO:1901342 regulation of vasculature development** |
| hsa-miR-223-3p (Previous ID: hsa-miR-223) |
| **GO:1901646 negative regulation of synoviocyte proliferation** |
| hsa-miR-181c-5p |
| **GO:1901670 negative regulation of superoxide dismutase activity** |
| hsa-miR-21-5p (Previous ID: hsa-miR-21) |
| **GO:1901726 negative regulation of histone deacetylase activity** |
| hsa-miR-182-5p (Previous ID: hsa-miR-182) |
| **GO:1901985 positive regulation of protein acetylation** |
| hsa-miR-34a-5p |
| **GO:1902004 positive regulation of amyloid-beta formation** |
| hsa-miR-206 |
| **GO:1902034 negative regulation of hematopoietic stem cell proliferation** |
| hsa-miR-221-3p |
| hsa-miR-222-3p (Previous ID: hsa-miR-222) |
| **GO:1902083 negative regulation of peptidyl-cysteine S-nitrosylation** |
| hsa-miR-132-3p (Previous ID: hsa-miR-132) |
| hsa-miR-212-3p (Previous ID: hsa-miR-212) |
| **GO:1902109 negative regulation of mitochondrial membrane permeability involved in apoptotic process** |
| hsa-miR-17-5p (Previous ID: hsa-miR-17) |
| **GO:1902110 positive regulation of mitochondrial membrane permeability involved in apoptotic process** |
| hsa-miR-29a-3p |
| hsa-miR-29b-3p |
| hsa-miR-29c-3p |
| **GO:1902254 negative regulation of intrinsic apoptotic signaling pathway by p53 class mediator** |
| hsa-miR-21-5p (Previous ID: hsa-miR-21) |
| **GO:1902396 protein localization to bicellular tight junction** |
| hsa-miR-155-5p |
| **GO:1902430 negative regulation of amyloid-beta formation** |
| hsa-miR-361-3p |
| hsa-miR-455-3p |
| hsa-miR-455-5p (Previous ID: hsa-miR-455) |
| **GO:1902461 negative regulation of mesenchymal stem cell proliferation** |
| hsa-miR-16-5p |
| hsa-miR-29b-3p |
| **GO:1902504 regulation of signal transduction involved in mitotic G2 DNA damage checkpoint** |
| hsa-miR-21-5p (Previous ID: hsa-miR-21) |
| **GO:1902511 negative regulation of apoptotic DNA fragmentation** |
| hsa-miR-146a-5p |
| **GO:1902514 regulation of calcium ion transmembrane transport via high voltage-gated calcium channel** |
| hsa-miR-21-5p (Previous ID: hsa-miR-21) |
| **GO:1902532 negative regulation of intracellular signal transduction** |
| hsa-miR-146a-5p |
| **GO:1902564 negative regulation of neutrophil activation** |
| hsa-miR-125a-5p |
| **GO:1902565 positive regulation of neutrophil activation** |
| hsa-miR-155-5p |
| **GO:1902714 negative regulation of interferon-gamma production** |
| hsa-miR-24-3p |
| **GO:1902731 negative regulation of chondrocyte proliferation** |
| hsa-miR-21-5p (Previous ID: hsa-miR-21) |
| **GO:1902807 negative regulation of cell cycle G1/S phase transition** |
| hsa-miR-10a-5p (Previous ID: hsa-miR-10a) |
| hsa-miR-26a-5p |
| hsa-miR-503-5p (Previous ID: hsa-miR-503) |
| **GO:1902949 positive regulation of tau-protein kinase activity** |
| hsa-miR-125b-1-3p |
| hsa-miR-26b-5p (Previous ID: hsa-miR-26b) |
| **GO:1902951 negative regulation of dendritic spine maintenance** |
| hsa-miR-30b-5p (Previous ID: hsa-miR-30b) |
| **GO:1902992 negative regulation of amyloid precursor protein catabolic process** |
| hsa-miR-107 |
| **GO:1903063 negative regulation of reverse cholesterol transport** |
| hsa-miR-144-3p |
| hsa-miR-19b-3p (Previous ID: hsa-miR-19b) |
| hsa-miR-33a-5p (Previous ID: hsa-miR-33a) |
| **GO:1903078 positive regulation of protein localization to plasma membrane** |
| hsa-miR-223-3p (Previous ID: hsa-miR-223) |
| **GO:1903122 negative regulation of TRAIL-activated apoptotic signaling pathway** |
| hsa-miR-221-3p |
| hsa-miR-222-3p (Previous ID: hsa-miR-222) |
| **GO:1903202 negative regulation of oxidative stress-induced cell death** |
| hsa-miR-195-5p (Previous ID: hsa-miR-195) |
| hsa-miR-19a-3p (Previous ID: hsa-miR-19a) |
| hsa-miR-29b-3p |
|  |
|  |
|  |
| **GO:1903206 negative regulation of hydrogen peroxide-induced cell death** |
| hsa-miR-17-3p (Previous ID: hsa-miR-17*) |
| hsa-miR-155-5p |
| hsa-miR-92a-3p |
| **GO:1903243 negative regulation of cardiac muscle hypertrophy in response to stress** |
| hsa-miR-214-5p (Previous ID: hsa-miR-214*) |
| hsa-miR-25-3p |
| **GO:1903244 positive regulation of cardiac muscle hypertrophy in response to stress** |
| hsa-miR-155-5p |
| hsa-miR-17-5p (Previous ID: hsa-miR-17) |
| hsa-miR-199a-5p |
| hsa-miR-20a-5p |
| hsa-miR-214-3p |
| hsa-miR-34c-5p (Previous ID: hsa-miR-34c) |
| **GO:1903347 negative regulation of bicellular tight junction assembly** |
| hsa-miR-142-5p |
| hsa-miR-155-5p |
| **GO:1903427 negative regulation of reactive oxygen species biosynthetic process** |
| hsa-miR-181a-5p |
| hsa-miR-21-5p (Previous ID: hsa-miR-21) |
| hsa-miR-590-5p |
| **GO:1903428 positive regulation of reactive oxygen species biosynthetic process** |
| hsa-miR-155-5p |
| hsa-miR-24-3p |
| **GO:1903588 negative regulation of blood vessel endothelial cell proliferation involved in sprouting angiogenesis** |
| hsa-miR-149-3p |
| hsa-miR-149-5p (Previous ID: hsa-miR-149) |
| hsa-miR-155-5p |
| hsa-miR-15a-5p (Previous ID: hsa-miR-15a) |
| hsa-miR-15b-5p |
| hsa-miR-16-5p |
| hsa-miR-193a-3p |
| hsa-miR-222-3p (Previous ID: hsa-miR-222) |
| hsa-miR-24-3p |
| hsa-miR-26a-5p |
| hsa-miR-342-5p |
| hsa-miR-361-5p (Previous ID: hsa-miR-361) |
| hsa-miR-410-3p |
| hsa-miR-424-5p (Previous ID: hsa-miR-424) |
| hsa-miR-483-5p |
| hsa-miR-497-5p (Previous ID: hsa-miR-497) |
| hsa-miR-503-5p (Previous ID: hsa-miR-503) |
| **GO:1903589 positive regulation of blood vessel endothelial cell proliferation involved in sprouting angiogenesis** |
| hsa-let-7b-5p |
| hsa-miR-101-3p |
| hsa-miR-10a-5p (Previous ID: hsa-miR-10a) |
| hsa-miR-10b-5p (Previous ID: hsa-miR-10b) |
| hsa-miR-124-3p |
| hsa-miR-126-3p |
| hsa-miR-126-5p |
| hsa-miR-132-3p (Previous ID: hsa-miR-132) |
| hsa-miR-146a-5p |
| hsa-miR-21-5p (Previous ID: hsa-miR-21) |
| hsa-miR-23a-3p |
| hsa-miR-23b-3p |
| hsa-miR-27a-3p |
| hsa-miR-27b-3p |
| hsa-miR-503-5p (Previous ID: hsa-miR-503) |
| **GO:1903609 negative regulation of inward rectifier potassium channel activity** |
| hsa-miR-212-3p (Previous ID: hsa-miR-212) |
| hsa-miR-26a-5p |
| **GO:1903670 regulation of sprouting angiogenesis** |
| hsa-miR-23a-3p |
| **GO:1903671 negative regulation of sprouting angiogenesis** |
| hsa-miR-138-5p |
| hsa-miR-17-5p (Previous ID: hsa-miR-17) |
| hsa-miR-18a-5p (Previous ID: hsa-miR-18a) |
| hsa-miR-19a-3p (Previous ID: hsa-miR-19a) |
| hsa-miR-20a-5p |
| hsa-miR-221-3p |
| hsa-miR-23b-3p |
| hsa-miR-30c-5p |
| hsa-miR-30e-5p |
| hsa-miR-34a-5p |
| hsa-miR-34c-5p (Previous ID: hsa-miR-34c) |
| hsa-miR-375-3p |
| hsa-miR-92a-3p |
| **GO:1903672 positive regulation of sprouting angiogenesis** |
| hsa-miR-125a-5p |
| hsa-miR-126-3p |
| hsa-miR-155-5p |
| hsa-miR-30b-5p (Previous ID: hsa-miR-30b) |
| hsa-miR-31-5p |
| hsa-miR-92a-3p |
| **GO:1903691 positive regulation of wound healing, spreading of epidermal cells** |
| hsa-miR-221-3p |
| **GO:1903720 negative regulation of I-kappaB phosphorylation** |
| hsa-miR-27b-5p (Previous ID: hsa-miR-27b*) |
| **GO:1903753 negative regulation of p38MAPK cascade** |
| hsa-miR-138-5p |
| hsa-miR-20a-5p |
| **GO:1903766 positive regulation of potassium ion export across plasma membrane** |
| hsa-miR-21-5p (Previous ID: hsa-miR-21) |
| **GO:1903769 negative regulation of cell proliferation in bone marrow** |
| hsa-miR-10a-3p (Previous ID: hsa-miR-10a*) |
| **GO:1903779 regulation of cardiac conduction** |
| hsa-miR-19a-3p (Previous ID: hsa-miR-19a) |
| **GO:1903817 negative regulation of voltage-gated potassium channel activity** |
| hsa-miR-153-3p |
| **GO:1903845 negative regulation of cellular response to transforming growth factor beta stimulus** |
| hsa-miR-29b-3p |
| **GO:1903847 regulation of aorta morphogenesis** |
| hsa-miR-205-5p (Previous ID: hsa-miR-205) |
| hsa-miR-29b-3p |
| **GO:1903900 regulation of viral life cycle** |
| hsa-miR-130a-3p (Previous ID: hsa-miR-130a) |
| **GO:1903901 negative regulation of viral life cycle** |
| hsa-miR-30c-5p |
| **GO:1903979 negative regulation of microglial cell activation** |
| hsa-miR-124-3p |
| hsa-miR-155-5p |
| **GO:1903980 positive regulation of microglial cell activation** |
| hsa-miR-128-3p |
| hsa-miR-142-3p |
| hsa-miR-155-5p |
| **GO:1904018 positive regulation of vasculature development** |
| hsa-miR-126-3p |
| **GO:1904027 negative regulation of collagen fibril organization** |
| hsa-miR-29b-3p |
| **GO:1904036 negative regulation of epithelial cell apoptotic process** |
| hsa-miR-182-5p (Previous ID: hsa-miR-182) |
| **GO:1904046 negative regulation of vascular endothelial growth factor production** |
| hsa-miR-106a-5p (Previous ID: hsa-miR-106a) |
| hsa-miR-107 |
| hsa-miR-125a-5p |
| hsa-miR-134-5p |
| hsa-miR-146a-5p |
| hsa-miR-15a-5p (Previous ID: hsa-miR-15a) |
| hsa-miR-15b-5p |
| hsa-miR-16-5p |
| hsa-miR-17-5p (Previous ID: hsa-miR-17) |
| hsa-miR-195-5p (Previous ID: hsa-miR-195) |
| hsa-miR-199b-3p / hsa-miR-199a-3p |
| hsa-miR-205-5p (Previous ID: hsa-miR-205) |
| hsa-miR-20a-5p |
| hsa-miR-20b-5p (Previous ID: hsa-miR-20b) |
| hsa-miR-302d-3p (Previous ID: hsa-miR-302d) |
| hsa-miR-34a-5p |
| hsa-miR-361-5p (Previous ID: hsa-miR-361) |
| hsa-miR-93-5p |
| **GO:1904465 negative regulation of matrix metallopeptidase secretion** |
| hsa-miR-146a-5p |
| hsa-miR-199b-3p / hsa-miR-199a-3p |
| hsa-miR-19a-3p (Previous ID: hsa-miR-19a) |
| hsa-miR-19b-3p (Previous ID: hsa-miR-19b) |
| hsa-miR-29b-3p |
| hsa-miR-766-3p (Previous ID: hsa-miR-766) |
| **GO:1904468 negative regulation of tumor necrosis factor secretion** |
| hsa-miR-101-3p |
| hsa-miR-125a-5p |
| hsa-miR-204-5p |
| hsa-miR-6869-5p |
| hsa-miR-98-5p |
| **GO:1904469 positive regulation of tumor necrosis factor secretion** |
| hsa-miR-144-3p |
| **GO:1904598 positive regulation of connective tissue replacement involved in inflammatory response wound healing** |
| hsa-miR-17-5p (Previous ID: hsa-miR-17) |
| **GO:1904639 cellular response to resveratrol** |
| hsa-miR-21-5p (Previous ID: hsa-miR-21) |
| **GO:1904645 response to amyloid-beta** |
| hsa-miR-200c-3p (Previous ID: hsa-miR-200c) |
| **GO:1904646 cellular response to amyloid-beta** |
| hsa-let-7f-5p (Previous ID: hsa-let-7f) |
| hsa-miR-106b-5p (Previous ID: hsa-miR-106b) |
| hsa-miR-146a-5p |
| hsa-miR-200a-3p (Previous ID: hsa-miR-200a) |
| hsa-miR-98-5p |
| **GO:1904673 negative regulation of somatic stem cell population maintenance** |
| hsa-miR-145-5p |
| **GO:1904676 negative regulation of somatic stem cell division** |
| hsa-miR-145-5p |
| **GO:1904684 negative regulation of metalloendopeptidase activity** |
| hsa-miR-152-3p |
| hsa-miR-195-5p (Previous ID: hsa-miR-195) |
| hsa-miR-24-3p |
|  |
|  |
| hsa-miR-29c-3p |
| hsa-miR-892b |
| **GO:1904685 positive regulation of metalloendopeptidase activity** |
| hsa-miR-17-5p (Previous ID: hsa-miR-17) |
| hsa-miR-205-5p (Previous ID: hsa-miR-205) |
| hsa-miR-21-5p (Previous ID: hsa-miR-21) |
| hsa-miR-29b-3p |
| hsa-miR-92a-3p |
| **GO:1904695 positive regulation of vascular associated smooth muscle contraction** |
| hsa-miR-21-5p (Previous ID: hsa-miR-21) |
| **GO:1904706 negative regulation of vascular associated smooth muscle cell proliferation** |
| hsa-miR-124-3p |
| hsa-miR-15a-5p (Previous ID: hsa-miR-15a) |
| hsa-miR-182-5p (Previous ID: hsa-miR-182) |
| hsa-miR-185-3p |
| hsa-miR-214-3p |
| hsa-miR-223-3p (Previous ID: hsa-miR-223) |
| hsa-miR-339-5p |
| hsa-miR-34a-5p |
| hsa-miR-362-3p |
| hsa-miR-424-5p (Previous ID: hsa-miR-424) |
| hsa-miR-503-5p (Previous ID: hsa-miR-503) |
| hsa-miR-638 |
| hsa-miR-665 |
| **GO:1904707 positive regulation of vascular associated smooth muscle cell proliferation** |
| hsa-miR-130a-3p (Previous ID: hsa-miR-130a) |
| hsa-miR-135b-5p |
| hsa-miR-17-5p (Previous ID: hsa-miR-17) |
| hsa-miR-20a-5p |
| hsa-miR-214-3p |
| hsa-miR-214-5p (Previous ID: hsa-miR-214*) |
| hsa-miR-21-5p (Previous ID: hsa-miR-21) |
| hsa-miR-221-3p |
| hsa-miR-222-3p (Previous ID: hsa-miR-222) |
| hsa-miR-26a-5p |
| hsa-miR-27a-3p |
| hsa-miR-27b-3p |
| hsa-miR-301a-3p (Previous ID: hsa-miR-301) |
| **GO:1904728 positive regulation of replicative senescence** |
| hsa-miR-21-5p (Previous ID: hsa-miR-21) |
| **GO:1904747 positive regulation of apoptotic process involved in development** |
| hsa-miR-19b-3p (Previous ID: hsa-miR-19b) |
| **GO:1904753 negative regulation of vascular associated smooth muscle cell migration** |
| hsa-miR-15a-5p (Previous ID: hsa-miR-15a) |
| hsa-miR-182-5p (Previous ID: hsa-miR-182) |
| hsa-miR-214-3p |
| hsa-miR-21-5p (Previous ID: hsa-miR-21) |
| hsa-miR-218-5p |
| hsa-miR-223-3p (Previous ID: hsa-miR-223) |
| hsa-miR-34a-5p |
| hsa-miR-362-3p |
| hsa-miR-424-5p (Previous ID: hsa-miR-424) |
| hsa-miR-503-5p (Previous ID: hsa-miR-503) |
| hsa-miR-638 |
| hsa-miR-665 |
| **GO:1904754 positive regulation of vascular associated smooth muscle cell migration** |
| hsa-miR-135b-5p |
| hsa-miR-143-3p |
| hsa-miR-20a-5p |
| hsa-miR-21-5p (Previous ID: hsa-miR-21) |
| hsa-miR-221-3p |
| hsa-miR-26a-5p |
| **GO:1904762 positive regulation of myofibroblast differentiation** |
| hsa-miR-21-5p (Previous ID: hsa-miR-21) |
| **GO:1904827 negative regulation of hydrogen sulfide biosynthetic process** |
| hsa-miR-21-5p (Previous ID: hsa-miR-21) |
| **GO:1904830 negative regulation of aortic smooth muscle cell differentiation** |
| hsa-miR-21-5p (Previous ID: hsa-miR-21) |
| **GO:1904831 positive regulation of aortic smooth muscle cell differentiation** |
| hsa-miR-424-5p (Previous ID: hsa-miR-424) |
| **GO:1904848 negative regulation of cell chemotaxis to fibroblast growth factor** |
| hsa-miR-15a-5p (Previous ID: hsa-miR-15a) |
| hsa-miR-16-5p |
| **GO:1904858 negative regulation of endothelial cell chemotaxis to vascular endothelial growth factor** |
| hsa-miR-16-5p |
| hsa-miR-424-5p (Previous ID: hsa-miR-424) |
| **GO:1904893 negative regulation of receptor signaling pathway via STAT** |
| hsa-let-7c-5p |
| hsa-let-7e-5p |
| hsa-miR-125a-5p |
| hsa-miR-125b-5p |
| hsa-miR-149-3p |
| hsa-miR-99a-5p |
| **GO:1904905 negative regulation of endothelial cell-matrix adhesion via fibronectin** |
| hsa-miR-92a-3p |
| **GO:1904989 positive regulation of endothelial cell activation** |
| hsa-miR-92a-3p |
| **GO:1904995 negative regulation of leukocyte adhesion to vascular endothelial cell** |
| hsa-let-7e-5p |
| hsa-let-7g-5p |
| hsa-miR-125a-5p |
| hsa-miR-141-3p (Previous ID: hsa-miR-141) |
| hsa-miR-146a-5p |
| hsa-miR-155-5p |
| hsa-miR-221-3p |
| hsa-miR-222-3p (Previous ID: hsa-miR-222) |
| hsa-miR-31-5p |
| **GO:1904996 positive regulation of leukocyte adhesion to vascular endothelial cell** |
| hsa-miR-21-5p (Previous ID: hsa-miR-21) |
| hsa-miR-92a-3p |
| **GO:1904999 positive regulation of leukocyte adhesion to arterial endothelial cell** |
| hsa-miR-92a-3p |
| **GO:1905041 regulation of epithelium regeneration** |
| hsa-miR-29b-3p |
| **GO:1905046 positive regulation of Schwann cell proliferation involved in axon regeneration** |
| hsa-miR-221-3p |
| hsa-miR-222-3p (Previous ID: hsa-miR-222) |
| **GO:1905049 negative regulation of metallopeptidase activity** |
| hsa-miR-199b-3p / hsa-miR-199a-3p |
| hsa-miR-29b-3p |
| **GO:1905061 negative regulation of cardioblast proliferation** |
| hsa-miR-20a-5p |
| **GO:1905064 negative regulation of vascular associated smooth muscle cell differentiation** |
| hsa-miR-100-5p |
| hsa-miR-15b-5p |
| hsa-miR-221-3p |
| hsa-miR-26a-5p |
|  |
|  |
|  |
| **GO:1905065 positive regulation of vascular associated smooth muscle cell differentiation** |
| hsa-miR-124-3p |
| hsa-miR-125b-5p |
| hsa-miR-21-5p (Previous ID: hsa-miR-21) |
| **GO:1905095 negative regulation of apolipoprotein A-I-mediated signaling pathway** |
| hsa-miR-19b-3p (Previous ID: hsa-miR-19b) |
| **GO:1905101 negative regulation of apoptosome assembly** |
| hsa-miR-17-5p (Previous ID: hsa-miR-17) |
| **GO:1905111 positive regulation of pulmonary blood vessel remodeling** |
| hsa-miR-143-3p |
| hsa-miR-17-5p (Previous ID: hsa-miR-17) |
| hsa-miR-20a-5p |
| **GO:1905149 positive regulation of smooth muscle hypertrophy** |
| hsa-miR-17-5p (Previous ID: hsa-miR-17) |
| **GO:1905175 negative regulation of vascular associated smooth muscle cell dedifferentiation** |
| hsa-miR-145-5p |
| hsa-miR-182-5p (Previous ID: hsa-miR-182) |
| **GO:1905176 positive regulation of vascular associated smooth muscle cell dedifferentiation** |
| hsa-miR-214-3p |
| hsa-miR-221-3p |
| **GO:1905180 positive regulation of cardiac muscle tissue regeneration** |
| hsa-miR-199b-3p / hsa-miR-199a-3p |
| hsa-miR-590-3p |
| **GO:1905203 regulation of connective tissue replacement** |
| hsa-miR-199a-5p |
| hsa-miR-199b-5p (Previous ID: hsa-miR-199b) |
| **GO:1905205 positive regulation of connective tissue replacement** |
| hsa-miR-155-5p |
| hsa-miR-15b-5p |
| hsa-miR-16-5p |
| hsa-miR-195-5p (Previous ID: hsa-miR-195) |
| hsa-miR-199a-5p |
| hsa-miR-214-3p |
| hsa-miR-34a-5p |
| hsa-miR-34c-5p (Previous ID: hsa-miR-34c) |
| **GO:1905206 positive regulation of hydrogen peroxide-induced cell death** |
| hsa-miR-34a-5p |
| **GO:1905241 positive regulation of canonical Wnt signaling pathway involved in osteoblast differentiation** |
| hsa-miR-29b-3p |
| **GO:1905299 negative regulation of intestinal epithelial cell development** |
| hsa-miR-29b-3p |
| **GO:1905460 negative regulation of vascular associated smooth muscle cell apoptotic process** |
| hsa-miR-138-5p |
| hsa-miR-17-5p (Previous ID: hsa-miR-17) |
| hsa-miR-21-5p (Previous ID: hsa-miR-21) |
| **GO:1905461 positive regulation of vascular associated smooth muscle cell apoptotic process** |
| hsa-miR-24-3p |
| **GO:1905522 negative regulation of macrophage migration** |
| hsa-miR-24-3p |
| **GO:1905562 regulation of vascular endothelial cell proliferation** |
| hsa-miR-150-5p (Previous ID: hsa-miR-150) |
| hsa-miR-200c-3p (Previous ID: hsa-miR-200c) |
| **GO:1905563 negative regulation of vascular endothelial cell proliferation** |
| hsa-miR-129-5p |
| hsa-miR-152-3p |
| hsa-miR-15b-5p |
| hsa-miR-193a-5p |
| hsa-miR-20b-5p (Previous ID: hsa-miR-20b) |
| hsa-miR-24-3p |
| hsa-miR-30b-5p (Previous ID: hsa-miR-30b) |
| hsa-miR-30e-5p |
| hsa-miR-34a-3p (Previous ID: hsa-miR-34a*) |
| hsa-miR-424-5p (Previous ID: hsa-miR-424) |
| hsa-miR-492 |
| hsa-miR-503-5p (Previous ID: hsa-miR-503) |
| hsa-miR-98-5p |
| **GO:1905564 positive regulation of vascular endothelial cell proliferation** |
| hsa-miR-126-5p |
| hsa-miR-135b-5p |
| hsa-miR-130a-3p (Previous ID: hsa-miR-130a) |
| hsa-miR-132-3p (Previous ID: hsa-miR-132) |
| hsa-miR-21-5p (Previous ID: hsa-miR-21) |
| hsa-miR-27a-3p |
| hsa-miR-29a-3p |
| **GO:1905596 negative regulation of low-density lipoprotein particle receptor binding** |
| hsa-miR-148a-3p |
| hsa-miR-27b-3p |
| **GO:1905598 negative regulation of low-density lipoprotein receptor activity** |
| hsa-miR-27a-3p |
| **GO:1905601 negative regulation of receptor-mediated endocytosis involved in cholesterol transport** |
| hsa-miR-17-5p (Previous ID: hsa-miR-17) |
| hsa-miR-27b-3p |
| **GO:1905608 positive regulation of presynapse assembly** |
| hsa-miR-431-5p (Previous ID: hsa-miR-431) |
| **GO:1905651 regulation of artery morphogenesis** |
| hsa-miR-153-3p |
| **GO:1905710 positive regulation of membrane permeability** |
| hsa-miR-142-5p |
| **GO:1905772 positive regulation of mesodermal cell differentiation** |
| hsa-miR-150-5p (Previous ID: hsa-miR-150) |
| hsa-miR-200c-3p (Previous ID: hsa-miR-200c) |
| **GO:1905884 negative regulation of triglyceride transport** |
| hsa-miR-30c-5p |
| **GO:1905932 positive regulation of vascular associated smooth muscle cell differentiation involved in phenotypic switching** |
| hsa-miR-18a-5p (Previous ID: hsa-miR-18a) |
| **GO:1905952 regulation of lipid localization** |
| hsa-miR-155-5p |
| **GO:1905955 negative regulation of endothelial tube morphogenesis** |
| hsa-miR-21-5p (Previous ID: hsa-miR-21) |
| **GO:2000134 negative regulation of G1/S transition of mitotic cell cycle** |
| hsa-miR-138-5p |
| hsa-miR-15a-5p (Previous ID: hsa-miR-15a) |
| hsa-miR-15b-5p |
| hsa-miR-16-5p |
| hsa-miR-193a-3p |
| hsa-miR-21-5p (Previous ID: hsa-miR-21) |
| hsa-miR-26a-5p |
| hsa-miR-29a-3p |
| hsa-miR-29b-3p |
| hsa-miR-29c-3p |
| hsa-miR-362-3p |
| hsa-miR-638 |
| hsa-miR-892b |
| **GO:2000270 negative regulation of fibroblast apoptotic process** |
| hsa-miR-24-3p |
| **GO:2000271 positive regulation of fibroblast apoptotic process** |
| hsa-miR-181b-5p |
| **GO:2000318 positive regulation of T-helper 17 type immune response** |
| hsa-miR-21-5p (Previous ID: hsa-miR-21) |
| **GO:2000321 positive regulation of T-helper 17 cell differentiation** |
| hsa-miR-21-5p (Previous ID: hsa-miR-21) |
| **GO:2000339 negative regulation of chemokine (C-X-C motif) ligand 1 production** |
| hsa-miR-155-5p |
| **GO:2000342 negative regulation of chemokine (C-X-C motif) ligand 2 production** |
| hsa-miR-146a-5p |
| hsa-miR-766-3p (Previous ID: hsa-miR-766) |
| **GO:2000351 regulation of endothelial cell apoptotic process** |
| hsa-miR-106b-5p (Previous ID: hsa-miR-106b) |
| **GO:2000352 negative regulation of endothelial cell apoptotic process** |
| hsa-miR-30b-5p (Previous ID: hsa-miR-30b) |
| hsa-miR-30e-5p |
| hsa-miR-590-5p |
| **GO:2000353 positive regulation of endothelial cell apoptotic process** |
| hsa-miR-101-3p |
| hsa-miR-125a-5p |
| hsa-miR-138-5p |
| hsa-miR-15a-5p (Previous ID: hsa-miR-15a) |
| hsa-miR-24-3p |
| hsa-miR-375-3p |
| **GO:2000377 regulation of reactive oxygen species metabolic process** |
| hsa-miR-21-5p (Previous ID: hsa-miR-21) |
| **GO:2000425 regulation of apoptotic cell clearance** |
| hsa-miR-155-5p |
| **GO:2000545 negative regulation of endothelial cell chemotaxis to fibroblast growth factor** |
| hsa-miR-149-3p |
| hsa-miR-149-5p (Previous ID: hsa-miR-149) |
| hsa-miR-16-5p |
| hsa-miR-424-5p (Previous ID: hsa-miR-424) |
| **GO:2000660 negative regulation of interleukin-1-mediated signaling pathway** |
| hsa-miR-21-3p |
| hsa-miR-27a-5p |
| **GO:2000670 positive regulation of dendritic cell apoptotic process** |
| hsa-miR-155-5p |
| **GO:2000724 positive regulation of cardiac vascular smooth muscle cell differentiation** |
| hsa-miR-145-5p |
| **GO:2000725 regulation of cardiac muscle cell differentiation** |
| hsa-miR-155-5p |
| **GO:2000726 negative regulation of cardiac muscle cell differentiation** |
| hsa-miR-200b-3p (Previous ID: hsa-miR-200b) |
| hsa-miR-222-3p (Previous ID: hsa-miR-222) |
| hsa-miR-590-5p |
| **GO:2000727 positive regulation of cardiac muscle cell differentiation** |
| hsa-miR-204-5p |
| **GO:2000736 regulation of stem cell differentiation** |
| hsa-miR-146a-5p |
| **GO:2000773 negative regulation of cellular senescence** |
| hsa-miR-17-3p (Previous ID: hsa-miR-17*) |
| hsa-miR-543 |
| hsa-miR-590-3p |
| **GO:2000774 positive regulation of cellular senescence** |
| hsa-miR-10a-3p (Previous ID: hsa-miR-10a*) |
| hsa-miR-146a-5p |
| hsa-miR-20b-5p (Previous ID: hsa-miR-20b) |
| hsa-miR-22-3p |
| hsa-miR-34a-5p |
|  |
|  |
| **GO:2000780 negative regulation of double-strand break repair** |
| hsa-miR-221-3p |
| **GO:2001198 regulation of dendritic cell differentiation** |
| hsa-miR-223-3p (Previous ID: hsa-miR-223) |
| **GO:2001235 positive regulation of apoptotic signaling pathway** |
| hsa-miR-26b-5p (Previous ID: hsa-miR-26b) |
| hsa-miR-449a (Previous ID: hsa-miR-449) |
| **GO:2001241 positive regulation of extrinsic apoptotic signaling pathway in absence of ligand** |
| hsa-miR-198 |
| **GO:2001243 negative regulation of intrinsic apoptotic signaling pathway** |
| hsa-miR-132-3p (Previous ID: hsa-miR-132) |
| hsa-miR-17-5p (Previous ID: hsa-miR-17) |
| **GO:2001244 positive regulation of intrinsic apoptotic signaling pathway** |
| hsa-miR-15a-5p (Previous ID: hsa-miR-15a) |
| hsa-miR-16-5p |
| hsa-miR-27b-5p (Previous ID: hsa-miR-27b*) |
